# Utilizing Estra-1,3,5,16-Tetraene Scaffold: Design and Synthesis of Nitric Oxide Donors as Chemotherapeutic Resistance Combating Agents in Liver Cancer

**DOI:** 10.3390/molecules28062754

**Published:** 2023-03-18

**Authors:** Mahrous A. Abou-Salim, Mohamed A. Shaaban, Mohammed K. Abd El Hameid, Mohammed M. Alanazi, Fathi Halaweish, Yaseen A. M. M. Elshaier

**Affiliations:** 1 Department of Pharmaceutical Organic Chemistry, Faculty of Pharmacy, Al-Azhar University, Assiut 71524, Egypt; 2 Department of Pharmaceutical Organic Chemistry, Faculty of Pharmacy, Cairo University, Cairo 11562, Egypt; 3 Department of Pharmaceutical Chemistry, College of Pharmacy, King Saud University, Riyadh 11451, Saudi Arabia; 4 Department of Chemistry and Biochemistry, South Dakota State University, Box 2202, Brookings, SD 57007, USA; 5 Department of Organic and Medicinal Chemistry, Faculty of Pharmacy, University of Sadat City, Menoufia 32958, Egypt

**Keywords:** cucurbitacin, MDR, furoxan, HCC, MRP2, DAF-FM DA, OpenEye, estrone

## Abstract

A new series of nitric oxide-releasing estra-1,3,5,16-tetraene analogs (NO-∆-16-CIEAs) was designed and synthesized as dual inhibitors for EGFR and MRP2 based on our previous findings on estra-1,3,5-triene analog NO-CIEA **17** against both HepG2 and HepG2-R cell lines. Among the target compounds, **14a** (*R*-isomer) and **14b** (*S*-isomer) displayed potent *anti*-proliferative activity against both HepG2 and HepG2-R cell lines in comparison to the reference drug erlotinib. Remarkably, compound **14a** resulted in a prominent reduction in EGFR phosphorylation at a concentration of 1.20 µM with slight activity on the phosphorylation of MEK1/2 and ERK1/2. It also inhibits MRP2 expression in a dose-dependent manner with 24% inhibition and arrested the cells in the S phase of the cell cycle. Interestingly, compound **14a** (estratetraene core) exhibited a twofold increase in *anti*-proliferative activity against both HepG2 and HepG2-R in comparison with the lead estratriene analog, demonstrating the significance of the designed ∆-16 unsaturation. The results shed a light on compound **14a** and support further investigations to combat multidrug resistance in chemotherapy of hepatocellular carcinoma patients.

## 1. Introduction

Hepatocellular carcinoma (HCC) is one of the most frequent and lethal human cancers, and its incidence-to-mortality ratio is rising [1,2,3,4,5,6]. In 2020, HCC represented 2.4% of all new cancer cases and 5.0% of all cancer deaths [4]. The C subset of the human ATP-binding cassette (ABCC) transporters contains a huge group of membrane transport proteins comprising thirteen subfamily members (1–13) and plays a pivotal role in the recognition and transport of most drugs. Among them, the second member of multidrug resistance protein 2 (MRP2) of the MRP subfamily of the encoded ABCC2 gene located on Chromosome 10q23-q24 with a unique amino acid sequence of the ATP-binding domain, which functions properly in drug resistance mechanisms as it is structurally similar to human MRP1 [7]. MRP2 is overexpressed in many cancers such as the liver, kidney, and small intestine to play a key role in the transport of many drugs such as cisplatin, doxorubicin, epirubicin, etoposide, irinotecan, mitoxantrone, methotrexate, SN-38, and Vinca alkaloids, in addition to endogenous metabolites. Korita and co-workers [8] and our research group [9] recently reported that HCC cells produce drug resistance via MRP2 overexpression, which comprises the major problem associated with the available chemotherapeutic agents in 30–80% of cancer patients [10,11]. Furthermore, the EGFR-MEK1/2-ERK1/2 signaling pathway, as well as MRP2, has been validated as effective targets for liver cancer therapy. So far, the discovered agents have revealed significant activity toward MRP1 and P-gp but rarely against MRP2 [8]. Therefore, it is still important to discover a dual inhibitor drug candidate with good efficacy and low toxicity targeting the EGFR-MEK1/2-ERK1/2 signaling pathway as well as MRP2.

**Figure 1:**
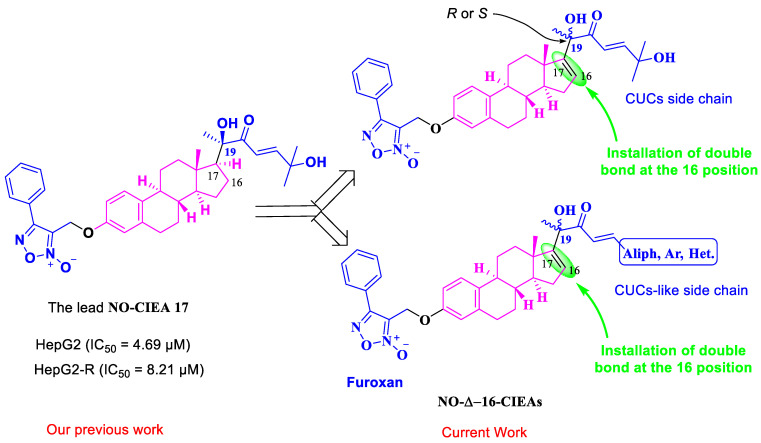
Design strategy for target compounds NO-∆-16-CIEAs.

It is worth mentioning that the role of NO in cancer therapy is not only that of enhancing tumor blood flow and oxygen supply [12] but also reversing chemotherapy resistance through the upregulation of efflux pumps [13,14]. Along this line of research, Maksimovic-Ivanic et al. demonstrated that the hybridization of some drugs with NO, such as NO-NSAIDs and NO-HIV-PIs, promoted anticancer activity in a wide range of cancer cell lines and in in vivo models, invariably more potent, less toxic, lower dosing, and fewer side effects than the corresponding des-NO analogs [15]. The mode of action is likely multifactorial as they inhibit tumor cell growth, induce apoptosis, exhibit antiangiogenic and antimetastatic activity, and potentially inhibit P-gp-, MRP-1-, and BCRP1-expressing cells making NO-releasing agents as candidates for the treatment of multidrug-resistant tumors [15]. Previously, we detailed the biological studies of NO-releasing cucurbitacin analogs with an estratriene core [16]. Among them, NO-CIEA **17** (Figure 1), exhibits broad-spectrum anti-proliferative activity against both HepG2 and HepG2-R cell lines with IC_50_ 4.69 and 8.21 µM, respectively [16]. Therefore, overcoming the toxicity and cellular resistance problems encouraged us to look for a more active molecular target anticancer agent with nitric oxide-releasing properties for the treatment of HCC.

Enlightened by the aforementioned and the activity profile displayed by NO-CIEA **17** [16], we explored whether further D-ring modification to rigidify the conformations of the estron skeleton would adopt strong interaction inside the target active sites to obtain the highest potency. To this end, a new series of ∆-16 unsaturated analogs NO-∆-16-CIEAs (Figure 1) was designed, allowing us to establish that the estratetraene core could successfully function as a bioisostere for the estratriene one keeping the NO-releasing furoxan moiety at C-3, Cucurbitacin-like side chain at C-17, and altering the configuration around C-19 in the hope of discovering even more potent compounds.

## 2. Results and Discussion

### 2.1. Chemistry

**Scheme 1:**
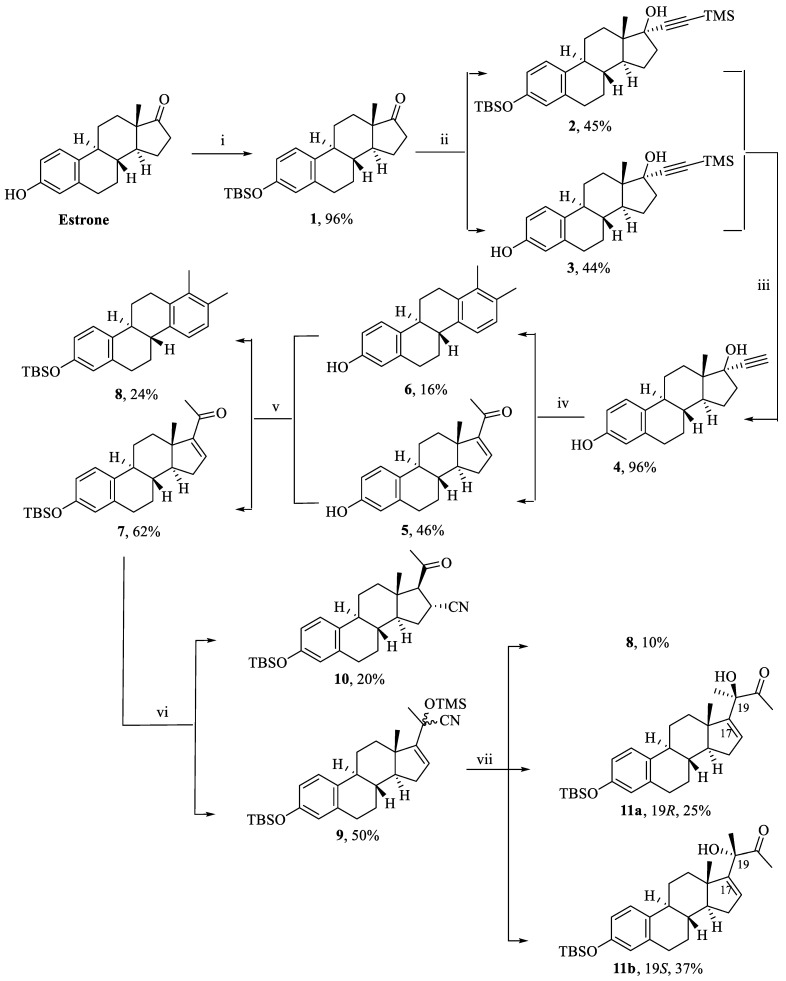
Synthesis of key intermediates α-hydroxy methyl ketones **11a**,**b**: (i) TBSCl, imidazole, dry DMF, 12 h; (ii) trimethylsilylacetylene, −20 °C, *n*-BuLi, 30 min, THF, 0 °C–r.t., 3.5 h; (iii) TBAF, THF, r.t., 12 h; (iv) Cu(OTf)_2_, dry EtOAc, 100 °C, 4 h; (v) TBSCl, imidazole, dry DMF, 12 h; (vi) TMSCN, ZnI_2_, THF, −20 °C; (vii) MeLi, Et_2_O, 0 °C.

As outlined in Scheme 1, the synthesis of the key intermediate ∆-16-α-hydroxy methyl ketones (**11a**,**b**) was started with estrone protection using *tert*-butyldimethylsilyl chloride (TBSCl) to obtain compound **1**, which was then treated with lithium trimethylsilylacetylide to afford a mixture of a ratio about 1:1 of the corresponding propargylic alcohol **2** and **3**. These alcohols were deprotected with TBAF (tetra-*n*-butylammonium fluoride) to form the desired intermediate 17-α-ethynylestradiol **4** in 95.50 % yield [17,18].

Copper triflate (Cu(OTf)_2_) catalyzed regioselective Markovnikov hydration of 17-α-ethynylestradiol **4** was developed to install the targeted double bond at the 16 position as well as the carbonyl functionality at the C-17 position via Rupe rearrangement. Hassam and Li [19] reported the synthesis of α,β-enone **5** only from the corresponding alkyne **4** using 20 mol% Cu(OTf)_2_ as a catalyst in ethyl acetate–water mixture under reflux for 2 h. On exposing the catalytic system with one equivalent of Cu(OTf)_2_ and refluxing in ethyl acetate for 4 h, compound **6** was observed along with α,β-enone **5** in a yield of 15.50 and 46.48%, respectively [20,21,22]. The yield was reversed when the equivalence of Cu(OTf)_2_ was decreased to 20 mol% in the presence or absence of the catalytic amount of sulfuric acid under reflux. Further, the same results were obtained when the reaction was refluxed and catalyzed with sulfuric acid in either formic or acetic acid. Hence, the formation of aryl derivative **6** is favorable in strong acidic conditions [23]. Furthermore, the crude mixture of α,β-enone **5** and aryl derivative **6** underwent a reaction with TBSCl to protect the phenolic hydroxyl group, hence overcoming the solubility problems through the formation of compounds **7** and **8**, respectively.

Compared to other cyano donors, such as acetone cyanohydrin or hydrogen cyanide, TMSCN has the advantage of catalyzing the irreversible addition of CN to the carbonyl electrophile. Furthermore, the large trimethylsilyl group provides some steric hindrance, which can be useful to hold stereopreference for one of the two possible diastereomers [22]. The catalysts used for the addition of TMSCN to α,β-unsaturated enone derivatives, i.e., ZnI_2_, TiCl_4_, Yb(OTf)_3_, LiOEt, Et_3_N, and 4-DMAP, have previously been shown to catalyze cyanohydrins synthesis [24]. As depicted in Table 1, the addition of TMSCN to the α,β-unsaturated enone **8** was dependent on both the catalyst used and the reaction temperature. Cyanosilylation product **9** (1,2-adduct) is favored at low temperature (−20 °C) in the presence of Lewis acids, either one equivalent ZnI_2_ or 5 mol% Cu(OTf)_2_ [25,26]. Conversely, either the neutral (lithium ethoxide) or basic (potassium cyanide in 18-crown-6) catalysts at room temperature were able to promote the cyanation reaction towards the formation of 16-cyano derivative **10** (1,4-adduct). Furthermore, either decreasing the temperature up to −78 °C or proceeding without a catalyst, only resulted in the recovery of unreacted starting material [24,27,28,29,30]. Finally, the enone system (substrate **8**) is planar concerning the D-ring and, thus, less constrained by the C-18 methyl group, allowing the approach of the reagent by both sides of the steroid molecule. Consequently, the ZnI_2_-catalysed addition of TMSCN yielded a mixture of epimeric cyanosilylated product **9** (50% yield), as observed by NMR, as well as 16-α-cyano analog **10** (20% yield) [24]. This lack of chemo selectivity in the ZnI_2_-catalysed TMSCN addition was not surprising. In fact, although the reported ZnI_2_ catalyzed 1,2-addition to conjugated enones, 1,4-addition was also noticed [24].

More specifically, the assignment of the 16-configuration of the cyano group was made based on the ^1^H NMR anisotropic shielding effect of this functional group toward the 18-CH_3_, which appears as a singlet peak at 0.42 ppm, in contrast with 0.73 ppm in the starting compound **8**. Such an effect suggests that the methyl group is positioned in the conical area of the cyano substituent. In agreement with this observation, the coupling constant of the doublet due to the 17-H (2.79 ppm) is relatively high, *J* = 8.7 Hz, pointing to a *trans*-vicinal coupling with the 16-H (3.41 ppm) [24].

**Figure 2:**
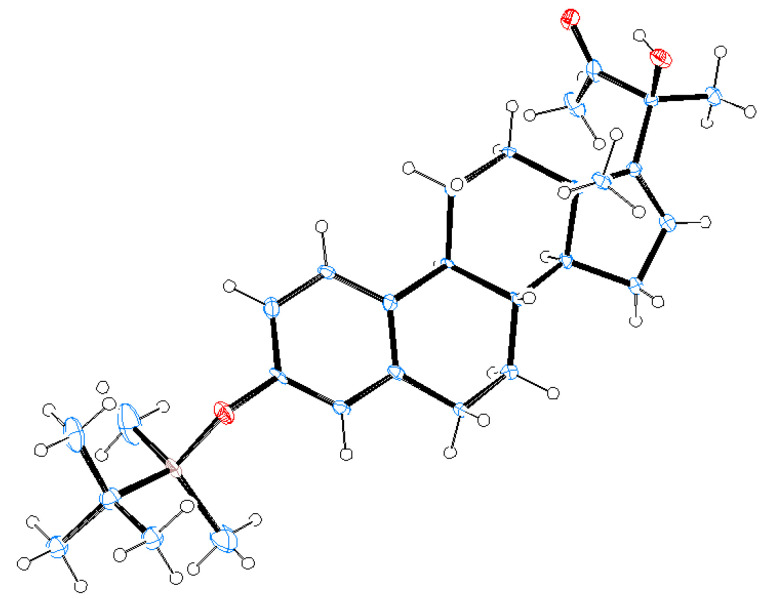
X-ray crystal structure of **11a** showing the *R*-configuration around C-19 [31].

Moreover, the mixture of the key intermediate **11a** and **11b** was isolated from the reaction of epimeric cyanosilylated **9** with methyl lithium in ether at 0 °C. This diastereomeric mixture with ∆R*_f_* equal to 0.1 was then separated by column chromatography (5% ethyl acetate in hexane) and, then, identified as (19*R*)-α-hydroxy methyl ketone (**11a**, 24.72% yield) and (19*S*)-α-hydroxy methyl ketone (**11b**, 36.78% yield). The assignment of the C-19 configuration of the hydroxyl group was made on the basis of ^1^H NMR and ^13^C NMR chemical shifts in this functional group as well as X-ray crystallographic analysis (Figure 2), and these data were compared to a CUCs side chain containing the function group reported previously by our research group [31]. ^1^H NMR showed a singlet signal at 4.07 ppm and 3.90 ppm corresponding to **11a** and **11b**, respectively. Surprisingly, the protected α,β-enone **8** was also detected in the mixture in 10.20% yield with an unknown mechanism.

**Scheme 2:**
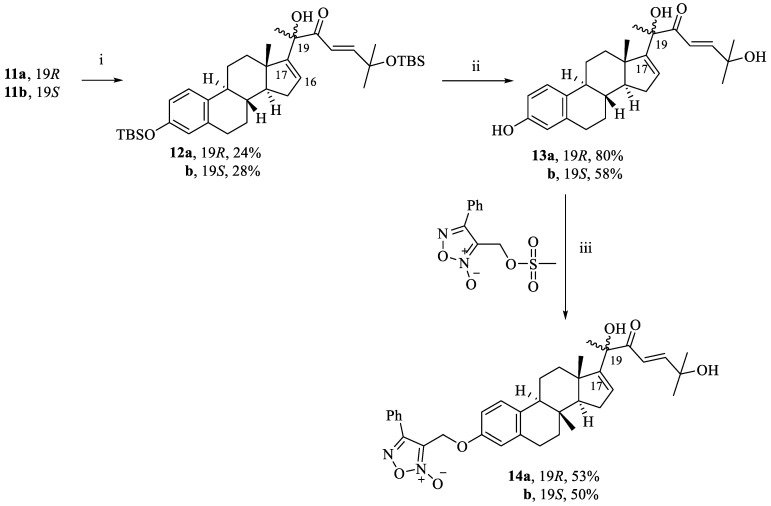
Synthesis of NO-∆-16-CIEAs **14a**,**b**: (i) LDA, THF, −78 °C, 1 h, 2-((*tert*-butyldimethylsilyl)oxy)-2-methylpropanal, −78 °C to r.t., 20 h; (ii) TBAF, THF, r.t., 12 h; (iii) NaOH, THF, 3-(((methylsulfonyl)oxy)methyl)-4-phenyl-1,2,5-oxadiazole 2-oxide, reflux, 2 h.

As illustrated in Scheme 2, the precursor 2-((*tert*-butyldimethylsilyl)oxy)-2-methylpropanal was synthesized as reported [31] and subjected to aldol condensation with the key intermediates α-hydroxymethylketones **11a**,**b** using LDA to afford the desired cucurbitacin-inspired estra-1,3,5,16-tetraene analogs **12a**,**b**. Then, they underwent deprotection to provide the phenolic derivatives **13a**,**b**, which were coupled with furoxan mesylate [16,32] to afford the target cucurbitacin-inspired estra-1,3,5,16-tetraene furoxan analogs (NO-CIETA) **14a**,**b**.

**Scheme 3:**
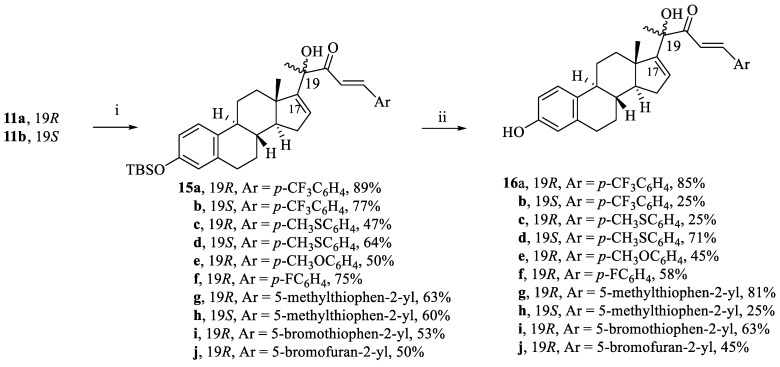

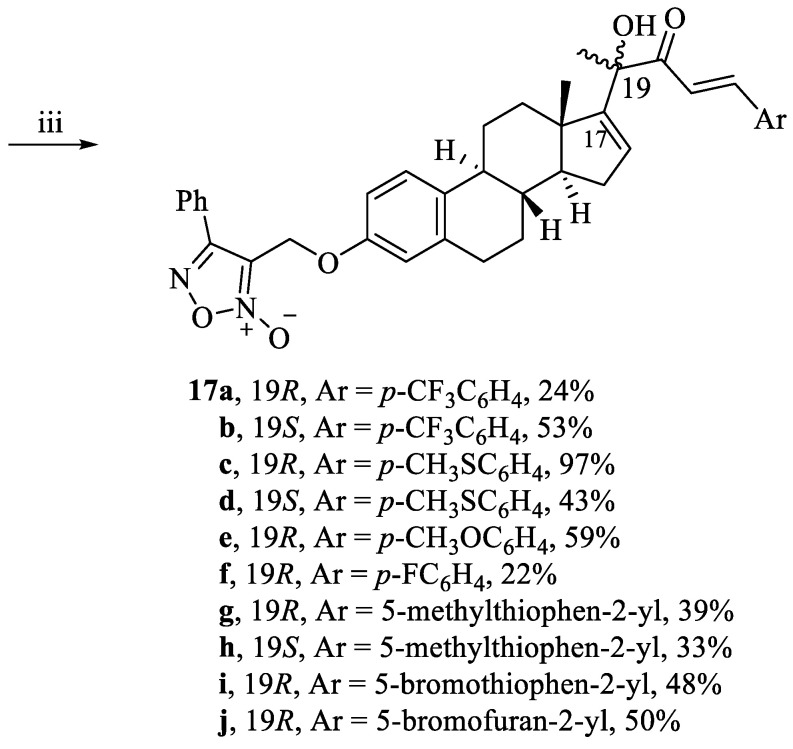
Synthesis of NO-∆-16-CIEA with aromatic/heterocyclic side chains: (i) ArCHO, NaOH, THF, 105 °C, 10–30 min; (ii) TBAF, THF, r.t., 12 h; (iii) 3-(((methylsulfonyl)oxy)methyl)-4-phenyl-1,2,5-oxadiazole 2-oxide, NaOH, THF, reflux, 2 h.

For more study about the SAR, the key intermediates α-hydroxymethylketones **11a**,**b** were then reacted with aromatic or heterocyclic aldehydes through aldol condensation and in situ elimination using NaOH to afford cucurbitacin-inspired estra-1,3,5,16-tetraene analogs **15a**–**j**. Deprotection and finally coupling with furoxan mesylate provided the target compounds **17a**–**j**, Scheme 3.

**Scheme 4:**
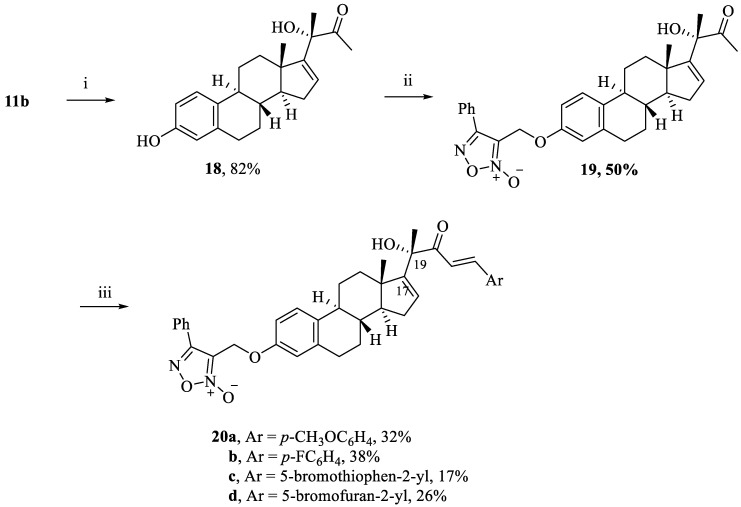
Modified pathway for the preparation of some target compounds: (i) TBAF, THF, r.t., 12 h; (ii) 3-(((methylsulfonyl)oxy)methyl)-4-phenyl-1,2,5-oxadiazole 2-oxide, NaOH, THF, reflux, 2 h. (iii) ArCHO, NaOH, THF, 105 °C, 10–30 min.

There are some attempts to improve the yield percentages for the target compounds; among them, deprotection of **11b** with TBAF and then *O*-alkylation with furoxan mesylate followed by aldol condensation with different aromatic or heterocyclic aldehydes to afford the desired compounds **20a**–**d**, as described in Scheme 4.

### 2.2. Biological Activity

#### 2.2.1. In Vitro Anti-Proliferative Activity

The target compounds **14a**,**b**, **17a**–**j**, and **20a**–**d** were examined for their in vitro *anti*-proliferative activity against HepG2 and HepG2-R (erlotinib-resistant [9]) liver cell lines. The cells were treated with serial dilutions of test compounds for 48 h, after which the cell viability was determined by a standard MTT colorimetric assay, using erlotinib as a reference drug.

As shown in Table 2, the results against HepG2 cells indicated that compound **14a** (IC_50_ = 2.40 ± 0.44 µM) was found to be the most potent analog among the tested compounds, being ten times more active than the reference drug erlotinib. Moreover, compounds **14b** and **17a** possessed higher *anti*-proliferative activities than the reference drug with IC_50_ 8.10 ± 0.92 and 9.44 ± 0.44 µM, respectively. In addition, compounds **17g** and **17h** were almost equipotent to erlotinib with IC_50_ 24.00 ± 3.80 and 25.00 ± 3.12 µM, respectively. Furthermore, compounds **17b**–**f**, **17i**, **17j**, and **20a**–**d** did not inhibit the proliferation of HepG2 cells at concentrations as high as 50 µM. Moreover, compounds linked with an aromatic or heterocyclic side chain at the C-17 position dramatically dropped or totally abolished the anticancer activity unlike compound **14a**.

Then, our attention was directed to screen our designed compounds against the HepG2-R cell line. The data revealed that analogs with cucurbitacin side chain **14a** and **14b** only have a potent cytotoxicity activity (IC_50_ 3.74 ± 0.66 µM and 6.47 ± 1.41 µM, respectively). Therefore, they may have a good impact on chemotherapeutic resistance.

Our consideration was then extended to examine the effect of compiling the furoxan ring with the estrone core as two pharmacophoric features. Accordingly, NO-releasing furoxan alcohol and cucurbitacin steroidal phenolic derivative **13a** and **13b** were examined (Table 3). The data revealed that compound **13a** showed IC_50_ 18.87 ± 2.40 and 24.22 ± 0.72 µM in HepG2 and HepG2-R cells, respectively, while compounds furoxan alcohol and **13b** did not inhibit the proliferation of both cell lines at concentrations as high as 50 µM. The results indicated that sp^2^-hybridization favored anticancer activity and consequently improved the *anti*-proliferative activity of estratetraene derivative dramatically by blocking the estrogenic activity [34] of C3 phenolic hydroxy.

Furthermore, some important physicochemical parameters were calculated to examine the drug-likeness properties of these compounds [35]. The lipophilicity parameter indicated by CLogP and the polar surface area (PSA) was estimated. As shown in Table 2, compounds **14a** and **14b** possesses good CLogP (6.39) and PSA (118.25) values among the target final analogs.

#### 2.2.2. Structure-Activity Relationships (SARs) Study

Substitution at CUCs aromatic-like side chain with either electron-withdrawing (EWG) CF_3_ and Br (i.e., compounds **17b**, **17i**,**j**, and **20c**,**d**) or electron-donating (EDG) CH_3_S and CH_3_O groups, as in compounds **17c**–**e** and **20a**, had no significant effect on the anticancer activity. Notably, the EWG group F substitution at the CUCs aromatic-like side chain (i.e., compound **20b**) may impact the activity. In contrast, CF_3_ substitution caused compound **17a** to exhibit significant inhibitory effects in its anticancer activity, indicating that the electron-withdrawing substituents likely led to enhanced potency. On the other hand, replacing both EDG and EWG with a polar *t*-BuOH group caused compounds **14a** and **14b** to exhibit significant inhibitory effects in their anticancer activity, indicating that appending the CUCs side chain at C-17 likely led to enhanced potency. Furthermore, the results showed a significant improvement in anticancer activity, especially for compounds **14a** and **14b**, compared to des-NO-releasing compounds **13a** and **13b** indicating synergistic efficiency.

**Figure 3:**
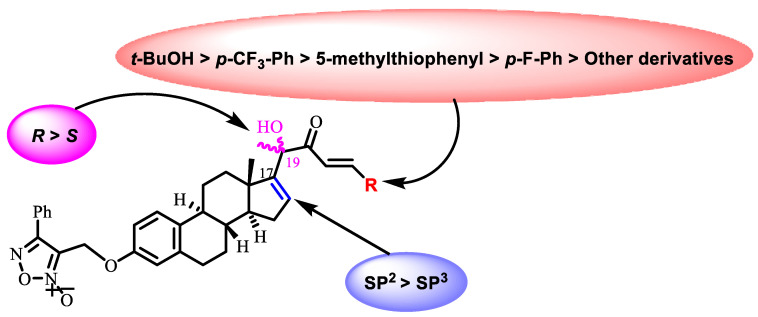
SAR of target compounds.

It is worth mentioning that the configuration around the C-19 played a crucial role in the biological activity among the most active compounds. Particularly, compound **14a** with *R*-configuration showed higher activity than the corresponding *S*-isomer **14b** in both HepG2 and HepG2-R cell lines. Moreover, compound **17a** with *R* configuration around C-19 showed a potent cytotoxicity activity against the HepG2 cell line while the corresponding *S*-isomer **17b** was inactive up to 50 µM. In addition, the anticancer activity showed a twofold increase against both the HepG2 and HepG2-R cell lines when the estratriene core in NO-CIEA **I** was replaced with estratetraene core in compound **14a**, demonstrating the significance of ∆-16 unsaturation. Appending the furoxan moiety at C3, and utilizing the CUCs side chain at C-17, ring D unsaturation at C-16, and *R* configuration around C-19 can be suggested as vital elements for the growth inhibitory activity of the hybrids reported here, see Figure 3. In this context, the synthesized analogs are considered promising candidates for the treatment of HCC and overcoming the chemotherapeutic resistance.

#### 2.2.3. Cell-Based ELISA

**Figure 4:**
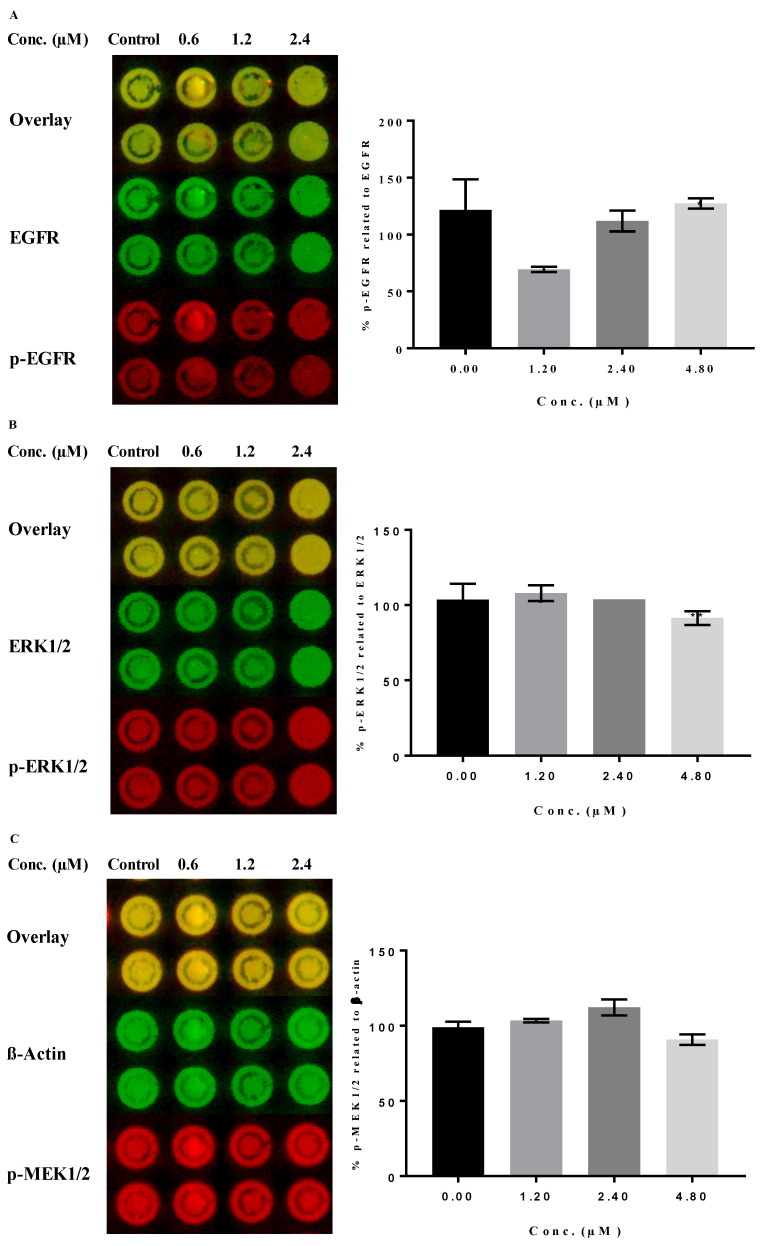

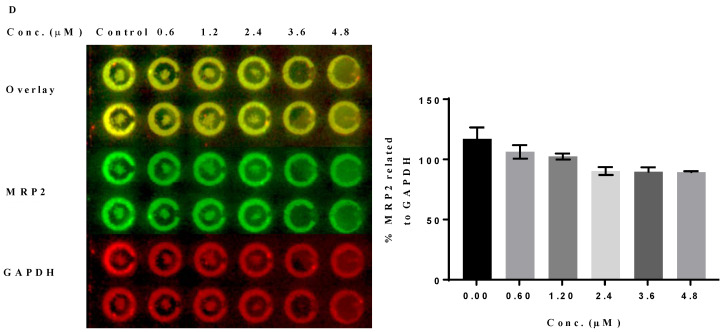
In-Cell Western analysis of predicted protein targets of NO-∆-16-CIEA **14a**: (**A**) Analysis of EGFR phosphorylation in HepG2 cells; ICW plate image and p-EGFR intensity in 800 channel was normalized to total EGFR intensity in 700 channel. (**B**) Analysis of ERK1/2 phosphorylation in HepG2 cells; ICW plate image and p-ERK1/2 intensity in 800 channel was normalized to total ERK1/2 intensity in 700 channel. (**C**) Analysis of MEK1/2 phosphorylation in HepG2 cells; ICW plate image and p-MEK1/2 intensity in 700 channel was normalized to β-actin intensity in 800 channel. (**D**) Analysis of MRP2 inhibition in HepG2-R cells; ICW plate image and MRP2 intensity in 800 channel was normalized to GAPDH intensity in 700 channel. Values reported are averages ± SD of at least two measurements.

As compound **14a** showed a good profile of *anti*-proliferative activity against both HepG2 and HepG2-R cancer cell lines, the study was extended to explore its mode of action using the In-cell Western assay (ICW). As shown in Figure 4, the Odyssey image shows the detection of total proteins regardless of phosphorylation status as well as the detection of decreasing amounts of phospho-protein as a function of increasing **14a** concentrations, while the graphical representations show the quantification of fluorescence. The data reveal the slight activity for compound **14a** after incubation for 24 h at a concentration of 4.80 µM to inhibit the phosphorylation of MEK1/2 and ERK1/2 (≈10% and 12% inhibition, respectively) compared to untreated cells (Control), whereas no effects were observed for phosphorylated EGFR. Surprisingly, **14a** resulted in a significant reduction in EGFR phosphorylation in treated cells at a concentration of 1.20 µM with 43% inhibition. Furthermore, as shown in Figure 4, there is a significant reduction in MRP2 expression in the HepG2-R cell line [36] caused by compound **14a** in a dose-dependent manner with 24% inhibition compared to control, demonstrating a significant impact on the chemotherapeutic resistance.

#### 2.2.4. Flow Cytometric Study

**Figure 5:**
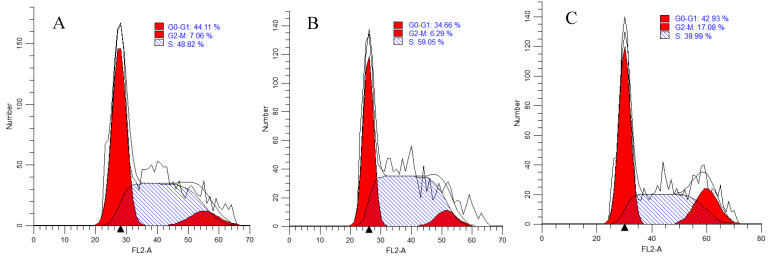
Flow cytometric analysis of cell cycle parameters: HepG2 liver cancer cells were incubated for 24 h; (**A**) control; (**B**) 2.4 µM (IC_50_) of NO-∆-16-CIEA **14a**; (**C**) 8.1 µM (IC_50_) of NO-∆-16-CIEA **14b**.

After the 24 h incubation of compounds **14a** (2.4 μM) and **14b** (8.1 μM) and vehicle DMSO as the control, quantitation of the results showed that **14a** exhibited a decrease in the fraction of cells in the G0/G1 phase (34.66% compared to 44.11% for the control) and an increase in the proportion of cells in the S phase (59.05 % compared to 48.82%) and the G2/M phase (6.29% compared to 7.06%) (Figure 5A,B). Moreover, compound **14b** also exhibited an increase in the fraction of cells in the G2/M phase (17.08% compared to 7.06% for the control) and a decrease in the proportion of cells in the S phase (39.99% compared to 48.82%) and the G0/G1 phase (42.93% compared to 44.11%) (Figure 5A,C). Therefore, compound **14a** arrested the cells in the S phase while **14b** arrested the cells in the G2/M phase of the cell cycle.

#### 2.2.5. NO Detection

**Figure 6:**
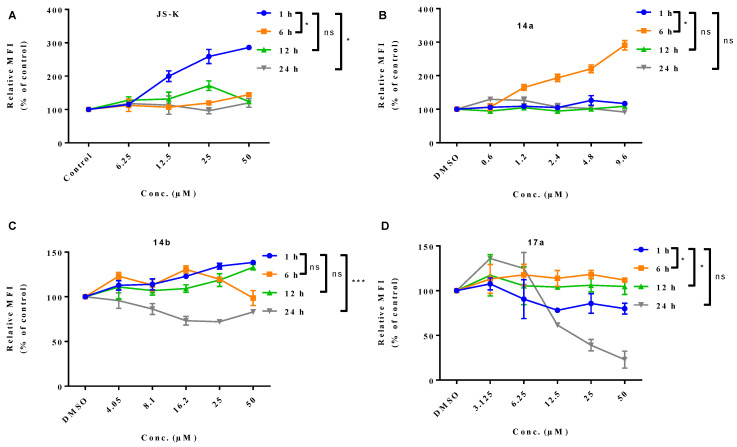
Intracellular NO release in HepG2 as measured by DAF-FM DA assay: (**A**) JS-K (reference prodrug); (**B**) NO-∆-16-CIEA **14a**; (**C**) NO-∆-16-CIEA **14b**; (**D**) NO-∆-16-CIEA **17a**. Values reported are averages ± SD of at least two measurements. * *p* ≤ 0.05, *** *p* ≤ 0.001, ns: nonsignificant.

The levels of NO produced intracellularly from compounds **14a**,**b** and **17a** along with JS-K (a known NO donor prodrug) were examined using NO-sensitive fluorophore DAF-FM DA, (Figure 6A–D). It was observed that compound **14a** showed a significant increase in fluorescence (2.9-fold compared to untreated cells) after 6 h of incubation in a dose-dependent manner and produced amounts of NO in tumor cells comparable to JS-K [16], which also showed a significant increase in fluorescence (2.9-fold compared to untreated cells) after 1 h of incubation in a dose-dependent manner. It was also found that compounds **14b** and **17a** showed a relative increase in fluorescence by about 1.38 and 1.13-fold compared to untreated cells (Control) after 1 h and 6 h of incubation in a dose-dependent manner, respectively. Furthermore, these results strongly suggest that compound **14a** is a far superior source of controlled NO release in comparison with the reference prodrug JS-K.

**Figure 7:**
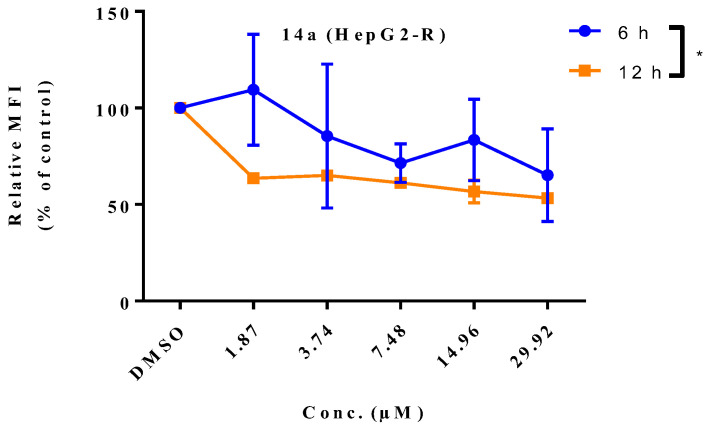
Intracellular NO release of NO-∆-16-CIEA **14a** in HepG2-R as measured by DAF-FM DA assay. Values reported are averages ± SD of at least two measurements. * *p* ≤ 0.05.

Additionally, compound **14a** was tested again for its NO-releasing activity in the HepG2-R cell line and the data revealed a marked increase in fluorescence intensity after incubation for 6 h with concentrations lower than the IC_50_. Moreover, treatment with higher concentrations than IC_50_ or a prolonged incubation showed a marked decrease in fluorescence intensity in a dose-dependent manner compared to the DMSO control (Figure 7). Therefore, the potent inhibitory effects of compound **14a** might be related to the obvious release of NO, estratetraene skeleton, and the absolute configuration of C-19 in the cucurbitacin side chain.

It is worth mentioning that our previously reported lead furoxan hybrid NO-CIEA **17** with IC_50_ values of 4.69 and 8.21 µM against both HepG2 and HepG2-R cell lines, respectively, showed an increase in fluorescence after 1 h of incubation in a dose-dependent manner and produced a significant concentration of NO in tumor cells compared to JS-K (reference drug). In addition, NO-CIEA **17** released a maximum level of NO at a concentration of 32.92 and 25 µM compared to the reference prodrug JS-K, which produced the maximum release at 50 µM.

In addition, in a dose-dependent manner, the tested compounds showed a marked decrease in NO level compared to the DMSO control after incubation for 24 h. The incubation of cells with the target compounds decreased the overall metabolic activity of the cells, as determined by MTT assay within the concentration range and incubation periods tested; thus, the low NO intracellular content at high concentrations of NO donors was caused by their cytotoxicity activity. Moreover, the DAF-FM DA probe was sufficient for detecting the increased fluorescence within a wide range of NO concentrations, as shown by the measurements after 1 h of incubation. These findings document that any potential quenching of the specific DAF-FM DA fluorescence after incubation for long periods by increased NO production at higher concentrations of the tested compound did not happen.

As a result, the NO-releasing activity showed an increase in fluorescence after 6 h of incubation with NO-∆^16^-CIEA **14a** (IC_50_: HepG2 = 2.40 µM; HepG2-R = 3.74 µM) in a dose-dependent manner, while the lead compound NO-CIEA **17** (IC_50_: HepG2 = 4.69 µM; HepG2-R = 8.21 µM) was, after 1 h, demonstrating the significance of ∆-16 unsaturation and controlled NO release.

### 2.3. Molecular Docking Studies

#### 2.3.1. Lead-Optimization and Library Design

The lead compound NO-CIEA **17**, in our previous work [16], was used for the efficient design of potent molecules using OpenEye’s EON software in lead optimization and library design. EON calculates the electrostatic similarity between the designed compounds and the existing lead compound, NO-CIEA **17**, in the form of an Electrostatic Tanimoto (ET) score [37]. The lower EON-ranked compounds have the highest shape and electrostatic similarity to the reference, as shown in Table 4.

#### 2.3.2. Structure-Based Design

**Figure 8:**
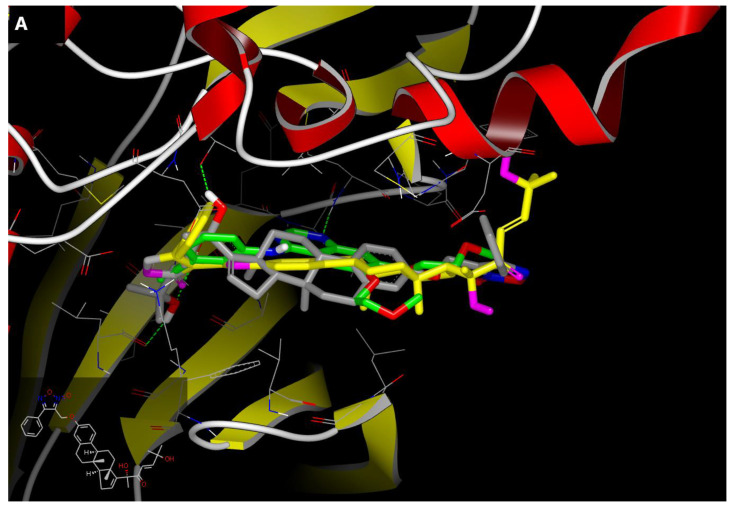

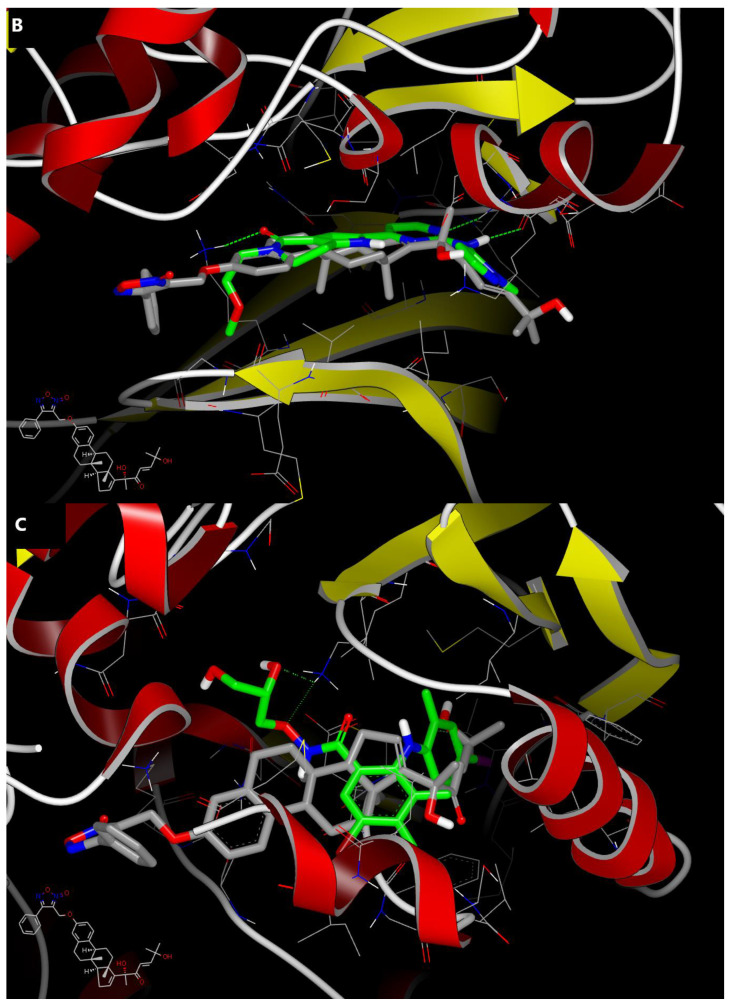

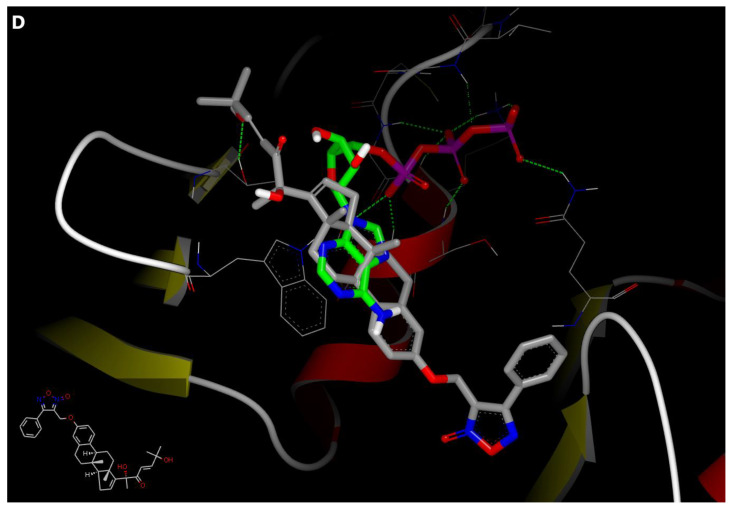
Visual representation for (**A**) erlotinib (green), **14a** (grey), and NO-CIEA **I** (yellow) overlay docked with EGFR showing hydrophobic–hydrophobic interactions and hydrogen bonds (green dash lines) towards ATP-binding site, (**B**) overlay of **14a** (grey) and co-crystallized ligand (green) docked with Erk2 showing hydrophobic–hydrophobic interactions, (**C**) overlay of **14a** (gray), and co-crystallized ligand (green) docked with MEK1 showing hydrophobic–hydrophobic interactions, (**D**) overlay of **14a** (gray) and co-crystallized ligand (green) docked with MRP1 showing hydrophobic–hydrophobic interactions and hydrogen bonds (green dash line) towards the active site.

A molecular docking study was carried out to examine the binding modes of the designed compounds in the active sites of EGFR (PDB:ID 1M17), ERK2 (PDB:ID 5NHJ), MEK1 (PDB:ID 1S9J), and MRP1 (PDB:ID 2CBZ) using the OpenEye Scientific software [38], as shown in Table 5. Among the docked compounds, **14a** scored the best Chemgauss4 scores within the target active sites [39]. As shown in Figure 8A, the FRED view of compound **14a** inside EGFR active site showed an overlay with the co-crystallized ligand erlotinib in addition to the inverted binding mode to the lead NO-CIEA **I** with three hydrogen bonding formations between (1) C-19-OH and a hydroxyl group of THR:830:A with bond length of 1.54 Å, (2) C23-OH and a hydroxyl group of THR:766:A with bond length of 2.22 Å, and (3) C23-OH and a carbonyl group of LEU:764:A with bond length of 1.59 Å. Inside the active site of both ERK2 and MEK1, compound **14a** scored the highest Chemgauss4 overlaying with the co-crystallized ligands with hydrophobic interactions as shown in Figure 8B,C, respectively. In the active site of MRP1, the compound **14a** showed an overlay with the adenosine moiety of the ATP ligand, forming a hydrogen bond coming from C23-OH and the hydroxyl group of THR:666:A with bond length of 1.91 Å (Figure 8D).

As a result, the rigidification of the D-ring through the installation of a double bond at C-16, particularly in compound **14a** with *R*-configuration around C-19, improved the binding affinity toward the tested protein active sites, which might serve as a good candidate for EGFR-ERK signal transduction inhibitors.

## 3. Materials and Methods

### 3.1. General Chemical Techniques

^1^H and ^13^C NMR spectra were acquired on a Bruker AVANCE-400 or 600 MHz NMR spectrometer, in DMSO-*d_6_*, CDCl_3,_ or (CD_3_)_2_CO using the solvent residual peak as the internal standard, with the reporting of coupling constants in Hz and the signal multiplicities are reported as singlet (s), doublet (d), triplet (t), quartet (q), doublet of doublets (dd), doublet of triplets (dt), multiplet (m), or broad (br). HRMS data were obtained using EI ionization on a ThermoFinnigan MAT 95 XL mass spectrometer at Buffalo mass spectroscopy facility, Buffalo, NY, USA. TLC analysis was performed using pre-coated silica gel PE sheets. Products were purified via column chromatography using silica gel 40–63 um (230–400 mesh) and prep-plates (Alltech preparative column, Econosil C18 10u, length 250 mm, ID 22 mm). All reagents and solvents were obtained from commercial suppliers and used as received. All chemical reactions requiring anhydrous conditions were performed with oven-dried glassware under an atmosphere of nitrogen.

Compounds **1**,**2** [40], **3** [17], **4** [18,41,42], **5** [19,43], and **6** [23] were prepared as reported.

#### 3.1.1. General Procedure A for C3 Phenolic OH Protection

To a stirred solution of crude mixture of phenolic enones **5** and **6** (0.51 mmol) in DMF (1.5 mL), imidazole (0.092 g, 1.35 mmol) and TBSCl (0.113 g, 0.75 mmol) were added. The reaction mixture was stirred for 12 h at room temperature then extracted with ethyl acetate (3 × 10 mL) and evaporated to give a brown oil. Column chromatography (10–30% ethyl acetate in hexane) was used to purify the crude products, which gives the target enone *tert*-butyldimethylsilyl ether **7** (62.20 %) as a white powder in addition to the side product **8** (24.15 %) as a white powder [23,44,45].

#### 3.1.2. General Procedure B for Cyanosilylation

To a stirred solution of enone **7** (0.131 g, 0.32 mmol) in dry dichloromethane (DCM) (1 mL) at −20 °C, TMSCN (0.053 mL, 0.42 mmol) and zinc iodide (0.102 g, 0.32 mmol) were added. The reaction mixture was stirred for 2 h at −20 °C then concentrated in vacuo. To the concentrated slurry, water was added, and then the aqueous layer was extracted using ethyl acetate (3 × 10 mL), dried over anhydrous sodium sulfate, and concentrated in vacuo. The crude product was purified using silica gel column chromatography (1% ethyl acetate in hexane) to give (0.108 g, 66.18 %) a mixture of diastereomers **9** (50 %) in the ratio about 1:0.5 (NMR detection), and a side product 16-cyano derivative **10** (20 %) as white powders [26,29].

#### 3.1.3. General Procedure C for Preparation of the Key Intermediated α-Hydroxy Ketones

To a stirred solution of diastereomers **9** (0.54 g, 1.06 mmol) in a dry diethyl ether (3.10 mL), methyl lithium (2.00 mL, 3.19 mmol) was added drop wise at 0 °C. The reaction mixture was stirred for 2 h at 0 °C, and then quenched by adding glacial acetic acid (0.40 mL) in one portion at 0 °C and allowed to stir for 30 min at 0 °C. Saturated sodium bicarbonate solution was added to neutralize the acidic mixture, and the aqueous layer was extracted by ethyl acetate (3 × 50 mL), dried under anhydrous sodium sulfate, and then concentrated under vacuo. The resulting crude products were separated by silica gel column chromatography (5% ethyl acetate in hexane) to give the hydroxyl methyl ketones **11a** (25%) and **11b** (37%) and the side product **8** (10.20%) [26].

#### 3.1.4. General Procedure D for Preparation of C-17 CUCs Side Chain Precursors

To a stirred solution of α-hydroxy ketone **11a**,**b** (0.500 g, 0.79 mmol) in THF (1.6 mL), LDA (1.42 mL, 2.83 mmol, 2 M in THF) was added at −78 °C and stirred for 1 h. A solution of 2-((*tert*-butyldimethylsilyl)oxy)-2-methylpropanal (0.3997 g, 1.98 mmol) in THF (5.3 mL) was then added at −78 °C, and the reaction mixture was allowed to slowly warm to room temperature and stirred for 20 h. The reaction mixture was then quenched by the addition of saturated NH_4_Cl (20 mL), followed by extraction with EtOAc (3 × 50 mL), dried over anhydrous sodium sulfate, and concentrated in vacuo. The crude product was purified by silica gel column chromatography with hexanes/EtOAc (9.5:0.5) to yield the desired products **12a**,**b**.

#### 3.1.5. General Procedure E for Aldol Condensation

In an open 20 mL glass vial, aromatic aldehyde derivatives (1.5 mmol) and NaOH (2.9 mmol) were added to a solution of hydroxyl methyl ketone **11a**,**b** and **19** (1 mmol) in 1 mL dry THF. The reaction mixture was heated at 110 °C for 10–45 min and THF was continuously added at a rate of ~0.5 mL per 1 min till the completion of the reaction (monitored by TLC). The reaction mixture was quenched with water and then the aqueous layer was extracted using ethyl acetate (3 × 10 mL), dried over anhydrous sodium sulfate, and concentrated under vacuo. The crude product was then purified using silica gel column chromatography (5% ethyl acetate in hexane) to yield enone products **15a**–**j** and NO-CIETA **20a**–**d** in good yields [46,47].

#### 3.1.6. General Procedure F for Deprotection

To a stirred solution of protected enone **12a**,**b**, **15a**–**j**, and **11b** (1 mmol) in THF (3 mL), TBAF (3.1 mmol) was added and stirred for 12 h. The reaction mixture was quenched with saturated ammonium chloride solution and then extracted with ethyl acetate (3 × 20 mL), dried over sodium sulfate anhydrous, filtrated, and concentrated in vacuo. Silica gel column chromatography was used to purify the crude material (15 % ethyl acetate in hexane) to afford NO-CIETA **13a**,**b**, **16a**–**j**, and **18** [18].

#### 3.1.7. General Procedure G for Coupling with Furoxan

To a stirred solution of the respective enone, **13a**,**b**, **16a**–**j**, and **18** (0.28 mmol) in THF (3 mL), furoxan mesylate (0.150 g, 0.56 mmol) and sodium hydroxide (0.011 g, 0.28 mmol) were added. The reaction mixture was heated under reflux for 1 h, then another 0.011 g (0.28 mmol) of sodium hydroxide was added and the reflux was continued for another 1 h (TLC monitoring). The reaction mixture was quenched with water and extracted with ethyl acetate (3 × 10 mL), dried over sodium sulfate anhydrous, filtrated, and concentrated under vacuo. Silica gel column chromatography was used to purify the crude material (10% ethyl acetate in hexane) to afford the titled NO-CIETA **14a**,**b**, **17a**–**j**, and **19.**

### 3.2. Chemical Synthesis and Characterization

*1-((8S,9S,13S,14S)-3-((tert-butyldimethylsilyl)oxy)-13-methyl-7,8,9,11,12,13,14,15-octahydro-6H-cyclopenta[a]phenanthren-17-yl)ethenone (***7***)*. Following general procedure, A, compounds **6** and **5** (0.51 mmol) were protected with TBSCl (0.113 g, 0.75 mmol). White powder; 62% yield; ^1^H NMR (400 MHz, CDCl_3_) δ 6.92 (d, *J* = 8.4 Hz, 1H), 6.53 (dd, *J* = 3.3, 1.8 Hz, 1H), 6.42 (dd, *J* = 8.4, 2.5 Hz, 1H), 6.36 (d, *J* = 2.5 Hz, 1H), 2.72–2.52 (m, 2H), 2.35–2.30 (m, 1H), 2.22–2.12 (m, 2H), 2.08 (s, 3H), 1.96–1.89 (m, 1H), 1.73–1.67 (m, 1H), 1.46–1.07 (m, 6H), 0.79 (s, 9H), 0.73 (s, 3H), 0.00 (s, 6H).^13^C NMR (101 MHz, CDCl_3_) δ 196.72, 155.57, 153.35, 144.34, 137.58, 133.19, 125.94, 119.97, 117.21, 55.67, 46.53, 44.33, 36.97, 34.86, 32.03, 29.49, 27.84, 27.17, 26.43, 25.78, 18.21, 15.99, −4.32. ^13^C DEPT135-NMR (151 MHz, CDCl_3_) δ 144.31, 125.93, 119.95, 117.19, 55.68, 44.32, 36.97, 34.84 (inverted), 32.02 (inverted), 29.47 (inverted), 27.82 (inverted), 27.17, 26.40 (inverted), 25.74, 15.97, −4.36.

*Tert-butyl(((4bS,10bR)-7,8-dimethyl-4b,5,6,10b,11,12-hexahydrochrysen-2-yl)oxy)dimethylsilane (***8***)*. Following general procedure, A, compound **8** was obtained as a side product. White powder; 24% yield; ^1^H NMR (600 MHz, CDCl_3_) δ 7.04 (d, *J* = 8.4 Hz, 1H), 7.00 (d, *J* = 8.0 Hz, 1H), 6.84 (d, *J* = 8.0 Hz, 1H), 6.47 (dd, *J* = 8.4, 2.6 Hz, 1H), 6.42 (d, *J* = 2.6 Hz, 1H), 2.83–2.70 (m, 3H), 2.69–2.61 (m, 1H), 2.52–2.38 (m, 4H), 2.09 (s, 3H), 1.98 (s, 3H), 1.50–1.42 (m, 2H), 0.79 (s, 9H), 0.00 (s, 6H). ^13^C NMR (151 MHz, CDCl_3_) δ 211.42, 138.18, 137.99, 135.29, 134.77, 133.95, 133.03, 127.32, 126.42, 122.91, 119.83, 117.37, 41.54, 39.90, 30.20, 28.28, 27.90, 25.75, 20.45, 18.22, 15.12, 1.05, −4.34. ^13^C DEPT135-NMR (151 MHz, CDCl_3_) δ 127.32, 126.42, 122.91, 119.83, 117.37, 41.54, 39.90, 30.20 (inverted), 28.28 (inverted), 27.90 (inverted), 27.84 (inverted), 25.75, 20.46, −4.34.

*2-((8S,9S,13S,14S)-3-((tert-butyldimethylsilyl)oxy)-13-methyl-7,8,9,11,12,13,14,15-octahydro-6H-cyclopenta[a]phenanthren-17-yl)-2-((trimethylsilyl)oxy)propanenitrile (***9***)*. Following general procedure, B, compound **7** was cyanosilylated. White powder; 50% yield; ^1^H NMR (400 MHz, CDCl_3_) δ 6.91 (d, *J* = 8.4 Hz, 1H), 6.42 (dd, *J* = 8.4, 2.5 Hz, 1H), 6.37 (d, *J* = 2.5 Hz, 1H), 5.74–5.70 (m, 1H), 2.68–2.60 (m, 2H), 2.17–1.97 (m, 3H), 1.84–1.67 (m, 2H), 1.58 (s, 3H), 1.51–1.00 (m, 6H), 0.84 (s, 3H), 0.79 (s, 9H), 0.08 (s, 9H), 0.00 (s, 6H). ^13^C NMR (101 MHz, CDCl_3_) δ 152.97, 152.88, 151.95, 136.27, 131.68, 126.52, 126.30, 124.39, 120.36, 120.19, 118.57, 115.74, 70.99, 68.03, 67.26, 56.23, 56.03, 46.16, 45.65, 42.61, 42.35, 35.65, 34.18, 33.59, 30.54, 29.39, 29.22, 29.11, 28.99, 28.32, 28.05, 26.16, 25.00, 24.91, 24.32, 21.31, 16.76, 16.01, 15.94, 12.75, 0.17, 0.00, −0.37, −5.78.

*(8S,9S,13S,14S)-17-acetyl-3-((tert-butyldimethylsilyl)oxy)-13-methyl-7,8,9,11,12,13,14,15,16,17-decahydro-6H-cyclopenta[a]phenanthrene-16-carbonitrile (***10***)*. Following general procedure, B, compound **10** was obtained as a side product. White powder; 20% yield; ^1^H NMR (400 MHz, CDCl_3_) δ 6.91 (d, *J* = 8.5 Hz, 1H), 6.44 (dd, *J* = 8.5, 2.4 Hz, 1H), 6.37 (d, *J* = 2.4 Hz, 1H), 3.41 (ddd, *J* = 11.6, 8.7, 2.9 Hz, 1H), 2.79 (d, *J* = 8.7 Hz, 1H), 2.65–2.60 (m, 2H), 2.21–2.08 (m, 2H), 2.04 (s, 3H), 2.00–1.94 (m, 1H), 1.65–1.53 (m, 3H), 1.37–1.21 (m, 3H), 0.79 (s, 9H), 0.42 (s, 3H), 0.00 (s, 6H). ^13^C NMR (101 MHz, CDCl_3_) δ 204.72, 153.62, 137.47, 131.92, 126.02, 123.40, 120.09, 117.42, 69.03, 54.88, 45.31, 43.34, 38.52, 38.26, 31.11, 30.40, 29.36, 27.49, 26.27, 25.74, 25.38, 18.20, 13.43, −4.35. HRESI-MS *m*/*z* calcd for [M + H]^+^ C_27_H_39_NO_2_Si: 460.264227, found: 460.265381.

*(R)-3-((8S,9S,13S,14S)-3-((tert-butyldimethylsilyl)oxy)-13-methyl-7,8,9,11,12,13,14,15-octahydro-6H-cyclopenta[a]phenanthren-17-yl)-3-hydroxybutan-2-one (***11a***)*. Following general procedure, C, *R*-isomer **11a** was obtained. White powder; 25% yield; ^1^H NMR (400 MHz, CDCl_3_) δ 6.88 (d, *J* = 8.5 Hz, 1H), 6.41 (dd, *J* = 8.5, 2.5 Hz, 1H), 6.36 (d, *J* = 2.5 Hz, 1H), 5.71 (dd, *J* = 3.1, 1.3 Hz, 1H), 4.07 (s, 1H), 2.73–2.57 (m, 2H), 2.08−2.05 (m, 1H), 2.03 (s, 3H), 1.89–1.82 (m, 1H), 1.73–1.70 (m, 2H), 1.43–1.34 (m, 2H), 1.33 (s, 3H), 1.23–1.98 (m, 5H), 0.83 (s, 3H), 0.79 (s, 9H), 0.00 (s, 6H). ^13^C NMR (101 MHz, CDCl_3_) δ 211.12, 155.63, 153.34, 137.70, 133.16, 128.82, 125.85, 119.98, 117.14, 79.55, 57.56, 47.81, 44.15, 37.06, 34.52, 31.08, 29.53, 27.68, 26.23, 25.77, 25.32, 23.32, 18.20, 17.28, −4.34.

*(S)-3-((8S,9S,13S,14S)-3-((tert-butyldimethylsilyl)oxy)-13-methyl-7,8,9,11,12,13,14,15-octahydro-6H-cyclopenta[a]phenanthren-17-yl)-3-hydroxybutan-2-one (***11b***)*. Following general procedure, C, *S*-isomer **11b** was obtained. White powder; 37% yield; ^1^H NMR (400 MHz, CDCl_3_) δ 6.90 (d, *J* = 8.6 Hz, 1H), 6.42 (dd, *J* = 8.4, 2.6 Hz, 1H), 6.36 (d, *J* = 2.4 Hz, 1H), 5.72–5.70 (m, 1H), 3.90 (s, 1H), 2.71–2.57 (m, 2H), 2.11–2.07 (m, 1H), 2.05 (s, 3H), 1.82–1.76 (m, 2H), 1.72–1.65 (m, 2H), 1.55–1.48 (m, 2H), 1.37 (s, 3H), 1.28–1.03 (m, 4H), 0.79 (s, 9H), 0.59 (s, 3H), 0.00 (s, 6H). ^13^C NMR (101 MHz, CDCl_3_) δ 210.99, 155.56, 153.32, 137.69, 133.22, 128.78, 125.86, 119.97, 117.15, 80.09, 56.73, 48.14, 43.93, 37.15, 36.20, 31.06, 29.49, 27.67, 26.46, 25.75, 24.92, 23.74, 18.20, 17.00, −4.35.

*(R,E)-6-((tert-butyldimethylsilyl)oxy)-2-((8S,9S,13S,14S)-3-((tert-butyldimethylsilyl)oxy)-13-methyl-7,8,9,11,12,13,14,15-octahydro-6H-cyclopenta[a]phenanthren-17-yl)-2-hydroxy-6-methylhept-4-en-3-one (***12a***)*. Following general procedure, D, compound **11a** (0.500 g, 0.79 mmol) was used as a starting material. White powder; 24% yield; ^1^H NMR (600 MHz, CDCl_3_) δ 6.88 (dd, *J* = 15.1, 8.4 Hz, 2H), 6.55 (d, *J* = 15.1 Hz, 1H), 6.41 (dd, *J* = 8.4, 2.4 Hz, 1H), 6.37 (d, *J* = 2.4 Hz, 1H), 5.73 (d, *J* = 1.9 Hz, 1H), 4.21 (s, 1H), 2.72–2.58 (m, 2H), 2.06–1.97 (m, 2H), 1.94–1.85 (m, 2H), 1.74–1.62 (m, 2H), 1.44–1.32 (m, 3H), 1.30 (s, 3H), 1.17, 1.16 (two s, 6H), 1.11–0.97 (m, 2H), 0.84 (s, 3H), 0.79 (s, 9H), 0.73 (s, 9H), 0.00 (s, 6H), −0.08 (s, 6H). ^13^C NMR (151 MHz, CDCl_3_) δ 203.99, 158.57, 157.14, 155.36, 139.78, 135.22, 131.33, 127.85, 122.03, 120.90, 119.15, 80.53, 75.64, 59.60, 49.57, 46.30, 39.02, 36.17, 33.07, 31.90, 31.53, 29.69, 28.18, 27.87, 27.78, 27.25, 20.23, 19.35, 0.00, −0.04, −2.33.

*(S,E)-6-((tert-butyldimethylsilyl)oxy)-2-((8S,9S,13S,14S)-3-((tert-butyldimethylsilyl)oxy)-13-methyl-7,8,9,11,12,13,14,15-octahydro-6H-cyclopenta[a]phenanthren-17-yl)-2-hydroxy-6-methylhept-4-en-3-one (***12b***)*. Following general procedure, D, compound **11b** (0.500 g, 0.79 mmol) was used as a starting material. White powder; 28% yield; ^1^H NMR (600 MHz, CDCl_3_) δ 6.90 (d, *J* = 8.5 Hz, 1H), 6.85 (d, *J* = 15.1 Hz, 1H), 6.53 (d, *J* = 15.1 Hz, 1H), 6.42 (dd, *J* = 8.5, 2.5 Hz, 1H), 6.36 (d, *J* = 2.5 Hz, 1H), 5.73 (d, *J* = 1.8 Hz, 1H), 4.12 (s, 1H), 2.71–2.57 (m, 2H), 2.11–2.00 (m, 3H), 1.80–1.66 (m, 4H), 1.55–1.50 (m, 1H), 1.34 (s, 3H), 1.30–1.19 (m, 2H), 1.17 (s, 3H), 1.14 (s, 3H), 1.08–1.06 (m, 1H), 0.79 (s, 9H), 0.73 (s, 9H), 0.53 (s, 3H), 0.00 (s, 6H), −0.07, −0.09 (two s, 6H). ^13^C NMR (151 MHz, CDCl_3_) δ 203.67, 157.94, 157.51, 155.36, 139.80, 135.41, 131.26, 127.95, 122.03, 121.46, 119.19, 81.27, 75.63, 58.90, 50.15, 46.05, 39.24, 38.44, 33.10, 31.94, 31.75, 31.59, 29.76, 28.55, 27.90, 27.83, 27.14, 20.30, 20.28, 19.30, 0.00, −0.02, −2.29.

*(R,E)-2,6-dihydroxy-2-((8S,9S,13S,14S)-3-hydroxy-13-methyl-7,8,9,11,12,13,14,15-octahydro-6H-cyclopenta[a]phenanthren-17-yl)-6-methylhept-4-en-3-one (***13a***)*. Following general procedure, F, compound **12a** (0.636 g, 1 mmol) was deprotected using TBAF (3.1 mL, 3.1 mmol). White powder; 80% yield; ^1^H NMR (600 MHz, Acetone) δ 7.81 (s, 1H), 6.97 (d, *J* = 15.4 Hz, 1H), 6.90 (d, *J* = 8.4 Hz, 1H), 6.65 (d, *J* = 15.5 Hz, 1H), 6.44 (dd, *J* = 8.4, 2.6 Hz, 1H), 6.38 (d, *J* = 2.6 Hz, 1H), 5.83 (dd, *J* = 3.3, 1.5 Hz, 1H), 4.33 (s, 1H), 3.97 (s, 1H), 2.71–2.59 (m, 2H), 2.12–2.04 (m, 2H), 1.97–1.87 (m, 3H), 1.77–1.71 (m, 2H), 1.44–1.39 (m, 1H), 1.32 (s, 3H), 1.24–1.20 (m, 1H), 1.19 (s, 3H), 1.18 (s, 3H), 1.16–1.12 (m, 2H), 0.87 (s, 3H). ^13^C NMR (151 MHz, Acetone) δ 202.03, 156.66, 156.60, 155.98, 138.35, 132.09, 129.19, 126.76, 120.04, 116.01, 113.58, 79.41, 70.84, 58.17, 48.44, 45.04, 38.28, 35.15, 31.65, 30.27, 29.50, 28.48, 27.16, 25.89, 17.65.

*(S,E)-2,6-dihydroxy-2-((8S,9S,13S,14S)-3-hydroxy-13-methyl-7,8,9,11,12,13,14,15-octahydro-6H-cyclopenta[a]phenanthren-17-yl)-6-methylhept-4-en-3-one (***13b***)*. Following general procedure, F, compound **12b** (0.636 g, 1 mmol) was deprotected using TBAF (3.1 mL, 3.1 mmol). White powder; 58% yield; ^1^H NMR (600 MHz, Acetone) δ 7.80 (s, 1H), 6.92 (dd, *J* = 15.6, 8.4 Hz, 2H), 6.69 (d, *J* = 15.6 Hz, 1H), 6.45 (dd, *J* = 8.4, 2.6 Hz, 1H), 6.39 (d, *J* = 2.6 Hz, 1H), 5.73 (dd, *J* = 3.2, 1.4 Hz, 1H), 4.26 (s, 1H, C-19-OH), 3.92 (s, 1H), 2.70–2.59 (m, 2H), 2.16–2.04 (m, 2H), 1.93–1.82 (m, 3H), 1.78–1.73 (m, 1H), 1.63–1.58 (m, 1H), 1.51–1.46 (m, 1H), 1.43–1.37 (m, 1H), 1.35 (s, 3H), 1.31–1.21 (m, 2H), 1.18 (s, 3H), 1.17 (s, 3H), 0.69 (s, 3H). ^13^C NMR (151 MHz, Acetone) δ 201.24, 157.05, 155.98, 155.48, 138.35, 132.18, 128.38, 126.76, 120.89, 116.00, 113.57, 79.99, 70.76, 57.93, 48.72, 44.96, 38.35, 36.94, 31.58, 30.26, 29.49, 28.51, 27.35, 25.71, 17.65.

*3-((((8S,9S,13S,14S)-17-((R,E)-2,6-dihydroxy-6-methyl-3-oxohept-4-en-2-yl)-13-methyl-7,8,9,11,12,13,14,15-octahydro-6H-cyclopenta[a]phenanthren-3-yl)oxy)methyl)-4-phenyl-1,2,5-oxadiazole 2-oxide (***14a***)*. Following general procedure, G, compound **13a** (0.115 g, 0.28 mmol) was alkylated with furoxan mesylate. Yellowish green powder; 53% yield; ^1^H NMR (600 MHz, CDCl_3_) δ 7.78 (d, *J* = 7.0 Hz, 2H), 7.50–7.42 (m, 3H), 7.10–7.06 (two d, *J* = 8.6, 15.4 Hz, 2H), 6.70 (dd, *J* = 8.6, 2.7 Hz, 1H), 6.64 (d, *J* = 2.7 Hz, 1H), 6.58 (d, *J* = 15.4 Hz, 1H), 5.87 (d, *J* = 1.8 Hz, 1H), 4.98 (s, 2H), 4.31 (s, 1H), 2.84–2.72 (m, 2H), 2.20–1.95 (m, 4H), 1.86–1.77 (m, 2H), 1.55–1.49 (m, 2H), 1.43 (s, 3H), 1.32 (s, 3H), 1.31 (s, 3H), 1.24–1.07 (m, 3H), 0.94 (s, 3H). ^13^C NMR (151 MHz, CDCl_3_) δ 201.54, 157.16, 155.30, 154.85, 154.84, 138.48, 134.64, 131.36, 129.48, 129.35, 128.62, 127.77, 126.42, 126.25, 118.77, 114.93, 112.25, 78.63, 71.32, 58.30, 57.39, 47.70, 44.10, 36.94, 34.28, 31.10, 29.64, 29.50, 29.46, 27.46, 26.18, 25.20, 17.25. ^13^C DEPT135-NMR (151 MHz, CDCl_3_) δ 155.31, 131.37, 129.49, 129.36, 127.77, 126.43, 118.76, 114.93, 112.25, 58.29 (inverted), 57.38, 44.10, 36.94, 34.28 (inverted), 31.10 (inverted), 29.64 (inverted), 29.51, 27.46 (inverted), 26.18 (inverted), 25.19, 17.24. HRESI-MS *m*/*z* calcd for [M + Na]^+^ C_35_H_40_N_2_O_6_: 607.277858, found: 607.279321.

*3-((((8S,9S,13S,14S)-17-((S,E)-2,6-dihydroxy-6-methyl-3-oxohept-4-en-2-yl)-13-methyl-7,8,9,11,12,13,14,15-octahydro-6H-cyclopenta[a]phenanthren-3-yl)oxy)methyl)-4-phenyl-1,2,5-oxadiazole 2-oxide (***14b***)*. Following general procedure, G, compound **13b** (0.115 g, 0.28 mmol) was alkylated with furoxan mesylate. Yellowish green powder; 50% yield; ^1^H NMR (600 MHz, CDCl_3_) δ 7.82–7.76 (m, 2H), 7.50–7.41 (m, 3H), 7.12 (d, *J* = 8.6 Hz, 1H), 7.05 (d, *J* = 15.4 Hz, 1H), 6.70 (dd, *J* = 8.6, 2.7 Hz, 1H), 6.64 (d, *J* = 2.7 Hz, 1H), 6.58 (d, *J* = 15.4 Hz, 1H), 5.86 (dd, *J* = 3.1, 1.3 Hz, 1H), 4.99 (s, 2H), 4.17 (s, 1H), 2.84–2.74 (m, 2H), 2.20–2.16 (m, 2H), 1.94–1.86 (m, 2H), 1.84–1.77 (m, 2H), 1.65–1.51 (m, 2H), 1.47 (s, 3H), 1.36–1.33 (m, 1H), 1.31 (s, 3H), 1.28 (s, 3H), 1.24–1.18 (m, 2H), 0.64 (s, 3H). ^13^C NMR (151 MHz, CDCl_3_) δ 201.30, 157.16, 155.24, 154.84, 154.73, 138.45, 134.74, 131.36, 129.35, 129.19, 127.77, 126.47, 126.25, 119.37, 114.90, 112.26, 112.24, 79.17, 71.24, 58.30, 56.74, 48.06, 43.90, 37.08, 36.19, 31.08, 29.61, 29.47, 29.34, 27.48, 26.46, 24.93, 17.34. ^13^C DEPT135-NMR (151 MHz, CDCl_3_) δ 154.74, 131.36, 129.35, 129.20, 127.77, 126.48, 119.36, 114.89, 112.25, 58.29 (inverted), 56.74, 43.89, 37.08, 36.19 (inverted), 31.08 (inverted), 29.62 (inverted), 29.34, 27.48 (inverted), 26.46 (inverted), 24.92, 17.34. HRESI-MS *m*/*z* calcd for [M + Na]^+^ C_35_H_40_N_2_O_6_: 607.277858, found: 607.279500.

*(R,E)-4-((8S,9S,13S,14S)-3-((tert-butyldimethylsilyl)oxy)-13-methyl-7,8,9,11,12,13,14,15-octahydro-6H-cyclopenta[a]phenanthren-17-yl)-4-hydroxy-1-(4-(trifluoromethyl)phenyl)pent-1-en-3-one (***15a***)*. Following general procedure, E, compound **11a** (0.455 g, 1 mmol) underwent aldol condensation using *p*-trifluoromethylbenzaldehyde (0.261 g, 1.5 mmol). White powder; 89% yield; ^1^H NMR (400 MHz, CDCl_3_) δ 7.68 (d, *J* = 15.9 Hz, 1H), 7.57–7.45 (m, 4H), 6.92 (d, *J* = 15.9 Hz, 1H), 6.86 (d, *J* = 8.4 Hz, 1H), 6.41 (dd, *J* = 8.4, 2.5 Hz, 1H), 6.36 (d, *J* = 2.5 Hz, 1H), 5.86 (d, *J* = 1.7 Hz, 1H), 4.23 (s, 1H), 2.73–2.56 (m, 2H), 2.16–2.09 (m, 1H), 2.04–1.88 (m, 2H), 1.74–1.71 (m, 2H), 1.48–1.43 (m, 1H), 1.41 (s, 3H), 1.38–0.97 (m, 5H), 0.88 (s, 3H), 0.79 (s, 9H), 0.00 (s, 6H). ^13^C NMR (101 MHz, CDCl_3_) δ 200.70, 155.16, 153.29, 142.93, 137.70, 133.15, 132.12, 129.75, 128.80, 125.99, 125.81, 122.40, 121.10, 119.94, 117.09, 78.79 (C-19-OH), 57.53, 47.85, 43.98, 37.03, 34.50, 31.24, 29.50, 27.60, 26.19, 25.72, 25.25, 18.18, 17.25, −4.38.

*(S,E)-4-((8S,9S,13S,14S)-3-((tert-butyldimethylsilyl)oxy)-13-methyl-7,8,9,11,12,13,14,15-octahydro-6H-cyclopenta[a]phenanthren-17-yl)-4-hydroxy-1-(4-(trifluoromethyl)phenyl)pent-1-en-3-one (***15b***)*. Following general procedure, E, compound **11b** (0.455 g, 1 mmol) underwent aldol condensation using *p*-trifluoromethylbenzaldehyde (0.261 g, 1.5 mmol). White powder; 77% yield; ^1^H NMR (400 MHz, CDCl_3_) δ 7.64 (d, *J* = 15.8 Hz, 1H), 7.53–7.43 (m, 4H), 6.91 (dd, *J* = 15.8, 8.4 Hz, 2H), 6.42 (dd, *J* = 8.4, 2.5 Hz, 1H), 6.37 (d, *J* = 2.5 Hz, 1H), 5.82 (d, *J* = 1.8 Hz, 1H), 4.01 (s, 1H), 2.69–2.63 (m, 2H), 2.14–2.03 (m, 3H), 1.85–1.67 (m, 2H), 1.60–1.49 (m, 1H), 1.43 (s, 3H), 1.40–1.03 (m, 5H), 0.79 (s, 9H), 0.59 (s, 3H), 0.00 (s, 6H). ^13^C NMR (101 MHz, CDCl_3_) δ 200.40, 155.57, 153.35, 142.32, 137.68, 133.17, 132.08, 129.31, 128.69, 125.96, 125.92, 125.86, 121.87, 119.97, 117.15, 79.38, 56.92, 48.16, 43.96, 37.19, 31.98, 29.76, 29.72, 29.42, 25.73, 22.74, 18.18, 17.54, 14.16, −4.38.

*(R,E)-4-((8S,9S,13S,14S)-3-((tert-butyldimethylsilyl)oxy)-13-methyl 7,8,9,11,12,13,14,15-octahydro-6H-cyclopenta[a]phenanthren-17-yl)-4-hydroxy-1-(4-(methylthio)phenyl)pent-1-en-3-one (***15c***)*. Following general procedure, E, compound **11a** (0.455 g, 1 mmol) underwent aldol condensation using 4-(methylthio)benzaldehyde (0.228 g, 1.5 mmol). Yellow powder; 47% yield; ^1^H NMR (400 MHz, CDCl_3_) δ 7.63 (d, *J* = 15.8 Hz, 1H), 7.32 (d, *J* = 8.4 Hz, 2H), 7.06 (d, *J* = 8.4 Hz, 2H), 6.86 (d, *J* = 8.4 Hz, 1H), 6.80 (d, *J* = 15.8 Hz, 1H), 6.40 (dd, *J* = 8.4, 2.5 Hz, 1H), 6.36 (d, *J* = 2.5 Hz, 1H), 5.83 (s, *J* = 1.7 Hz, 1H), 4.34 (s, 1H), 2.73–2.56 (m, 2H), 2.32 (s, 3H), 2.14–2.08 (m, 1H), 2.03–1.86 (m, 2H), 1.75–1.68 (m, 2H), 1.46–1.44 (m, 1H), 1.39 (s, 3H), 1.35–1.09 (m, 5H) 0.87 (s, 3H), 0.79 (s, 9H), 0.00 (s, 6H). ^13^C NMR (101 MHz, CDCl_3_) δ 200.85, 155.52, 153.25, 144.46, 143.16, 137.72, 133.29, 130.74, 129.24, 129.09, 125.90, 125.84, 119.92, 117.70, 117.07, 78.56, 57.44, 47.81, 43.96, 37.05, 34.38, 31.21, 29.53, 27.62, 26.22, 25.75, 25.47, 18.19, 17.28, 15.08, −4.35.

*(S,E)-4-((8S,9S,13S,14S)-3-((tert-butyldimethylsilyl)oxy)-13-methyl-7,8,9,11,12,13,14,15-octahydro-6H-cyclopenta[a]phenanthren-17-yl)-4-hydroxy-1-(4-(methylthio)phenyl)pent-1-en-3-one (***15d***)*. Following general procedure, E, compound **11b** (0.455 g, 1 mmol) underwent aldol condensation using 4-(methylthio)benzaldehyde (0.228 g, 1.5 mmol). Yellow powder; 64% yield; ^1^H NMR (600 MHz, CDCl_3_) δ 7.60 (d, *J* = 15.7 Hz, 1H), 7.30 (d, *J* = 8.4 Hz, 2H), 7.05 (d, *J* = 8.4 Hz, 2H), 6.91 (d, *J* = 8.5 Hz, 1H), 6.79 (d, *J* = 15.7 Hz, 1H), 6.42 (dd, *J* = 8.5, 2.6 Hz, 1H), 6.36 (d, *J* = 2.6 Hz, 1H), 5.80 (dd, *J* = 3.2, 1.4 Hz, 1H), 4.17 (s, 1H), 2.69–2.60 (m, 2H), 2.32 (s, 3H), 2.12–2.04 (m, 3H), 1.87–1.79 (m, 2H), 1.71–1.69 (m, 2H), 1.57–1.52 (m, 1H), 1.42 (s, 3H), 1.31–1.20 (m, 2H), 1.08 (s, 1H), 0.79 (s, 9H), 0.57 (s, 3H), 0.00 (s, 6H). ^13^C NMR (151 MHz, CDCl_3_) δ 200.66, 155.87, 153.31, 143.90, 143.03, 137.72, 133.29, 130.79, 129.00, 128.94, 125.93, 125.87, 119.95, 118.46, 117.12, 79.13, 56.92, 48.15, 43.96, 37.17, 36.28, 31.15, 29.50, 27.67, 26.47, 25.75, 25.18, 18.20, 17.51, 15.10, −4.35.

*(R,E)-4-((8S,9S,13S,14S)-3-((tert-butyldimethylsilyl)oxy)-13-methyl-7,8,9,11,12,13,14,15-octahydro-6H-cyclopenta[a]phenanthren-17-yl)-1-(4-methoxyphenyl)-4-hydroxypent-1-en-3-one (***15e***)*. Following general procedure, E, compound **11a** (0.455 g, 1 mmol) underwent aldol condensation using 4-methoxybenzaldehyde (0.204 g, 1.5 mmol). White powder; 50% yield; ^1^H NMR (600 MHz, CDCl_3_) δ 7.65 (d, *J* = 15.7 Hz, 1H), 7.38 (d, *J* = 8.8 Hz, 2H), 6.86 (d, *J* = 8.5 Hz, 1H), 6.76 (d, *J* = 8.8 Hz, 2H), 6.73 (d, *J* = 15.7 Hz, 1H), 6.40 (dd, *J* = 8.5, 2.5 Hz, 1H), 6.36 (d, *J* = 2.5 Hz, 1H), 5.82 (d, *J* = 1.8 Hz, 1H), 4.38 (s, 1H), 3.68 (s, 3H), 2.71–2.56 (m, 2H), 2.12–2.08 (m, 1H), 2.02–1.85 (m, 3H), 1.74–1.66 (m, 2H), 1.45–1.41 (m, 1H), 1.38 (s, 3H), 1.37–1.31 (m, 2H), 1.23–1.16 (m, 1H), 1.10–1.02 (m, 1H), 0.88 (s, 3H), 0.79 (s, 9H), 0.00 (s, 6H). ^13^C NMR (151 MHz, CDCl_3_) δ 200.87, 162.04, 155.65, 153.24, 144.77, 137.73, 133.32, 130.58, 129.05, 127.08, 125.84, 119.90, 117.05, 116.46, 114.49, 78.46, 57.40, 55.47, 47.80, 43.95, 37.06, 34.33, 31.18, 29.53, 27.61, 26.21, 25.73, 25.53, 18.18, 17.26, −4.37.

*(R,E)-4-((8S,9S,13S,14S)-3-((tert-butyldimethylsilyl)oxy)-13-methyl-7,8,9,11,12,13,14,15-octahydro-6H-cyclopenta[a]phenanthren-17-yl)-1-(4-fluorophenyl)-4-hydroxypent-1-en-3-one (***15f***)*. Following general procedure, E, compound **11a** (0.455 g, 1 mmol) underwent aldol condensation using 4-fluorobenzaldehyde (0.186 g, 1.5 mmol). White powder; 75% yield; ^1^H NMR (600 MHz, CDCl_3_) δ 7.64 (d, *J* = 15.8 Hz, 1H), 7.43–7.38 (m, 2H), 6.96–6.91 (m, 2H), 6.86 (d, *J* = 8.4 Hz, 1H), 6.78 (d, *J* = 15.8 Hz, 1H), 6.40 (dd, *J* = 8.4, 2.6 Hz, 1H), 6.36 (d, *J* = 2.6 Hz, 1H), 5.83 (dd, *J* = 3.2, 1.4 Hz, 1H), 4.29 (s, 1H), 2.70–2.58 (m, 2H), 2.13–2.09 (m, 1H), 2.02–1.88 (m, 3H), 1.73–1.69 (m, 2H), 1.45–1.41 (m, 1H), 1.39 (s, 3H), 1.36–1.34 (m, 1H), 1.23 –1.04 (m, 3H), 0.88 (s, 3H), 0.79 (s, 9H), −0.00 (s, 6H). ^13^C NMR (151 MHz, CDCl_3_) δ 200.80, 155.41, 153.27, 143.65, 137.70, 133.22, 130.72, 130.66, 129.37, 125.80, 119.91, 118.52, 117.07, 116.30, 116.16, 78.61, 57.48, 47.83, 43.98, 37.04, 34.41, 31.20, 29.50, 27.61, 26.19, 25.71, 25.37, 18.17, 17.25, −4.38.

*(R,E)-4-((8S,9S,13S,14S)-3-((tert-butyldimethylsilyl)oxy)-13-methyl-7,8,9,11,12,13,14,15-octahydro-6H-cyclopenta[a]phenanthren-17-yl)-4-hydroxy-1-(5-methylthiophen-2-yl)pent-1-en-3-one (***15g***)*. Following general procedure, E, compound **11a** (0.455 g, 1 mmol) underwent aldol condensation using 5-methylthiophene-2-carbaldehyde (0.189 g, 1.5 mmol). Yellow powder; 63% yield; ^1^H NMR (600 MHz, CDCl_3_) δ 7.69 (d, *J* = 15.4 Hz, 1H), 6.99 (d, *J* = 3.6 Hz, 1H), 6.86 (d, *J* = 8.5 Hz, 1H), 6.57 (dd, *J* = 3.6, 1.1 Hz, 1H,), 6.48 (d, *J* = 15.4 Hz, 1H), 6.40 (dd, *J* = 8.5, 2.5 Hz, 1H), 6.36 (d, *J* = 2.5 Hz, 1H), 5.80 (dd, *J* = 3.2, 1.4 Hz, 1H), 4.33 (s, 1H), 2.71–2.56 (m, 2H), 2.34 (s, 3H), 2.12–2.08 (m, 1H), 2.01–1.86 (m, 3H), 1.73–1.66 (m, 2H), 1.43–1.38 (m, 1H), 1.36 (s, 3H), 1.24–1.02 (m, 4H), 0.86 (s, 3H), 0.79 (s, 9H), −0.00 (s, 6H). ^13^C NMR (151 MHz, CDCl_3_) δ 200.63, 155.50, 153.24, 145.39, 137.90, 137.74, 137.66, 133.59, 133.33, 129.12, 127.03, 125.84, 119.90, 117.05, 116.41, 78.37, 57.39, 47.77, 43.97, 37.06, 34.32, 31.17, 29.54, 27.62, 26.21, 25.73, 25.53, 18.18, 17.25, 15.97, −4.37.

*(S,E)-4-((8S,9S,13S,14S)-3-((tert-butyldimethylsilyl)oxy)-13-methyl-7,8,9,11,12,13,14,15-octahydro-6H-cyclopenta[a]phenanthren-17-yl)-4-hydroxy-1-(5-methylthiophen-2-yl)pent-1-en-3-one (***15h***)*. Following general procedure, E, compound **11b** (0.455 g, 1 mmol) underwent aldol condensation using 5-methylthiophene-2-carbaldehyde (0.189 g, 1.5 mmol). Yellow powder; 60% yield; ^1^H NMR (400 MHz, CDCl_3_) δ 7.66 (d, *J* = 15.4 Hz, 1H), 6.97 (d, *J* = 3.6 Hz, 1H), 6.91 (d, *J* = 8.5 Hz, 1H), 6.56 (dd, *J* = 3.6, 1.1 Hz, 1H), 6.47 (d, *J* = 15.4 Hz, 1H), 6.42 (dd, *J* = 8.5, 2.4 Hz, 1H), 6.36 (d, *J* = 2.4 Hz, 1H), 5.83–5.75 (m, 1H), 4.19 (s, 1H), 2.70–2.58 (m, 2H), 2.33 (s, 3H), 2.13–2.00 (m, 3H), 1.87–1.78 (m, 2H), 1.75–1.67 (m, 2H), 1.57–1.50 (m, 1H), 1.39 (s, 3H), 1.30–1.03 (m, 3H), 0.79 (s, 9H), 0.57 (s, 3H), 0.00 (s, 6H). ^13^C NMR (151 MHz, CDCl_3_) δ 200.42, 155.85, 153.29, 145.26, 137.90, 137.74, 137.20, 133.52, 133.32, 128.88, 127.01, 125.87, 119.94, 117.13, 117.10, 78.96, 56.90, 48.14, 43.96, 37.16, 36.30, 31.13, 29.51, 27.67, 26.46, 25.74, 25.25, 18.19, 17.46, 15.96, −4.36.

*(R,E)-1-(5-bromothiophen-2-yl)-4-((8S,9S,13S,14S)-3-((tert-butyldimethylsilyl)oxy)-13-methyl-7,8,9,11,12,13,14,15-octahydro-6H-cyclopenta[a]phenanthren-17-yl)-4-hydroxypent-1-en-3-one (***15i***)*. Following general procedure, E, compound **11a** (0.455 g, 1 mmol) underwent aldol condensation using 5-bromothiophene-2-carbaldehyde (0.286 g, 1.5 mmol). Yellow powder; 53% yield; ^1^H NMR (600 MHz, CDCl_3_) δ 7.63 (d, *J* = 15.4 Hz, 1H), 6.92 (d, *J* = 3.9 Hz, 1H), 6.87–6.85 (m, 2H), 6.51 (d, *J* = 15.4 Hz, 1H), 6.40 (dd, *J* = 8.5, 2.6 Hz, 1H), 6.36 (d, *J* = 2.6 Hz, 1H), 5.80 (dd, *J* = 3.2, 1.3 Hz, 1H), 4.23 (s, 1H), 2.72–2.55 (m, 2H), 2.12- 2.08 (m, 1H), 2.02–1.85 (m, 3H), 1.74–1.65 (m, 2H), 1.44–1.38 (m, 1H), 1.36 (s, 3H), 1.34–1.32 (m, 1H), 1.20–1.01 (m, 3H), 0.86 (s, 3H), 0.79 (s, 9H), 0.00 (s, 6H). ^13^C NMR (151 MHz, CDCl_3_) δ 200.46, 155.24, 153.27, 141.38, 137.74, 136.24, 133.25, 132.97, 131.45, 129.50, 125.82, 119.93, 117.98, 117.15, 117.07, 78.53, 57.47, 47.80, 43.98, 37.05, 34.42, 31.21, 29.52, 27.60, 26.21, 25.74, 25.39, 18.19, 17.24, −4.36.

*(R,E)-1-(5-bromofuran-2-yl)-4-((8S,9S,13S,14S)-3-((tert-butyldimethylsilyl)oxy)-13-methyl-7,8,9,11,12,13,14,15-octahydro-6H-cyclopenta[a]phenanthren-17-yl)-4-hydroxypent-1-en-3-one (***15j***)*. Following general procedure, E, compound **11a** (0.455 g, 1 mmol) underwent aldol condensation using 5-bromofuran-2-carbaldehyde (0.261 g, 1.5 mmol). Yellow powder; 50% yield; ^1^H NMR (600 MHz, CDCl_3_) δ 7.29 (d, *J* = 15.4 Hz, 1H), 6.86 (d, *J* = 8.5 Hz, 1H), 6.64 (d, *J* = 15.4 Hz, 1H), 6.49 (d, *J* = 3.5 Hz, 1H), 6.40 (dd, *J* = 8.5, 2.5 Hz, 1H), 6.36 (d, *J* = 2.5 Hz, 1H), 6.27 (d, *J* = 3.5 Hz, 1H), 5.83 (dd, *J* = 3.1, 1.3 Hz, 1H), 4.28 (s, 1H), 2.71–2.57 (m, 2H), 2.17–2.09 (m, 1H), 2.03–1.85 (m, 3H), 1.74–1.66 (m, 2H), 1.45–1.38 (m, 1H), 1.37 (s, 3H), 1.35–1.30 (m, 1H), 1.22–0.96 (m, 3H), 0.86 (s, 3H), 0.79 (s, 9H), −0.00 (s, 6H). ^13^C NMR (151 MHz, CDCl_3_) δ 200.69, 154.96, 153.25, 153.05, 137.78, 133.32, 129.60, 129.41, 126.42, 125.81, 119.92, 119.17, 117.05, 116.74, 114.81, 78.60, 57.21, 47.79, 43.93, 37.05, 34.45, 31.18, 29.54, 27.60, 26.23, 25.73, 25.35, 18.18, 17.26, −4.37.

*(R,E)-4-hydroxy-4-((8S,9S,13S,14S)-3-hydroxy-13-methyl-7,8,9,11,12,13,14,15-octahydro-6H-cyclopenta[a]phenanthren-17-yl)-1-(4-(trifluoromethyl)phenyl)pent-1-en-3-one (***16a***)*. Following general procedure, F, compound **15a** (0.305 g, 0.5 mmol) was deprotected using TBAF (1.55 mL, 1.55 mmol). White powder; 63% yield; ^1^H NMR (600 MHz, CDCl_3_) δ 7.77 (d, *J* = 15.8 Hz, 1H), 7.64–7.53 (m, 4H), 7.01 (d, *J* = 15.8 Hz, 1H), 6.97 (d, *J* = 8.4 Hz, 1H), 6.52 (dd, *J* = 8.4, 2.5 Hz, 1H), 6.47 (d, *J* = 2.5 Hz, 1H), 5.96 (d, *J* = 1.8 Hz, 1H), 5.04 (s, 1H), 4.38 (s, 1H), 2.80–2.67 (m, 2H), 2.24–2.19 (m, 1H), 2.10–1.98 (m, 3H), 1.82–1.79 (m, 2H), 1.51 (s, 3H), 1.47–1.40 (m, 2H), 1.32–1.06 (m, 3H), 0.97 (s, 3H). ^13^C NMR (151 MHz, CDCl_3_) δ 200.73, 155.03, 153.41, 143.11, 138.11, 137.63, 132.70, 129.88, 128.83, 126.19, 125.99, 125.97, 121.01, 115.28, 112.66, 78.87, 57.46, 47.83, 43.89, 37.05, 34.48, 31.22, 29.48, 27.51, 26.26, 25.20, 17.25.

*(S,E)-4-hydroxy-4-((8S,9S,13S,14S)-3-hydroxy-13-methyl-7,8,9,11,12,13,14,15-octahydro-6H-cyclopenta[a]phenanthren-17-yl)-1-(4-(trifluoromethyl)phenyl)pent-1-en-3-one (***16b***)*. Following general procedure, F, compound **15b** (0.305 g, 0.5 mmol) was deprotected using TBAF (1.55 mL, 1.55 mmol). White powder; 25% yield; ^1^H NMR (400 MHz, CDCl_3_) δ 7.74 (d, *J* = 15.8 Hz, 1H), 7.65–7.52 (m, 4H), 7.04–6.99 (two d, *J* = 15.8, 8.4 Hz, 2H), 6.54 (dd, *J* = 8.4, 2.6 Hz, 1H), 6.48 (d, *J* = 2.6 Hz, 1H), 5.97–5.88 (m, 1H), 4.97 (s, 1H), 4.17 (s, 1H), 2.80–2.71 (m, 2H), 2.24–2.10 (m, 3H), 1.97–1.90 (m, 1H), 1.85–1.77 (m, 2H), 1.68–1.61 (m, 1H), 1.54 (s, 3H), 1.51–1.18 (m, 4H), 0.67 (s, 3H). ^13^C NMR (101 MHz, CDCl_3_) δ 200.47, 155.37, 153.43, 142.51, 138.11, 137.67, 132.75, 129.50, 128.71, 126.24, 125.97, 125.94, 121.74, 115.27, 112.68, 79.43, 56.79, 48.12, 43.82, 37.18, 36.17, 31.14, 29.45, 27.54, 26.50, 24.92, 17.53.

*(R,E)-4-hydroxy-4-((8S,9S,13S,14S)-3-hydroxy-13-methyl-7,8,9,11,12,13,14,15-octahydro-6H-cyclopenta[a]phenanthren-17-yl)-1-(4-(methylthio)phenyl)pent-1-en-3-one (***16c***)*. Following general procedure, F, compound **15c** (0.294 g, 0.5 mmol) was deprotected using TBAF (1.55 mL, 1.55 mmol). Yellow powder; 25% yield; ^1^H NMR (400 MHz, CDCl_3_) δ 7.73 (d, *J* = 15.8 Hz, 1H), 7.42 (d, *J* = 8.4 Hz, 2H), 7.17 (d, *J* = 8.4 Hz, 2H), 6.98 (d, *J* = 8.4 Hz, 1H), 6.89 (d, *J* = 15.8 Hz, 1H), 6.52 (dd, *J* = 8.4, 2.5 Hz, 1H), 6.47 (d, *J* = 2.5 Hz, 1H), 5.93 (d, *J* = 1.7 Hz, 1H), 4.74 (s, 1H), 4.47 (s, 1H), 2.80–2.67 (m, 2H), 2.43 (s, 3H), 2.23–2.16 (m, 1H), 2.10–1.96 (m, 3H), 1.82–1.79 (m, 2H), 1.49 (s, 3H), 1.46–1.42 (m, 1H), 1.34–1.11 (m, 4H), 0.97 (s, 3H). ^13^C NMR (151 MHz, CDCl_3_) δ 200.86, 155.41, 153.38, 148.92, 144.56, 143.19, 138.13, 132.83, 129.31, 129.10, 126.22, 125.90, 117.63, 115.23, 112.61, 78.59, 60.50, 57.35, 47.78, 43.86, 37.07, 34.33, 31.18, 29.51, 25.42, 21.09, 17.24, 15.08, 14.21.

*(S,E)-4-hydroxy-4-((8S,9S,13S,14S)-3-hydroxy-13-methyl-7,8,9,11,12,13,14,15-octahydro-6H-cyclopenta[a]phenanthren-17-yl)-1-(4-(methylthio)phenyl)pent-1-en-3-one (***16d***)*. Following general procedure, F, compound **15d** (0.294 g, 0.5 mmol) was deprotected using TBAF (1.55 mL, 1.55 mmol). Yellow powder; 71% yield; ^1^H NMR (600 MHz, CDCl_3_) δ 7.70 (d, *J* = 15.7 Hz, 1H), 7.39 (d, *J* = 8.3 Hz, 2H), 7.14 (d, *J* = 8.3 Hz, 2H), 7.00 (d, *J* = 8.5 Hz, 1H), 6.88 (d, *J* = 15.7 Hz, 1H), 6.54 (dd, *J* = 8.5, 2.4 Hz, 1H), 6.47 (d, *J* = 2.4 Hz, 1H), 5.91 (d, *J* = 1.6 Hz, 1H), 4.43 (s, 1H), 2.78–2.69 (m, 2H), 2.41 (s, 3H), 2.20–2.08 (m, 3H), 1.92–1.86 (m, 2H), 1.80–1.77 (m, 1H), 1.64–1.59 (m, 1H), 1.53 (s, 3H), 1.49–1.15 (m, 4H), 0.65 (s, 3H). ^13^C NMR (151 MHz, CDCl_3_) δ 200.69, 155.60, 153.67, 144.21, 143.16, 138.03, 132.59, 130.70, 129.24, 129.05, 126.20, 125.91, 118.30, 115.36, 112.79, 79.28, 56.81, 48.12, 43.82, 37.19, 36.21, 31.14, 29.49, 27.60, 26.53, 25.10, 17.52, 15.08.

*(R,E)-4-hydroxy-4-((8S,9S,13S,14S)-3-hydroxy-13-methyl-7,8,9,11,12,13,14,15-octahydro-6H-cyclopenta[a]phenanthren-17-yl)-1-(4-methoxyphenyl)pent-1-en-3-one (***16e***)*. Following general procedure, F, compound **15e** (0.286 g, 0.5 mmol) was deprotected using TBAF (1.55 mL, 1.55 mmol). White powder; 45% yield; ^1^H NMR (600 MHz, CDCl_3_) δ 7.75 (d, *J* = 15.7 Hz, 1H), 7.46 (d, *J* = 8.8 Hz, 2H), 6.97 (d, *J* = 8.5 Hz, 1H), 6.84 (d, *J* = 8.8 Hz, 2H), 6.81 (d, *J* = 15.7 Hz, 1H), 6.51 (dd, *J* = 8.8, 2.6 Hz, 1H), 6.46 (d, *J* = 2.6 Hz, 1H), 5.92 (d, *J* = 1.8 Hz, 1H), 5.34 (bs, 1H), 4.57 (s, 1H), 3.76 (s, 3H), 2.79–2.66 (m, 2H), 2.21–2.17 (m, 1H), 2.10–1.95 (m, 3H), 1.83–1.76 (m, 2H), 1.49 (s, 3H), 1.47–1.38 (m, 2H), 1.31–1.12 (m, 3H), 0.96 (s, 3H). ^13^C NMR (151 MHz, CDCl_3_) δ 200.90, 162.08, 155.48, 153.47, 145.02, 138.10, 132.77, 130.64, 129.23, 127.04, 126.20, 116.34, 115.27, 114.51, 112.66, 78.56, 57.34, 55.49, 47.78, 43.87, 37.09, 34.31, 31.18, 29.52, 27.54, 26.27, 25.47, 17.28.

*(R,E)-1-(4-fluorophenyl)-4-hydroxy-4-((8S,9S,13S,14S)-3-hydroxy-13-methyl-7,8,9,11,12,13,14,15-octahydro-6H-cyclopenta[a]phenanthren-17-yl)pent-1-en-3-one (***16f***)*. Following general procedure, F, compound **15f** (0.280 g, 0.5 mmol) was deprotected using TBAF (1.55 mL, 1.55 mmol). White powder; 58% yield; ^1^H NMR (600 MHz, CDCl_3_) δ 7.74 (d, *J* = 15.8 Hz, 1H), 7.49 (dd, *J* = 8.7, 5.4 Hz, 2H), 7.01 (t, *J* = 8.6 Hz, 2H), 6.96 (d, *J* = 8.5 Hz, 1H), 6.87 (d, *J* = 15.8 Hz, 1H), 6.51 (dd, *J* = 8.5, 2.4 Hz, 1H), 6.46 (d, *J* = 2.4 Hz, 1H), 5.93 (d, *J* = 1.7 Hz, 1H), 5.34 (s, 1H), 4.50 (s, 1H), 2.79–2.65 (m, 2H), 2.22–2.18 (m, 1H), 2.10–1.96 (m, 3H), 1.83–1.76 (m, 2H), 1.49 (s, 3H), 1.46–1.40 (m, 2H), 1.34–1.10 (m, 3H), 0.96 (s, 3H). ^13^C NMR (151 MHz, CDCl_3_) δ 200.85, 155.21, 153.47, 143.94, 138.09, 132.69, 130.80, 130.74, 129.60, 126.19, 118.39, 116.33, 116.19, 115.30, 112.69, 78.74, 57.42, 47.81, 43.89, 37.07, 34.41, 31.21, 29.50, 27.54, 26.27, 25.31, 17.29.

*(R,E)-4-hydroxy-4-((8S,9S,13S,14S)-3-hydroxy-13-methyl-7,8,9,11,12,13,14,15-octahydro-6H-cyclopenta[a]phenanthren-17-yl)-1-(5-methylthiophen-2-yl)pent-1-en-3-one (***16g***)*. Following general procedure, F, compound **15g** (0.281 g, 0.5 mmol) was deprotected using TBAF (1.55 mL, 1.55 mmol). Yellow powder; 81% yield; ^1^H NMR (600 MHz, CDCl_3_) δ 7.79 (d, *J* = 15.4 Hz, 1H), 7.09 (d, *J* = 3.6 Hz, 1H), 6.99 (d, *J* = 8.4 Hz, 1H), 6.67 (dd, *J* = 3.6, 1.0 Hz, 1H), 6.57 (d, *J* = 15.4 Hz, 1H), 6.52 (dd, *J* = 8.4, 2.6 Hz, 1H), 6.47 (d, *J* = 2.6 Hz, 1H), 5.90 (dd, *J* = 3.2, 1.4 Hz, 1H, C-16-H), 4.85 (bs, 1H), 4.48 (s, 1H), 2.82–2.70 (m, 2H), 2.44 (s, 3H), 2.21–2.16 (m, 1H), 2.10–1.96 (m, 3H), 1.83–1.77 (m, 2H), 1.54–1.49 (m, 1H), 1.46 (s, 3H), 1.44–1.39 (m, 1H), 1.33–1.12 (m, 3H), 0.95 (s, 3H). ^13^C NMR (151 MHz, CDCl_3_) δ 200.63, 155.39, 153.33, 145.49, 138.17, 137.87, 137.80, 133.69, 132.92, 129.22, 127.05, 126.24, 116.31, 115.23, 112.61, 78.42, 57.33, 47.75, 43.88, 37.08, 34.29, 31.16, 29.53, 27.53, 26.28, 25.49, 17.24, 15.98.

*(S,E)-4-hydroxy-4-((8S,9S,13S,14S)-3-hydroxy-13-methyl-7,8,9,11,12,13,14,15-octahydro-6H-cyclopenta[a]phenanthren-17-yl)-1-(5-methylthiophen-2-yl)pent-1-en-3-one (***16h***)*. Following general procedure, F, compound **15h** (0.281 g, 0.5 mmol) was deprotected using TBAF (1.55 mL, 1.55 mmol). Yellow powder; 25% yield; ^1^H NMR (600 MHz, CDCl_3_) δ 7.77 (d, *J* = 15.3 Hz, 1H), 7.08 (d, *J* = 3.6 Hz, 1H), 7.04 (d, *J* = 8.4 Hz, 1H, Ar-H), 6.67 (dd, *J* = 3.6, 1.1 Hz, 1H), 6.56 (d, *J* = 15.4 Hz, 1H), 6.54 (dd, *J* = 8.4, 2.6 Hz, 1H), 6.48 (d, *J* = 2.6 Hz, 1H), 5.89 (dd, *J* = 3.3, 1.4 Hz, 1H, C-16-H), 4.87 (bs, 1H), 4.34 (s, 1H), 2.81–2.70 (m, 2H), 2.44 (s, 3H), 2.21–2.12 (m, 3H), 1.95–1.88 (m, 2H), 1.83–1.77 (m, 1H), 1.66–1.60 (m, 1H), 1.50 (s, 3H), 1.41–1.18 (m, 3H), 0.82–0.76 (m, 1H), 0.66 (s, 3H).

*(R,E)-1-(5-bromothiophen-2-yl)-4-hydroxy-4-((8S,9S,13S,14S)-3-hydroxy-13-methyl-7,8,9,11,12,13,14,15-octahydro-6H-cyclopenta[a]phenanthren-17-yl)pent-1-en-3-one (***16i***)*. Following general procedure, F, compound **15i** (0.313 g, 0.5 mmol) was deprotected using TBAF (1.55 mL, 1.55 mmol). Yellow powder; 63% yield; ^1^H NMR (600 MHz, CDCl_3_) δ 7.74 (d, *J* = 15.4 Hz, 1H), 7.01 (d, *J* = 3.9 Hz, 1H), 6.98 (d, *J* = 8.4 Hz, 1H), 6.97 (d, *J* = 3.9 Hz, 1H), 6.60 (d, *J* = 15.4 Hz, 1H), 6.52 (dd, *J* = 8.4, 2.7 Hz, 1H), 6.47 (d, *J* = 2.7 Hz, 1H), 5.91 (dd, *J* = 3.2, 1.4 Hz, 1H), 5.07 (bs, 1H), 4.41 (s, 1H), 2.80–2.68 (m, 2H), 2.22–2.17 (m, 1H), 2.10–1.96 (m, 3H), 1.82–1.76 (m, 2H), 1.53–1.48 (m, 1H), 1.46 (s, 3H), 1.45–1.40 (m, 1H), 1.35–1.08 (m, 3H), 0.95 (s, 3H). ^13^C NMR (151 MHz, CDCl_3_) δ 200.50, 155.09, 153.40, 141.33, 138.15, 136.43, 133.10, 132.78, 131.48, 129.64, 126.20, 117.85, 117.27, 115.27, 112.65, 78.61, 57.40, 47.77, 43.88, 37.07, 34.39, 31.19, 29.51, 27.51, 26.27, 25.34, 17.24.

*(R,E)-1-(5-bromofuran-2-yl)-4-hydroxy-4-((8S,9S,13S,14S)-3-hydroxy-13-methyl-7,8,9,11,12,13,14,15-octahydro-6H-cyclopenta[a]phenanthren-17-yl)pent-1-en-3-one (***16j***)*. Following general procedure, F, compound **15j** (0.305 g, 0.5 mmol) was deprotected using TBAF (1.55 mL, 1.55 mmol). Yellow powder; 45% yield; ^1^H NMR (600 MHz, CDCl_3_) δ 7.39 (d, *J* = 15.5 Hz, 1H), 6.99 (d, *J* = 8.5 Hz, 1H), 6.74 (d, *J* = 15.5 Hz, 1H), 6.59 (d, *J* = 3.5 Hz, 1H), 6.52 (dd, *J* = 8.5, 2.7 Hz, 1H), 6.47 (d, *J* = 2.7 Hz, 1H), 6.38 (d, *J* = 3.5 Hz, 1H), 5.94 (dd, *J* = 3.3, 1.4 Hz, 1H), 4.88 (bs, 1H), 4.44 (s, 1H), 2.81–2.69 (m, 2H), 2.25–2.20 (m, 1H), 2.10–1.97 (m, 3H), 1.82–1.77 (m, 2H), 1.54–1.50 (m, 1H), 1.48 (s, 3H), 1.47–1.41 (m, 1H), 1.35–1.09 (m, 3H), 0.96 (s, 3H). ^13^C NMR (151 MHz, CDCl_3_) δ 200.71, 154.84, 153.34, 153.02, 138.20, 132.91, 129.70, 129.53, 126.50, 126.21, 119.29, 116.65, 115.25, 114.84, 112.61, 78.65, 57.14, 47.76, 43.85, 37.07, 34.42, 31.16, 29.52, 27.51, 26.29, 25.32, 17.26.

*3-((((8S,9S,13S,14S)-17-((R,E)-2-hydroxy-3-oxo-5-(4-(trifluoromethyl)phenyl)pent-4-en-2-yl)-13-methyl-7,8,9,11,12,13,14,15-octahydro-6H-cyclopenta[a]phenanthren-3-yl)oxy)methyl)-4-phenyl-1,2,5-oxadiazole 2-oxide (***17a***)*. Following general procedure, G, compound **16a** (0.139 g, 0.28 mmol) was alkylated with furoxan mesylate. Yellowish white powder; 24% yield; ^1^H NMR (600 MHz, CDCl_3_) δ 7.90–7.88 (m, 3H), 7.73–7.69 (m, 4H), 7.60–7.51 (m, 3H), 7.18 (d, *J* = 8.7 Hz, 1H), 7.13 (d, *J* = 15.8 Hz, 1H), 6.79 (dd, *J* = 8.7, 2.6 Hz, 1H), 6.74 (d, *J* = 2.6 Hz, 1H), 6.08 (d, *J* = 1.7 Hz, 1H), 5.08 (s, 2H), 4.44 (s, 1H), 2.95–2.82 (m, 2H), 2.37–2.30 (m, 1H), 2.25–2.18 (m, 1H), 2.17–2.12 (m, 1H), 1.98–1.90 (m, 2H), 1.62 (s, 3H), 1.59–1.53 (m, 2H), 1.45–1.21 (m, 4H), 1.08 (s, 3H). ^13^C NMR (151 MHz, CDCl_3_) δ 200.67, 157.16, 155.08, 154.84, 143.00, 138.42, 137.66, 134.62, 131.36, 129.76, 129.35, 128.82, 127.77, 126.40, 126.24, 125.99, 125.97, 121.04, 114.90, 112.24, 78.80, 58.28, 57.42, 47.81, 43.91, 36.94, 34.43, 31.22, 29.61, 27.44, 26.20, 25.25, 17.19. HRESI-MS *m*/*z* calcd for [M + Na]^+^ C_39_H_37_F_3_N_2_O_5_: 693.254678, found: 693.257459.

*3-((((8S,9S,13S,14S)-17-((S,E)-2-hydroxy-3-oxo-5-(4-(trifluoromethyl)phenyl)pent-4-en-2-yl)-13-methyl-7,8,9,11,12,13,14,15-octahydro-6H-cyclopenta[a]phenanthren-3-yl)oxy)methyl)-4-phenyl-1,2,5-oxadiazole 2-oxide (***17b***)*. Following general procedure, G, compound **16b** (0.139 g, 0.28 mmol) was alkylated with furoxan mesylate. Yellowish white powder; 53% yield; ^1^H NMR (600 MHz, CDCl_3_) δ 7.78 (d, *J* = 7.0 Hz, 1H), 7.75 (d, *J* = 15.8 Hz, 1H), 7.61–7.56 (m, 4H), 7.49–7.41 (m, 3H), 7.13 (d, *J* = 8.6 Hz, 1H), 7.01 (d, *J* = 15.8 Hz, 1H), 6.71 (dd, *J* = 8.6, 2.6 Hz, 1H), 6.64 (d, *J* = 2.6 Hz, 1H), 5.94 (d, *J* = 1.9 Hz, 1H), 4.98 (s, 2H), 4.11 (s, 1H), 2.85–2.72 (m, 2H), 2.25–2.15 (m, 3H), 1.97–1.91 (m, 2H), 1.85–1.81 (m, 1H), 1.69–1.61 (m, 1H), 1.54 (s, 3H, C20-H_3_), 1.43–1.15 (m, 3H), 0.81–0.77 (m, 1H), 0.68 (s, 3H). ^13^C NMR (151 MHz, CDCl_3_) δ 200.45, 157.15, 155.45, 154.85, 142.44, 138.43, 137.69, 134.66, 131.36, 129.37, 129.35, 128.71, 127.77, 126.47, 126.25, 125.98, 125.95, 121.77, 114.90, 112.28, 112.23, 79.36, 58.29, 56.79, 48.10, 43.88, 37.08, 36.17, 31.14, 29.59, 27.48, 26.46, 24.98, 17.50. HRESI-MS *m*/*z* calcd for [M + Na]^+^: C_39_H_37_F_3_N_2_O_5_: 693.254678, found: 693.257450.

*3-((((8S,9S,13S,14S)-17-((R,E)-2-hydroxy-5-(4-(methylthio)phenyl)-3-oxopent-4-en-2-yl)-13-methyl-7,8,9,11,12,13,14,15-octahydro-6H-cyclopenta[a]phenanthren-3-yl)oxy)methyl)-4-phenyl-1,2,5-oxadiazole 2-oxide (***17c***)*. Following general procedure, G, compound **16c** (0.132 g, 0.28 mmol) was alkylated with furoxan mesylate. Yellow powder; 97% yield; ^1^H NMR (600 MHz, CDCl_3_) δ 7.80–7.77 (m, 2H), 7.73 (d, *J* = 15.7 Hz, 1H), 7.48–7.41 (m, 5H), 7.17 (d, *J* = 8.5 Hz, 2H), 7.07 (d, *J* = 8.6 Hz, 1H), 6.89 (d, *J* = 15.7 Hz, 1H), 6.68 (dd, *J* = 8.6, 2.7 Hz, 1H), 6.63 (d, *J* = 2.7 Hz, 1H), 5.93 (dd, *J* = 3.2, 1.4 Hz, 1H), 4.97 (s, 2H), 4.44 (s, 1H), 2.81–2.75 (m, 2H), 2.43 (s, 3H), 2.23–2.19 (m, 1H), 2.13–1.98 (m, 3H), 1.84–1.80 (m, 2H), 1.49 (s, 3H), 1.47–1.44 (m, 1H), 1.35–1.11 (m, 3H), 0.97 (s, 3H), 0.86–0.76 (m, 1H). ^13^C NMR (151 MHz, CDCl_3_) δ 200.81, 157.15, 155.45, 154.81, 144.49, 143.19, 138.44, 134.75, 131.34, 130.73, 129.34, 129.22, 129.08, 127.77, 126.43, 126.25, 125.91, 117.65, 114.95, 114.87, 112.23, 78.55, 58.29, 57.32, 47.76, 43.88, 36.96, 34.30, 31.17, 29.64, 27.44, 26.21, 25.45, 17.19, 15.09. HRESI-MS *m*/*z* calcd for [M + Na]^+^: C_39_H_40_N_2_O_5_S: 671.255014, found: 671.257601.

*3-((((8S,9S,13S,14S)-17-((S,E)-2-hydroxy-5-(4-(methylthio)phenyl)-3-oxopent-4-en-2-yl)-13-methyl-7,8,9,11,12,13,14,15-octahydro-6H-cyclopenta[a]phenanthren-3-yl)oxy)methyl)-4-phenyl-1,2,5-oxadiazole 2-oxide (***17d***).* Following general procedure, G, compound **16d** (0.132 g, 0.28 mmol) was alkylated with furoxan mesylate. Yellow powder; 43% yield; ^1^H NMR (600 MHz, CDCl_3_) δ 7.79–7.76 (m, 2H), 7.69 (d, *J* = 15.7 Hz, 1H), 7.48–7.42 (m, 3H), 7.40 (d, *J* = 8.4 Hz, 2H), 7.15 (d, *J* = 8.4 Hz, 2H), 7.11 (d, *J* = 8.7 Hz, 1H), 6.89 (d, *J* = 15.7 Hz, 1H), 6.69 (dd, *J* = 8.7, 2.7 Hz, 1H), 6.63 (d, *J* = 2.7 Hz, 1H), 5.91 (d, *J* = 1.9 Hz, 1H), 4.97 (s, 2H), 4.27 (s, 1H), 2.82–2.74 (m, 2H), 2.42 (s, 3H), 2.22–2.13 (m, 3H), 1.96–1.90 (m, 2H), 1.84–1.81 (m, 1H), 1.65–1.62 (m, 1H), 1.52 (s, 3H), 1.37–1.18 (m, 3H), 0.81–0.77 (m, 1H), 0.66 (s, 3H). ^13^C NMR (151 MHz, CDCl_3_) δ 200.64, 157.16, 155.80, 154.85, 143.96, 143.08, 138.45, 134.76, 131.36, 130.76, 129.35, 129.00, 128.93, 127.77, 126.49, 126.26, 125.92, 118.41, 114.90, 112.27, 112.23, 79.12, 58.31, 56.81, 48.09, 43.90, 37.09, 36.21, 31.13, 29.62, 27.51, 26.49, 25.19, 17.46, 15.10. HRESI-MS *m*/*z* calcd for [M + Na]^+^: C_39_H_40_N_2_O_5_S: 671.255014, found: 671.257434.

*3-((((8S,9S,13S,14S)-17-((R,E)-2-hydroxy-5-(4-methoxyphenyl)-3-oxopent-4-en-2-yl)-13-methyl-7,8,9,11,12,13,14,15-octahydro-6H-cyclopenta[a]phenanthren-3-yl)oxy)methyl)-4-phenyl-1,2,5-oxadiazole 2-oxide (***17e***)*. Following general procedure, G, compound **16e** (0.128 g, 0.28 mmol) was alkylated with furoxan mesylate. Yellow powder; 59% yield; ^1^H NMR (600 MHz, CDCl_3_) δ 7.79–7.73 (m, 3H), 7.47 (d, *J* = 8.4 Hz, 2H), 7.45–7.41 (m, 3H), 7.06 (d, *J* = 8.6 Hz, 1H), 6.85 (d, *J* = 8.4 Hz, 2H), 6.82 (d, *J* = 15.6 Hz, 2H), 6.67 (dd, *J* = 8.6, 2.6 Hz, 1H), 6.62 (d, *J* = 2.6 Hz, 1H), 5.92 (d, *J* = 1.8 Hz, 1H), 4.96 (s, 2H), 4.48 (s, 1H), 3.76 (s, 3H), 2.83–2.70 (m, 2H), 2.22–2.18 (m, 1H), 2.11–1.96 (m, 3H), 1.84–1.80 (m, 2H), 1.54–1.50 (m, 1H), 1.48 (s, 3H), 1.46–1.42 (m, 1H), 1.37–1.13 (m, 2H), 0.96 (s, 3H), 0.82–0.77 (m, 1H). ^13^C NMR (151 MHz, CDCl_3_) δ 200.82, 162.07, 157.16, 155.58, 154.81, 144.84, 138.45, 134.79, 131.35, 130.60, 129.35, 129.05, 127.77, 127.05, 126.44, 126.25, 116.39, 114.86, 114.50, 112.22, 78.46, 58.29, 57.29, 55.48, 47.75, 43.88, 36.97, 34.26, 31.17, 29.65, 27.45, 26.22, 25.54, 17.21. HRESI-MS *m*/*z* calcd for [M + Na]^+^: C_39_H_40_N_2_O_6_: 655.277858, found: 655.279825.

*3-((((8S,9S,13S,14S)-17-((R,E)-5-(4-fluorophenyl)-2-hydroxy-3-oxopent-4-en-2-yl)-13-methyl-7,8,9,11,12,13,14,15-octahydro-6H-cyclopenta[a]phenanthren-3-yl)oxy)methyl)-4-phenyl-1,2,5-oxadiazole 2-oxide (***17f***)*. Following general procedure, G, compound **16f** (0.124 g, 0.28 mmol) was alkylated with furoxan mesylate. Yellow powder; 22% yield; ^1^H NMR (600 MHz, CDCl_3_) δ 7.79–7.76 (m, 2H), 7.74 (d, *J* = 15.8 Hz, 1H), 7.52–7.48 (m, 2H), 7.46–7.41 (m, 3H), 7.06 (d, *J* = 8.6 Hz, 1H), 7.02 (t, *J* = 8.6 Hz, 2H), 6.87 (d, *J* = 15.8 Hz, 1H), 6.67 (dd, *J* = 8.6, 2.7 Hz, 1H), 6.62 (d, *J* = 2.7 Hz, 1H), 6.02–5.87 (m, 1H, C-16-*H*), 4.96 (s, 2H), 4.39 (s, 1H), 2.82–2.71 (m, 2H), 2.23–1.19 (m, 1H), 2.11–1.98 (m, 3H), 1.84–1.80 (m, 2H), 1.55–1.49 (m, 1H), 1.46–1.08 (m, 3H), 0.97 (s, 3H), 0.81–0.78 (m, 1H). ^13^C NMR (151 MHz, CDCl_3_) δ 200.74, 165.17, 163.50, 157.14, 155.37, 154.83, 143.72, 138.42, 134.68, 131.34, 130.74, 130.68, 129.34, 127.77, 126.41, 126.27, 118.45, 116.32, 116.17, 114.89, 112.24, 78.61, 58.29, 57.37, 47.79, 43.92, 36.96, 34.35, 31.96, 29.74, 27.46, 22.72, 17.19, 14.15. HRESI-MS *m*/*z* calcd for [M + Na]^+^: C_38_H_37_FN_2_O_5_: 643.257871, found: 643.259297.

*3-((((8S,9S,13S,14S)-17-((R,E)-2-hydroxy-5-(5-methylthiophen-2-yl)-3-oxopent-4-en-2-yl)-13-methyl-7,8,9,11,12,13,14,15-octahydro-6H-cyclopenta[a]phenanthren-3-yl)oxy)methyl)-4-phenyl-1,2,5-oxadiazole 2-oxide (***17g***)*. Following general procedure, G, compound **16g** (0.120 g, 0.28 mmol) was alkylated with furoxan mesylate. Yellow powder; 39% yield; ^1^H NMR (600 MHz, CDCl_3_) δ 7.82–7.76 (m, 3H), 7.49–7.42 (m, 4H), 7.10 (d, *J* = 3.6 Hz, 1H), 7.07 (d, *J* = 8.8 Hz, 1H), 6.69–6.65 (m, 2H), 6.63 (d, *J* = 2.7 Hz, 1H), 6.57 (d, *J* = 15.4 Hz, 1H), 5.90 (dd, *J* = 3.6, 1.3 Hz, 1H), 4.97 (s, 2H), 4.43 (s, 1H), 2.84–2.75 (m, 2H), 2.44 (s, 3H), 2.22–2.18 (m, 1H), 2.13–2.03 (m, 2H), 2.02–1.95 (m, 1H), 1.84–1.77 (m, 2H), 1.54–1.49 (m, 1H), 1.46 (s, 3H), 1.37–1.10 (m, 3H), 0.96 (s, 3H), 0.87–0.74 (m, 1H). ^13^C NMR (151 MHz, CDCl_3_) δ 200.58, 157.16, 155.43, 154.80, 145.45, 138.47, 137.88, 137.72, 134.80, 133.64, 131.35, 129.35, 129.11, 127.77, 127.05, 126.44, 126.26, 116.34, 114.86, 112.22, 78.37, 58.29, 57.28, 47.72, 43.90, 36.97, 34.24, 31.15, 29.66, 27.45, 26.22, 25.53, 17.19, 15.97. HRESI-MS *m*/*z* calcd for [M + Na]^+^: C_37_H_38_N_2_O_5_S: 645.239364, found: 645.241095.

*3-((((8S,9S,13S,14S)-17-((S,E)-2-hydroxy-5-(5-methylthiophen-2-yl)-3-oxopent-4-en-2-yl)-13-methyl-7,8,9,11,12,13,14,15-octahydro-6H-cyclopenta[a]phenanthren-3-yl)oxy)methyl)-4-phenyl-1,2,5-oxadiazole 2-oxide (***17h***)*. Following general procedure, G, compound **16h** (0.120 g, 0.28 mmol) was alkylated with furoxan mesylate. Yellow powder; 33% yield; ^1^H NMR (600 MHz, CDCl_3_) δ 7.80–7.78 (m, 2H), 7.76 (d, *J* = 15.3 Hz, 1H), 7.54–7.40 (m, 3H), 7.13 (d, *J* = 8.6 Hz, 1H), 7.08 (d, *J* = 3.6 Hz, 1H), 6.70 (dd, *J* = 8.6, 2.8 Hz, 1H), 6.67 (dd, *J* = 3.6, 1.1 Hz, 1H), 6.64 (d, *J* = 2.8 Hz, 1H), 6.57 (d, *J* = 15.3 Hz, 1H), 5.89 (dd, *J* = 3.3, 1.4 Hz, 1H), 4.98 (s, 2H), 4.29 (s, 1H), 2.83–2.75 (m, 2H), 2.44 (s, 3H), 2.26–2.14 (m, 3H), 2.00–1.90 (m, 2H), 1.85–1.81 (m, 1H), 1.67–1.62 (m, 1H), 1.50 (s, 3H), 1.42–1.18 (m, 3H), 0.83–0.79 (m, 1H), 0.67 (s, 3H). ^13^C NMR (151 MHz, CDCl_3_) δ 207.11, 154.75, 145.27, 138.46, 137.88, 134.80, 133.54, 131.34, 129.34, 128.85, 127.77, 127.01, 126.49, 126.26, 118.66, 117.08, 114.88, 114.88, 112.26, 112.22, 78.81, 66.58, 58.30, 56.78, 48.08, 43.91, 37.08, 36.23, 31.10, 29.62, 27.50, 26.48, 17.40, 15.95. HRESI-MS *m*/*z* calcd for [M + Na]^+^ C_37_H_38_N_2_O_5_S: 645.239364, found: 645.241750.

*3-((((8S,9S,13S,14S)-17-((R,E)-5-(5-bromothiophen-2-yl)-2-hydroxy-3-oxopent-4-en-2-yl)-13-methyl-7,8,9,11,12,13,14,15-octahydro-6H-cyclopenta[a]phenanthren-3-yl)oxy)methyl)-4-phenyl-1,2,5-oxadiazole 2-oxide (***17i***)*. Following general procedure, G, compound **16i** (0.144 g, 0.28 mmol) was alkylated with furoxan mesylate. Yellowish brown powder; 48% yield; ^1^H NMR (600 MHz, CDCl_3_) δ 7.79–7.77 (m, 2H), 7.74 (d, *J* = 15.4 Hz, 1H), 7.48–7.42 (m, 3H), 7.07 (d, *J* = 8.6 Hz, 1H), 7.02 (d, *J* = 3.9 Hz, 1H), 6.97 (d, *J* = 3.9 Hz, 1H), 6.68 (dd, *J* = 8.6, 2.7 Hz, 1H), 6.63 (d, *J* = 2.7 Hz, 1H), 6.60 (d, *J* = 15.4 Hz, 1H), 5.91 (dd, *J* = 3.1, 1.3 Hz, 1H), 4.97 (s, 2H), 4.34 (s, 1H), 2.83–2.75 (m, 2H), 2.21–2.19 (m, 1H), 2.12–1.97 (m, 3H), 1.85–1.77 (m, 2H), 1.54–1.51 (m, 1H), 1.46 (s, 3H), 1.38–1.08 (m, 3H), 0.95 (s, 3H), 0.81–0.79 (m, 1H). ^13^C NMR (151 MHz, CDCl_3_) δ 200.43, 157.16, 155.16, 154.82, 141.36, 138.47, 136.30, 134.72, 133.02, 131.46, 131.35, 129.49, 129.35, 127.77, 126.42, 126.25, 117.90, 117.20, 114.89, 112.22, 78.52, 58.29, 57.35, 47.75, 43.90, 36.96, 34.34, 31.18, 29.64, 27.43, 26.22, 25.38, 17.17. HRESI-MS *m*/*z* calcd for [M + Na]^+^ C_36_H_35_BrN_2_O_5_S: 709.134226, found: 709.136204.

*3-((((8S,9S,13S,14S)-17-((R,E)-5-(5-bromofuran-2-yl)-2-hydroxy-3-oxopent-4-en-2-yl)-13-methyl-7,8,9,11,12,13,14,15-octahydro-6H-cyclopenta[a]phenanthren-3-yl)oxy)methyl)-4-phenyl-1,2,5-oxadiazole 2-oxide (***17j***)*. Following general procedure, G, compound **16j** (0.139 g, 0.28 mmol) was alkylated with furoxan mesylate. Yellowish brown powder; 50% yield; ^1^H NMR (600 MHz, CDCl_3_) δ 7.80–7.76 (m, 2H), 7.48–7.42 (m, 3H), 7.39 (d, *J* = 15.4 Hz, 1H), 7.08 (d, *J* = 8.7 Hz, 1H), 6.74 (d, *J* = 15.4 Hz, 1H), 6.68 (dd, *J* = 8.7, 2.7 Hz, 1H), 6.63 (d, *J* = 2.7 Hz, 1H), 6.60 (d, *J* = 3.5 Hz, 1H), 6.38 (d, *J* = 3.5 Hz, 1H), 5.94 (dd, *J* = 3.2, 1.3 Hz, 1H), 4.98 (s, 2H), 4.38 (s, 1H), 2.84–2.73 (m, 2H), 2.25–2.20 (m, 1H), 2.13–2.06 (m, 2H), 2.02–1.97 (m, 1H), 1.86–1.77 (m, 2H), 1.52–1.49 (m, 1H), 1.48 (s, 3H), 1.37–1.09 (m, 3H), 0.96 (s, 3H), 0.82–0.78 (m, 1H). ^13^C NMR (151 MHz, CDCl_3_) δ 200.66, 157.16, 154.88, 154.81, 153.03, 138.50, 134.79, 131.35, 129.60, 129.46, 129.35, 127.77, 126.46, 126.42, 126.26, 119.23, 116.68, 114.89, 114.83, 112.23, 112.21, 78.59, 58.29, 57.10, 47.73, 43.87, 36.97, 34.38, 31.16, 29.66, 27.43, 26.24, 25.36, 17.20. HRESI-MS *m*/*z* calcd for [M + Na]^+^ C_36_H_35_BrN_2_O_6_: 693.157070, found: 693.159715.

*(S)-3-hydroxy-3-((8S,9S,13S,14S)-3-hydroxy-13-methyl-7,8,9,11,12,13,14,15-octahydro-6H-cyclopenta[a]phenanthren-17-yl)butan-2-one (***18***).* Following general procedure, F, compound **11b** (0.227 g, 0.5 mmol) was deprotected using TBAF (1.55 mL, 1.55 mmol). White powder; 82% yield; ^1^H NMR (600 MHz, CDCl_3_) δ 6.99 (d, *J* = 8.4 Hz, 1H), 6.54 (dd, *J* = 8.4, 2.7 Hz, 1H), 6.48 (d, *J* = 2.7 Hz, 1H), 6.10 (s, 1H), 5.83 (dd, *J* = 3.3, 1.5 Hz, 1H), 4.22 (s, 1H), 2.78–2.67 (m, 2H), 2.18–2.11 (m, 5H), 2.10–2.05 (m, 1H), 1.89–1.86 (m, 1H), 1.80–1.71 (m, 2H), 1.61–1.56 (m, 1H), 1.49 (s, 3H), 1.47–1.17 (m, 3H), 0.82–0.75 (m, 1H), 0.66 (s, 3H). ^13^C NMR (151 MHz, CDCl_3_) δ 211.26, 155.17, 153.67, 138.00, 132.51, 129.24, 126.17, 115.40, 112.83, 80.33, 56.63, 48.12, 43.76, 37.16, 36.14, 31.07, 29.49, 27.58, 26.51, 24.78, 23.76, 17.01.

*3-((((8S,9S,13S,14S)-17-((S)-2-hydroxy-3-oxobutan-2-yl)-13-methyl-7,8,9,11,12,13,14,15-octahydro-6H-cyclopenta[a]phenanthren-3-yl)oxy)methyl)-4-phenyl-1,2,5-oxadiazole 2-oxide (***19***)*. Following general procedure, G, compound **18** (0.095 g, 0.28 mmol) was alkylated with furoxan mesylate. White powder; 50% yield; ^1^H NMR (600 MHz, CDCl_3_) δ 7.75–7.72 (m, 2H), 7.42–7.37 (m, 3H), 7.06 (d, *J* = 8.7 Hz, 1H), 6.65 (dd, *J* = 8.7, 2.6 Hz, 1H), 6.60 (d, *J* = 2.6 Hz, 1H), 5.78 (d, *J* = 1.8 Hz, 1H), 4.91 (s, 2H), 3.98 (s, 1H), 2.79–2.70 (m, 2H), 2.16–2.08 (m, 6H), 1.89–1.85 (m, 2H), 1.81–1.77 (m, 1H), 1.75–1.69 (m, 1H), 1.60–1.55 (m, 1H), 1.43 (s, 3H), 1.36–1.12 (m, 2H), 0.78 - 0.71 (m, 1H), 0.65 (s, 3H). ^13^C NMR (151 MHz, CDCl_3_) δ 210.96, 157.13, 155.52, 154.91, 138.40, 134.65, 131.37, 129.36, 128.74, 127.76, 126.45, 126.28, 114.93, 112.27, 112.21, 80.12, 58.32, 56.67, 48.10, 43.90, 37.08, 36.17, 31.06, 29.61, 27.53, 26.50, 24.97, 23.74, 16.96.

*3-((((8S,9S,13S,14S)-17-((S,E)-2-hydroxy-5-(4-methoxyphenyl)-3-oxopent-4-en-2-yl)-13-methyl-7,8,9,11,12,13,14,15-octahydro-6H-cyclopenta[a]phenanthren-3-yl)oxy)methyl)-4-phenyl-1,2,5-oxadiazole 2-oxide (***20a***)*. Following general procedure, E, compound **11b** (0.455 g, 1 mmol) underwent aldol condensation using 4-methoxybenzaldehyde (0.204 g, 1.5 mmol). Yellowish brown powder; 32% yield; ^1^H NMR (600 MHz, CDCl_3_) δ 7.80–7.75 (m, 2H), 7.72 (d, *J* = 15.7 Hz, 1H), 7.48–7.42 (m, 5H), 7.12 (d, *J* = 8.7 Hz, 1H), 6.84 (d, *J* = 8.8 Hz, 2H), 6.81 (d, *J* = 15.7 Hz, 1H), 6.70 (dd, *J* = 8.7, 2.7 Hz, 1H), 6.63 (d, *J* = 2.7 Hz, 1H), 5.91 (dd, *J* = 3.1, 1.2 Hz, 1H), 4.97 (s, 2H), 4.33 (s, 1H), 3.77 (s, 3H), 2.83–2.74 (m, 2H), 2.22–2.16 (m, 3H), 1.94–1.91 (m, 1H), 1.85–1.79 (m, 2H), 1.67–1.62 (m, 1H), 1.52 (s, 3H), 1.48–1.45 (m, 1H), 1.36–1.17 (m, 2H), 0.81–0.79 (m, 1H), 0.67 (s, 3H). ^13^C NMR (151 MHz, CDCl_3_) δ 200.66, 162.01, 157.16, 155.91, 154.84, 144.31, 138.45, 134.79, 131.35, 130.49, 129.35, 128.78, 127.77, 127.06, 126.49, 126.26, 117.15, 114.88, 114.48, 112.26, 112.23, 79.01, 58.30, 56.81, 55.47, 48.09, 43.90, 37.09, 36.22, 31.11, 29.62, 27.51, 26.49, 25.26, 17.44. HRESI-MS *m*/*z* calcd for [M + Na]^+^: C_39_H_40_N_2_O_6_: 655.277858, found: 655.279543.

*3-((((8S,9S,13S,14S)-17-((S,E)-5-(4-fluorophenyl)-2-hydroxy-3-oxopent-4-en-2-yl)-13-methyl-7,8,9,11,12,13,14,15-octahydro-6H-cyclopenta[a]phenanthren-3-yl)oxy)methyl)-4-phenyl-1,2,5-oxadiazole 2-oxide (***20b***)*. Following general procedure, E, compound **11b** (0.455 g, 1 mmol) underwent aldol condensation using 4-fluorobenzaldehyde (0.186 g, 1.5 mmol). White powder; 38% yield; ^1^H NMR (600 MHz, CDCl_3_) δ 7.78–7.77 (m, 2H), 7.71 (d, *J* = 15.8 Hz, 1H), 7.51–7.40 (m, 5H), 7.12 (d, *J* = 8.7 Hz, 1H), 7.02 (t, *J* = 8.6 Hz, 2H), 6.87 (d, *J* = 15.8 Hz, 1H), 6.70 (dd, *J* = 8.7, 2.7 Hz, 1H), 6.64 (d, *J* = 2.7 Hz, 1H), 5.97–5.84 (m, 1H, C-16-H), 4.98 (s, 2H), 4.22 (s, 1H), 2.83–2.74 (m, 2H), 2.23–2.14 (m, 3H), 1.94–1.91 (m, 1H), 1.85–1.79 (m, 2H), 1.68–1.64 (m, 1H), 1.52 (s, 3H), 1.49–0.77 (m, 4H), 0.67 (s, 3H). ^13^C NMR (151 MHz, CDCl_3_) δ 200.58, 165.13, 163.45, 157.15, 155.69, 154.85, 143.17, 138.44, 134.72, 131.35, 130.63, 130.57, 129.35, 129.06, 127.77, 126.48, 126.26, 119.23, 116.31, 116.16, 114.89, 112.27, 79.18, 58.30, 56.80, 48.10, 43.90, 37.09, 36.20, 31.12, 29.61, 27.50, 26.48, 25.12, 17.46. HRESI-MS *m*/*z* calcd for [M + Na]^+^: C_38_H_37_FN_2_O_5_: 643.257871, found: 643.259571.

*3-((((8S,9S,13S,14S)-17-((S,E)-5-(5-bromothiophen-2-yl)-2-hydroxy-3-oxopent-4-en-2-yl)-13-methyl-7,8,9,11,12,13,14,15-octahydro-6H-cyclopenta[a]phenanthren-3-yl)oxy)methyl)-4-phenyl-1,2,5-oxadiazole 2-oxide (***20c***)*. Following general procedure, E, compound **11b** (0.455 g, 1 mmol) underwent aldol condensation using 5-bromothiophene-2-carbaldehyde (0.286 g, 1.5 mmol). Yellowish brown powder; 26% yield; ^1^H NMR (600 MHz, CDCl_3_) δ 7.80–7.77 (m, 2H), 7.71 (d, *J* = 15.4 Hz, 1H), 7.49–7.42 (m, 3H), 7.12 (d, *J* = 8.7 Hz, 1H), 7.01 (d, *J* = 3.9 Hz, 1H), 6.97 (d, *J* = 3.9 Hz, 1H), 6.70 (dd, *J* = 8.7, 2.5 Hz, 1H), 6.64 (d, *J* = 2.5 Hz, 1H), 6.60 (d, *J* = 15.4 Hz, 1H), 5.89 (d, *J* = 1.9 Hz, 1H), 4.98 (s, 2H), 4.17 (s, 1H), 2.82–2.76 (m, 2H), 2.23–2.15 (m, 3H), 1.94–1.90 (m, 1H), 1.83–1.80 (m, 2H), 1.66–1.61 (m, 1H), 1.49 (s, 3H), 1.40–1.16 (m, 3H), 0.84–0.77 (m, 1H), 0.66 (s, 3H). ^13^C NMR (151 MHz, CDCl_3_) δ 200.21, 157.15, 155.53, 154.84, 141.37, 138.45, 135.81, 134.73, 132.90, 131.44, 131.35, 129.35, 129.19, 127.77, 126.47, 126.26, 118.65, 117.05, 114.90, 112.26, 112.22, 79.11, 58.30, 56.77, 48.08, 43.89, 37.08, 36.20, 31.12, 29.61, 27.49, 26.47, 25.12, 17.44. HRESI-MS *m*/*z* calcd for [M + Na]^+^ C_36_H_35_BrN_2_O_5_S: 709.134226, found: 709.136110.

*3-((((8S,9S,13S,14S)-17-((S,E)-5-(5-bromofuran-2-yl)-2-hydroxy-3-oxopent-4-en-2-yl)-13-methyl-7,8,9,11,12,13,14,15-octahydro-6H-cyclopenta[a]phenanthren-3-yl)oxy)methyl)-4-phenyl-1,2,5-oxadiazole 2-oxide (***20d***)*. Following general procedure, E, compound **11b** (0.455 g, 1 mmol) underwent aldol condensation using 5-bromofuran-2-carbaldehyde (0.261 g, 1.5 mmol). Yellowish brown powder; 17% yield; ^1^H NMR (600 MHz, CDCl_3_) δ 7.80–7.77 (m, 2H), 7.49–7.42 (m, 3H), 7.36 (d, *J* = 15.4 Hz, 1H), 7.13 (d, *J* = 8.7 Hz, 1H), 6.76 (d, *J* = 15.4 Hz, 1H), 6.70 (dd, *J* = 8.7, 2.7 Hz, 1H), 6.64 (d, *J* = 2.7 Hz, 1H), 6.58 (d, *J* = 3.5 Hz, 1H), 6.37 (d, *J* = 3.5 Hz, 1H), 5.92 (dd, *J* = 3.2, 1.4 Hz, 1H), 4.99 (s, 2H), 4.22 (s, 1H), 2.82–2.74 (m, 2H), 2.23–2.14 (m, 3H), 1.98–1.90 (m, 2H), 1.84–1.81 (m, 1H), 1.67–1.62 (m, 1H), 1.52 (s, 3H), 1.49–1.14 (m, 3H), 0.82–0.76 (m, 1H), 0.66 (s, 3H, C-18-*H_3_*). ^13^C NMR (151 MHz, CDCl_3_) δ 200.56, 157.16, 155.37, 154.84, 153.02, 138.48, 134.78, 131.35, 129.35, 129.24, 128.97, 127.77, 126.48, 126.38, 126.26, 119.07, 117.41, 114.89, 114.77, 112.25, 112.22, 79.15, 58.30, 56.74, 48.08, 43.89, 37.09, 36.25, 31.14, 29.72, 27.50, 26.49, 25.06, 17.44. HRESI-MS *m*/*z* calcd for [M + Na]^+^ C_36_H_35_BrN_2_O_6_: 693.157070, found: 693.159263.

### 3.3. Biological Evaluation

#### 3.3.1. In Vitro Cytotoxicity Assay

The in vitro antiproliferation activity of the target NO-CIETA was measured by the MTT assay, as reported [48,49,50]. Briefly, HepG2 cells were seeded in 96-well plates (2 × 10^4^ cells/well) and incubated for 24 h in a 37 °C humidified incubator (5% CO_2_). After that, cells were incubated for 48 h with the reference drug erlotinib, the absence (negative control), and the presence of various concentrations of tested compounds, respectively. Each group was arranged in four parallel wells. MTT solution (5 mg/mL) was added (20 µL/well) and cells were incubated for 2 h at 37 °C. Finally, the MTT-containing medium was replaced with DMSO (200 μL/well) and mixed well. The absorbance was measured by a microplate reader (Hidex Sense) at a 570 nm wavelength. Survival percentage was calculated using the following Equation (1):

(1) $$Inhibitory\;rate\%=\frac{\left({\mathrm{Abs}}_{570\;control\;cells}–{Abs}_{570\;treated\;cells}\right)}{{Abs}_{570\;control\;cells}}\times 100$$

IC_50_ values were obtained from linear regression analysis of the concentration–response curves plotted for each tested compound.

#### 3.3.2. Cell-Based ELISA Assay

In-cell Western protocol on an Odyssey^®^ imager (LI-COR^®^) was followed according to manufacturer’s directions [51,52,53,54]. Briefly, in clear-bottomed and black-walled 96-well plates, HepG2 cells were seeded (0.5 × 10^6^ cells/mL) and allowed to grow to confluence. Then, cells were incubated for 24 h with different concentrations of compound **21a** (0.6, 1.2, and 2.4 µM) and DMSO as control. Cells were then fixed, permeabilized, blocked, and incubated for 2 h with an antibody with gentle shaking and then incubated overnight at 4 °C. Cells were then washed and incubated with secondary antibodies conjugated to IRdye for 1 h. Finally, cells were washed 4 times, and the plate was blotted dry. Phospho-proteins were normalized for total protein signals while p-MEK1/2 and MRP2 were normalized for the β-actin and GAPDH loading controls, respectively, to correct for well-to-well variation in cell number and %inhibition determined relative to control wells. Data are expressed as mean values of at least two runs ± SD.

#### 3.3.3. Cell Cycle Analysis

The HepG2 cells were seeded in a 6-well plate (5 × 10^5^ cells/well) and allowed to adhere overnight at 37 °C and 5% CO_2_. The cells were then incubated for 24 h with NO-CIETA **21a** (2.4 μM) and **21b** (8.1 μM), and DMSO (0.001%) as a control. The cells were then washed twice with ice-cold 1X PBS and collected after trypsinization [55]. Then, the cell pellet was washed twice with ice-cold 1X PBS and fixed overnight with ice-cold 70% ethanol at −20 °C. After that, the cells were washed once with ice-cold PBS and the second wash with ice-cold PBS-2% FBS. The cell pellet was re-suspended in 500 μL propidium iodide (PI)/RNase staining solution (BD Biosciences) and 0.1% triton x-100 for 30 min at room temperature in the dark and analyzed within 1 h by BD Accuri C6 flow cytometer (Becton-Dickinson, Mountain View, CA, USA) [56]. Data were analyzed by MFLT32 software and 10,000 events with slow flow rates were recorded for each sample.

#### 3.3.4. DAF-FM DA Assay for Intracellular Measurement of NO

The NO-sensitive fluorophore DAF-FM DA was used for quantification of NO intracellular levels. Briefly, HepG2 cells were seeded in a six-well plate (7.5 × 10^5^ cells/well) and allowed to grow overnight. Cells were incubated for 30 min with 2 mL per well of 2.5 μM DAF-FM DA at 37 °C and 5% CO_2_, rinsed twice, and then loaded with different concentrations of target compounds. After incubation time (1, 6, 12, and 24 h), cells were washed twice with HPSS, trypsinized, and pipetted into flow cytometer test tubes. The fluorescence formed intracellularly was analyzed by BD Acuuri C6 flow cytometer (Becton-Dickinson, Mountain View, CA, USA) with the excitation source at 495 nm and emission at 515 nm. Data are expressed as mean values of at least two runs ± SD [33,49,57,58].

### 3.4. Computational Studies

OpenEye^®^ software (version 2022.2.1) was used for molecular Modeling studies. This software package generates chemguas4 scoring, which is a filtering process to obtain virtual binding affinity, the lower score, the better the binding affinity of the ligands toward the receptor. Briefly, a virtual library of NO-∆-16-CIEAs was energy minimized using MMFF94 force field, followed by generation of multi-conformers using OMEGA^®^ application. The whole library was then subjected to shape and electrostatic similarity with the lead compound NO-CIEA **I** using EON application. The designed library was then docked along with the prepared EGFR (PDB:ID 1M17), ERK2 (PDB:ID 5NHJ), MEK1 (PDB:ID 1S9J), and MRP1 (PDB:ID 2CBZ) using FRED^®^ application. Finally, Vida application was used to show the potential binding interactions of the ligands to the receptor of interest [37,38,39,59,60,61,62].

## 4. Conclusions

A new series of NO-∆-16-CIEAs was synthesized and evaluated for their *anti*-proliferative activity against hepatocellular carcinoma. Among them, **14a**,**b** showed broad-spectrum potent *anti*-proliferative activity against both HepG2 and HepG2-R cell lines in comparison to erlotinib. Moreover, compound **17a** showed remarkably high inhibitory activity against the HepG2 cell line comparable to erlotinib, while **17b** did not show any activity at a concentration as high as 50 µM. Furthermore, compounds **17g** and **17h** were almost equipotent to Erlotinib. Compound **14a** showed a significant increase in fluorescence after 6 h of incubation in a dose-dependent manner and produced amounts of NO in tumor cells comparable to the reference prodrug JS-K. Compound **14a** arrested the cells in the S phase while **14b** arrested the cells in the G2/M phase of the cell cycle. Compound **14a** has a slight inhibitory activity toward the EGFR/MEK/MAPK pathway as shown by In-Cell-Based ELISA screening. It also showed a significant reduction in MRP2 expression in the HepG2-R cell line in a dose-dependent manner, demonstrating a significant impact on the chemotherapeutic resistance. The molecular docking studies were found to be well correlated with cell study. The findings indicated that ∆-16 unsaturated analog **14a** with *R* configuration around C-19 and furoxan moiety at C-3 improved the anticancer activity twofold more than the corresponding estratriene analog NO-CIEA **17**. The data support further investigation of **14a** as a promising anti-liver cancer agent.

## Figures and Tables

**Table 1:** Addition of TMSCN to α,β-enone **8**.

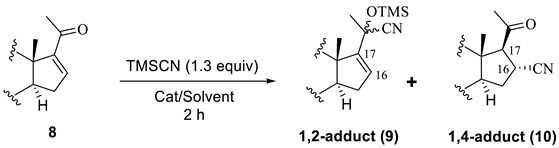					
Entry	Catalyst	Solvent	Temp (°C)	% Yield (9:10)	% Recovery (8)
1	-	THF	r.t. or reflux	0	95
2	LiOC_2_H_5_ (0.05 equiv) [28]	THF	r.t.	0:25	60
3	KCN (0.1 equiv)18-crown-6 (0.1 equiv) [27]	DCM	0–r.t.	0:50	30
4	ZnI_2_ (0.03 equiv)	THF	r.t.	20:12	31
5	ZnI_2_ (1 equiv)	DCM	−20	40:20	3
6	ZnI_2_ (1 equiv)	DCM	−78	0	94
7	Cu(OTf)_2_ (5 mol %)	DCM–MeCN	r.t.	30:20	20

**Table 2:** Anticancer activity of the NO-∆-16-CIEA **14a**,**b**, **17a**–**j**, and **20a**–**d** in human cancer cell lines.

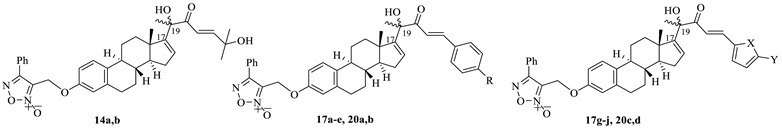								
Compound	C-19	R	X	Y	IC_50_ (µM)		ClogP ^b^	PSA ^b,c^
					HepG2	HepG2-R		
14a	*R*				2.40 ± 0.44	3.74 ± 0.66	6.39	118.25
14b	*S*				8.10 ± 0.92	6.47 ± 1.41	6.39	118.25
17a	*R*	CF_3_			9.00 ± 0.44	>50	8.66	98.02
17b	*S*	CF_3_			>50	>50	8.66	98.02
17c	*R*	SCH_3_			>50	>50	8.39	98.02
17d	*S*	SCH_3_			>50	>50	8.39	98.02
17e	*R*	OCH_3_			>50	>50	8.10	107.26
17f	*R*	F			>50	>50	8.19	98.02
17g	*R*		S	CH_3_	24.00 ± 3.80	>50	7.98	98.02
17h	*S*		S	CH_3_	25.00 ± 3.12	>50	7.98	98.02
17i	*R*		S	Br	>50	>50	8.53	98.02
17j	*R*		O	Br	>50	>50	8.05	111.16
20a	*S*	OCH_3_			>50	>50	8.10	107.26
20b	*S*	F			39.07 ± 5.64	44.48 ± 3.54	8.19	98.02
20c	*S*		S	Br	>50	>50	8.53	98.02
20d	*S*		O	Br	>50	>50	8.05	111.16
Erlotinib ^a^					25.00 ± 0.47	47.16 ± 3.02	2.79	74.75
CUCs D					26.00 ± 0.20	--	2.13	132.12
JS-K					7.01 [33]	17.84 ± 1.58	1.99	174.78

^a^ Positive control, ^b^ Calculated by Molinspiration, ^c^ Molecular polar surface area.

**Table 3:** Anticancer activity of the ∆-16-CIEA **13a**,**b** and furoxan alcohol in HepG2 and HepG2-R cell lines.

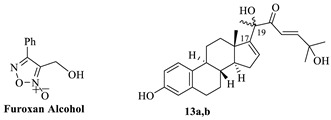					
Compound	C-19	IC_50_ (µM)		ClogP ^b^	PSA ^b,c^
		HepG2	HepG2-R		
Erlotinib ^a^		25.00 ± 0.47	47.16 ±3.02	2.79	74.75
13a	R	18.87 ± 2.40	24.22 ± 0.72	3.93	77.75
13b	S	>50	>50	3.93	77.75
Furoxan alco. [16]	-	>50	>50	1.60	71.72

^a^ Positive control, ^b^ Calculated by Molinspiration [35], ^c^ Molecular polar surface area.

**Table 4:** EON results of shape and electrostatic similarity between the designed compounds and the query NO-CIEA **17**.

Cpd. No.	ET_coul *	ET_pb **	ShapeTanimoto ***	ET_combo ****	Rank
20c	0.093	0.115	0.354	0.469	1
20b	0.084	0.11	0.344	0.453	2
17h	0.076	0.103	0.344	0.447	3
17d	0.087	0.112	0.335	0.447	4
20a	0.077	0.105	0.332	0.437	5
14b	0.062	0.096	0.341	0.436	6
17i	0.079	0.118	0.315	0.433	7
20d	0.079	0.118	0.314	0.432	8
17j	0.079	0.118	0.314	0.432	9
17c	0.067	0.086	0.335	0.421	10
17f	0.102	0.134	0.286	0.420	11
17b	0.11	0.127	0.289	0.416	12
17a	0.106	0.135	0.28	0.415	13
17g	0.093	0.127	0.286	0.413	14
14a	0.093	0.104	0.306	0.410	15
17e	0.056	0.077	0.332	0.409	16
NO-CIEA **17**	EON query (Reference compound)				

* The value of electrostatic Tanimoto (ET) using only the coulombic part of Poisson–Boltzmann (PB) electrostatics. ** The value of ET using full PB electrostatics. *** The shape Tanimoto between the given molecule and the query. **** Sum of ET-pb and EON-shape-tani.

**Table 5:** FRED Chemgauss4 score for the designed compounds, lead compound NO-CIEA **17** and erlotinib docked with EGFR, MEK1, Erk2, and MRP1 protein active sites.

EGFR (1M17)		MEK1 (1S9J)		Erk2 (5NHJ)		MRP1 (2CBZ)	
Cpd. No.	Chemgauss4 Score	Cpd. No.	Chemgauss4 Score	Cpd. No.	Chemgauss4 Score	Cpd. No.	Chemgauss4 Score
20b	−8.6334	14a	−11.0399	14a	−12.7475	17g	−3.5027
17f	−7.6956	14b	−9.6458	17f	−12.3146	17c	−2.5383
17h	−7.0982	17j	−9.3788	17g	−11.2281	17i	−1.7834
14a	−7.0530	20d	−9.3788	14b	−11.1280	14b	−1.6776
17g	−7.0466	17i	−9.0964	17i	−11.0947	14a	−1.6713
20a	−6.9339	17g	−8.9700	17e	−11.0571	17e	−1.6579
14b	−6.7689	17f	−8.9029	17j	−10.3963	17f	−1.4649
17j	−6.5061	17h	−7.3359	20d	−10.3963	17j	−1.1089
20d	−6.5061	17d	−7.0771	17a	−10.3073	20d	−1.1089
20c	−6.1182	17c	−6.8698	20c	−9.1946	20b	−0.9622
17d	−5.8920	20c	−6.4424	20b	−9.0891	17h	−0.3698
17e	−5.7812	17b	−6.2935	20a	−8.6158	20c	−0.3186
17i	−5.5075	20b	−6.2171	17d	−8.3840	17d	0.1454
17b	−5.3954	20a	−6.1073	17c	−8.3759	17a	0.5193
17a	−5.2240	17a	−6.0166	17h	−8.3408	20a	0.5344
17c	−4.4860	17e	−5.8291	17b	−7.4512	17b	0.8334
L *	−6.7089	L *	−10.4221	L *	−9.9372	L *	−1.5337
E **	−10.8300	E **	−10.7400	E **	−12.7400	E **	−2.9000

* L = Lead compound NO-CIEA 17. ** E = Erlotinib.

## Data Availability

The data presented in this study are available in the Supplementary Materials.

## Supplementary Materials

The following supporting information can be downloaded at https://www.mdpi.com/article/10.3390/molecules28062754/s1, copies of spectral data for the synthesized compounds are provided in the Supplementary Materials. Figures S1–S99: NMR spectra of compounds **7**–**10, 11a,b, 12a,b, 13a,b,14a,b, 15a**–**j, 16a**–**j, 17a**–**j, 18, 19 and 20a**–**d**.

## Sample Availability

Samples of the compounds are available from the authors.
