# Supplementary material for: Utilizing Estra-1,3,5,16-Tetraene Scaffold: Design and Synthesis of Nitric Oxide Donors as Chemotherapeutic Resistance Combating Agents in Liver Cancer
Source: Molecules. 2023 Mar 18;28(6):2754. doi: 10.3390/molecules28062754 (PMC10055446; doi:10.3390/molecules28062754)
Supplement: Supplementary file 1 [file molecules-28-02754-s001.zip › molecules-2256721-supplementary.pdf]

Utilizing Estra-1,3,5,16-tetraene Scaffold: Design and Synthesis of Nitric Oxide Donors as Chemotherapeutic Resistance Combating Agents in Liver Cancer

Mahrous A. Abou-Salim [1]*, Mohamed A. Shaaban [2], Mohammed K. Abd El Hameid [2], Mohammed M. Alanazi [3], Fathi Halaweish [4], and Yaseen A.M.M. Elshaier [5]

1 Spectral data

1.1 Compound 7

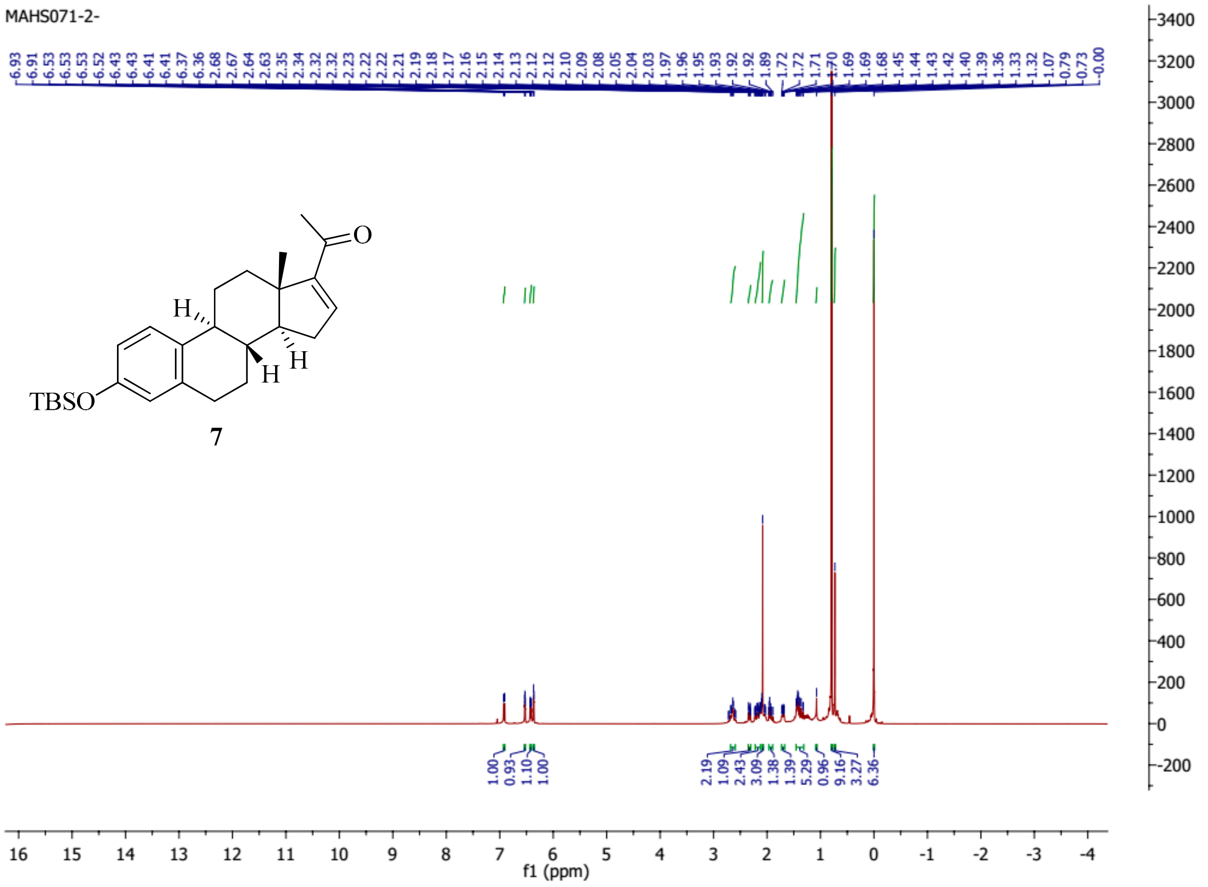

Figure S 1. ^{1}H NMR of compound **7**.

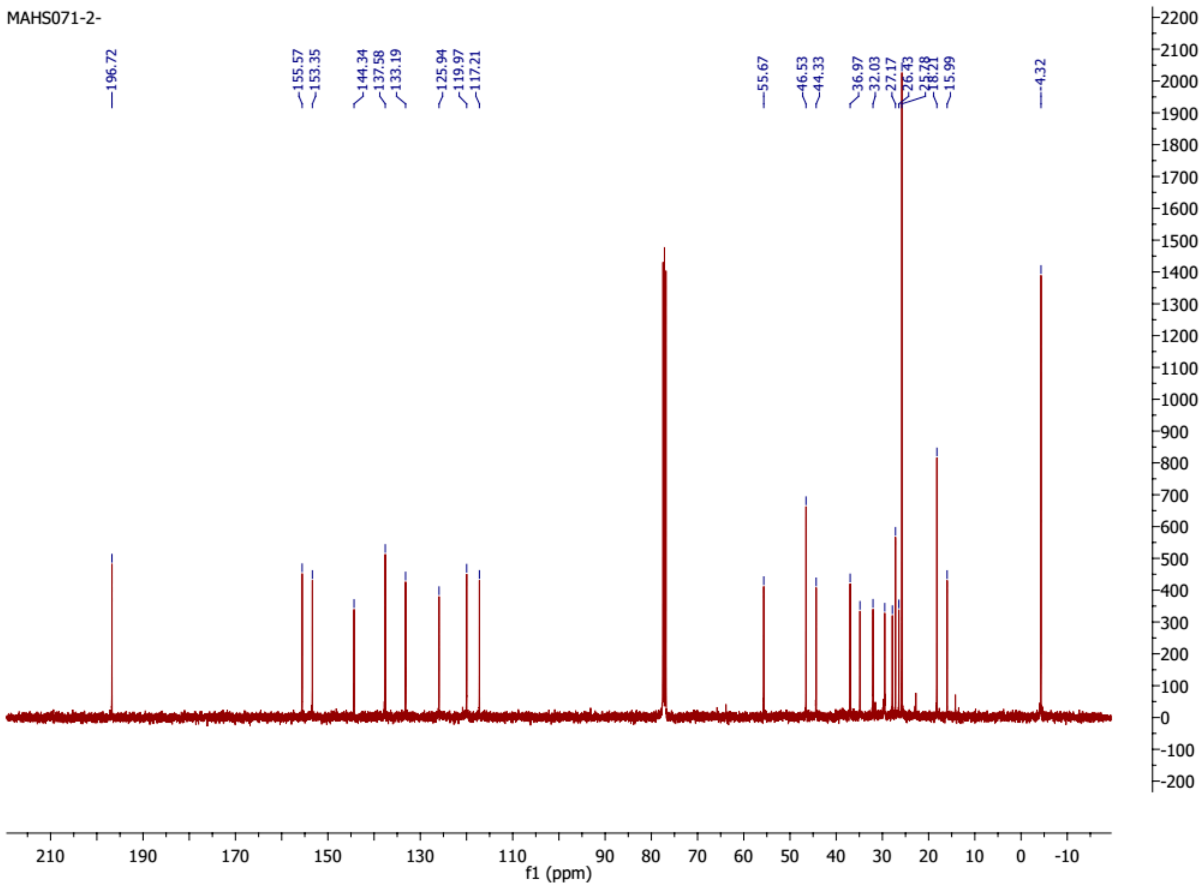

Figure S 2. ^{13}C NMR of compound **7**.

1.2 Compound 8

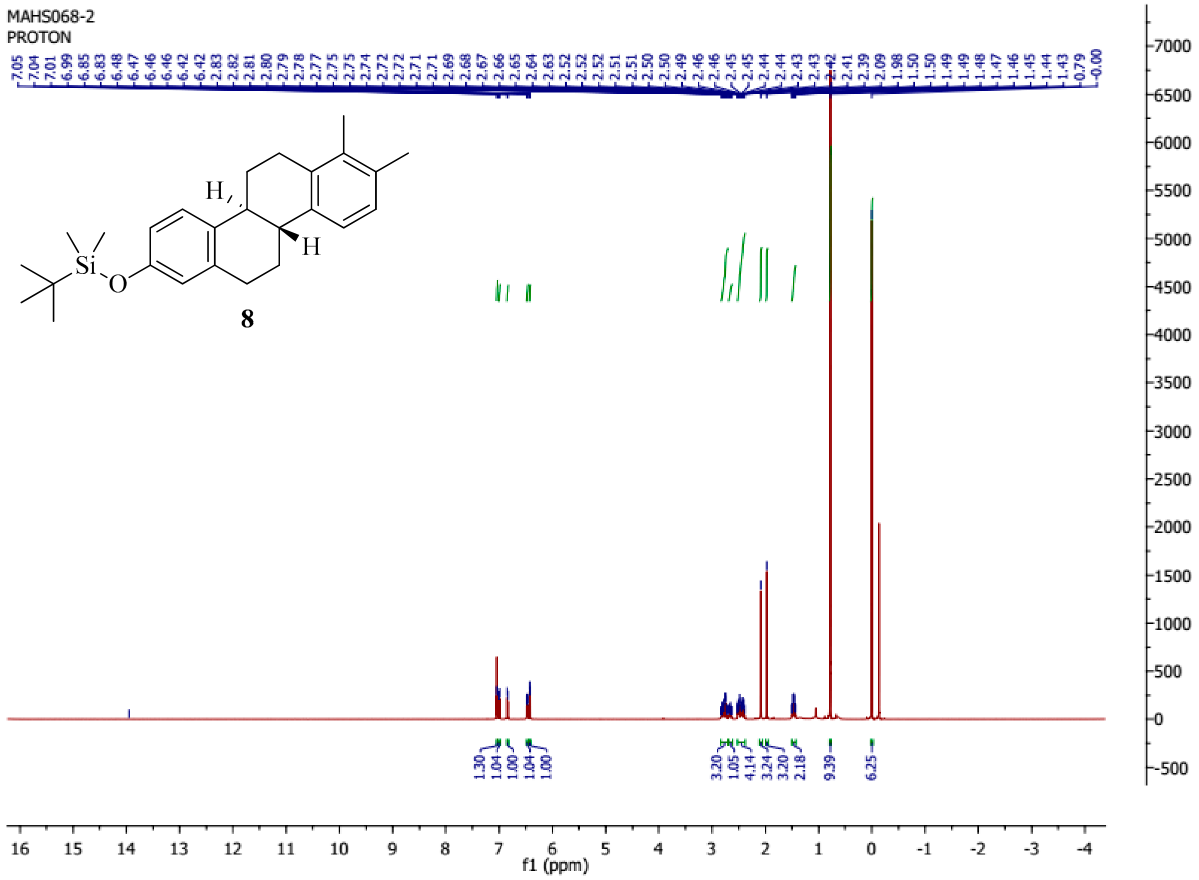

Figure S 3. ^{1}H NMR of compound **8**.

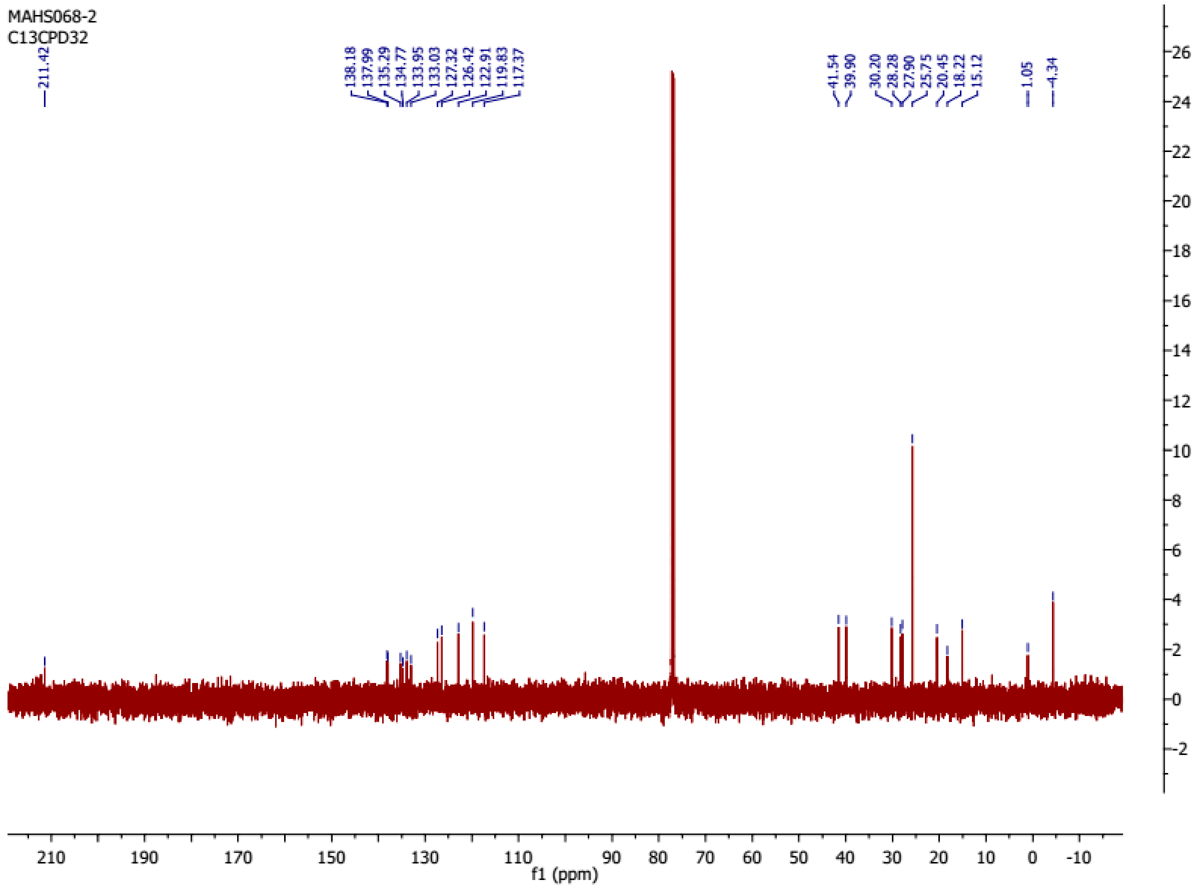

Figure S 4. ^{13}C NMR of compound **8**.

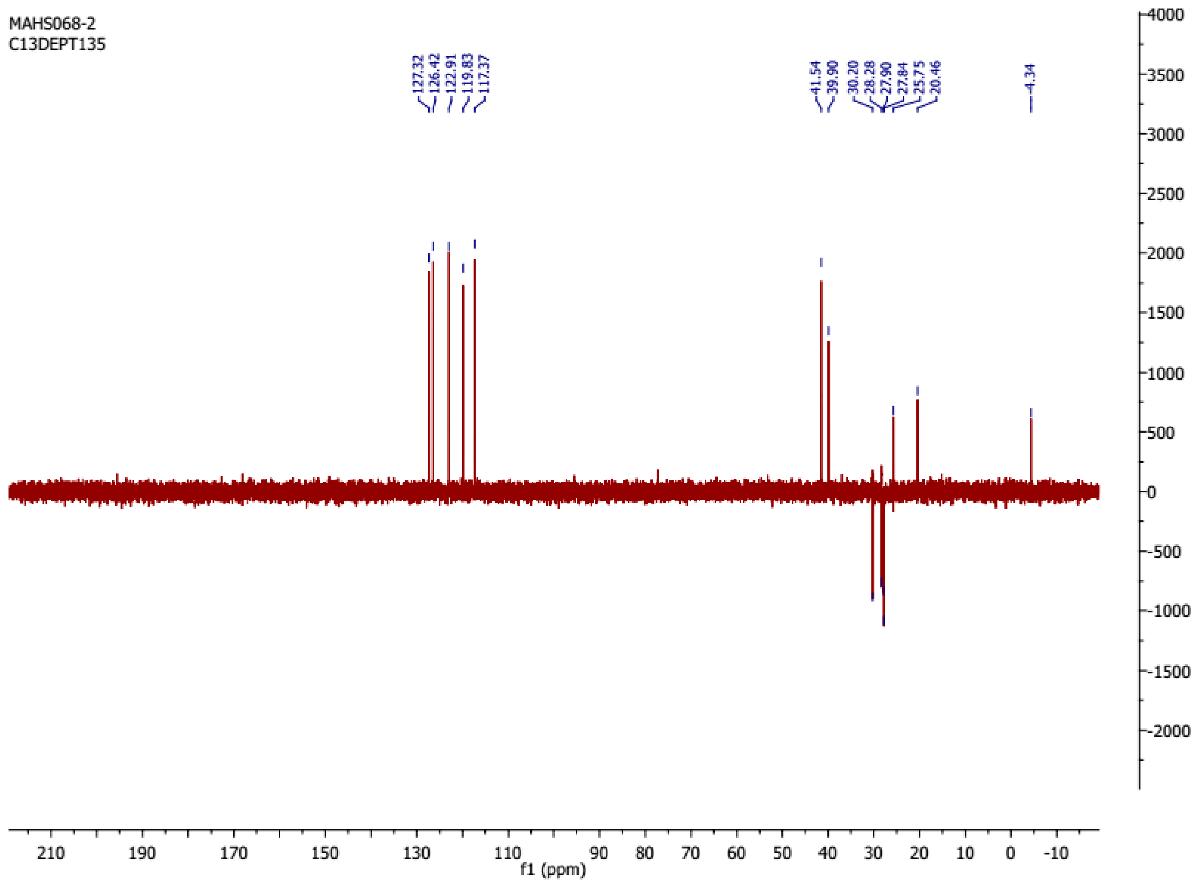

Figure S 5. DEPT135 ^{13}C NMR of compound **8**.

1.3 Compound 9

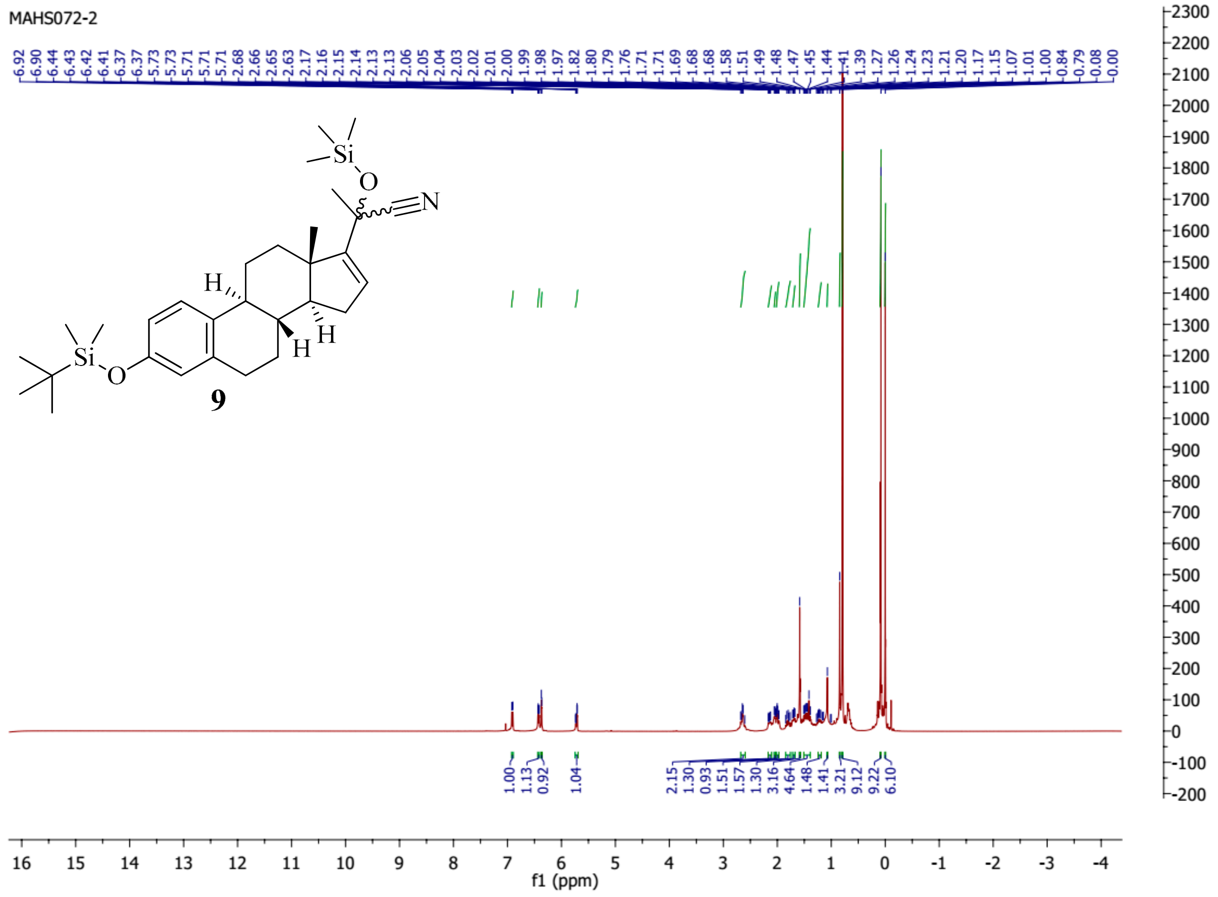

Figure S 6. ^{1}H NMR of compound **9**.

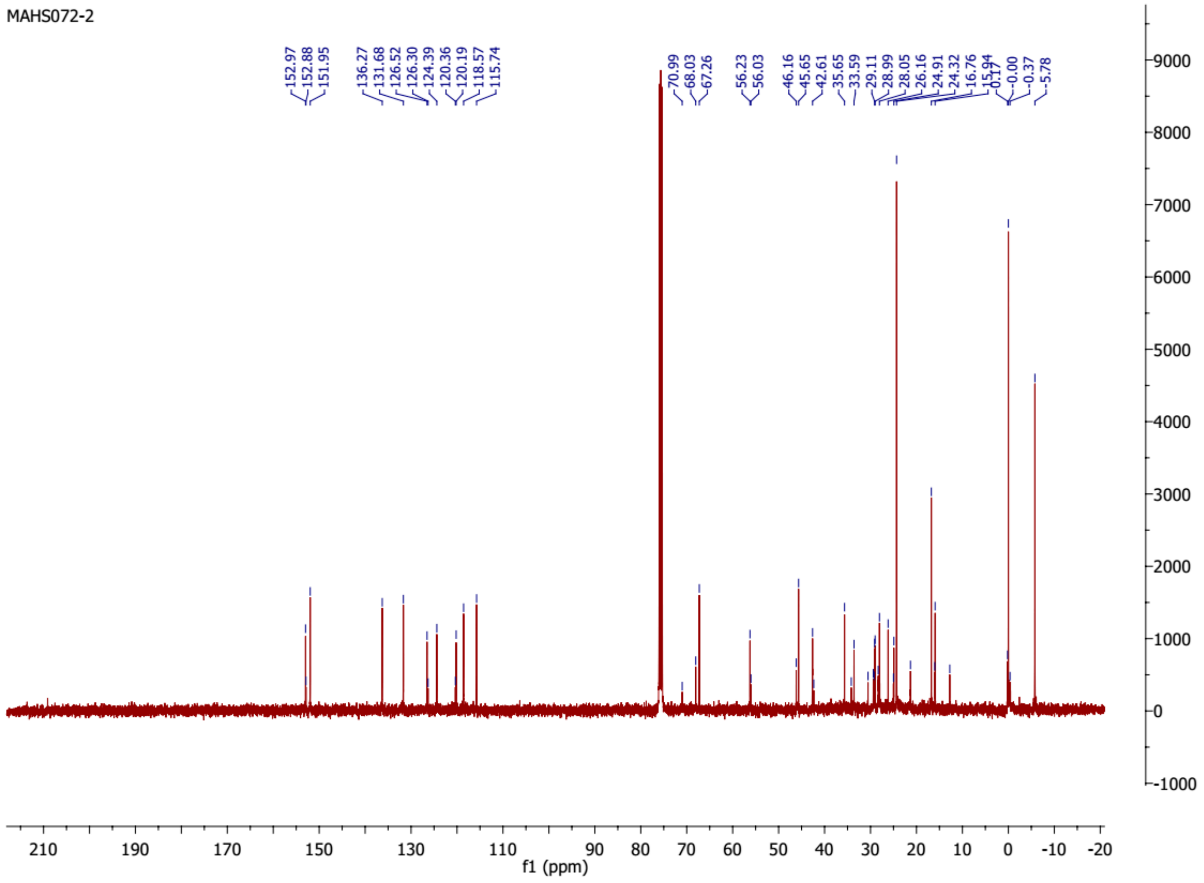

Figure S 7. ^{13}C NMR of compound **9**.

1.4 Compound 10

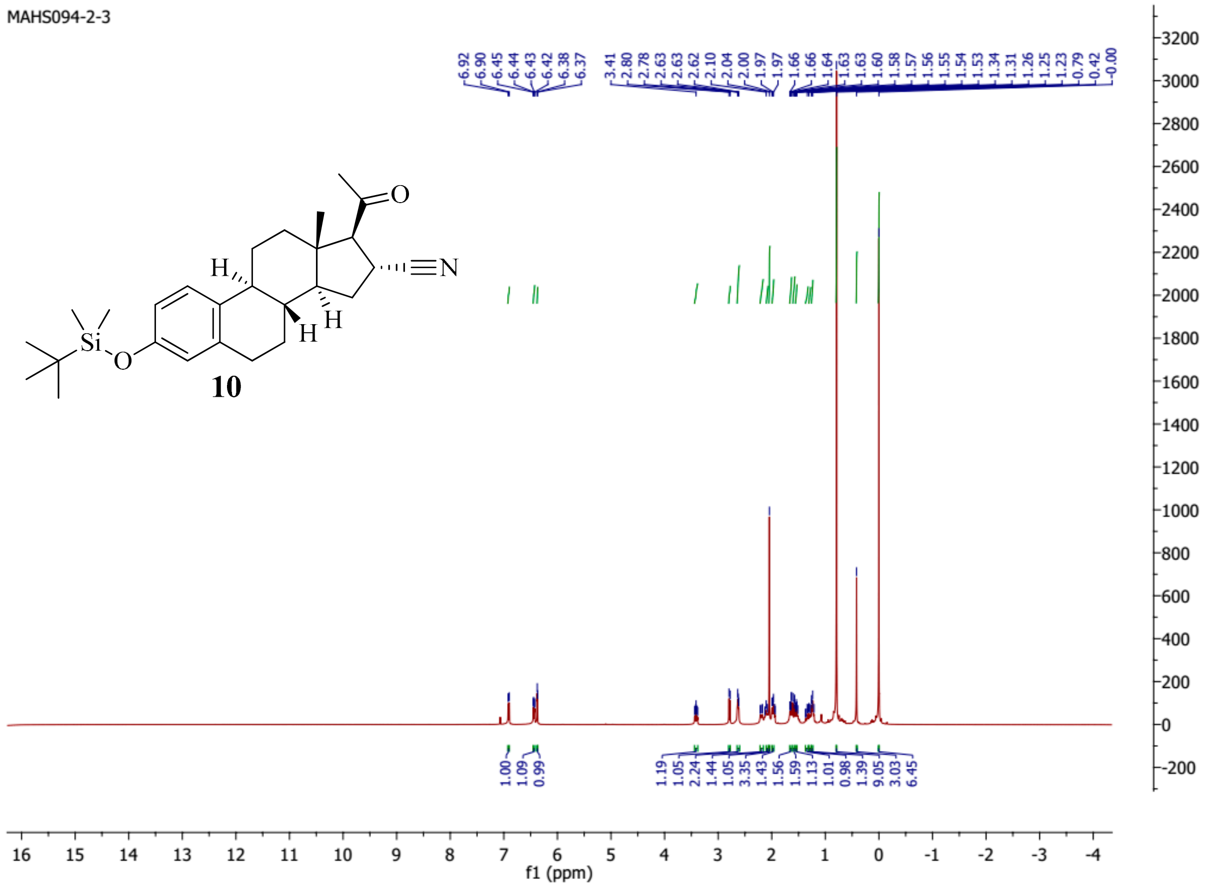

Figure S 8. ^{1}H NMR of compound **10**.

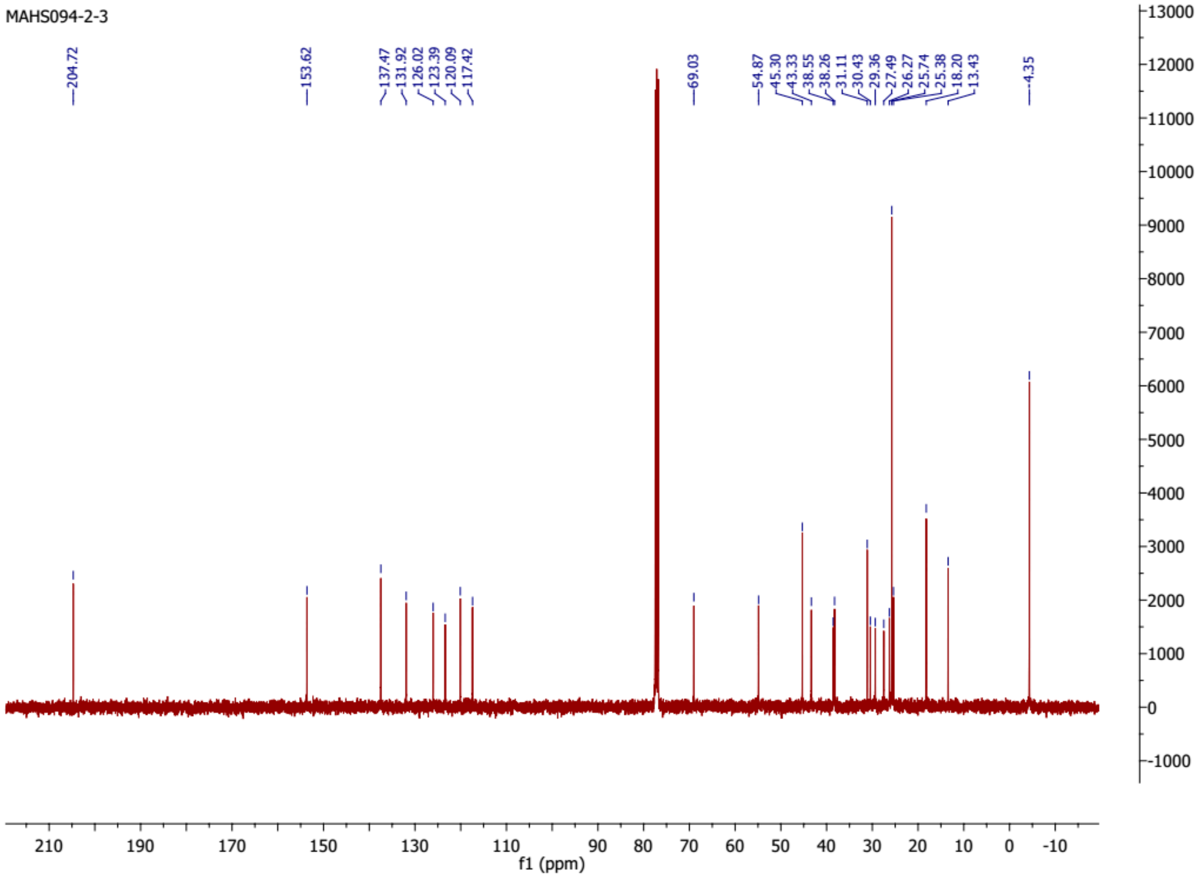

Figure S 9. ^{13}C NMR of compound **10**.

1.5 Compound 11a

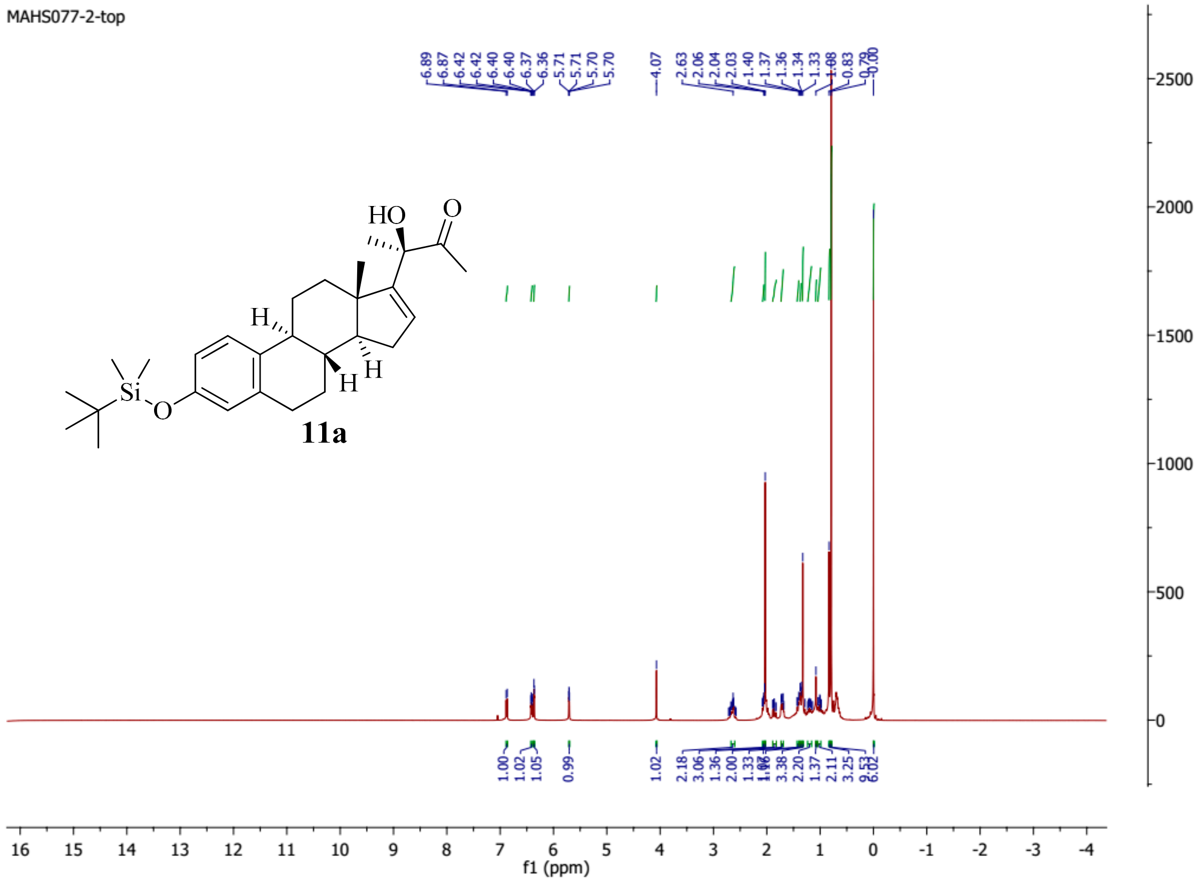

Figure S 10. ^{1}H NMR of compound **11a**.

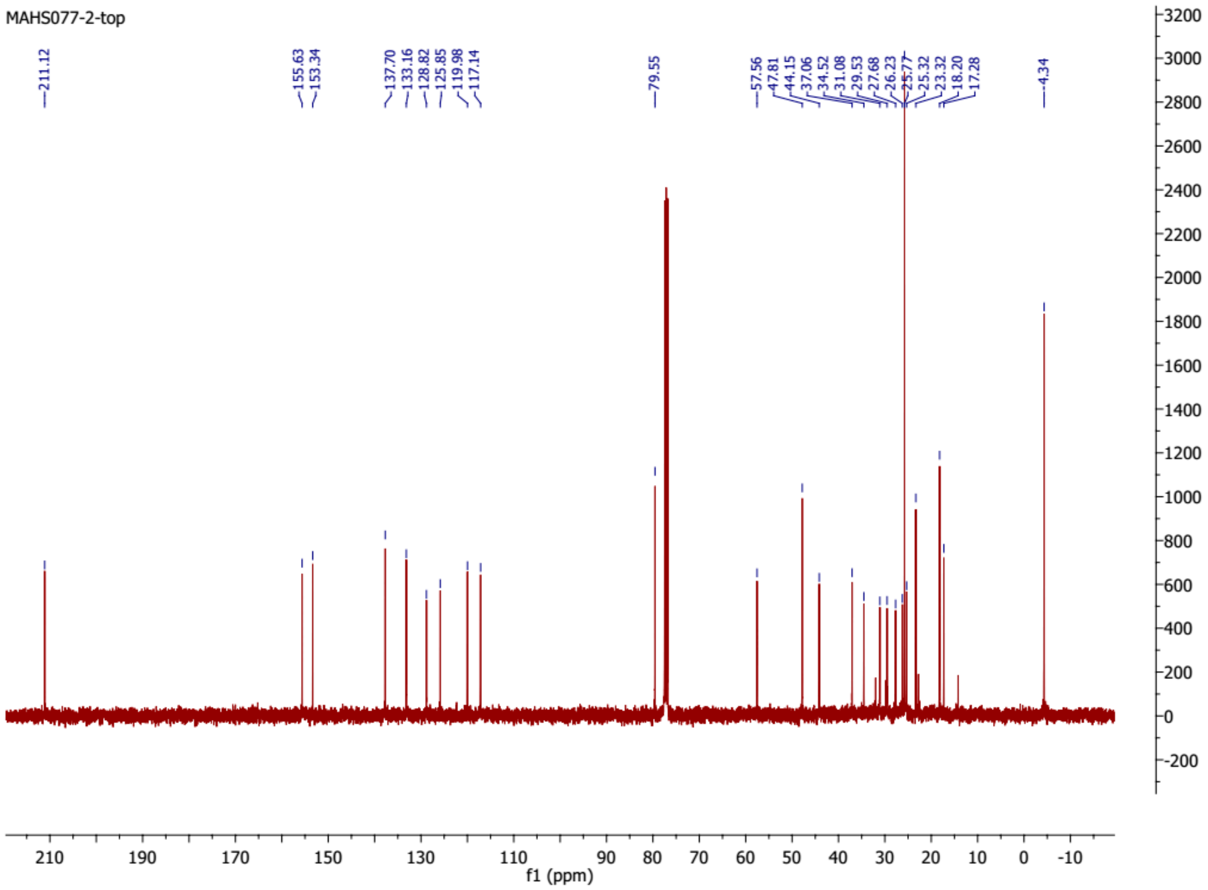

Figure S 11. ^{13}C NMR of compound **11a**.

1.6 Compound 11b

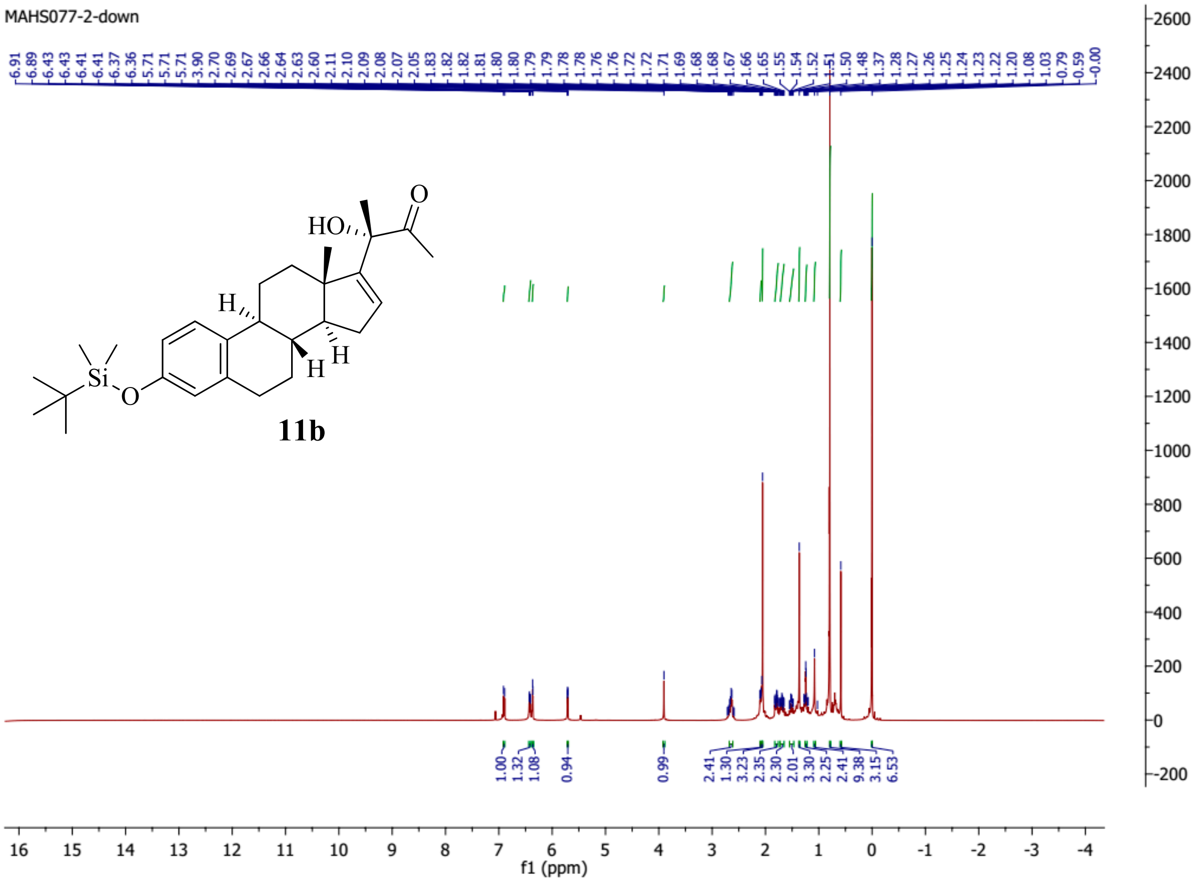

Figure S 12. ^{1}H NMR of compound **11b**.

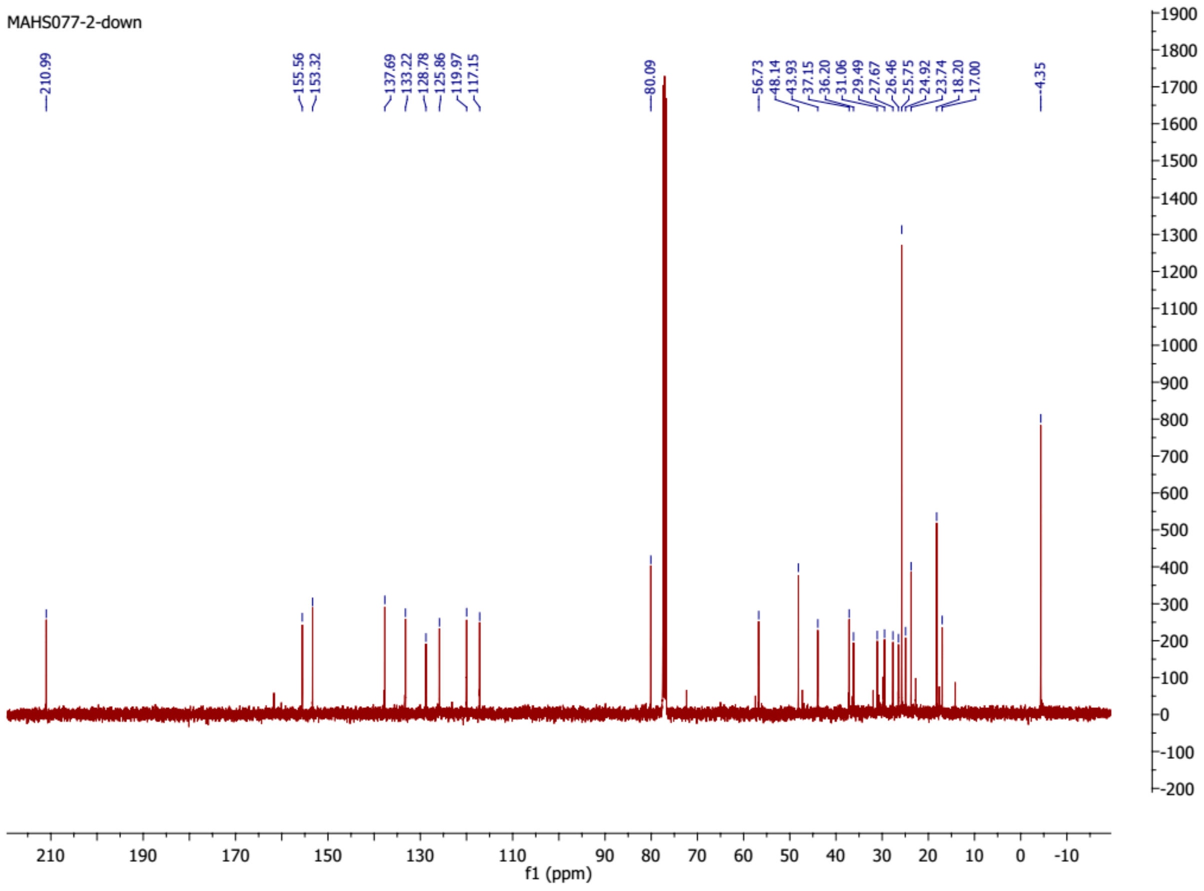

Figure S 13. ^{13}C NMR of compound **11b**.

1.7 Compound 12a

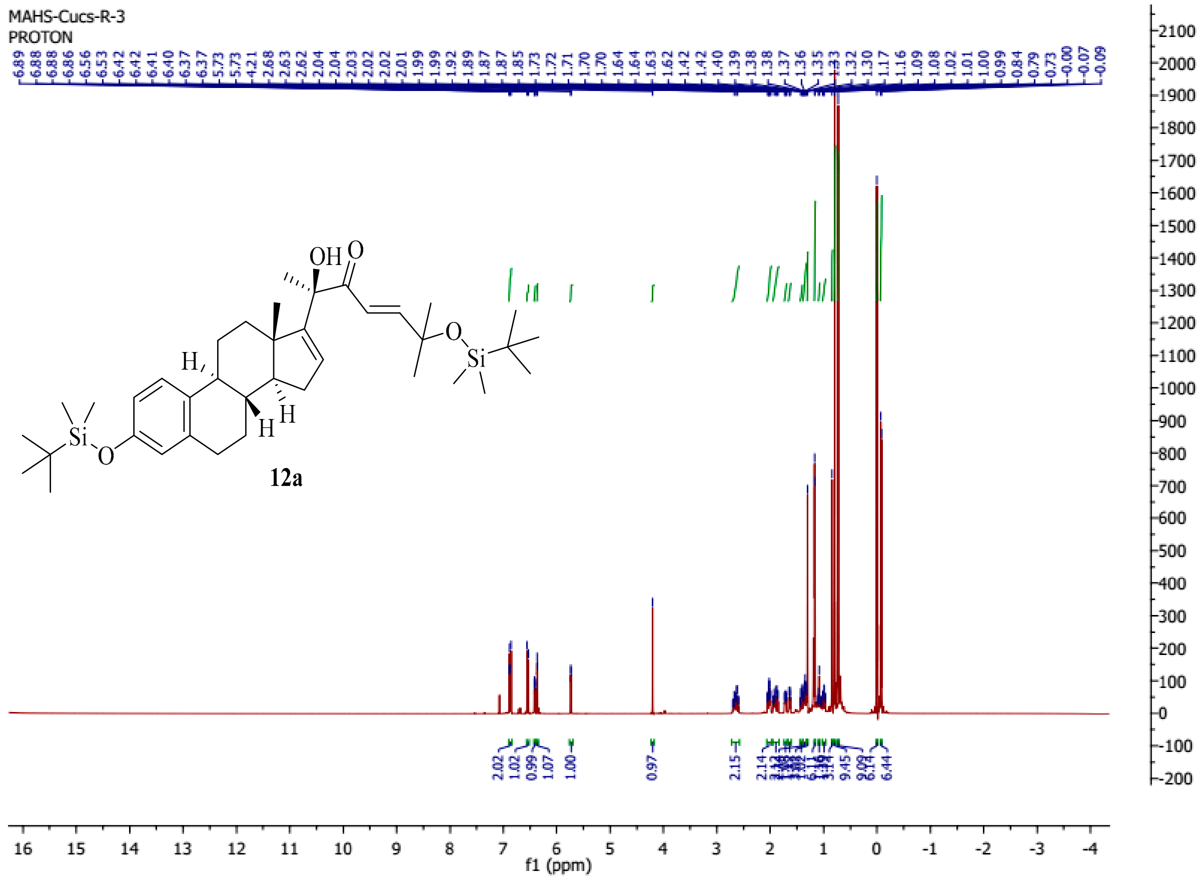

Figure S 14. ^{1}H NMR of compound **12a**.

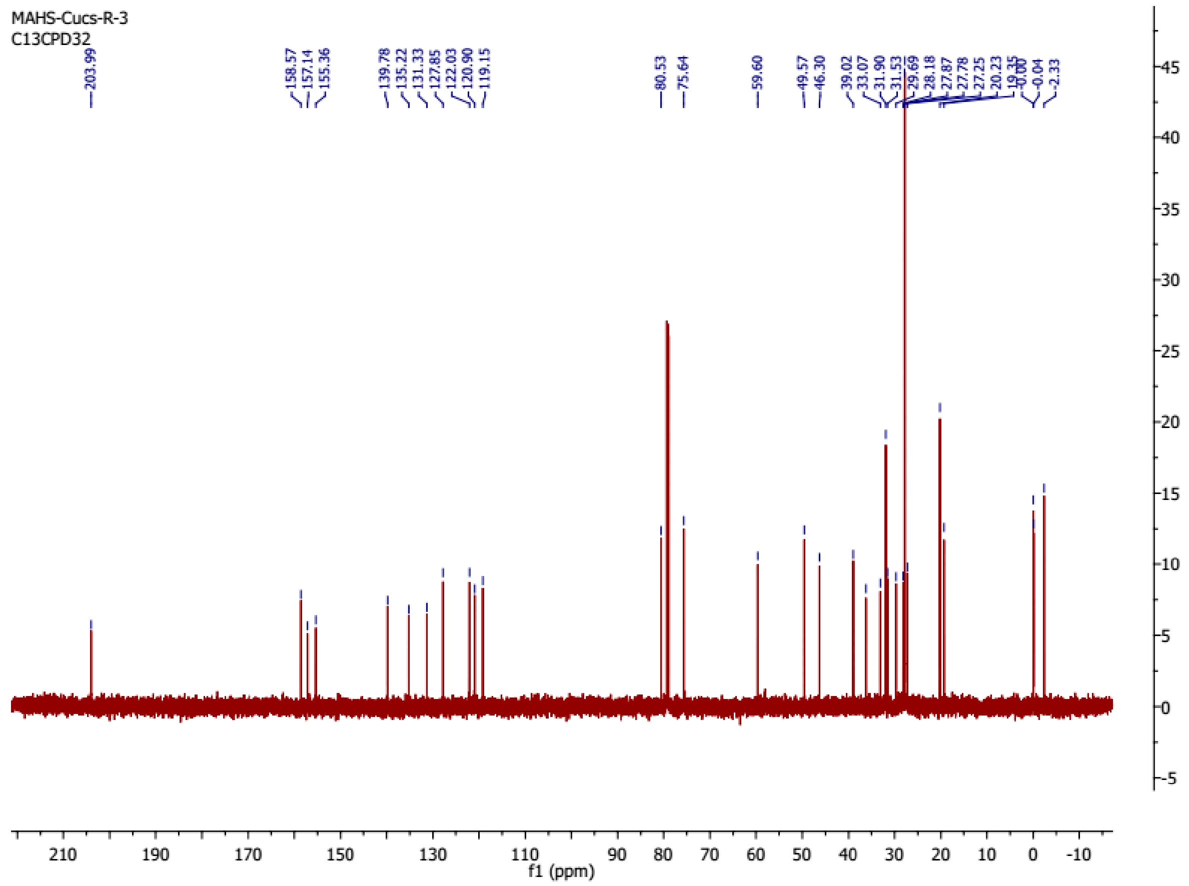

Figure S 15. ^{13}C NMR of compound **12a**.

1.8 *Compound 12b*

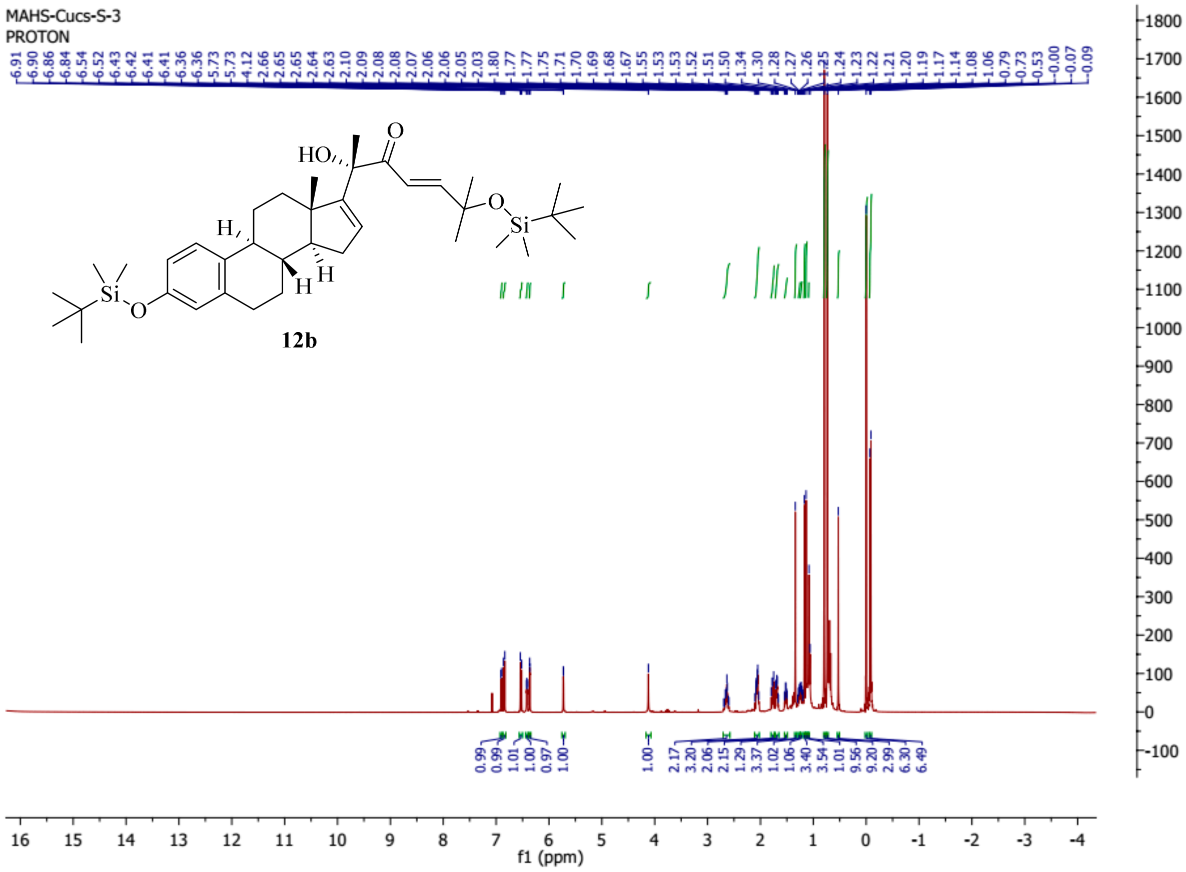

Figure S 16. ^{1}H NMR of compound **12b**.

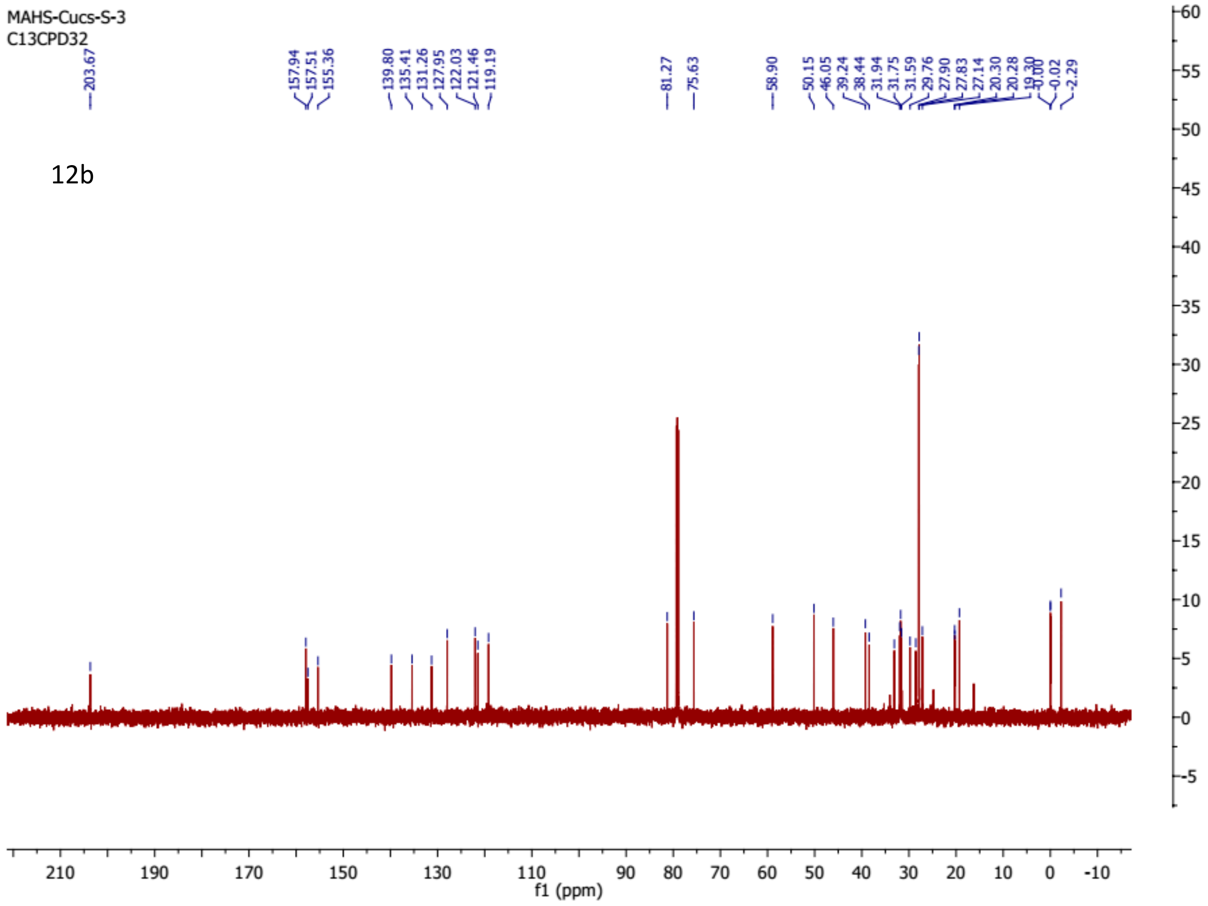

Figure S 17. ^{13}C NMR of compound **12b**.

1.9 Compound 13a

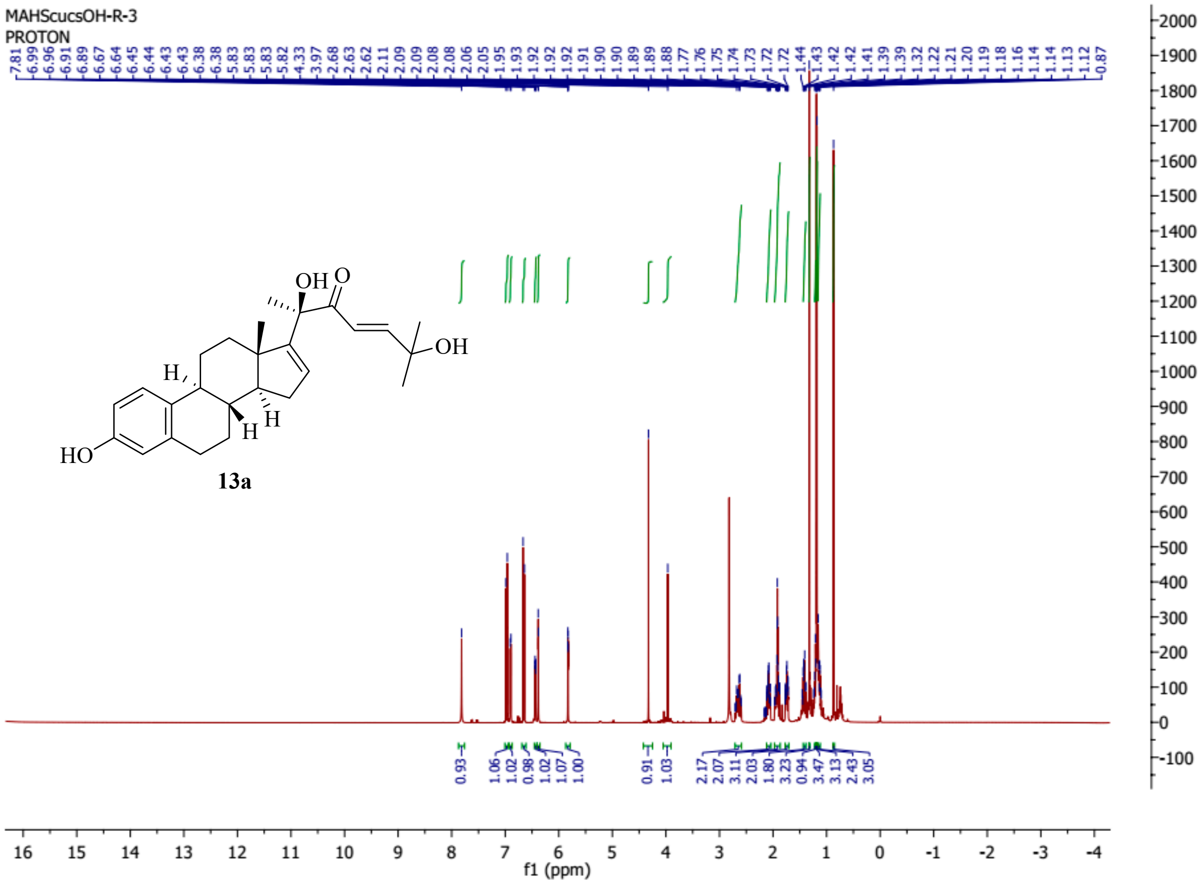

Figure S 18. ^{1}H NMR of compound **13a**.

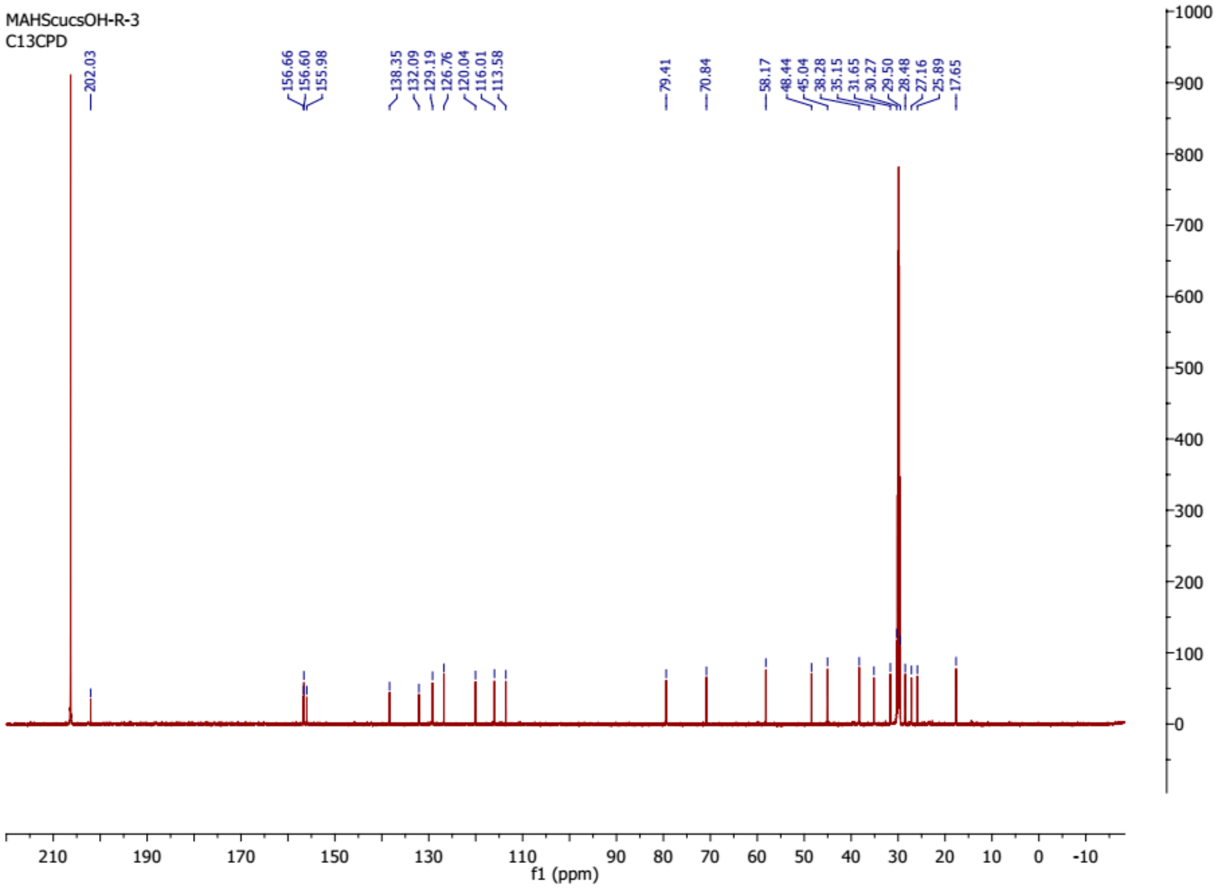

Figure S 19. ^{13}C NMR of compound **13a**.

1.10 Compound 13b

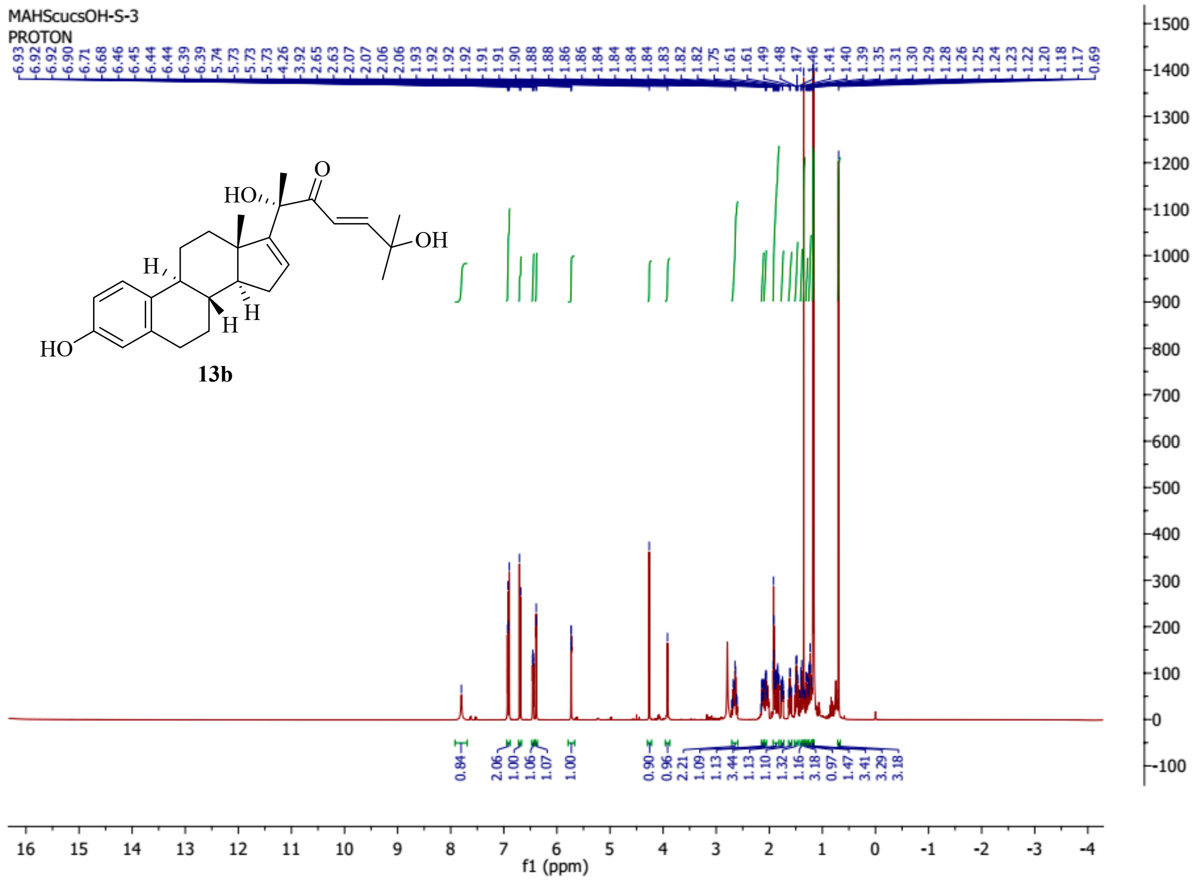

Figure S 20. ^{1}H NMR of compound **13b**.

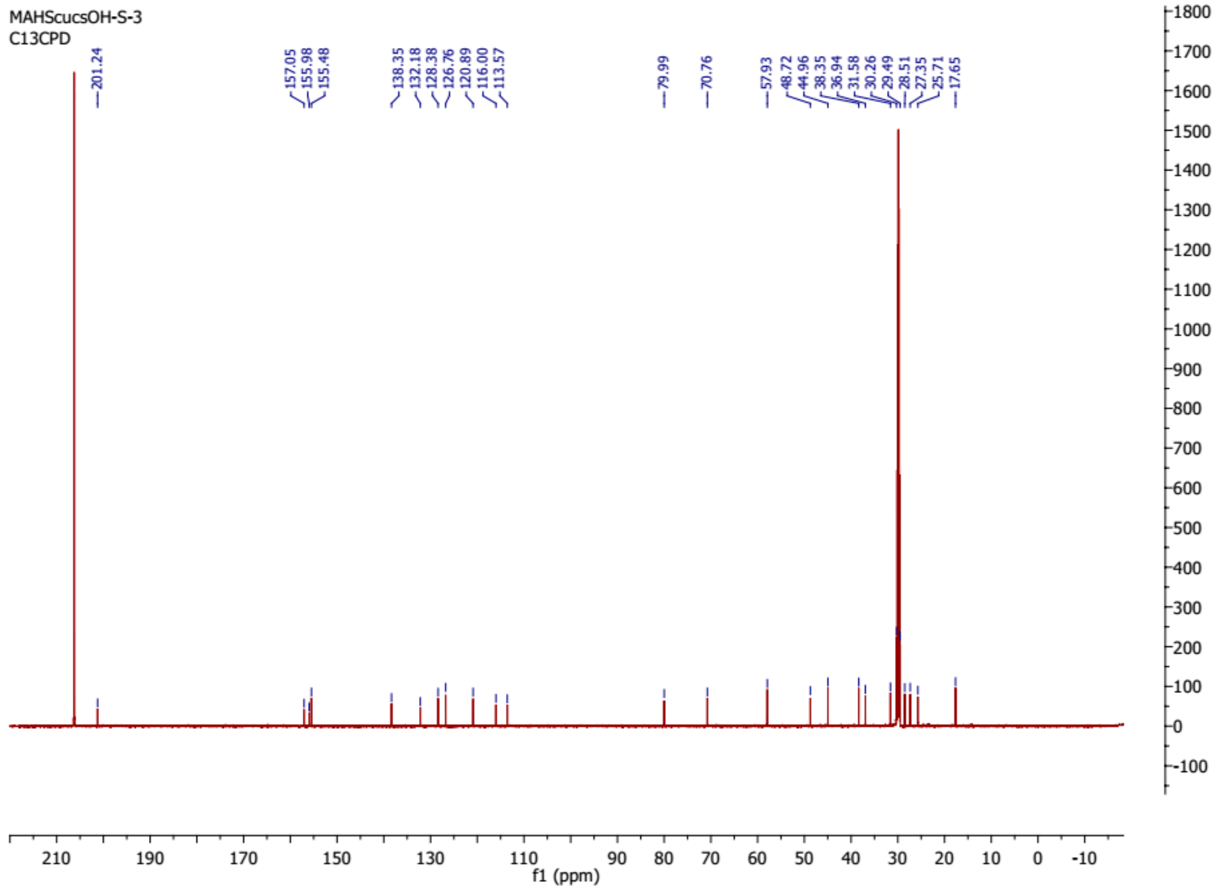

Figure S 21. ^{13}C NMR of compound **13b**.

1.11 *Compound 14a*

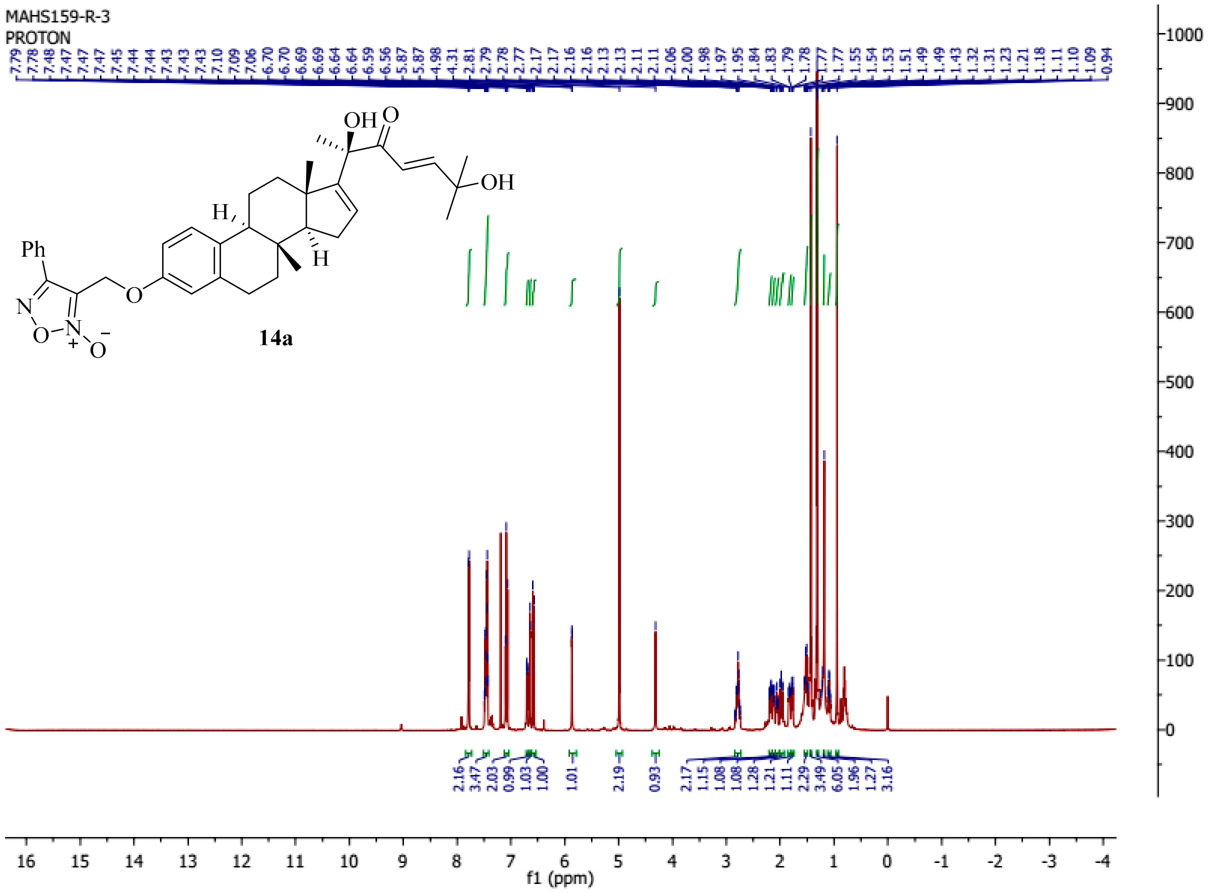

Figure S 22. ^{1}H NMR of compound **14a**.

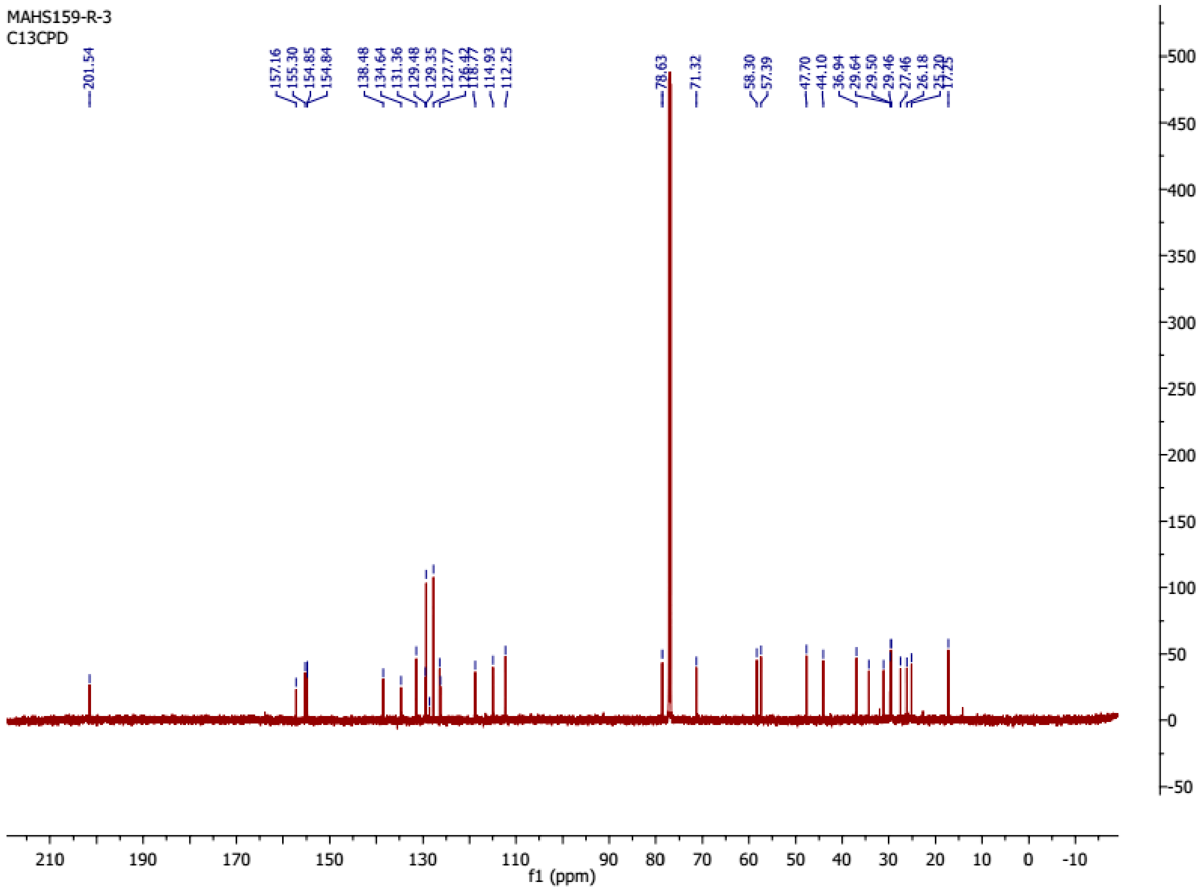

Figure S 23. ^{13}C NMR of compound **14a**.

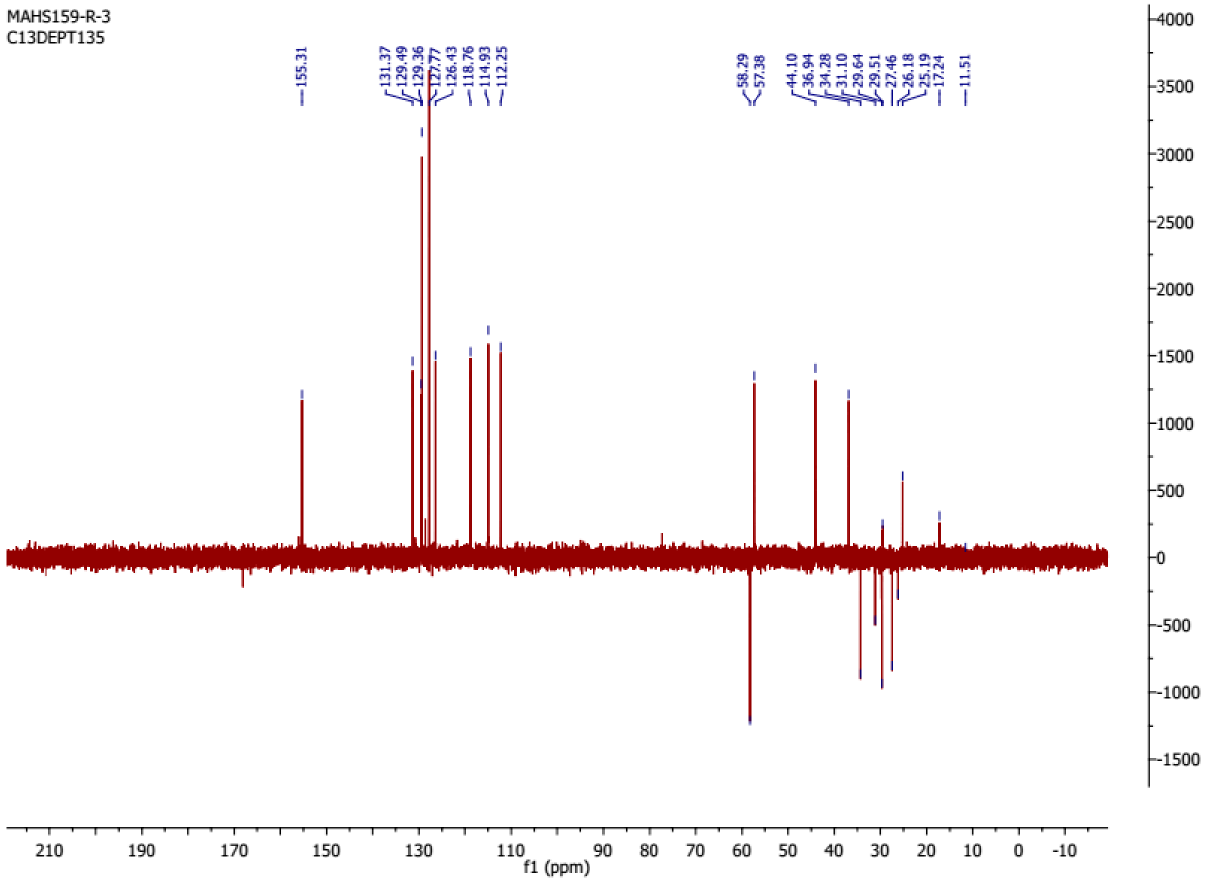

Figure S 24. DEPT135 ^{13}C NMR of compound **14a**.

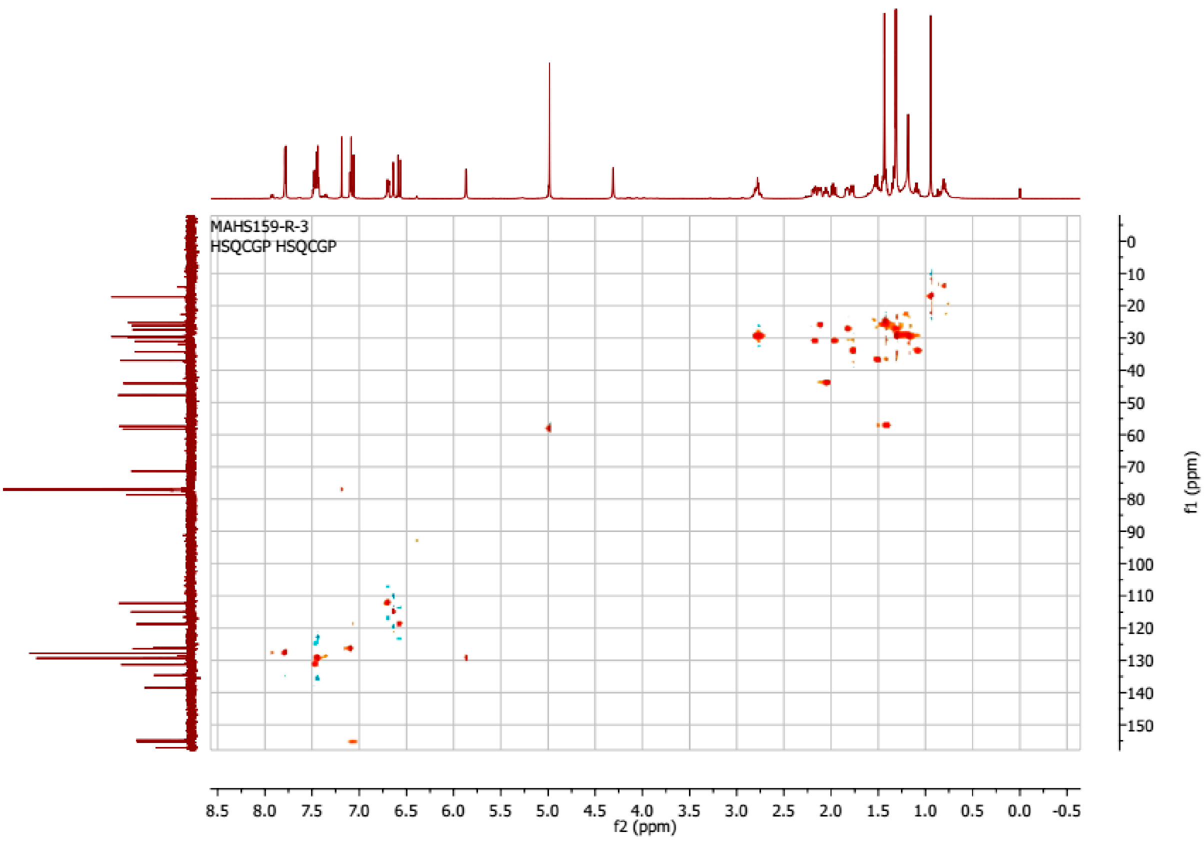

Figure S 25. HSQCGP of compound **14a.**

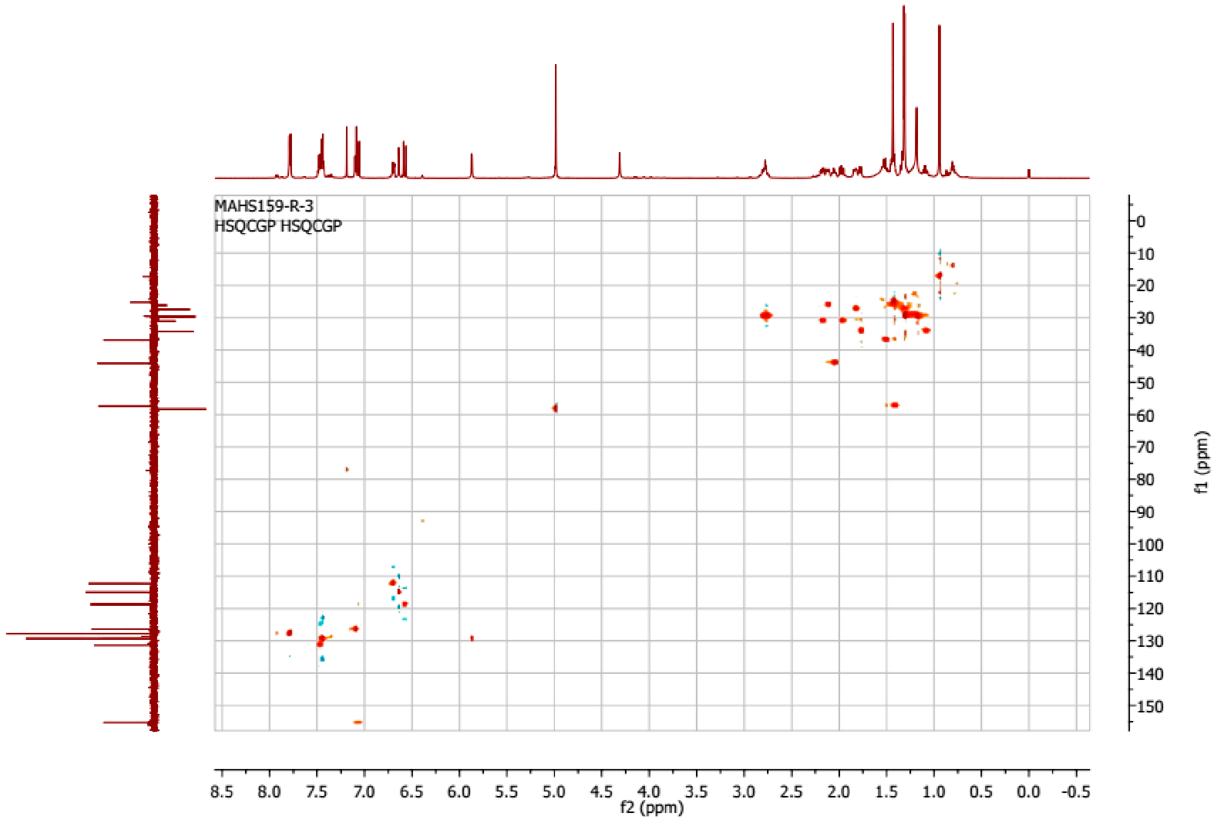

Figure S 26. HSQCGP of compound **14a.**

1.12 Compound 14b

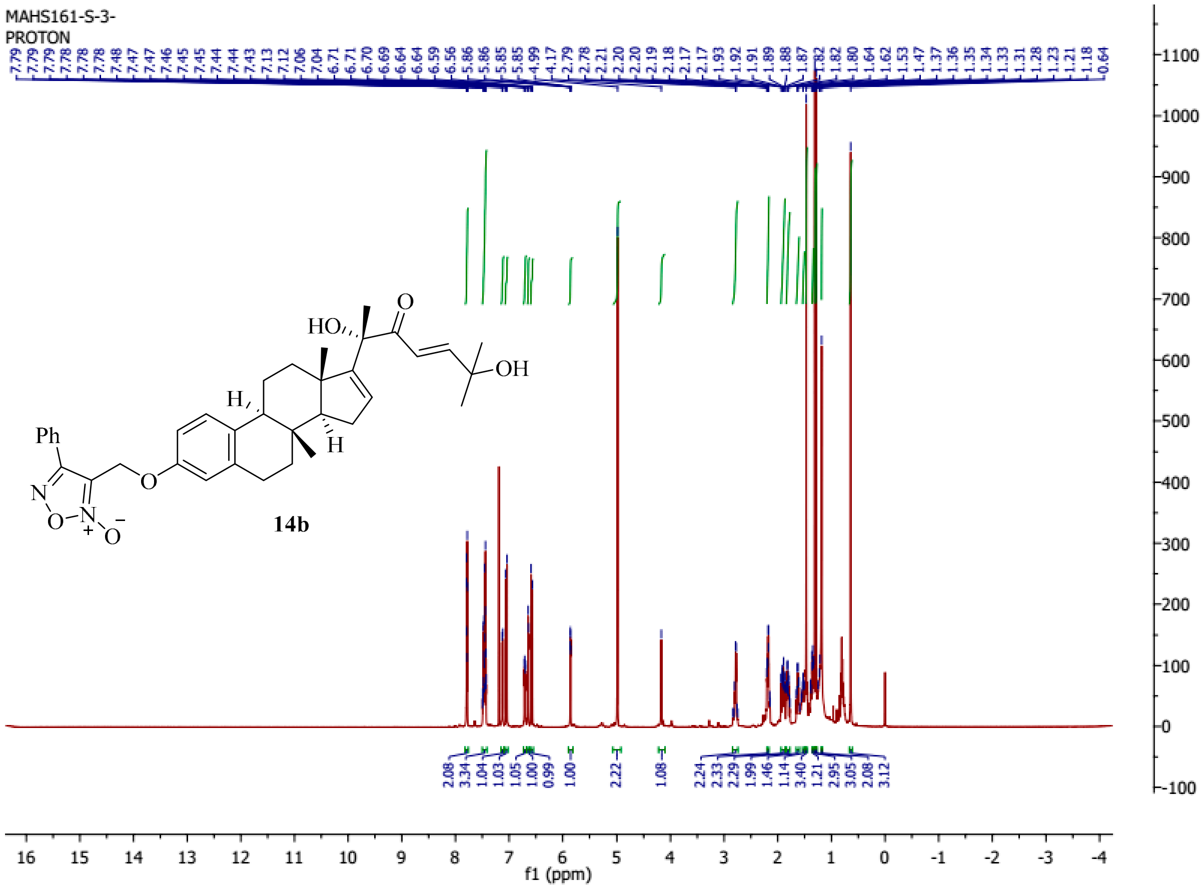

Figure S 27. ^{1}H NMR of compound **14b**.

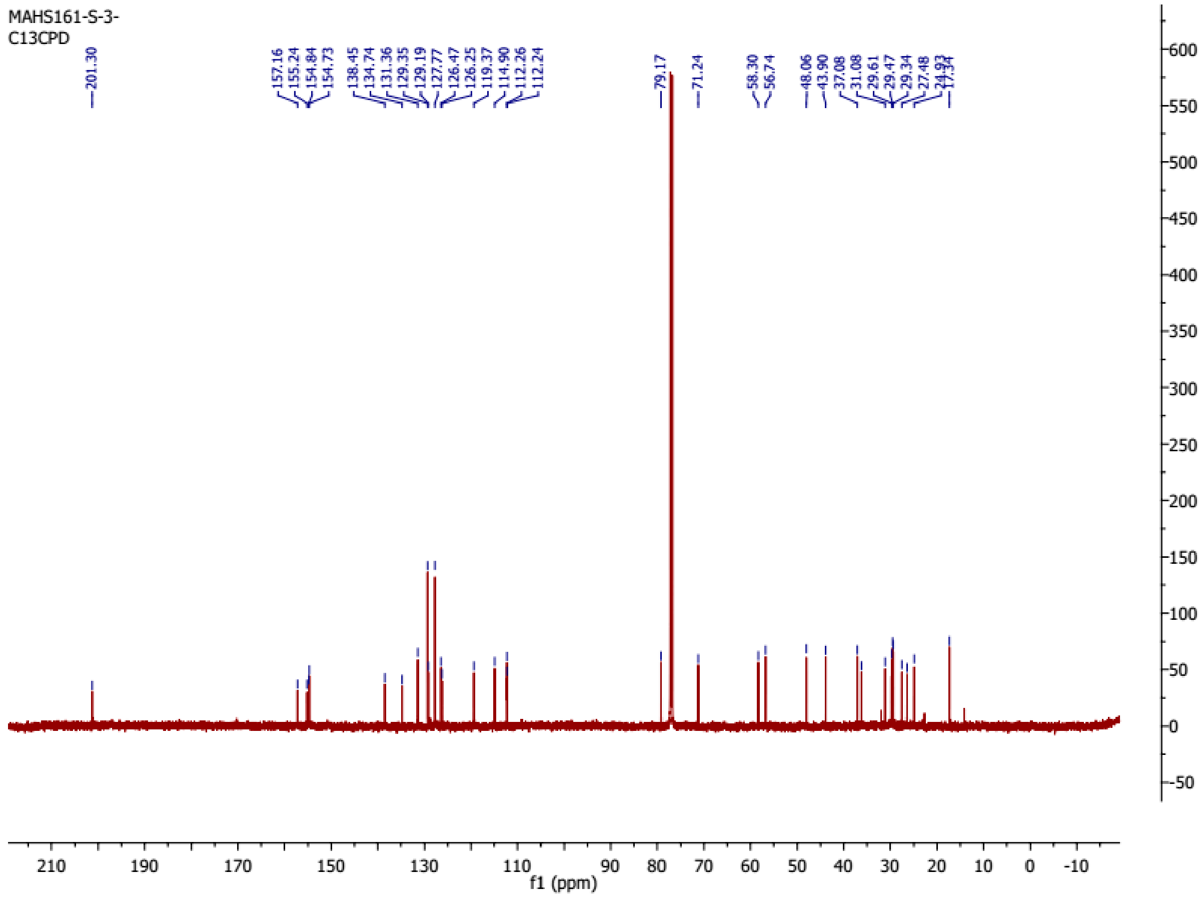

Figure S 28. ^{13}C NMR of compound **14b**.

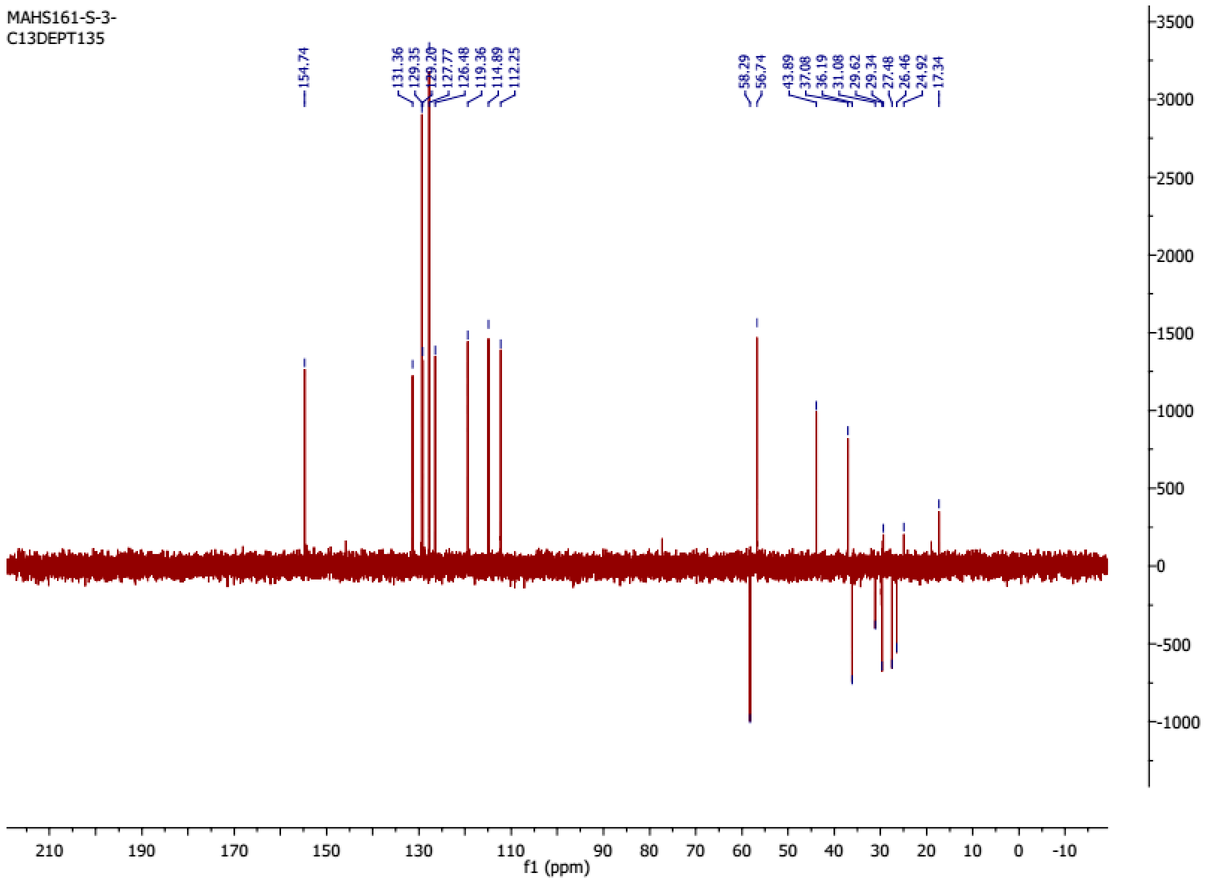

Figure S 29. DEPT135 ^{13}C NMR of compound **14b**.

1.13 *Compound 15a*

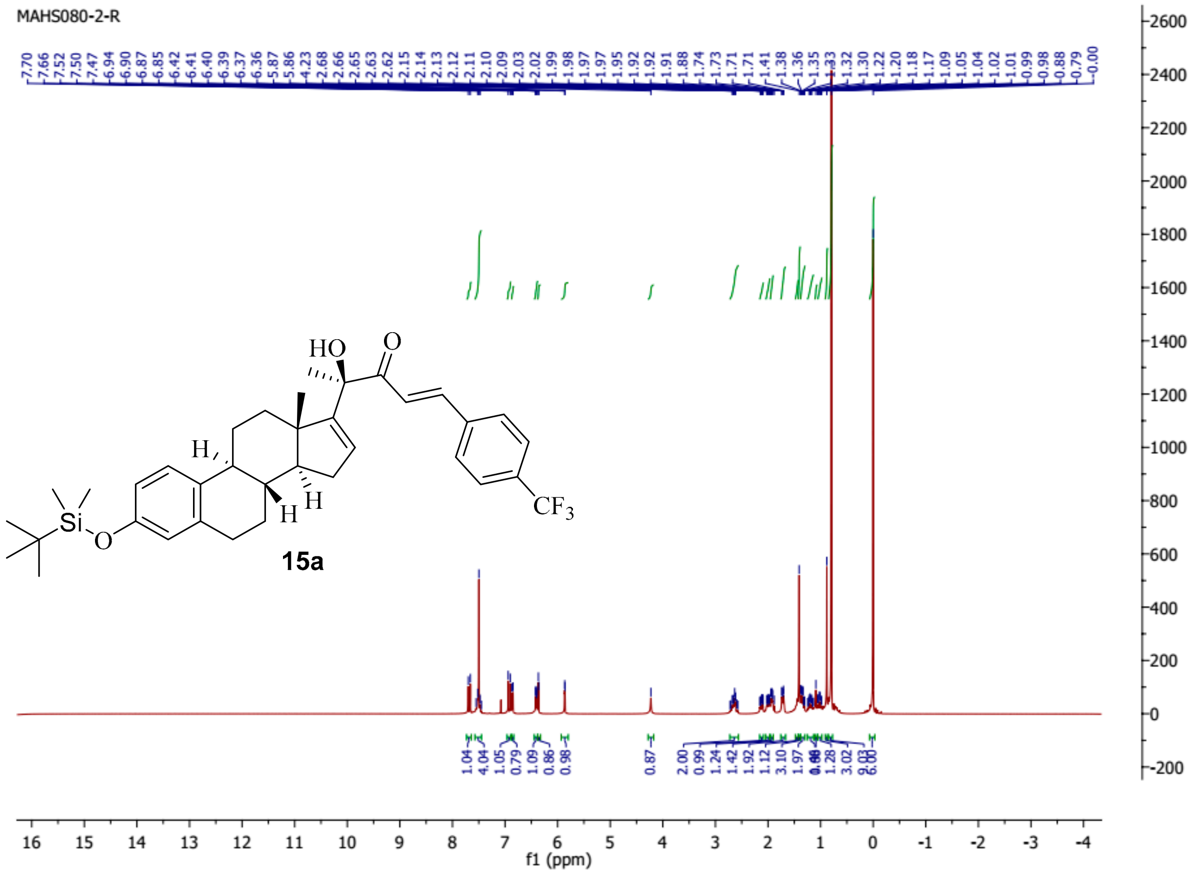

Figure S 30. ^{1}H NMR of compound **15a**.

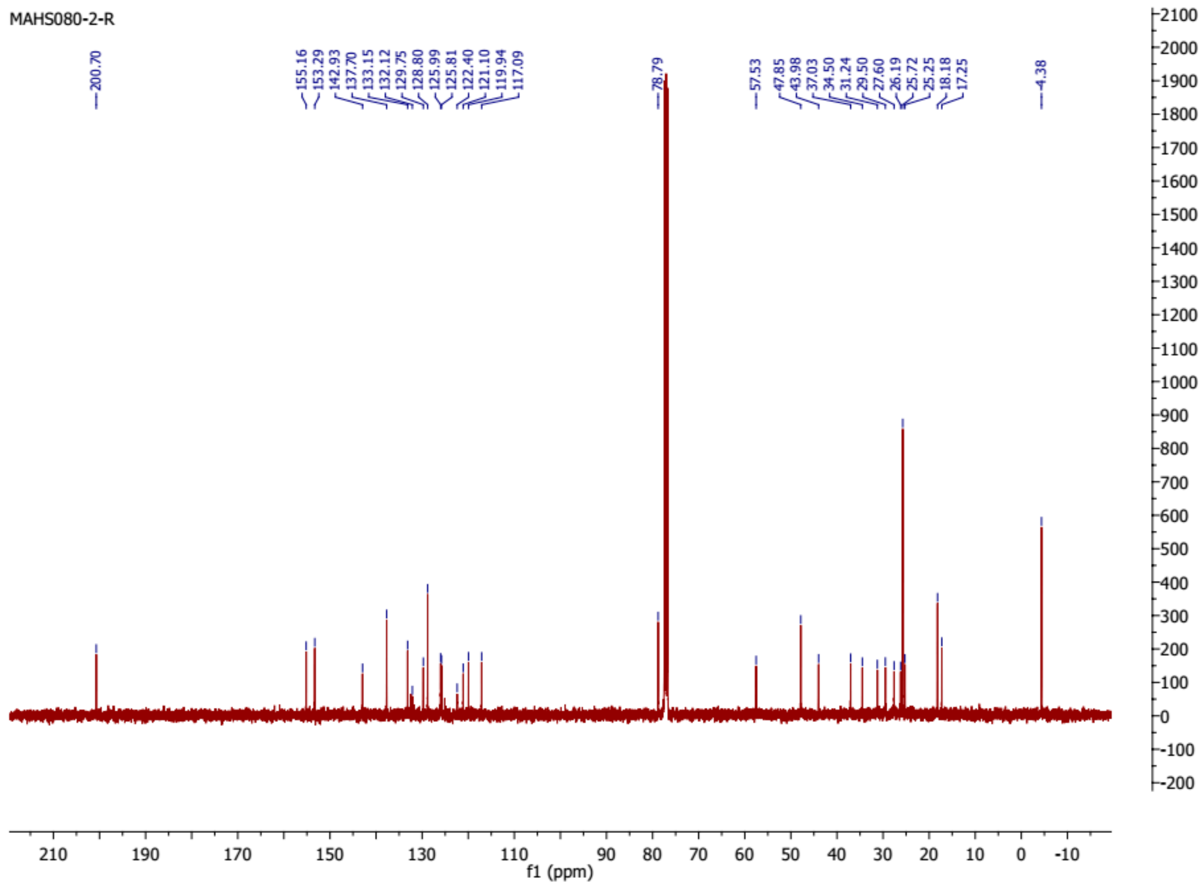

Figure S 31. ^{13}C NMR of compound **15a**.

1.14 Compound 15b

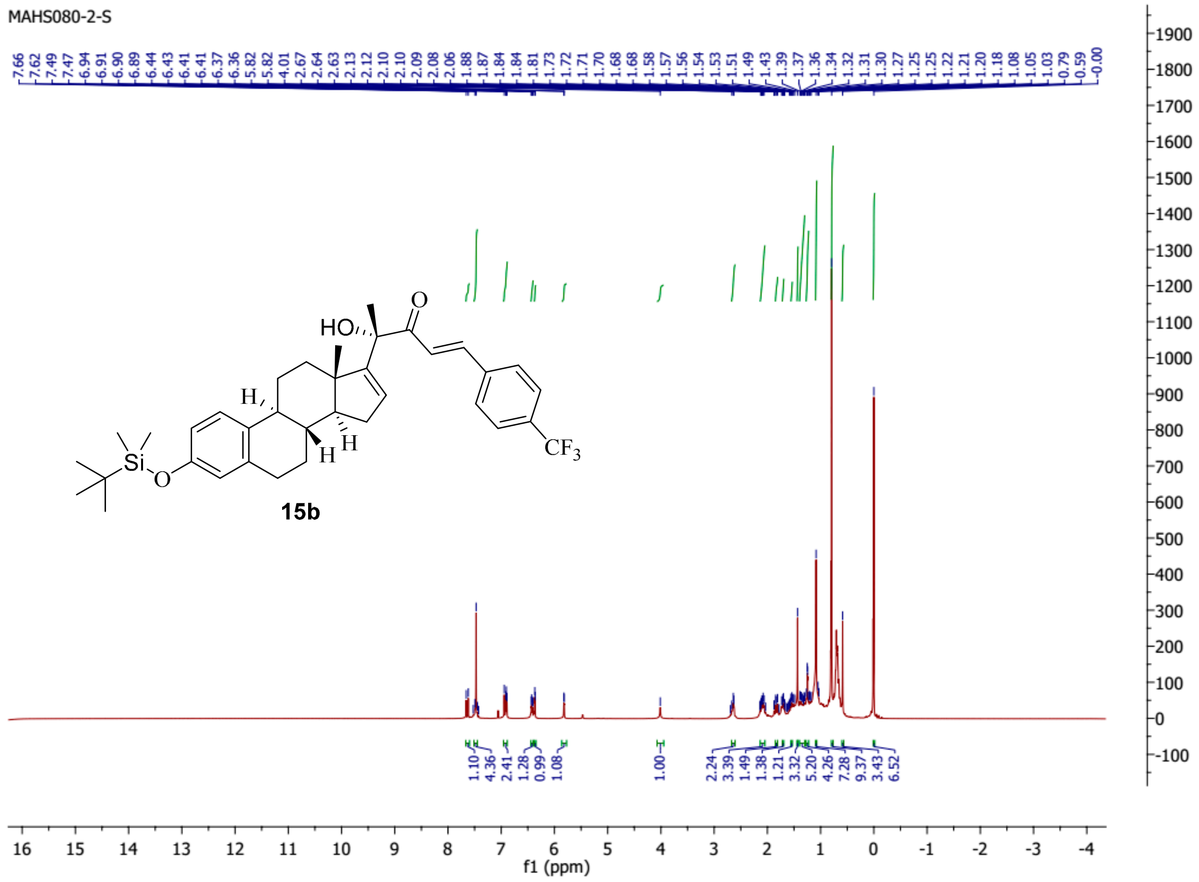

Figure S 32. ^{1}H NMR of compound **15b**.

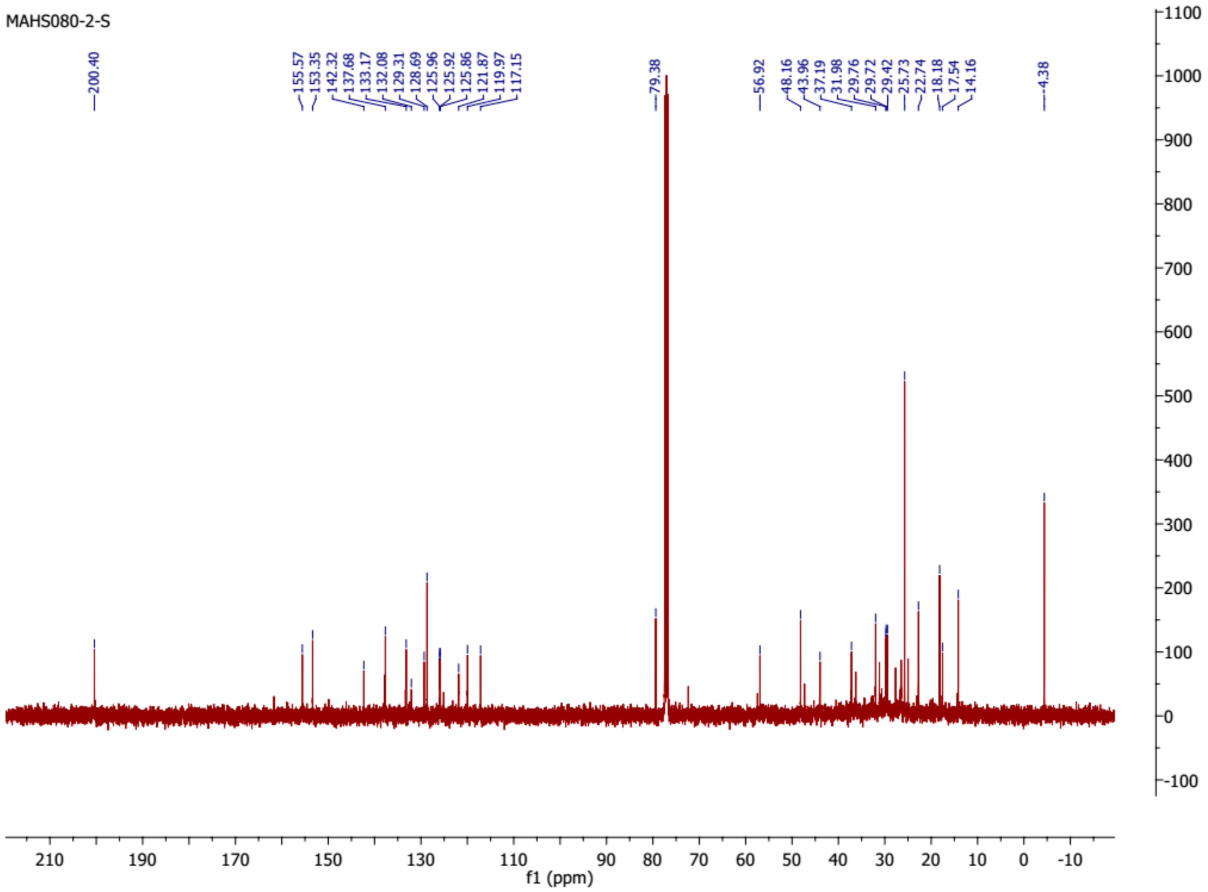

Figure S 33. ^{13}C NMR of compound **15b**.

1.15 Compound 15c

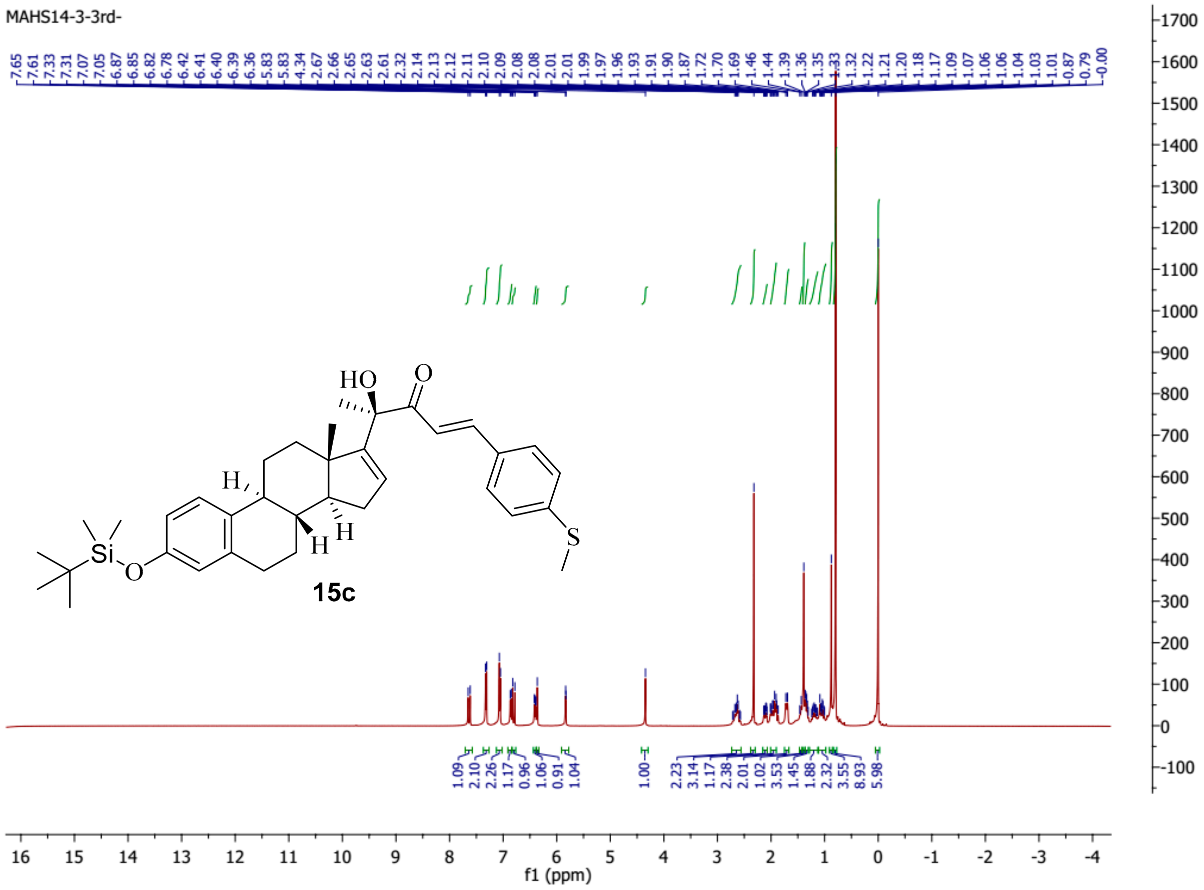

Figure S 34. ^{1}H NMR of compound **15c**.

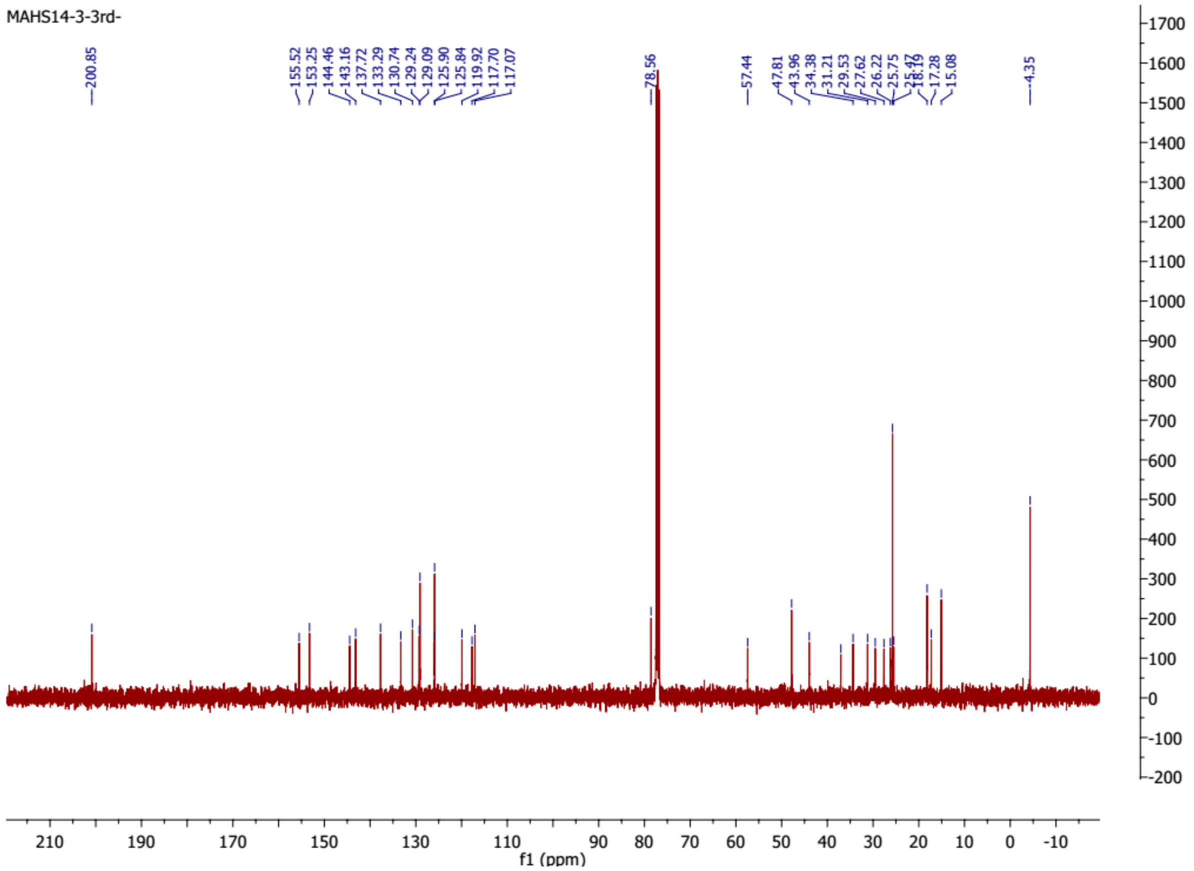

Figure S 35. ^{13}C NMR of compound **15c**.

1.16 *Compound 15d*

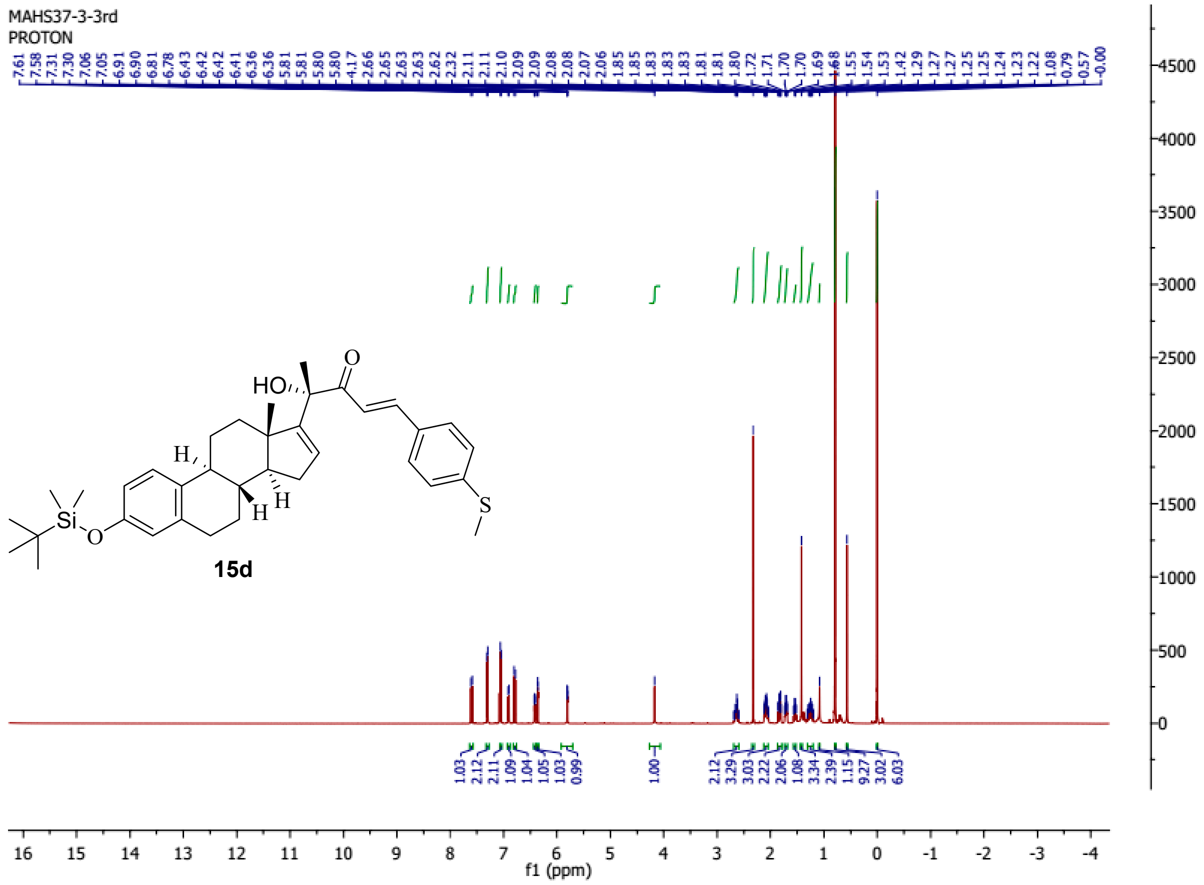

Figure S 36. ^{1}H NMR of compound **15d**.

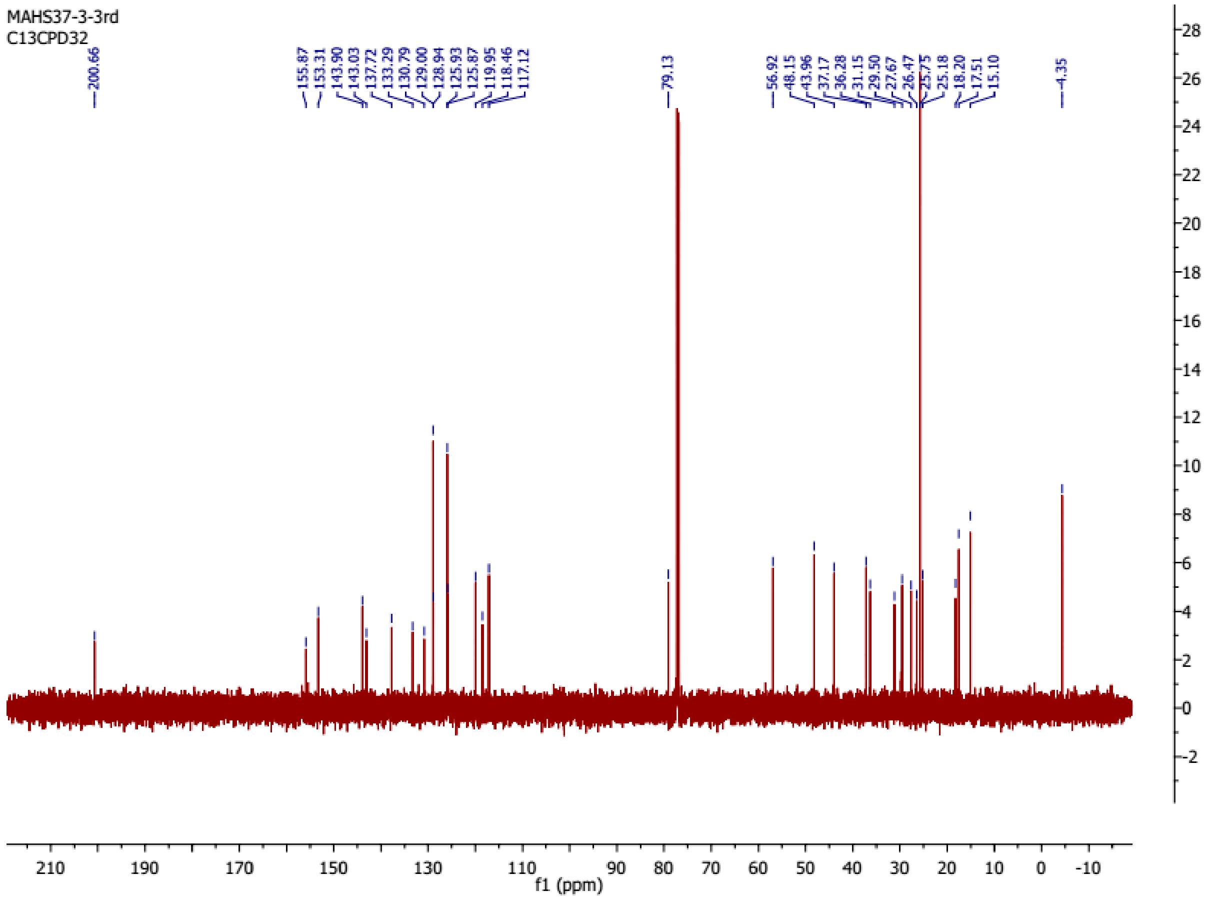

Figure S 37. ^{13}C NMR of compound **15d**.

1.17 *Compound 15e*

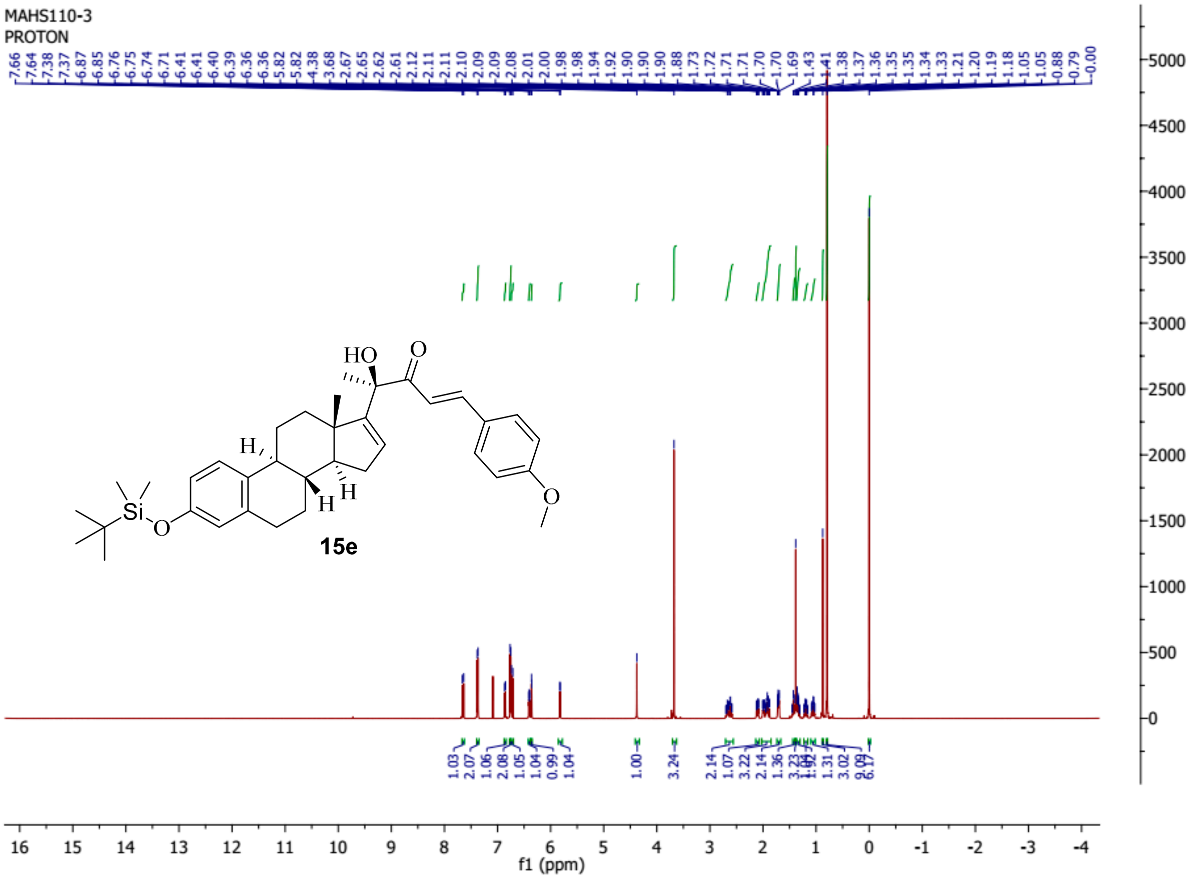

Figure S 38. ^{1}H NMR of compound **15e**.

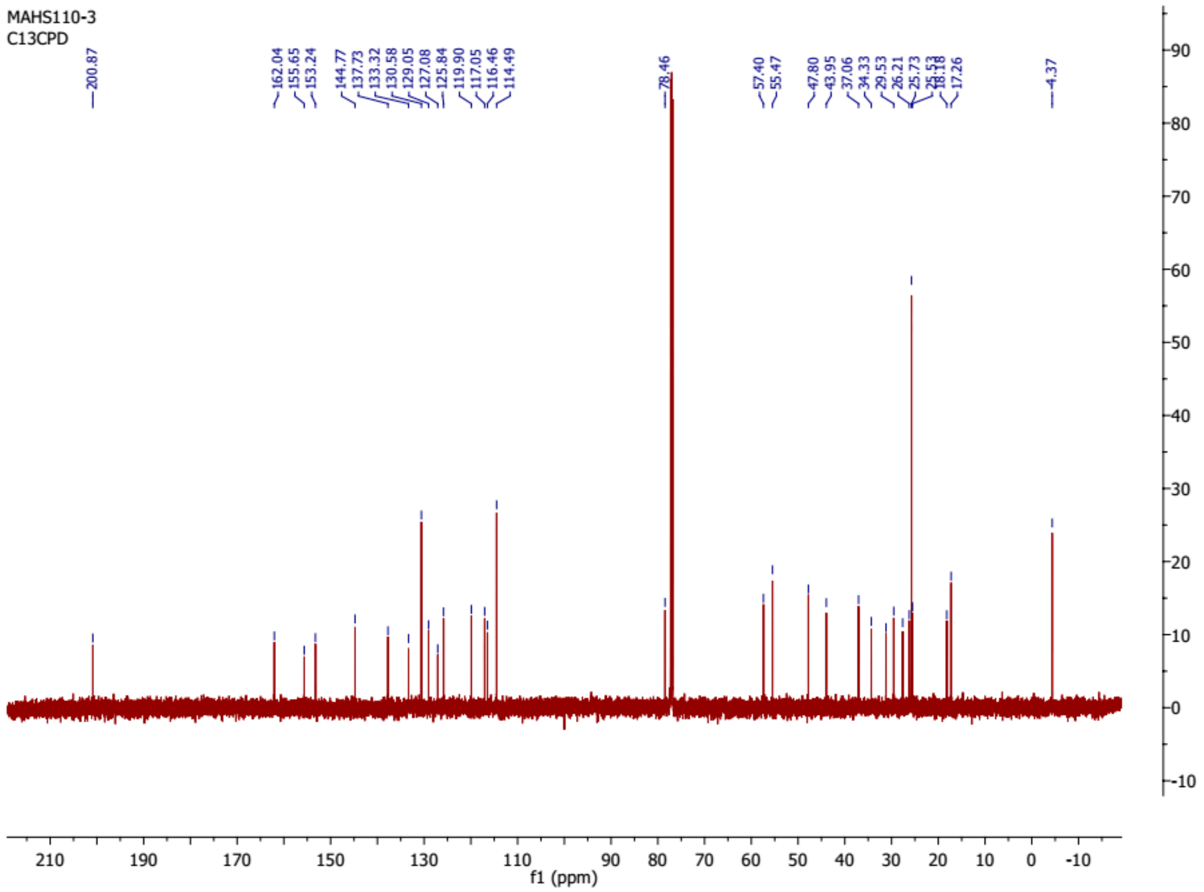

Figure S 39. ^{13}C NMR of compound **15e**.

1.18 Compound 15f

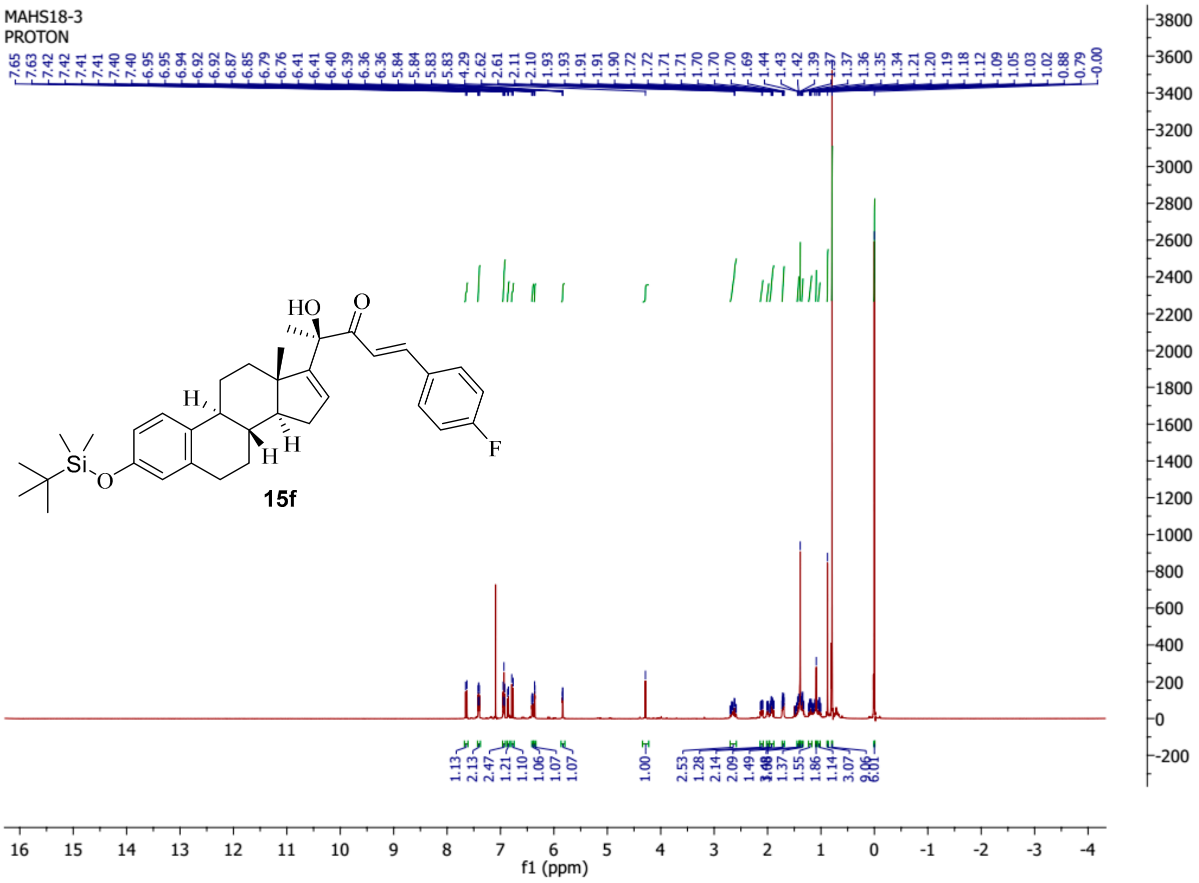

Figure S 40. ^{1}H NMR of compound **15f**.

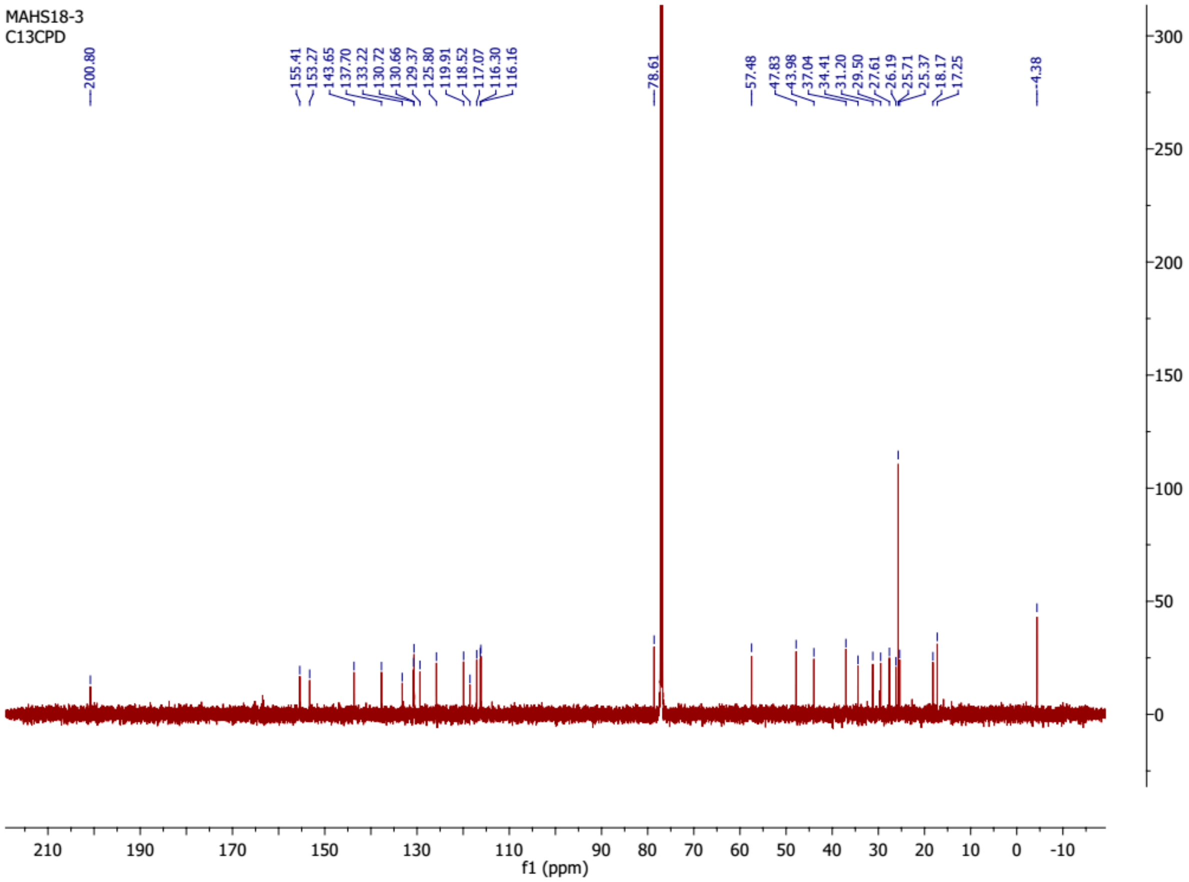

Figure S 41. ^{13}C NMR of compound **15f**.

1.19 Compound 15g

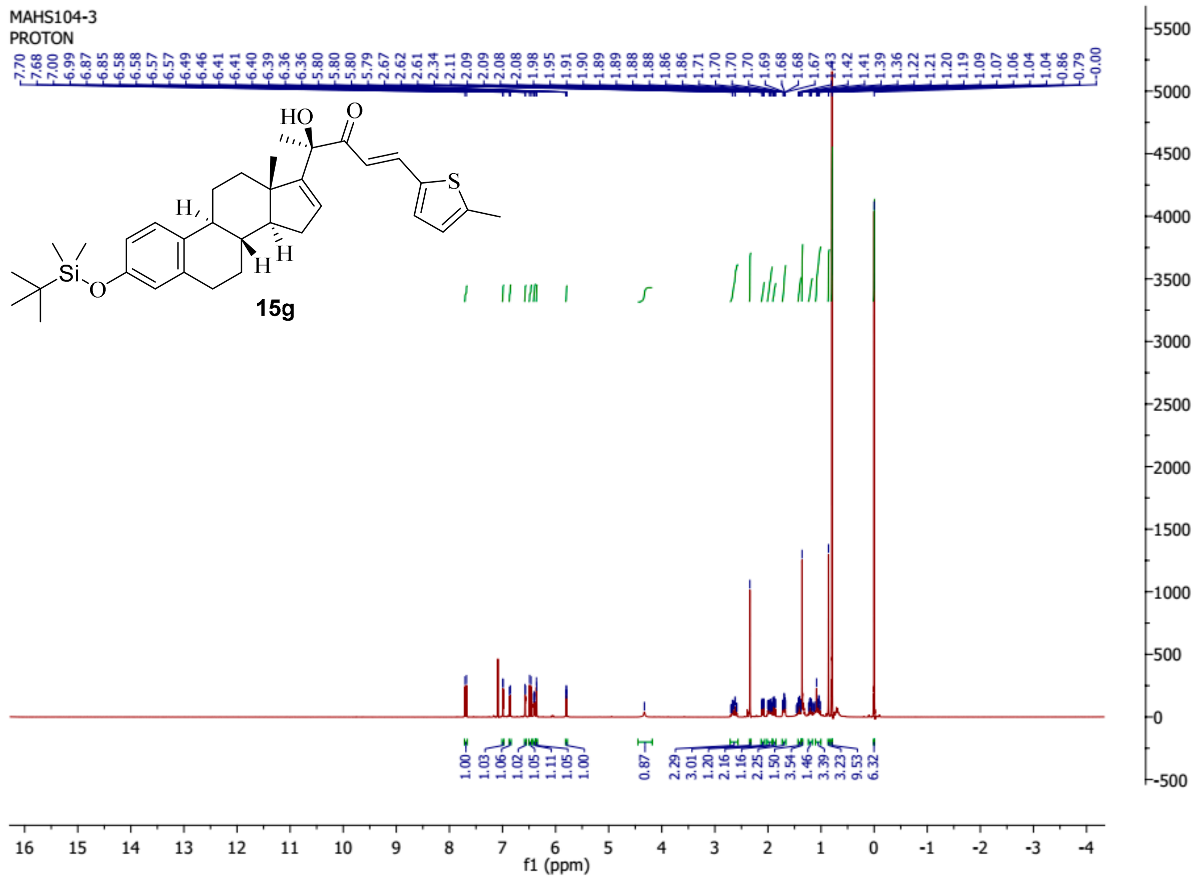

Figure S 42. ^{1}H NMR of compound **15g**.

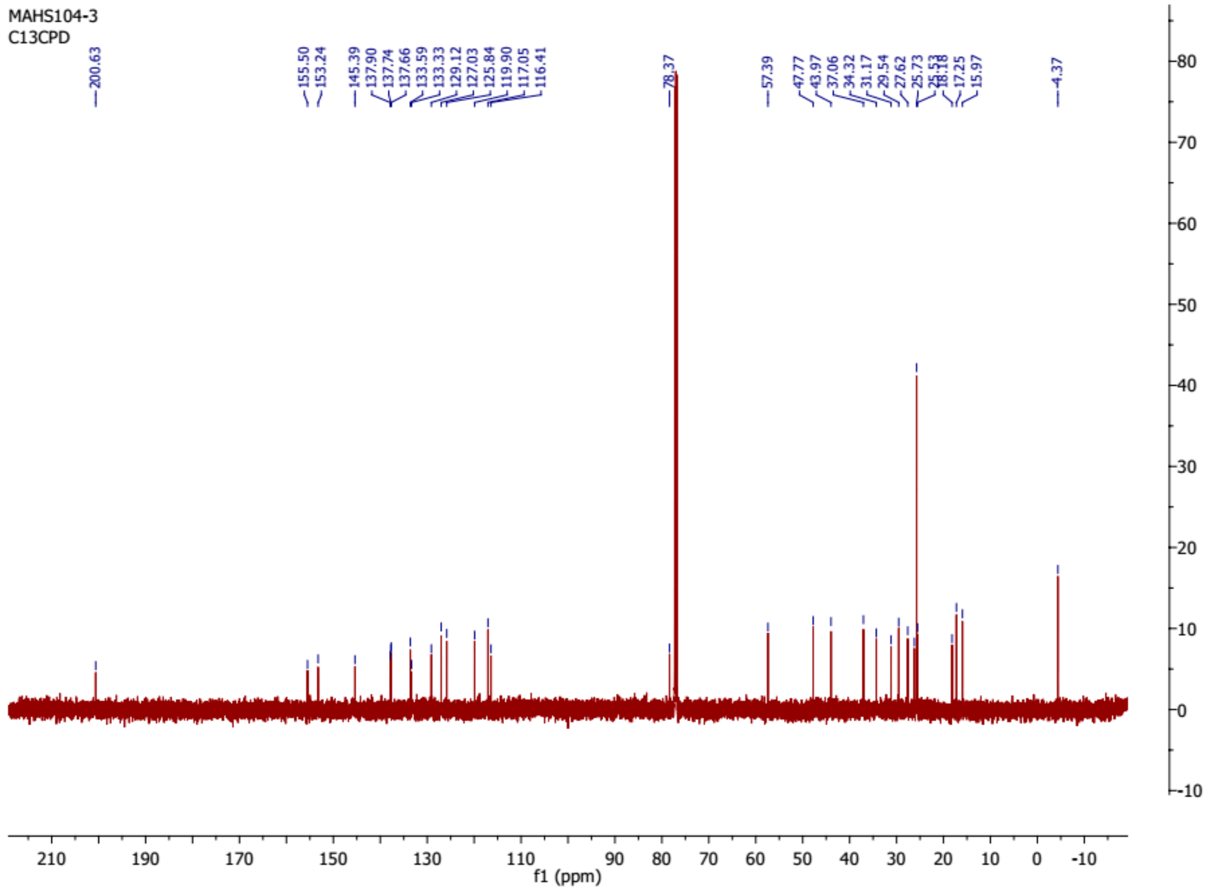

Figure S 43. ^{13}C NMR of compound **15g**.

1.20 Compound 15h

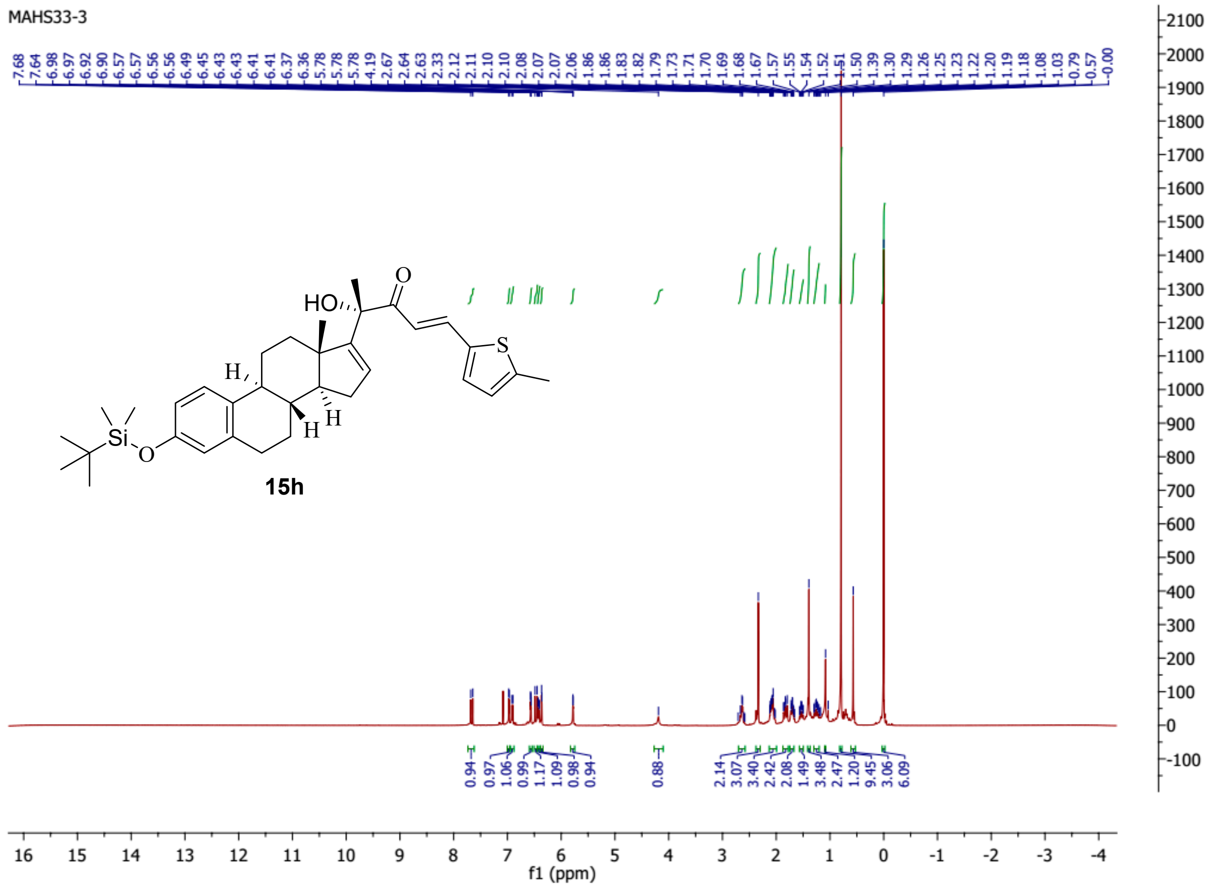

Figure S 44. ^{1}H NMR of compound **15h**.

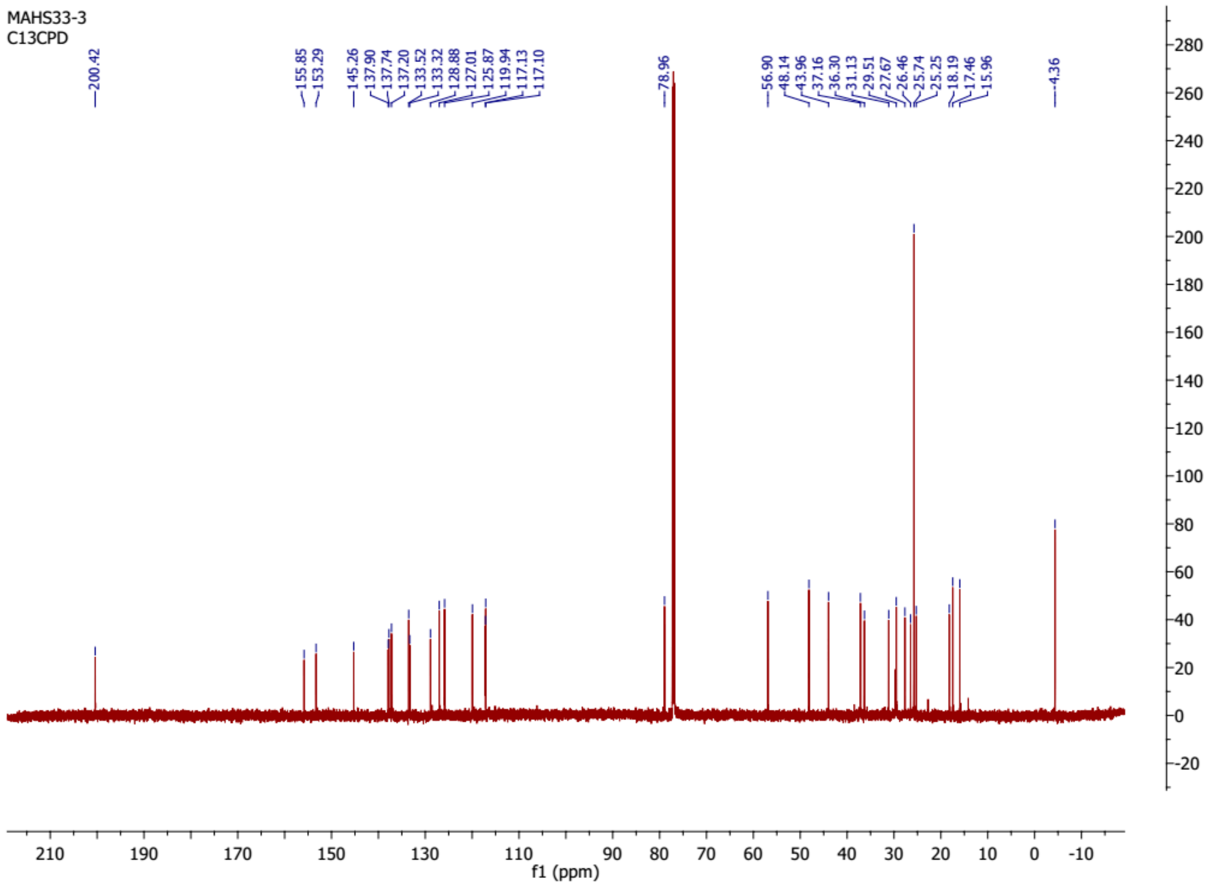

Figure S 45. ^{13}C NMR of compound **15h**.

1.21 *Compound 15i*

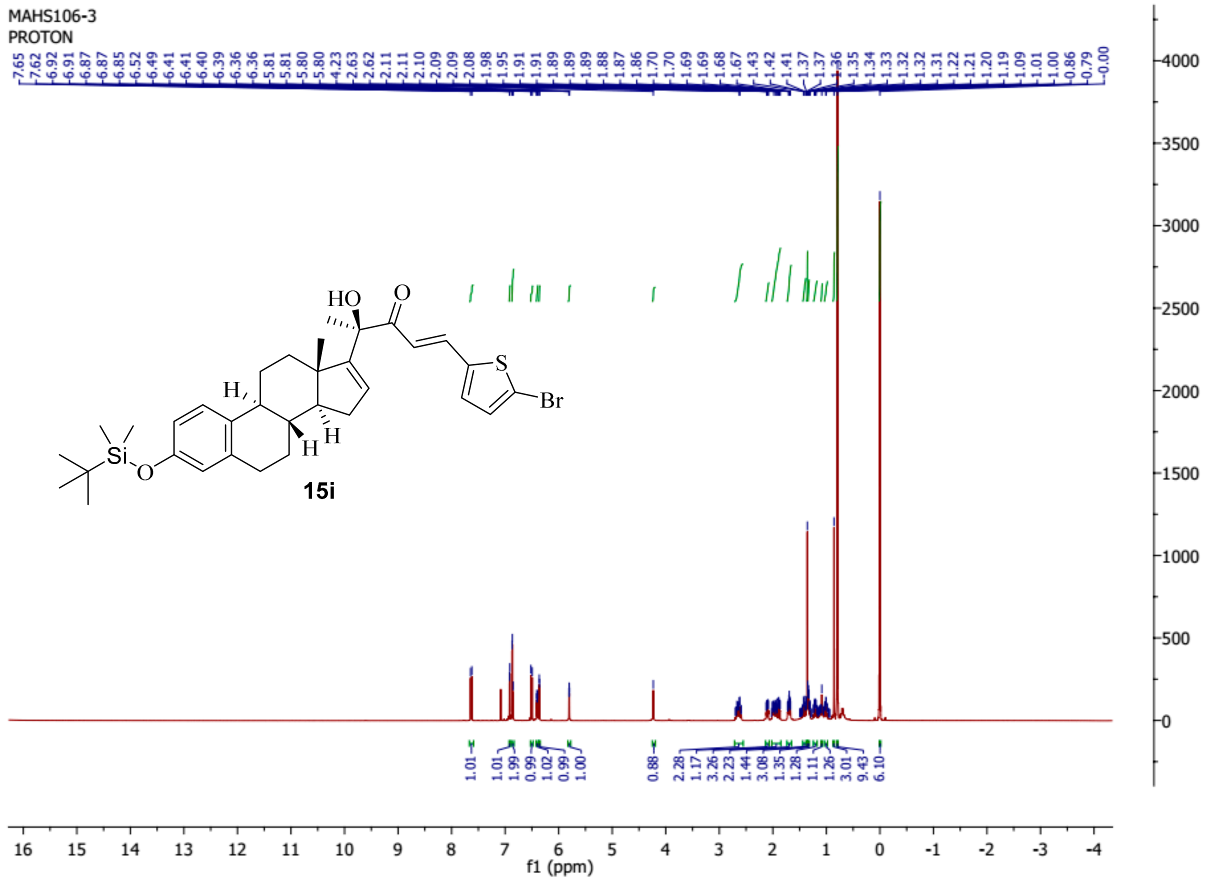

Figure S 46. ^{1}H NMR of compound **15i**.

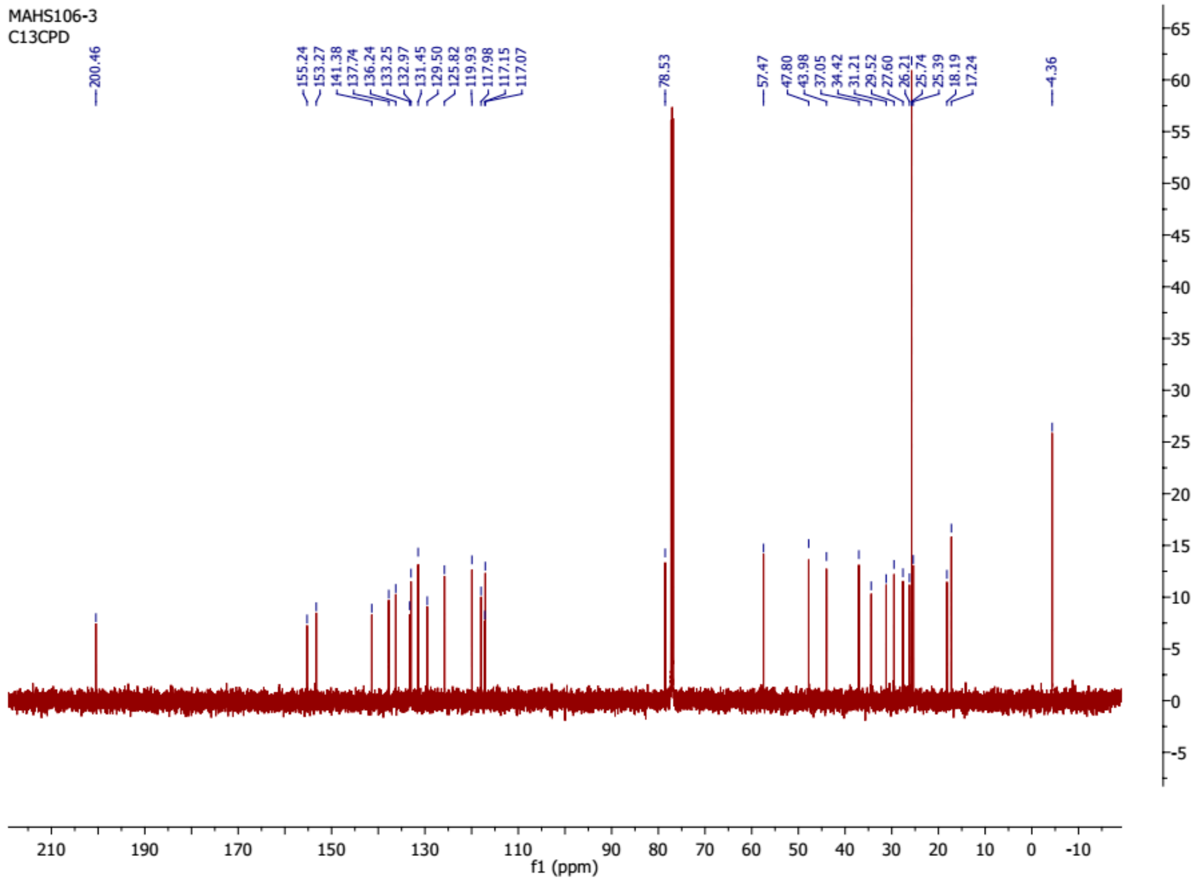

Figure S 47. ^{13}C NMR of compound **15i**.

1.22 Compound 15j

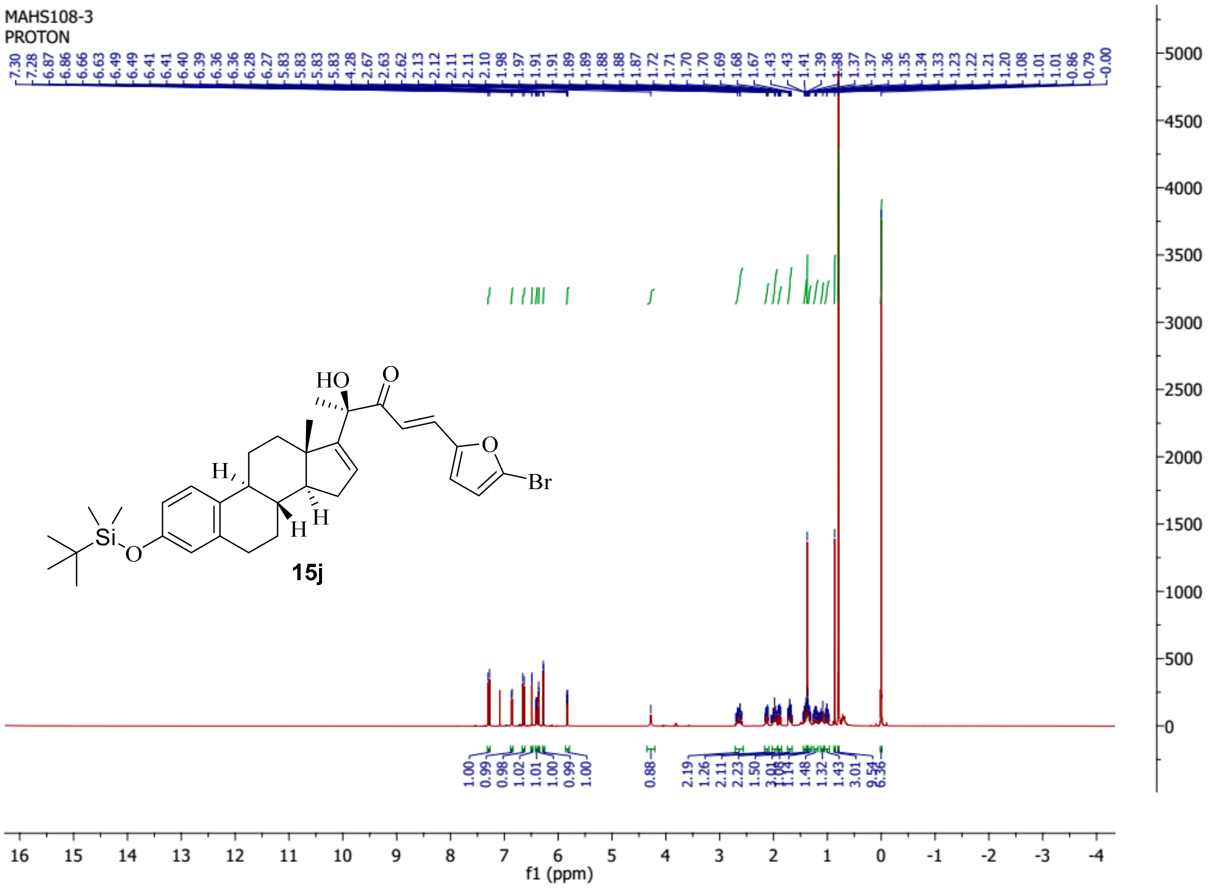

Figure S 48. ^{1}H NMR of compound **15j**.

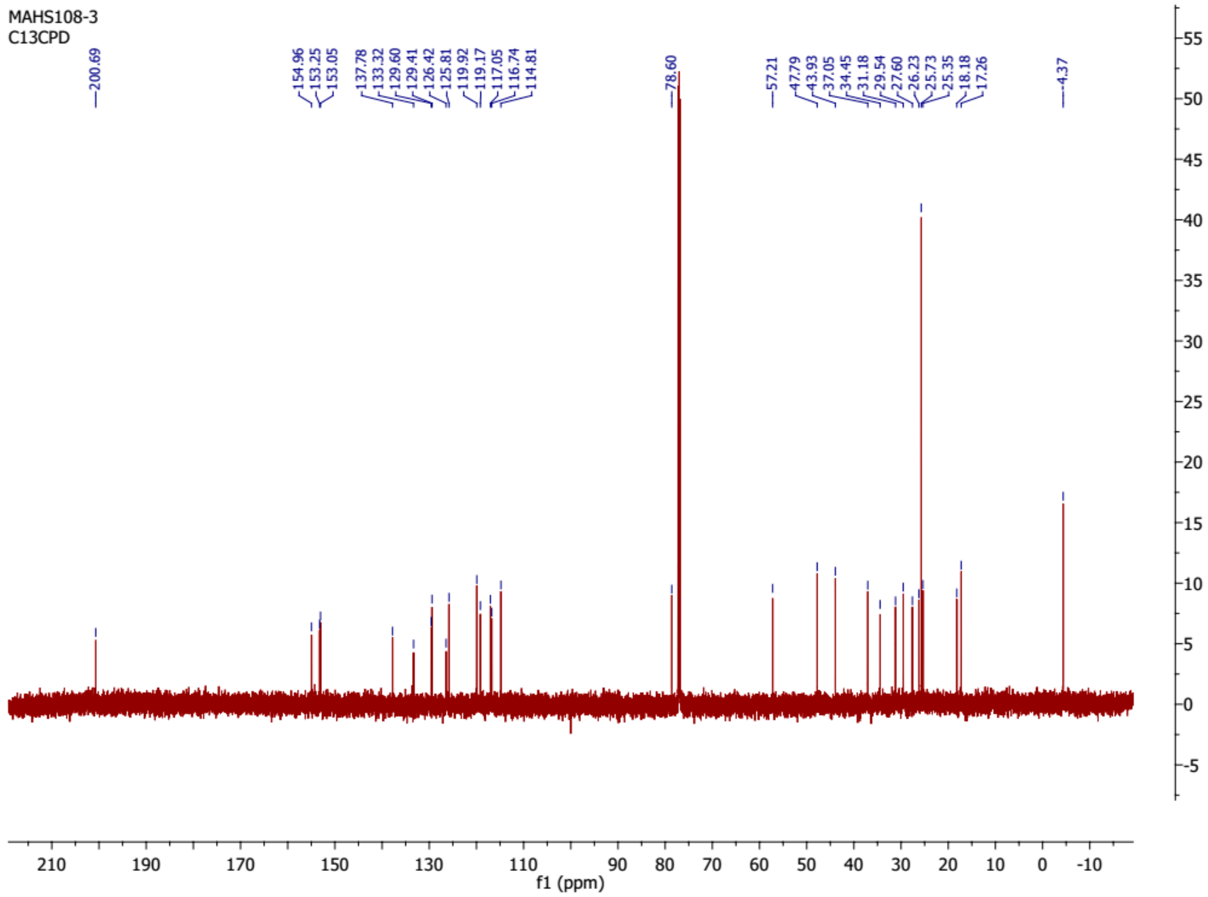

Figure S 49. ^{13}C NMR of compound **15j**.

1.23 Compound 16a

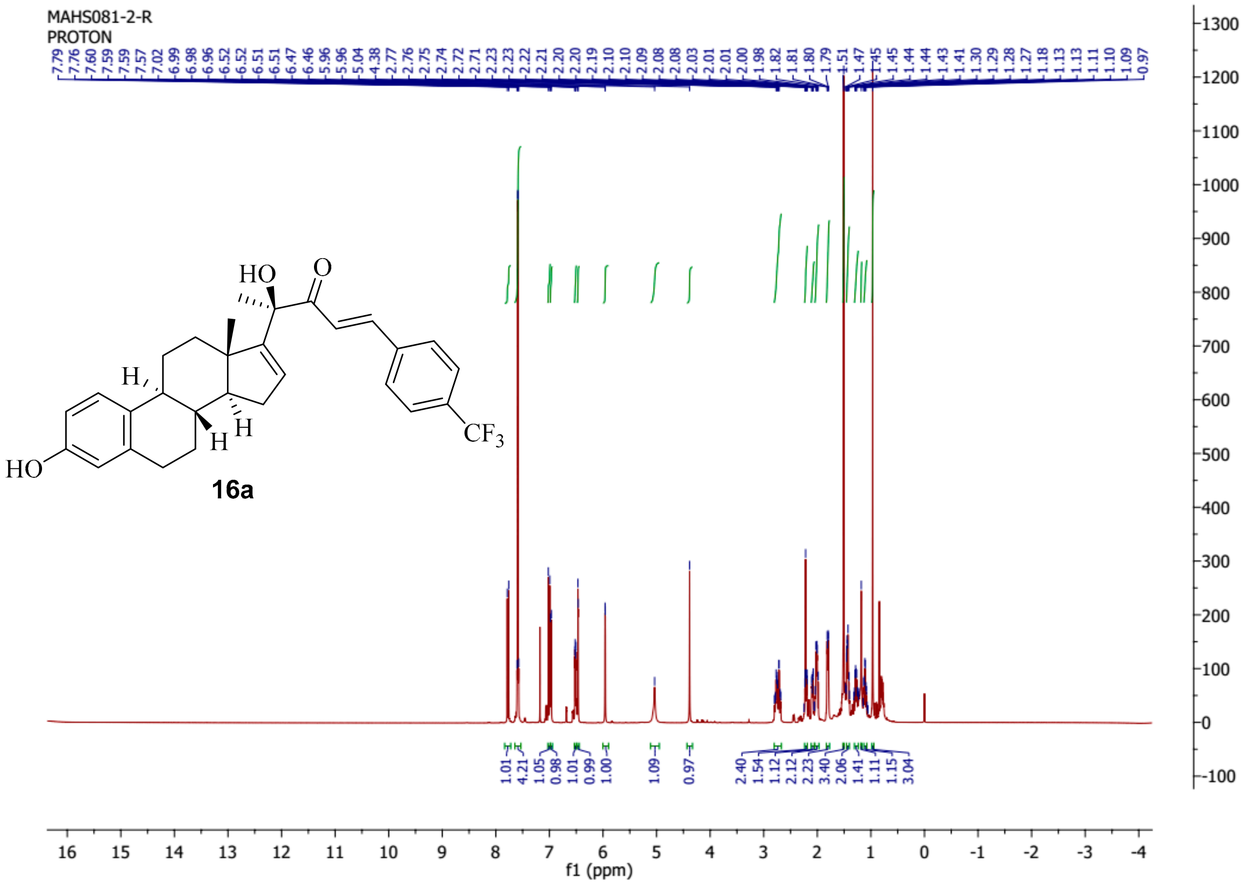

Figure S 50. ^{1}H NMR of compound **16a**.

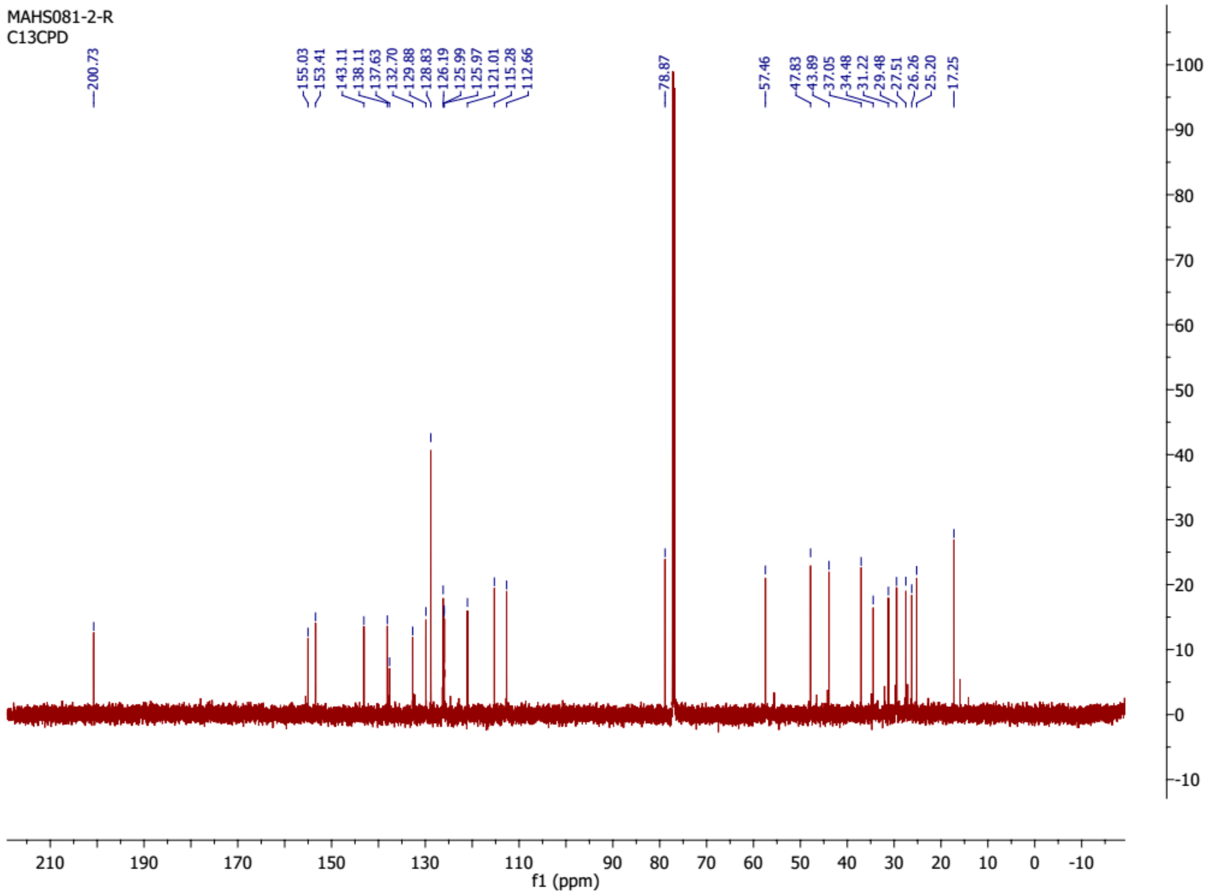

Figure S 51. ^{13}C NMR of compound **16a**.

1.24 Compound 16b

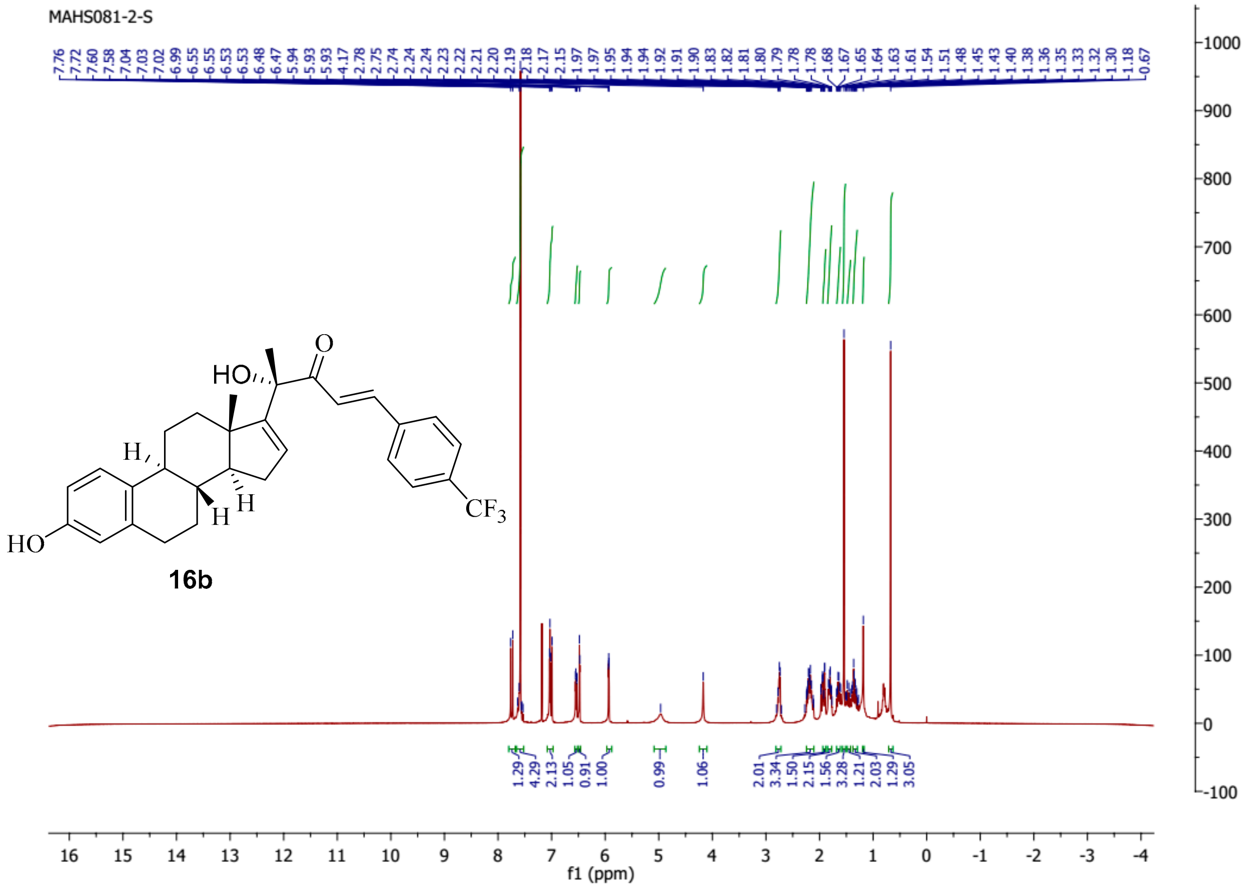

Figure S 52. ^{1}H NMR of compound **16b**.

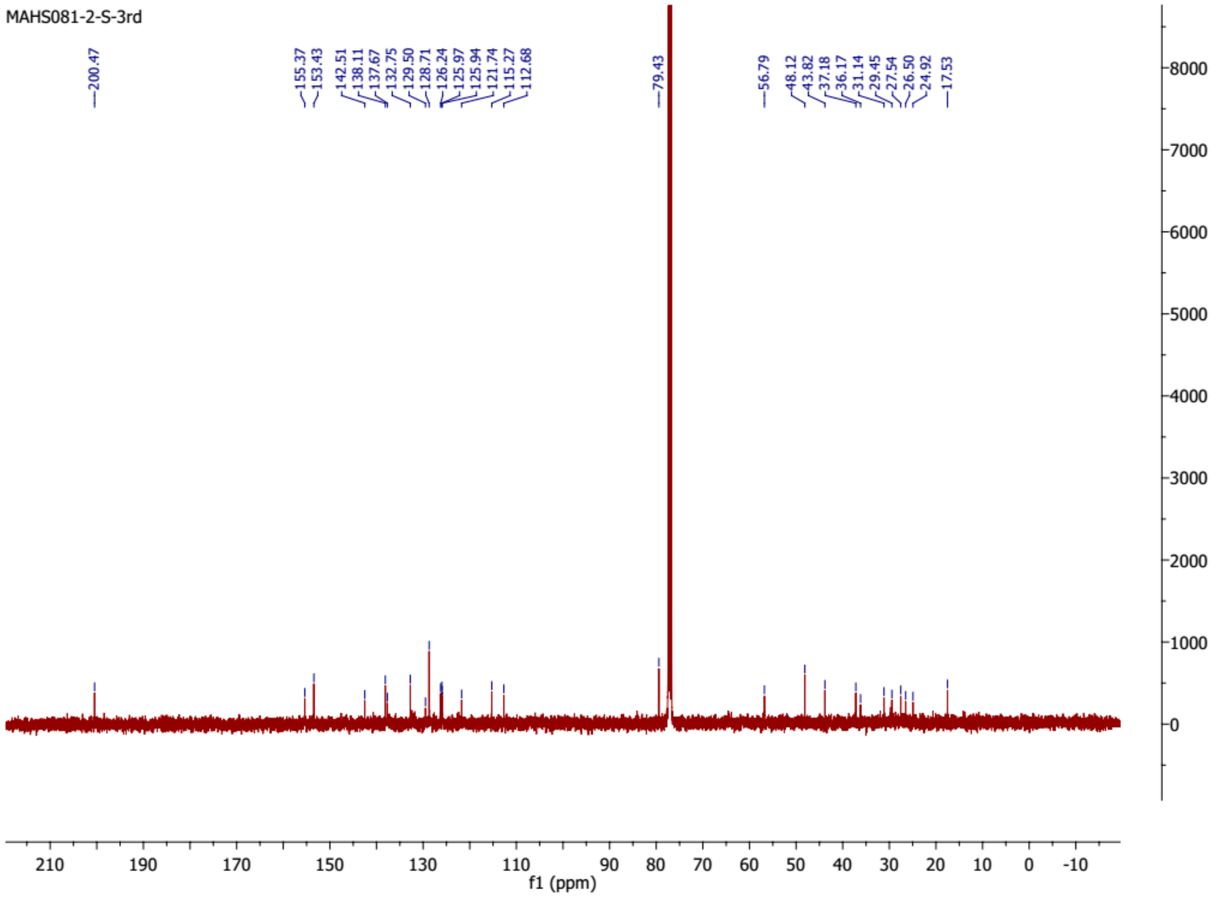

Figure S 53. ^{13}C NMR of compound **16b**.

1.25 Compound 16c

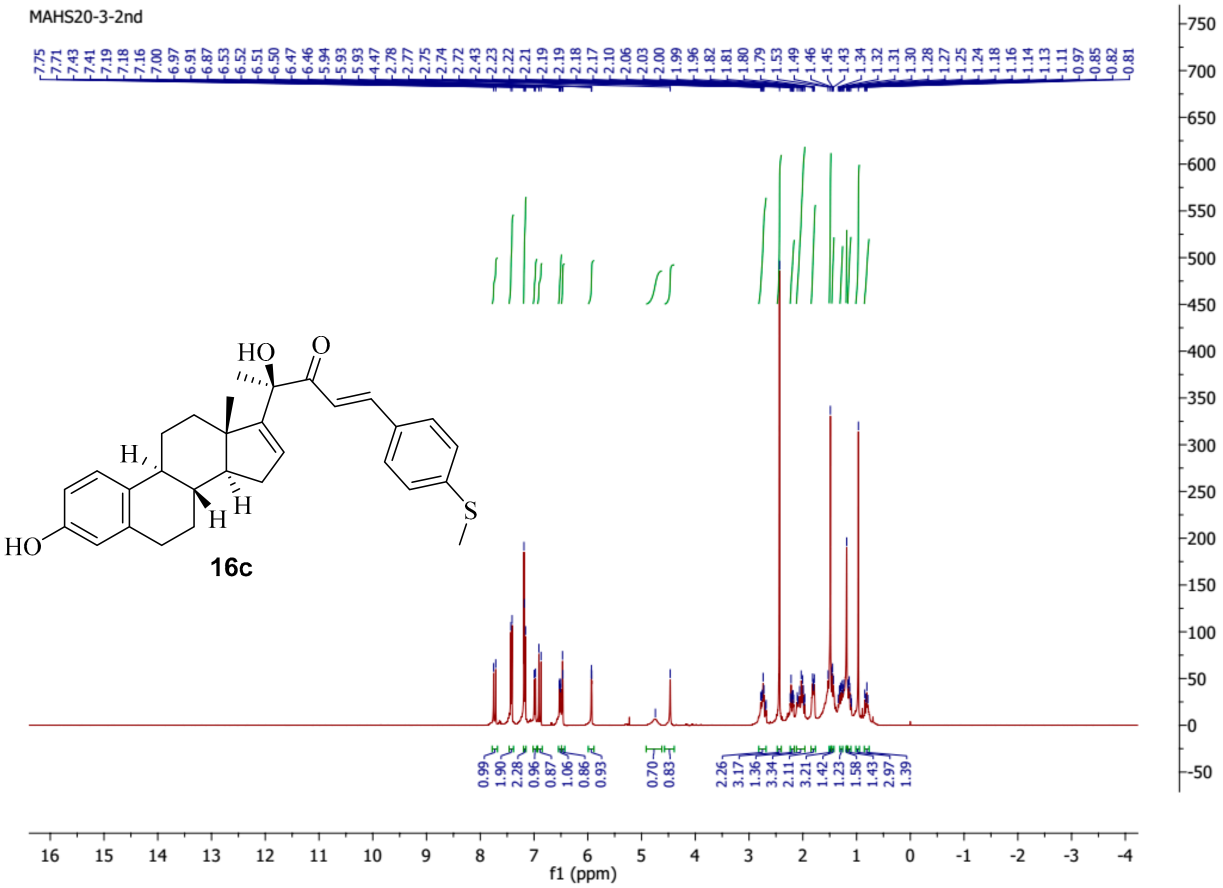

Figure S 54. ^{1}H NMR of compound **16c**.

1.26 Compound 16d

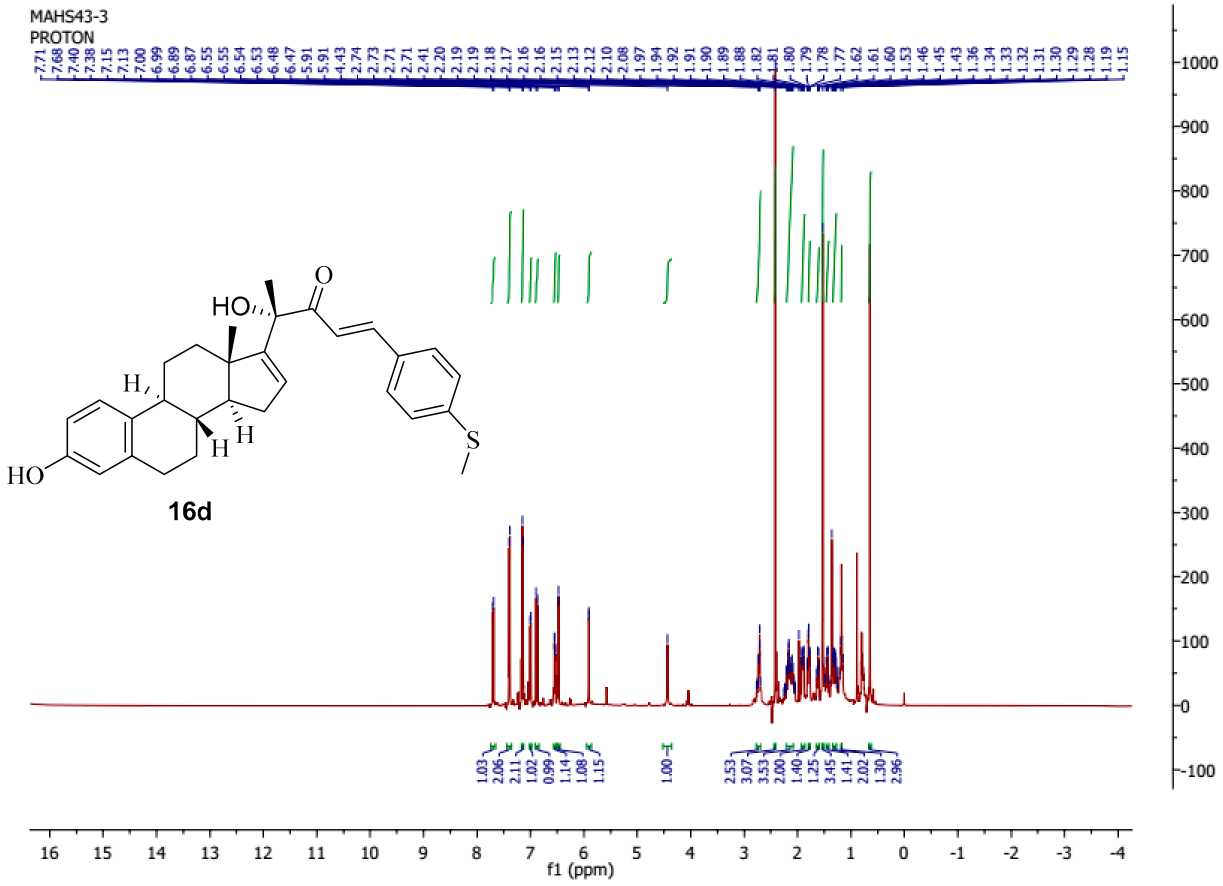

Figure S 55. ^{1}H NMR of compound **16d**.

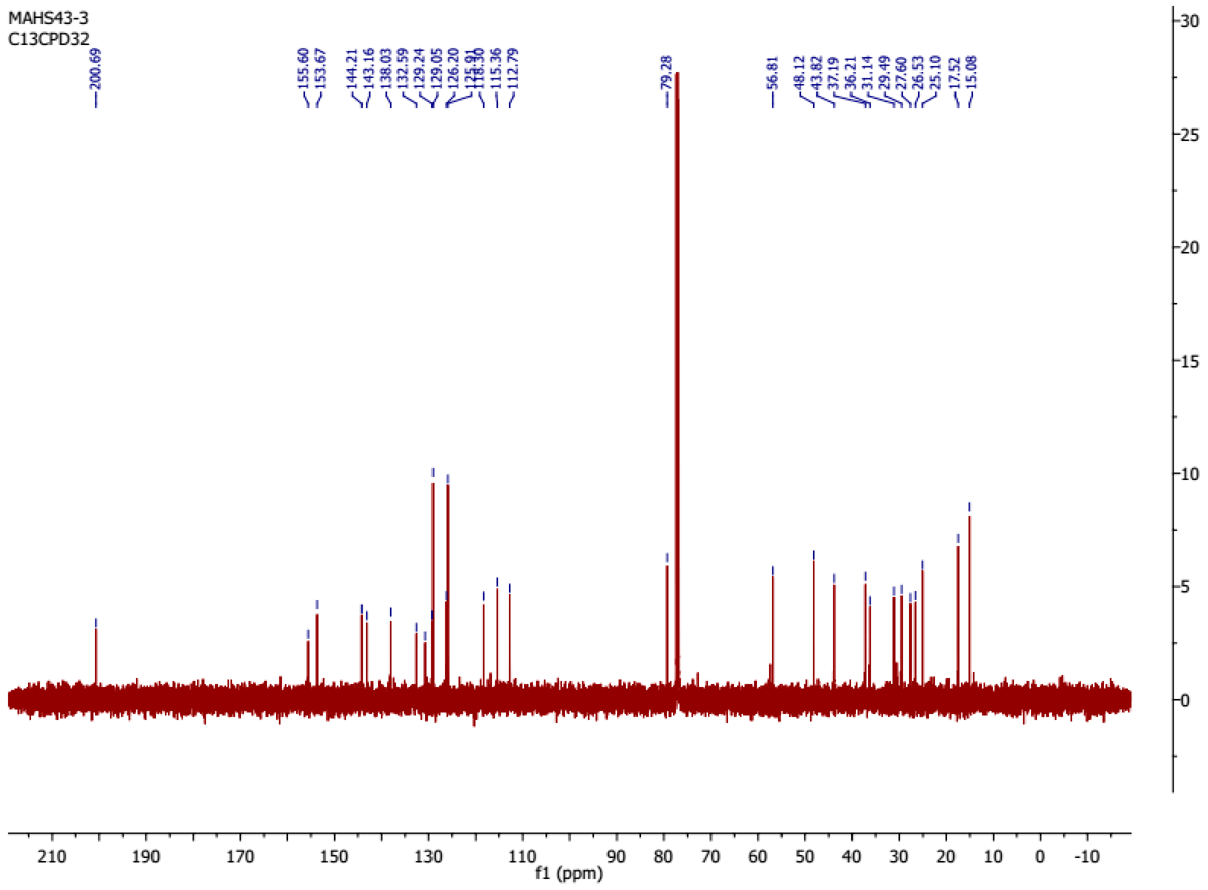

Figure S 56. ^{13}C NMR of compound **16d**.

1.27 *Compound 16e*

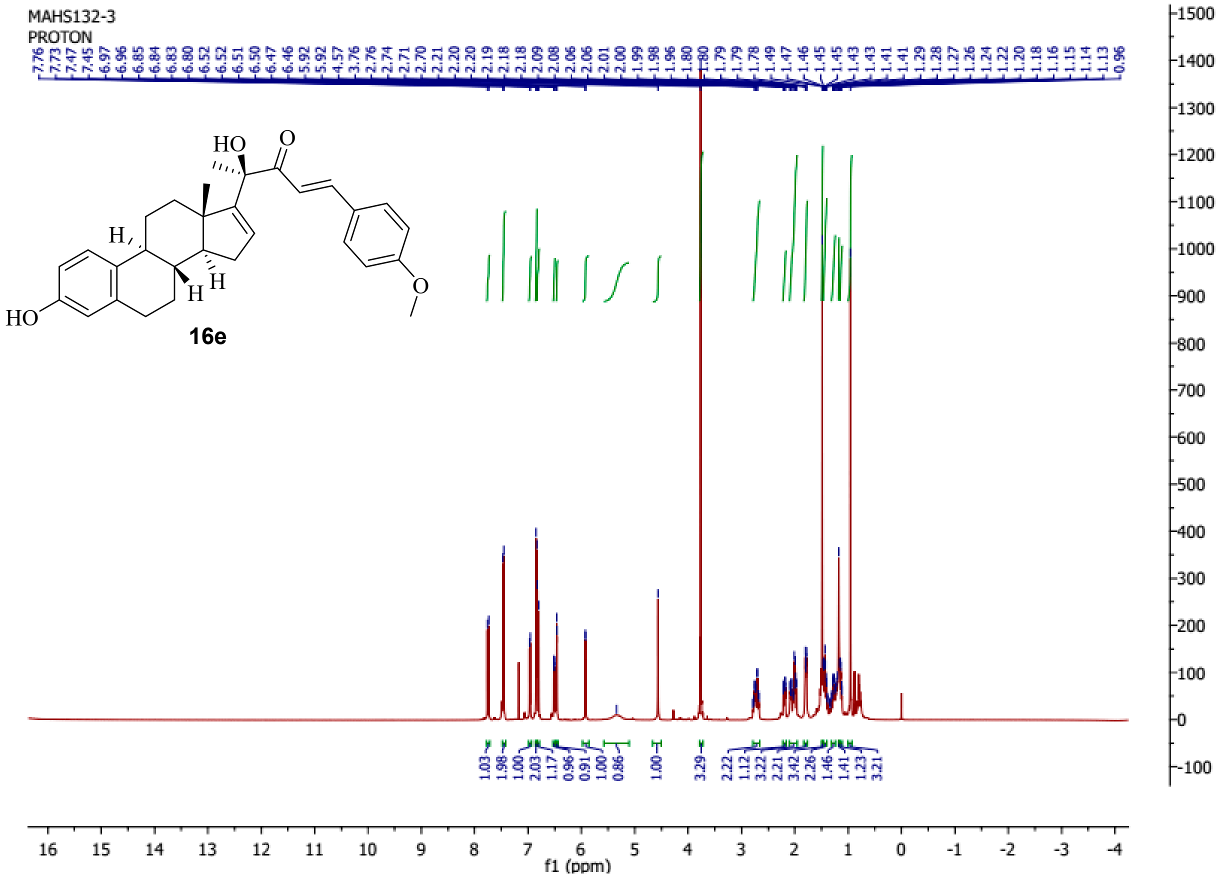

Figure S 57. ^{1}H NMR of compound **16e**.

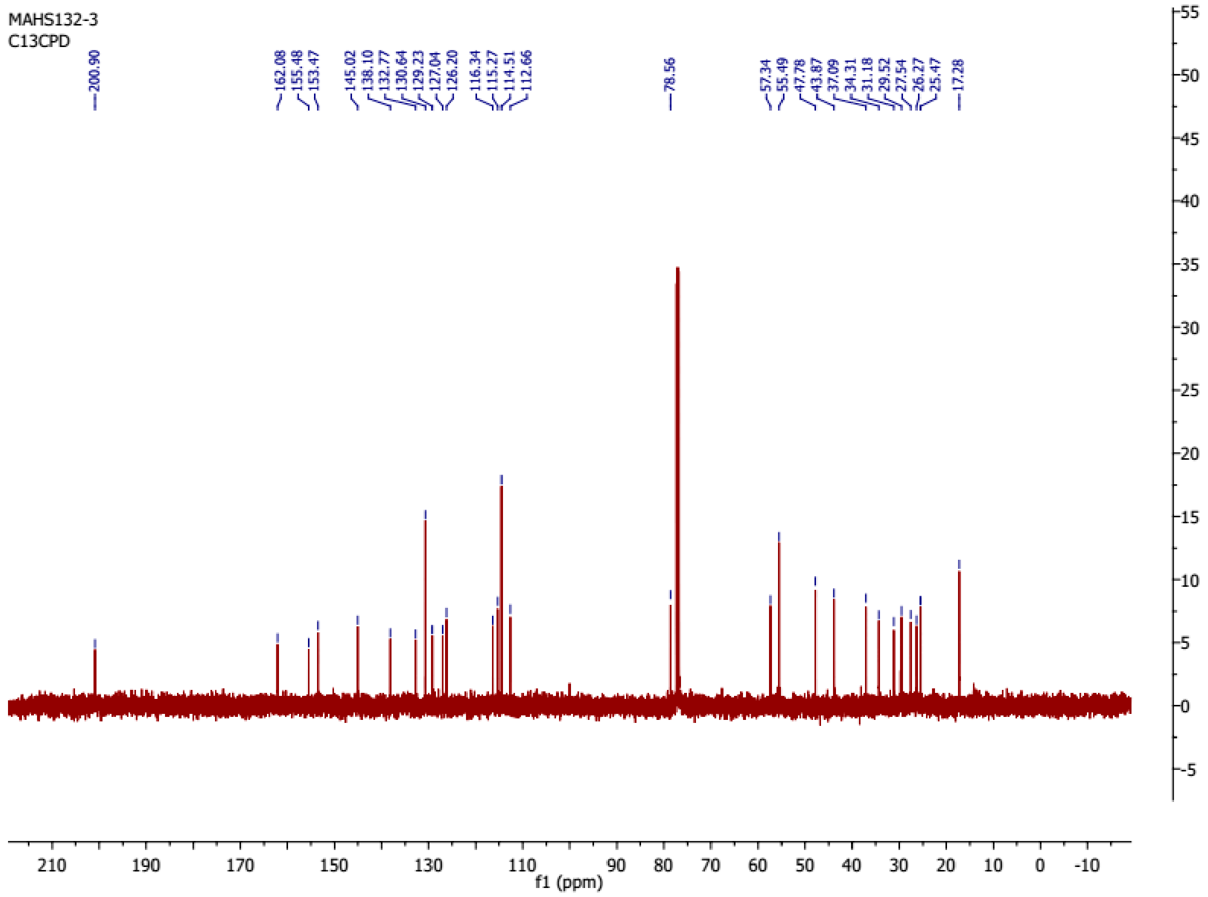

Figure S 58. ^{13}C NMR of compound **16e**.

1.28 Compound 16f

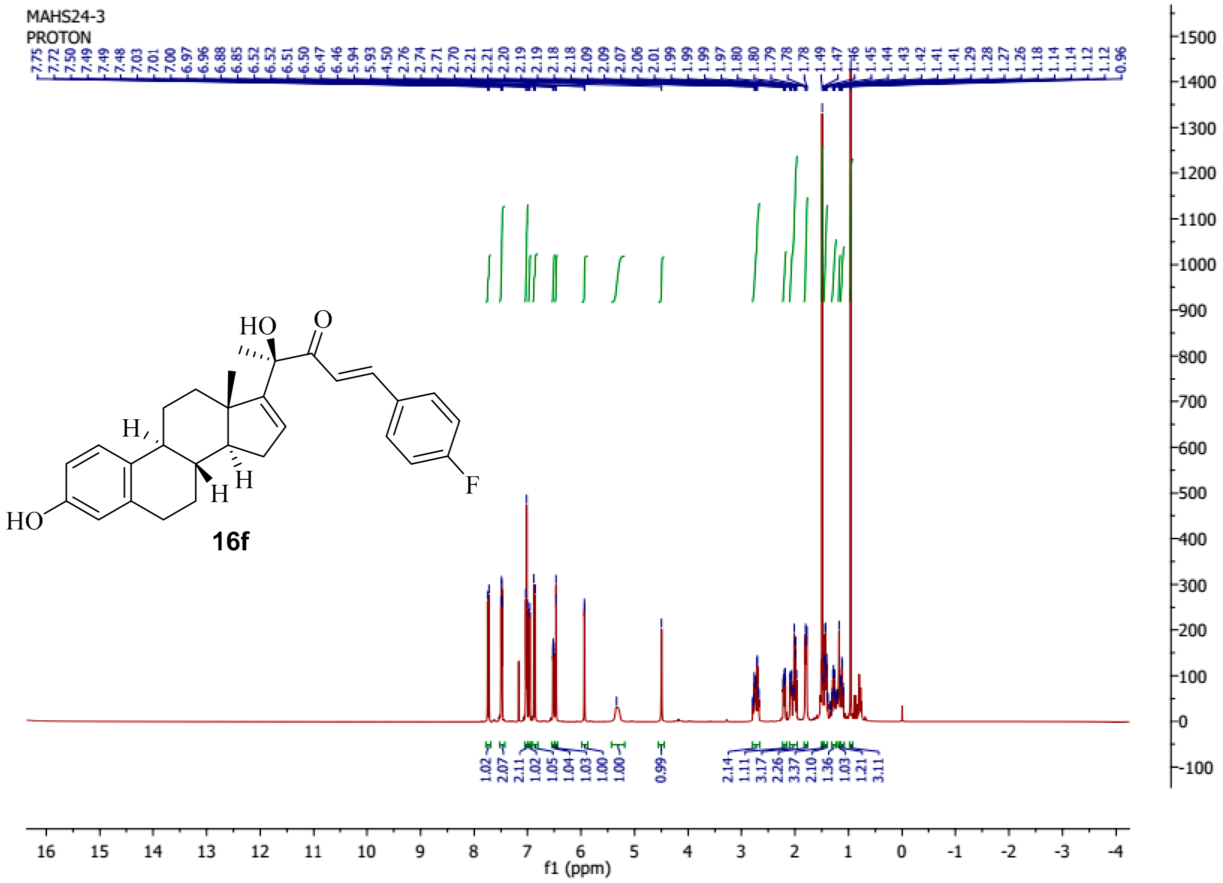

Figure S 59. ^{1}H NMR of compound **16f**.

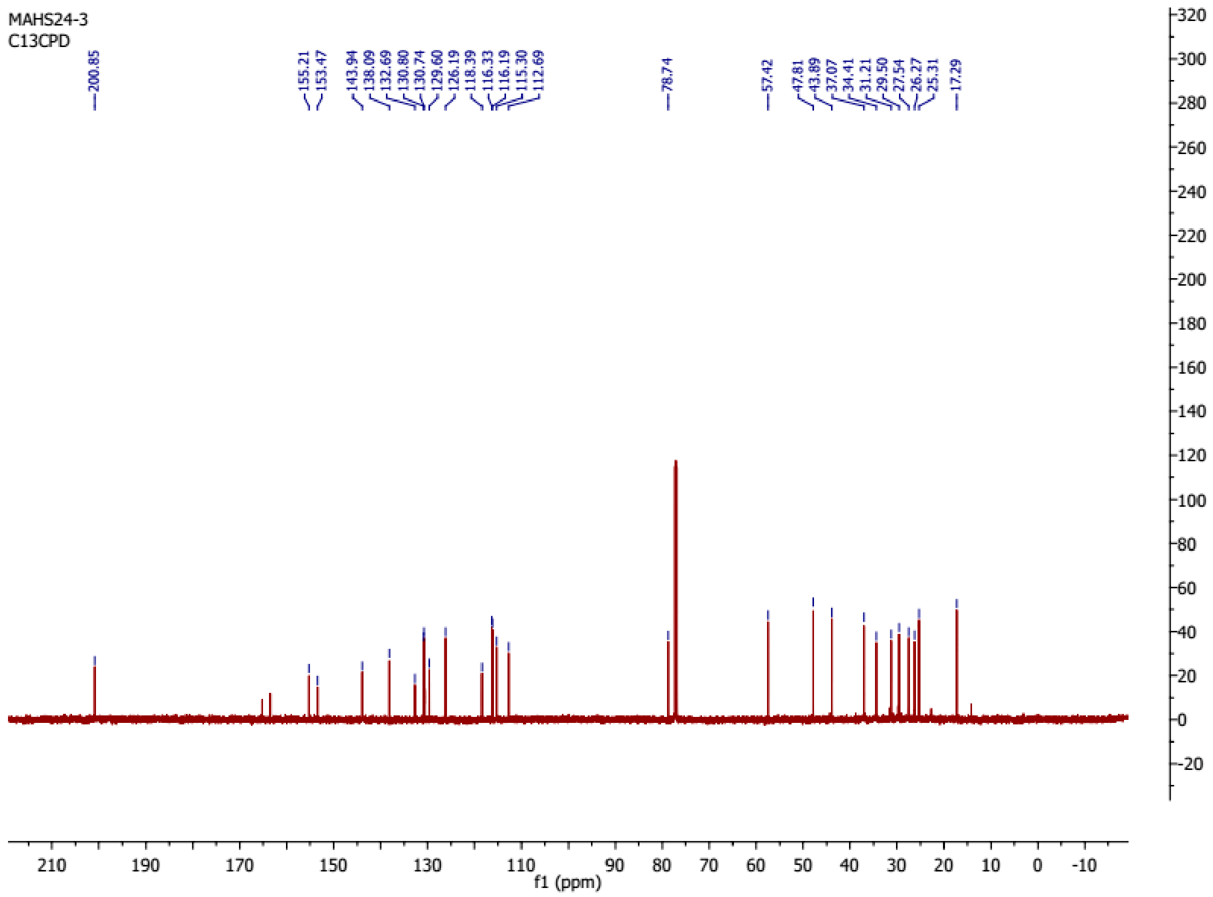

Figure S 60. ^{13}C NMR of compound **16f**.

1.29 Compound 16g

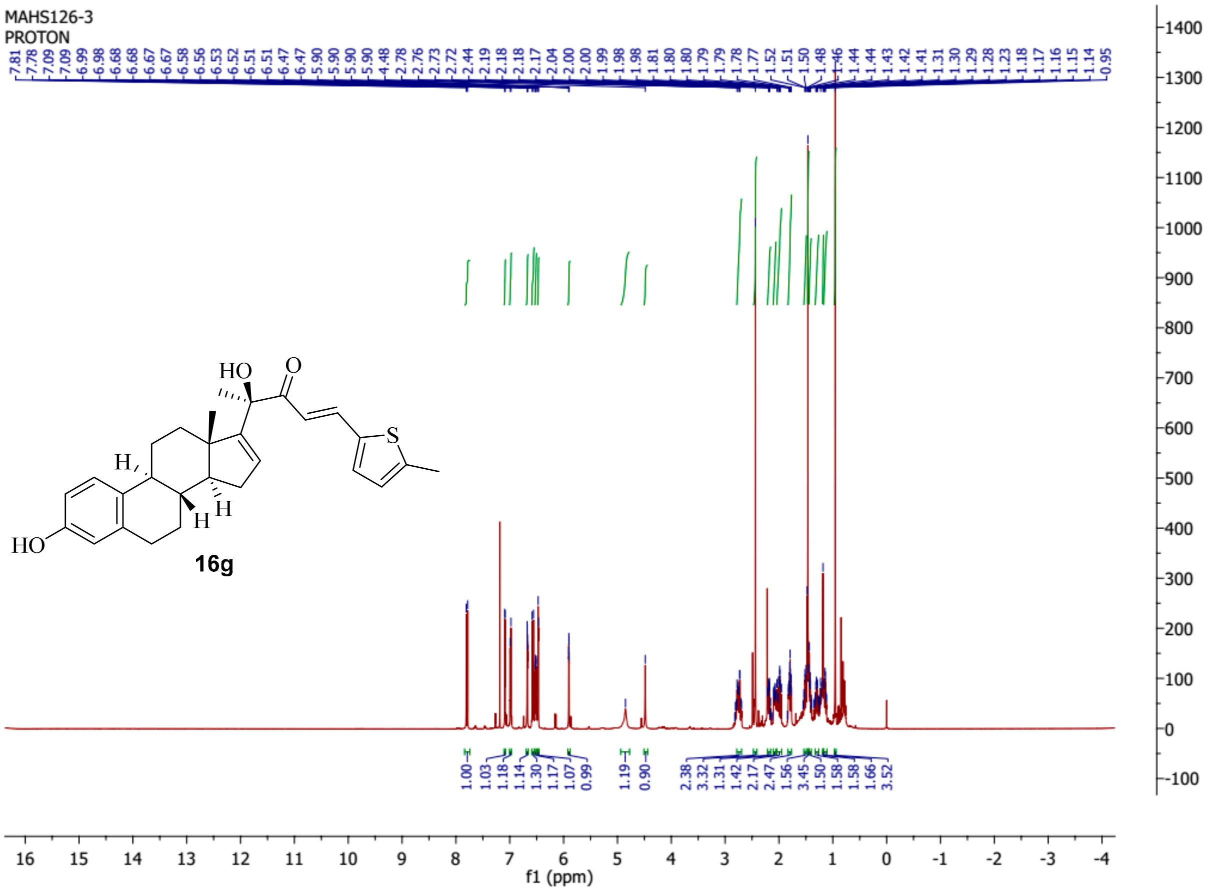

Figure S 61. ^{1}H NMR of compound **16g**.

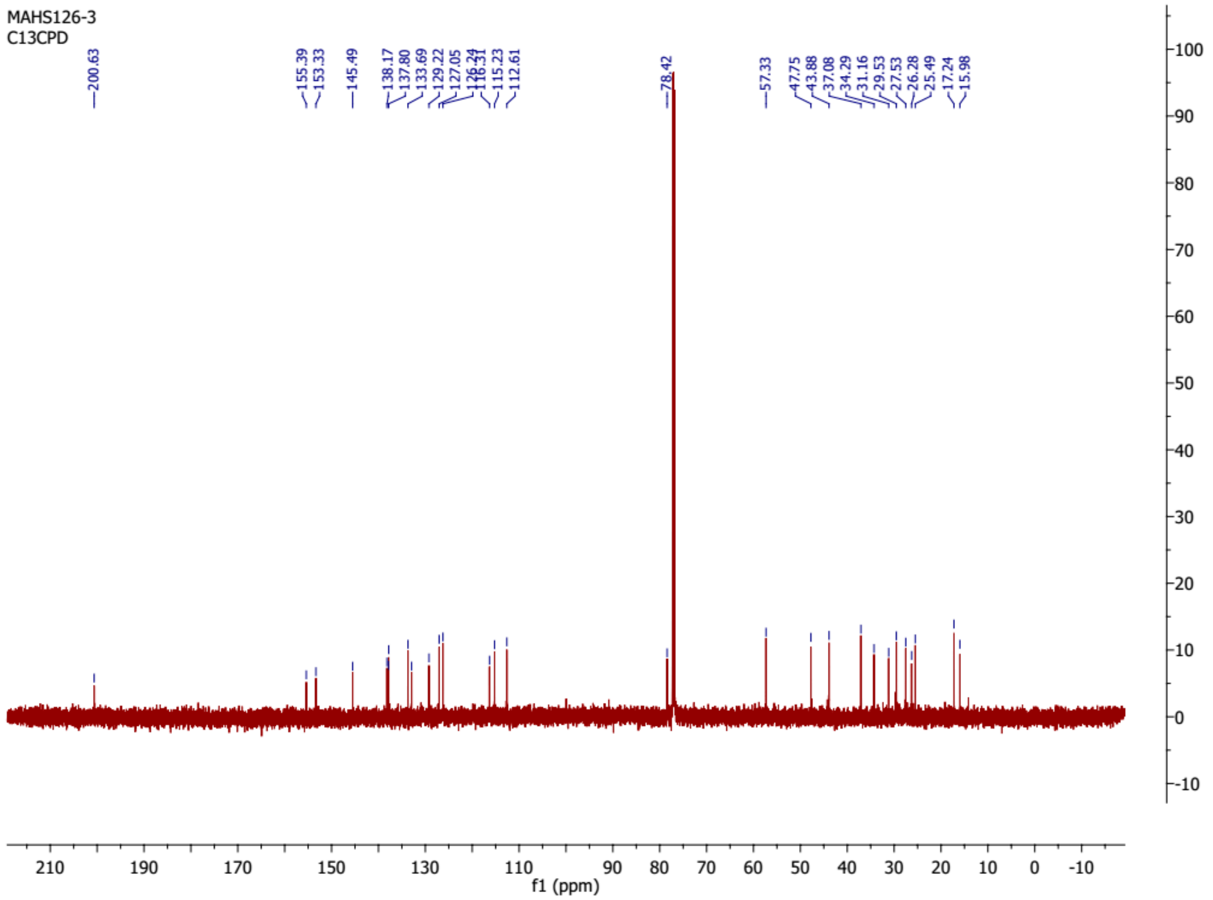

Figure S 62. ^{13}C NMR of compound **16g**.

1.30 Compound 16h

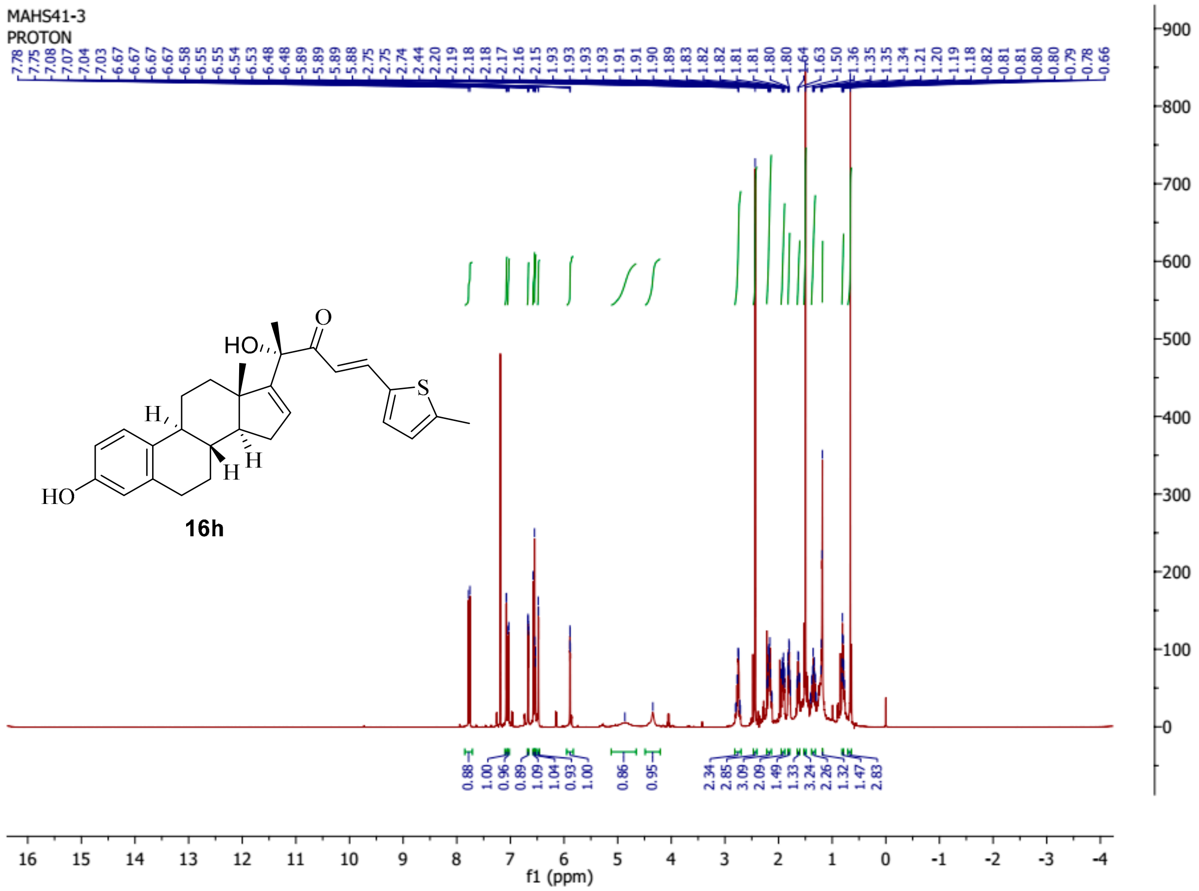

Figure S 63. ^{1}H NMR of compound **16h**.

1.31 Compound 16i

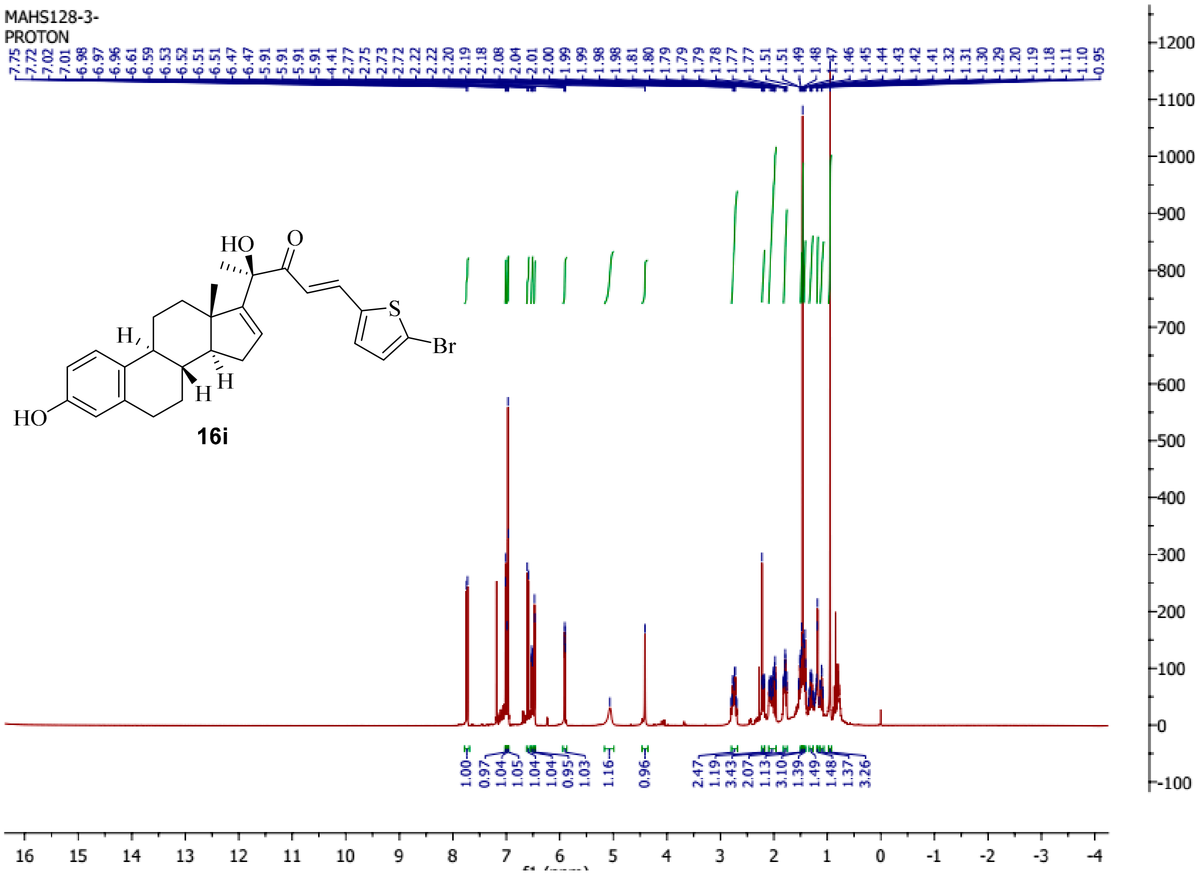

Figure S 64. ^{1}H NMR of compound **16i**.

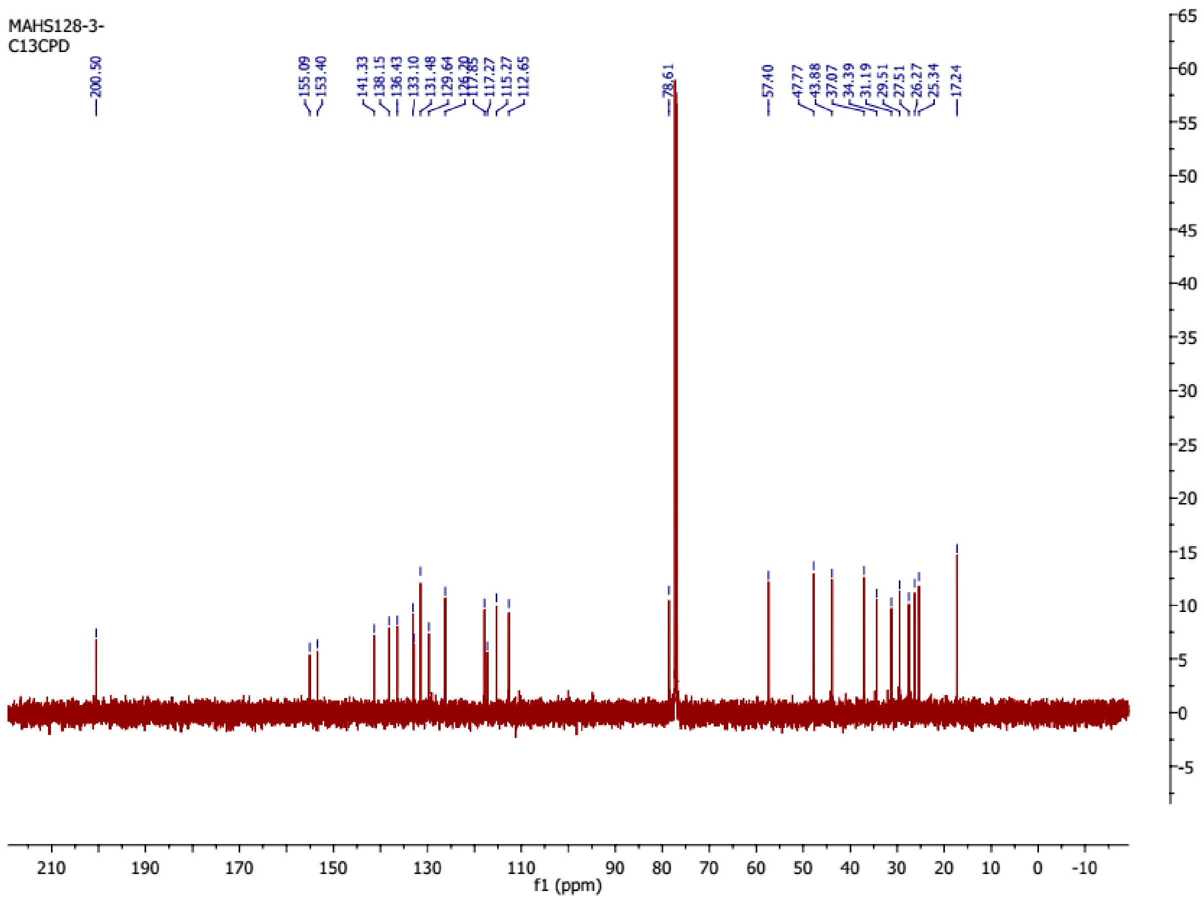

Figure S 65. ^{13}C NMR of compound **16i**.

1.32 *Compound 16j*

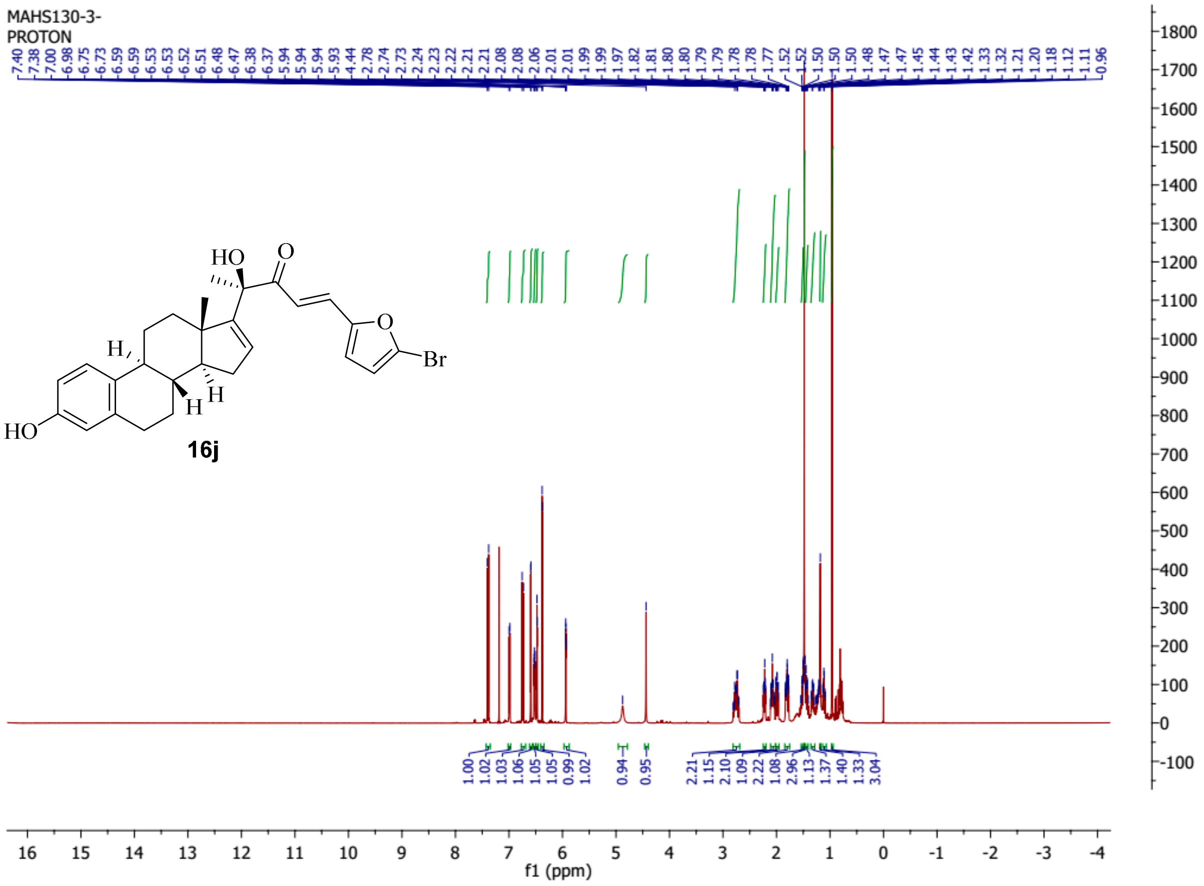

Figure S 66. ^{1}H NMR of compound **16j**.

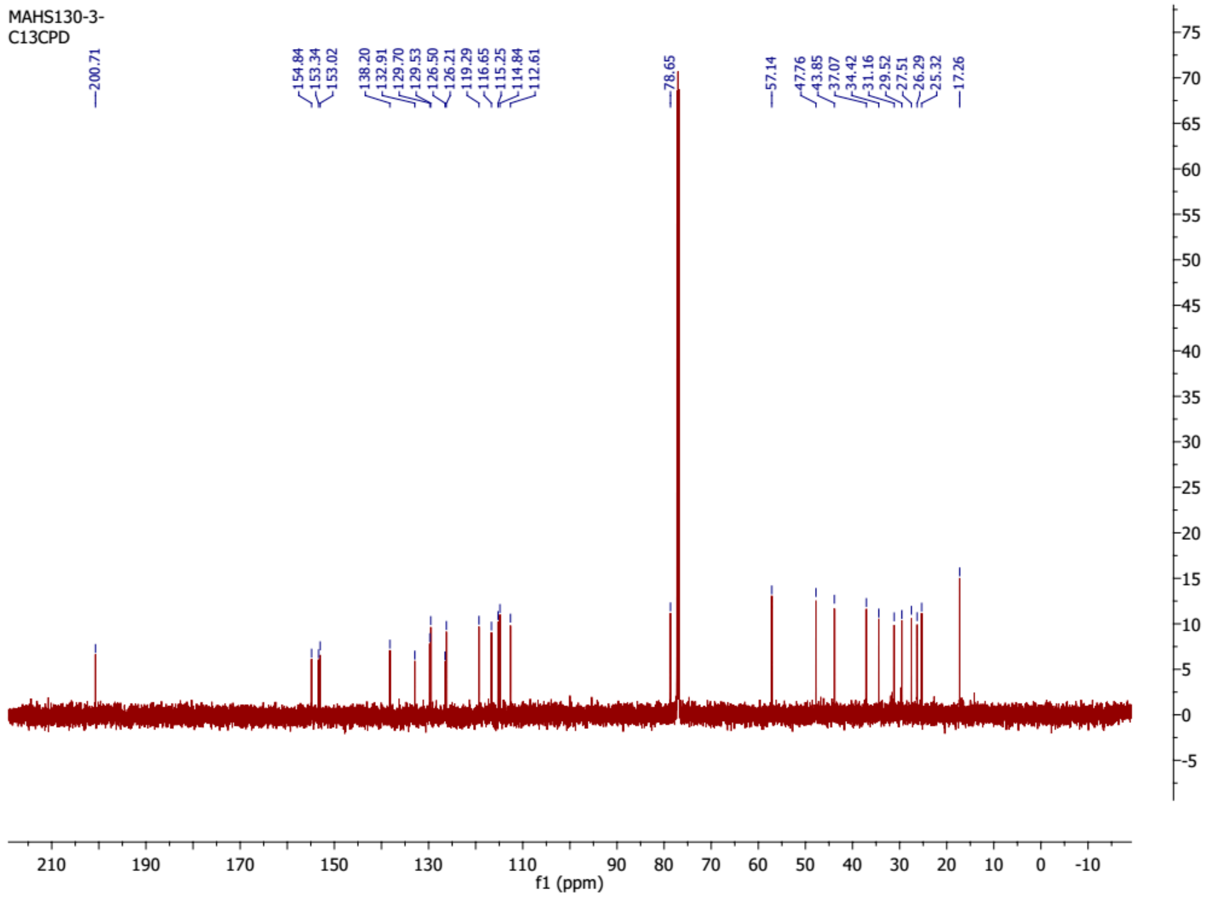

Figure S 67. ^{13}C NMR of compound **16j**.

1.33 Compound 17a

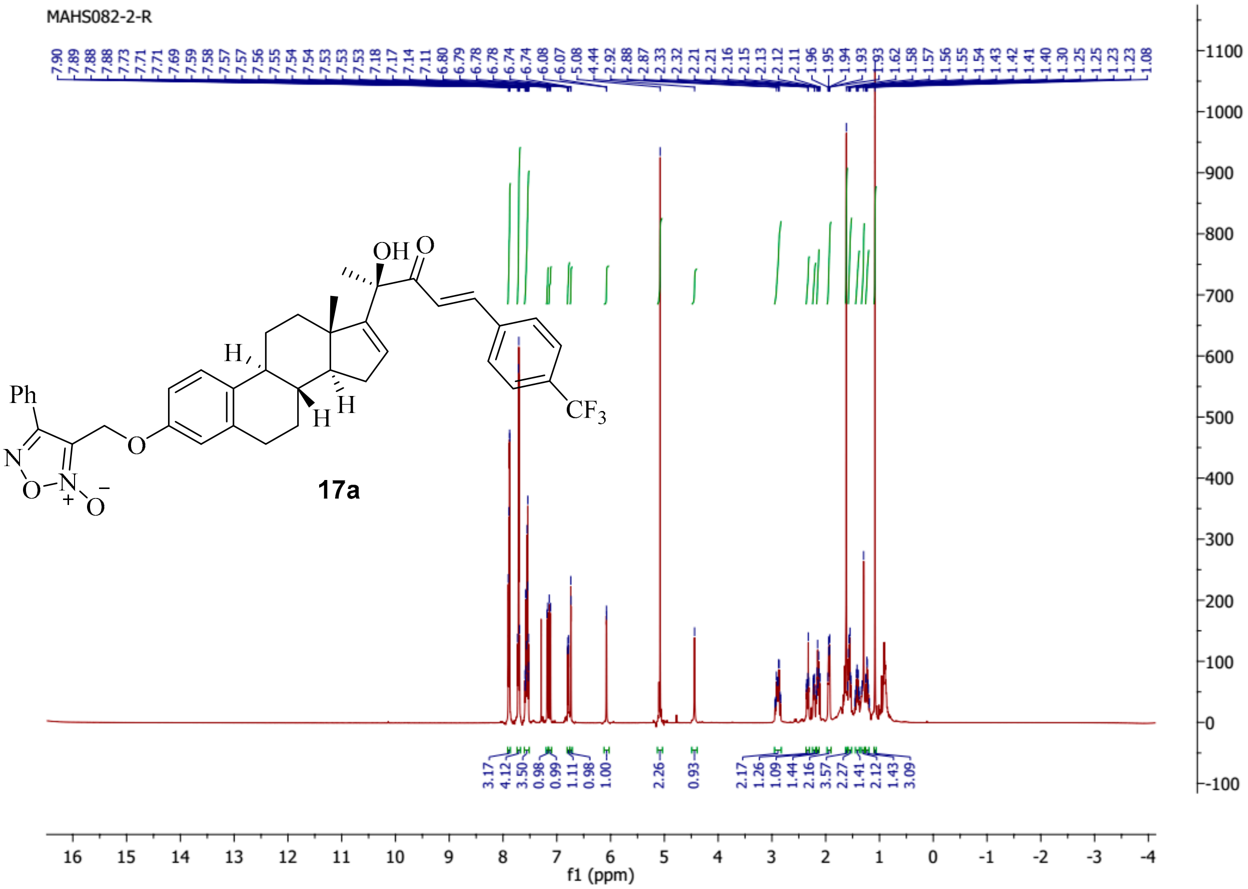

Figure S 68. ^{1}H NMR of compound **17a**.

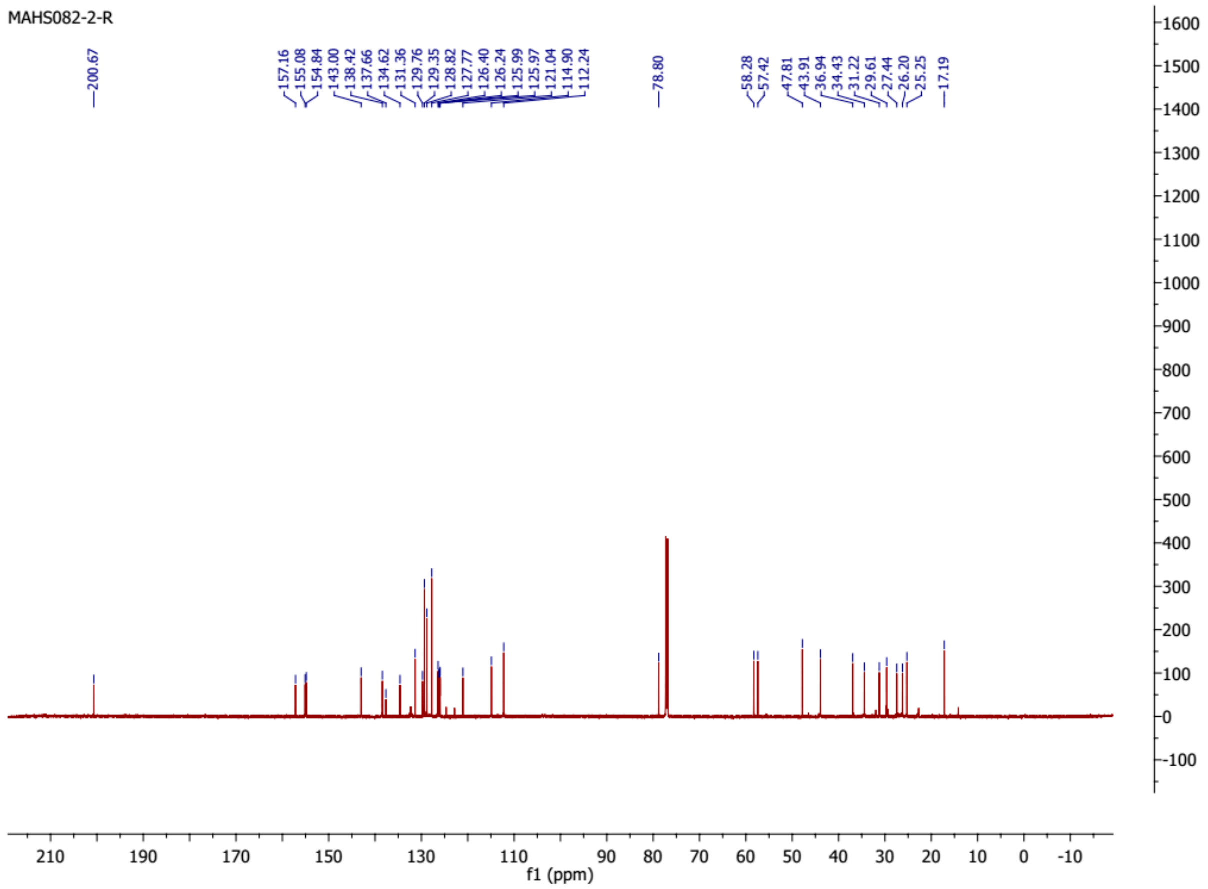

Figure S 69. ^{13}C NMR of compound **17a**.

1.34 Compound 17b

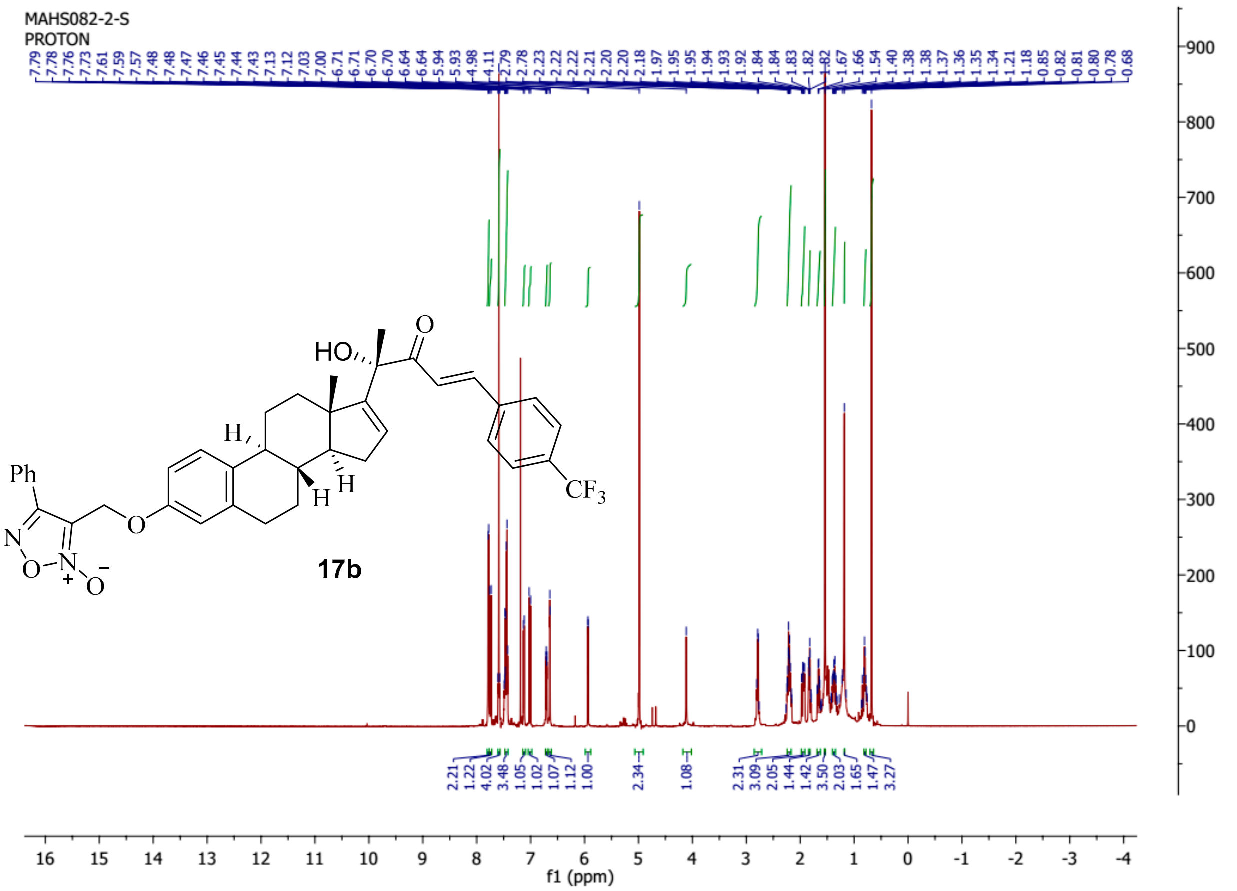

Figure S 70. ^{1}H NMR of compound **17b**.

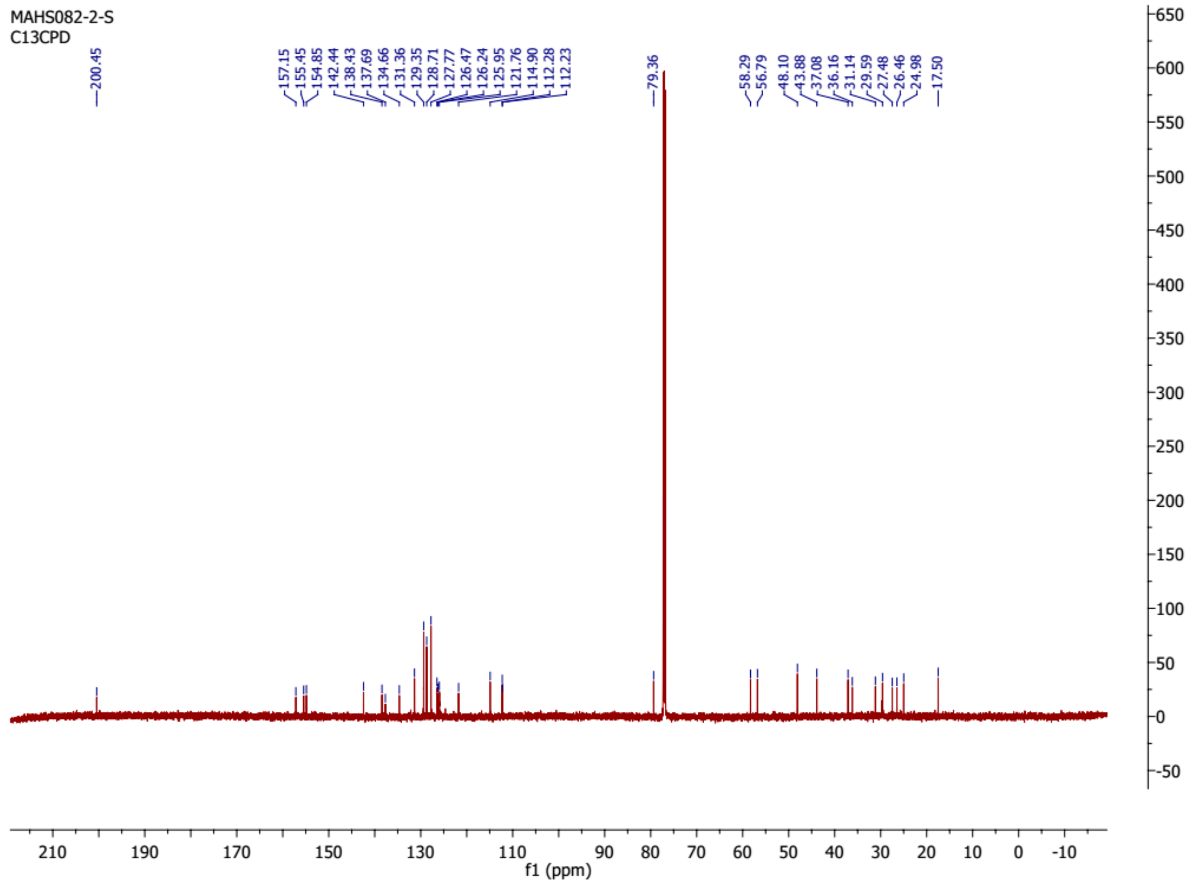

Figure S 71. ^{13}C NMR of compound **17b**.

1.35 Compound 17c

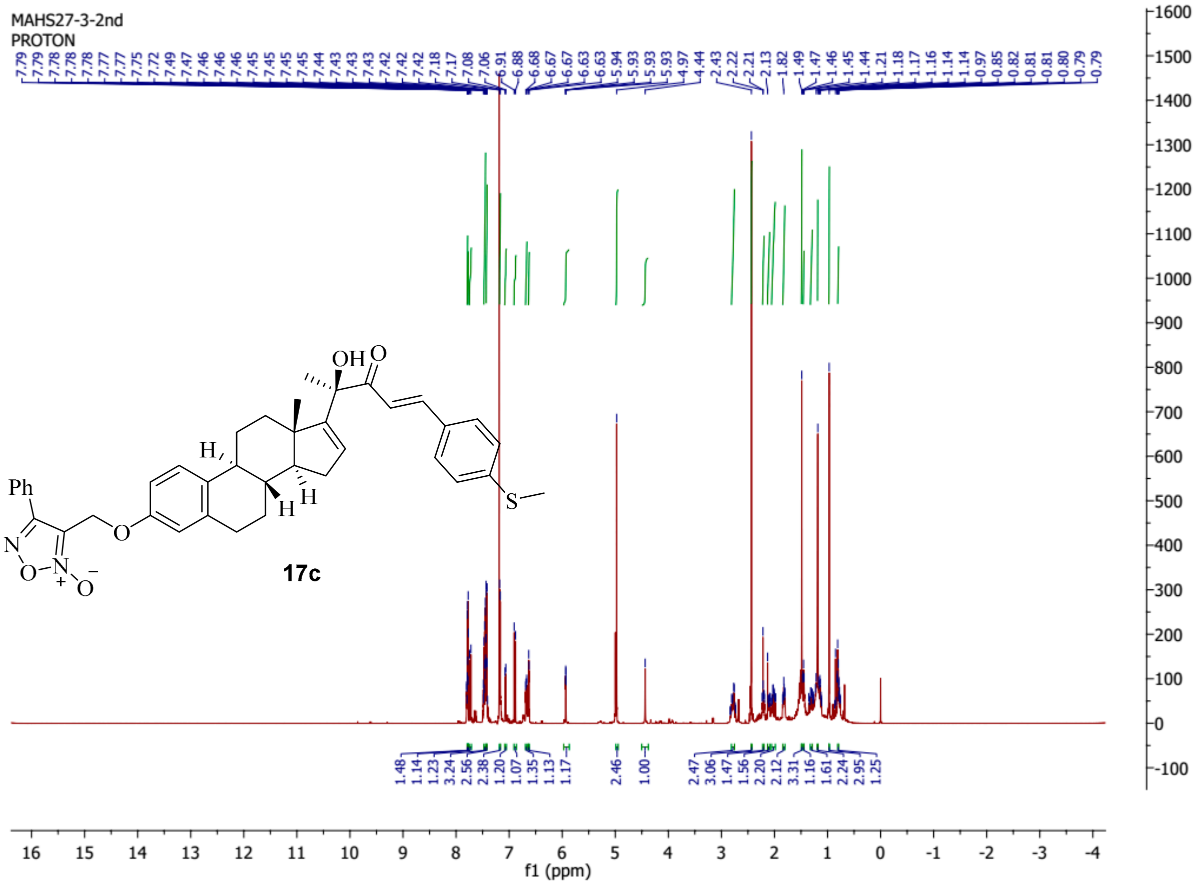

Figure S 72. ^{1}H NMR of compound **17c**.

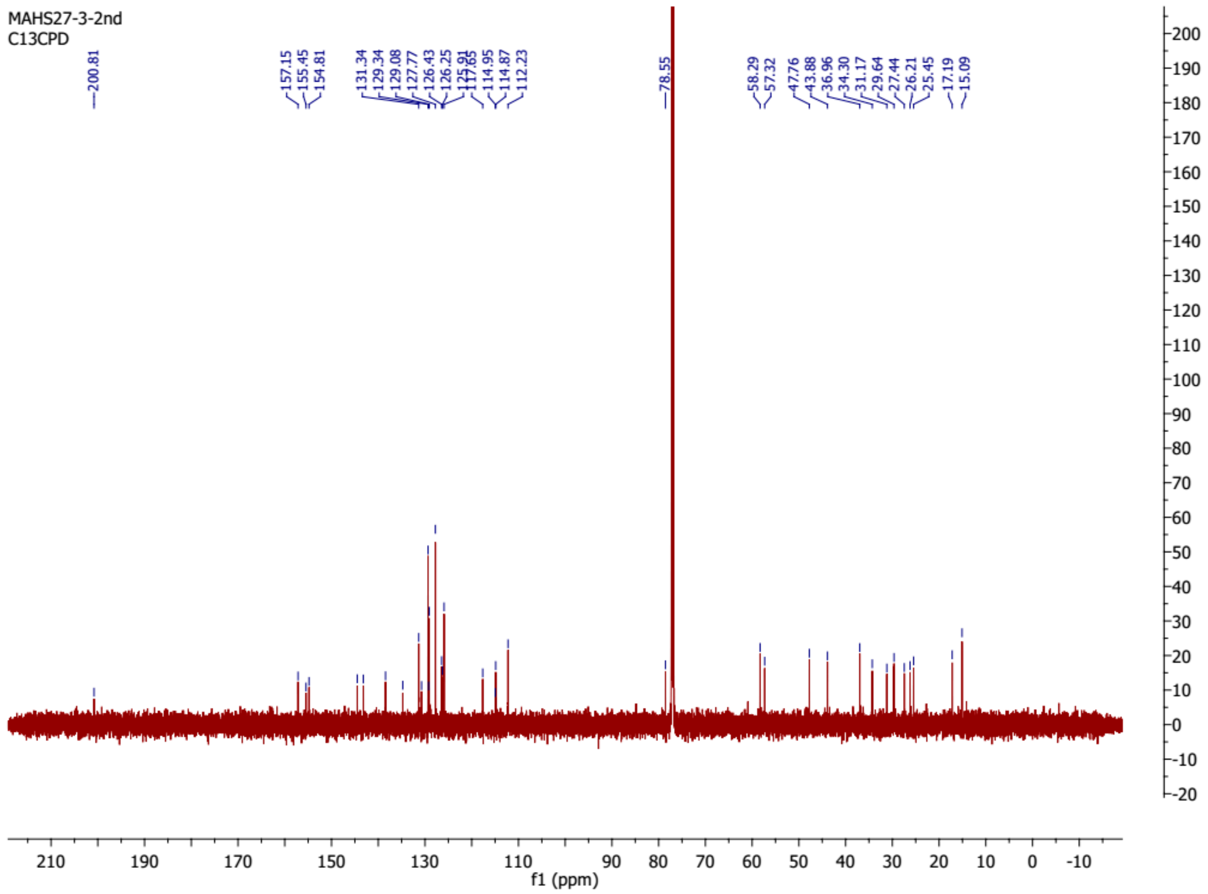

Figure S 73. ^{13}C NMR of compound **17c**.

1.36 Compound 17d

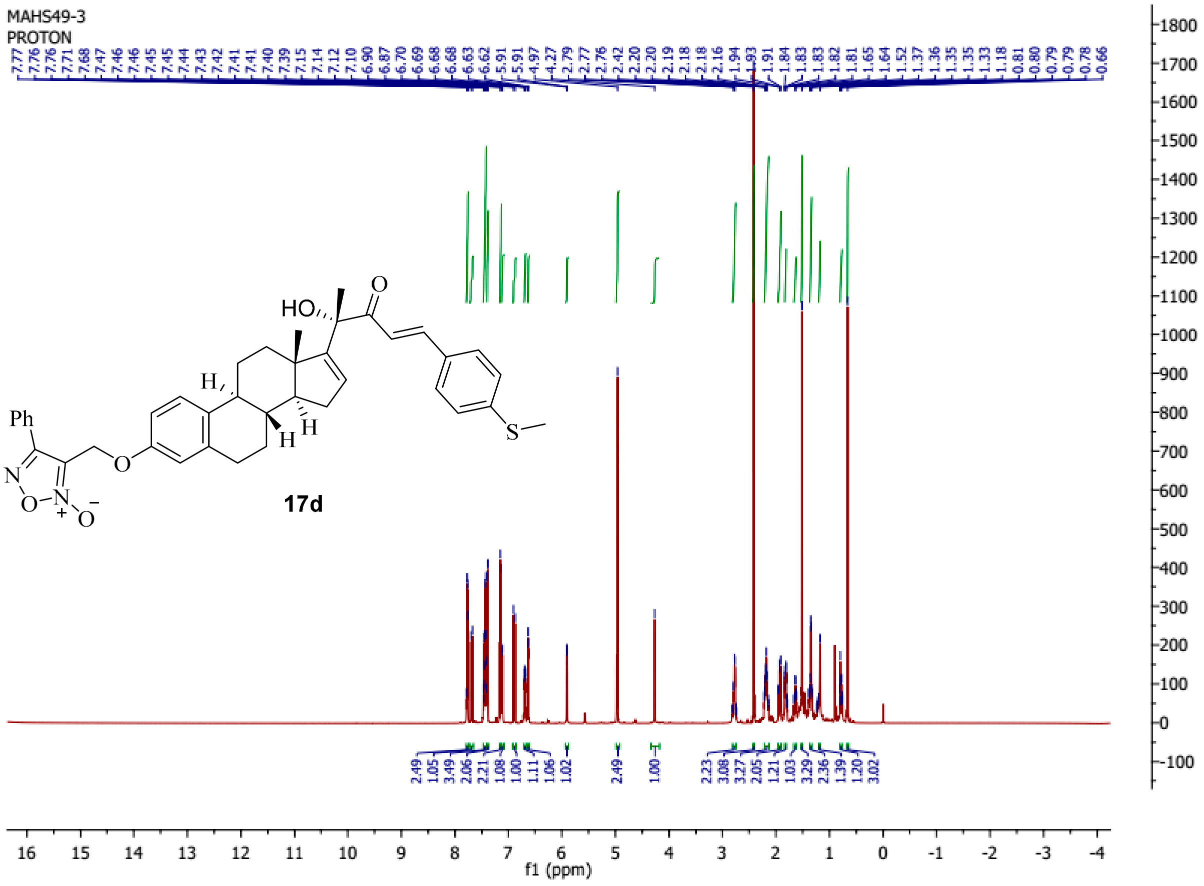

Figure S 74. ^{1}H NMR of compound **17d**.

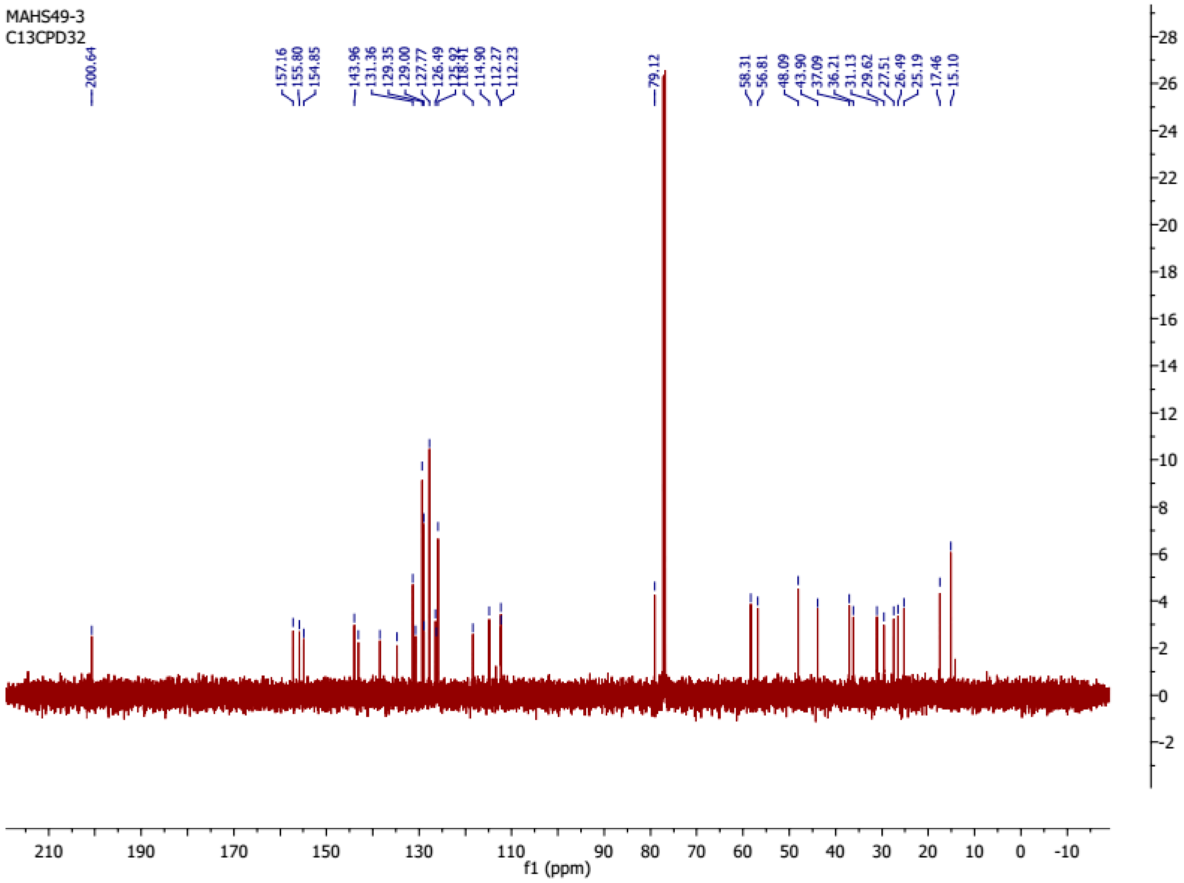

Figure S 75. ^{13}C NMR of compound **17d**.

1.37 Compound 17e

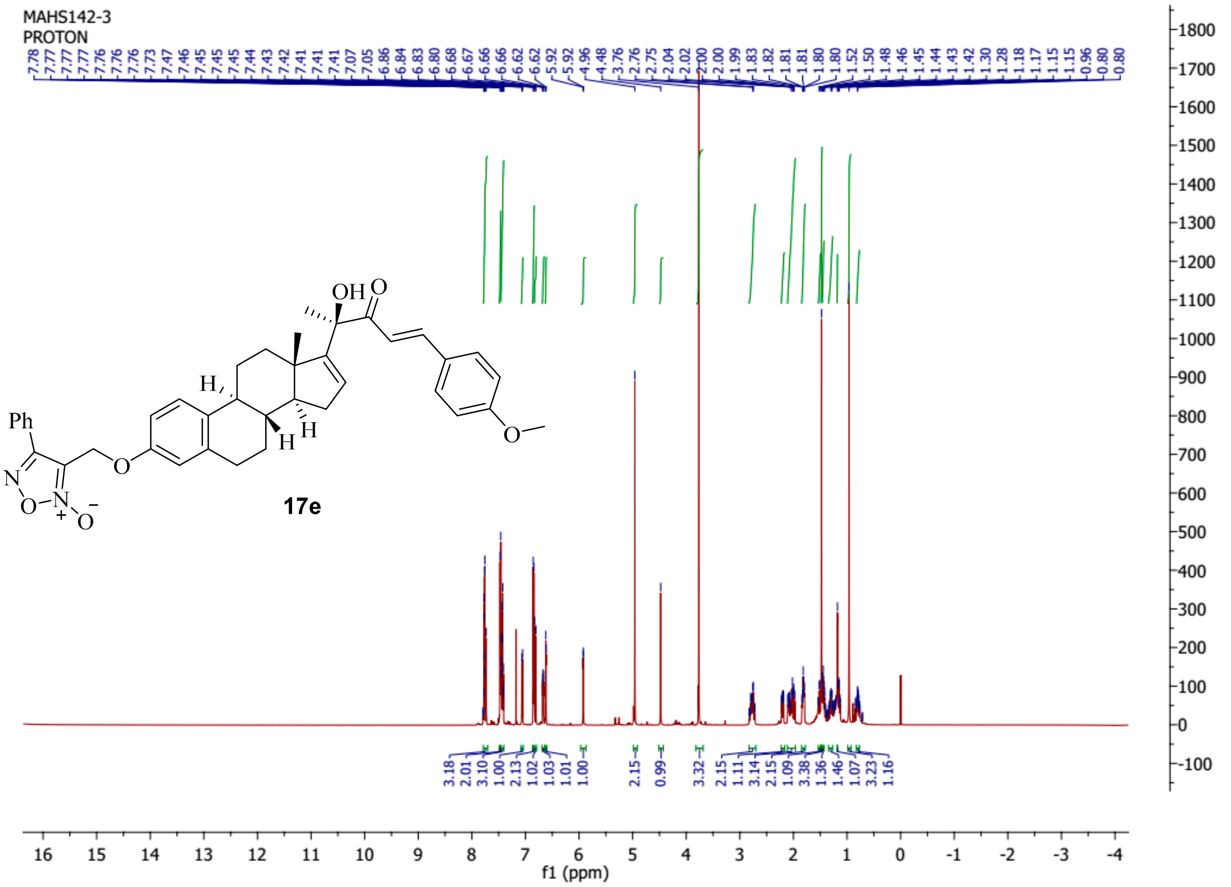

Figure S 76. ^{1}H NMR of compound **17e**.

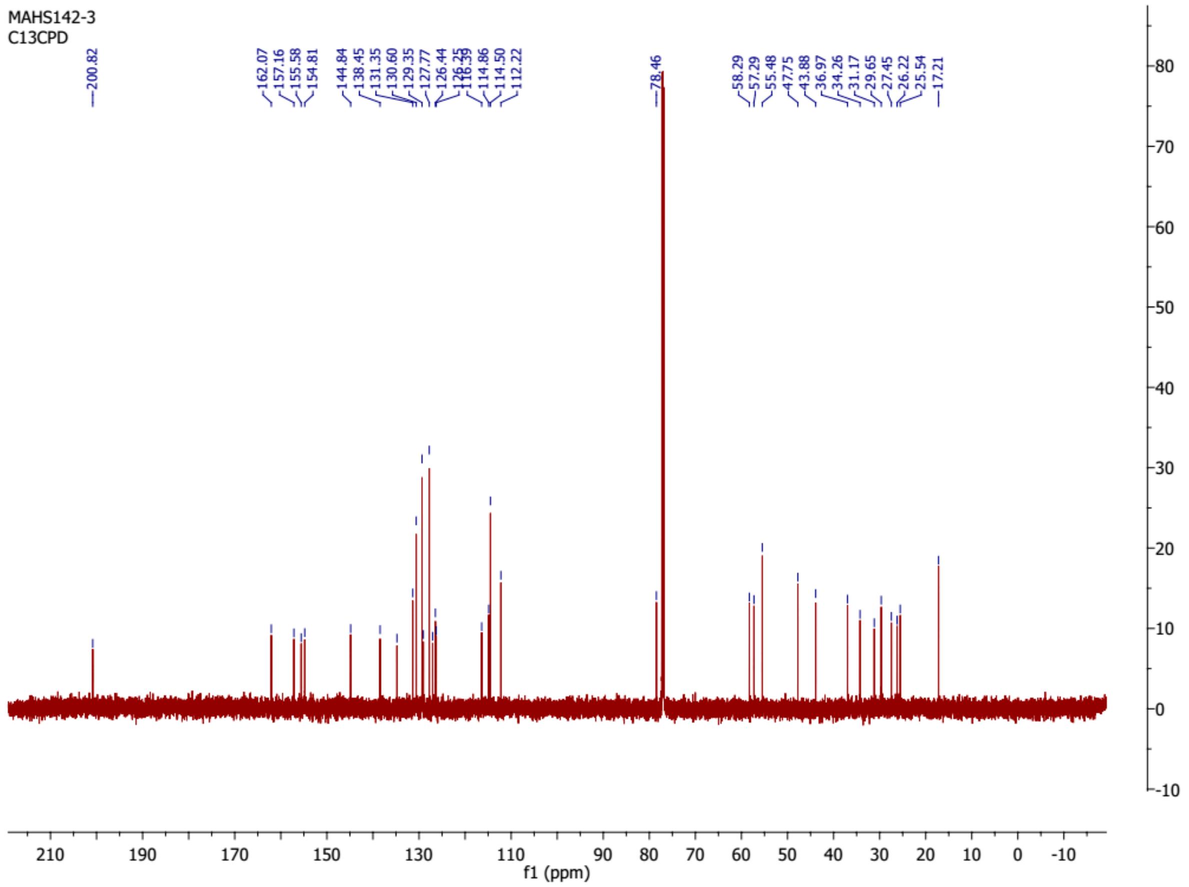

Figure S 77. ^{13}C NMR of compound **17e**.

1.38 Compound 17f

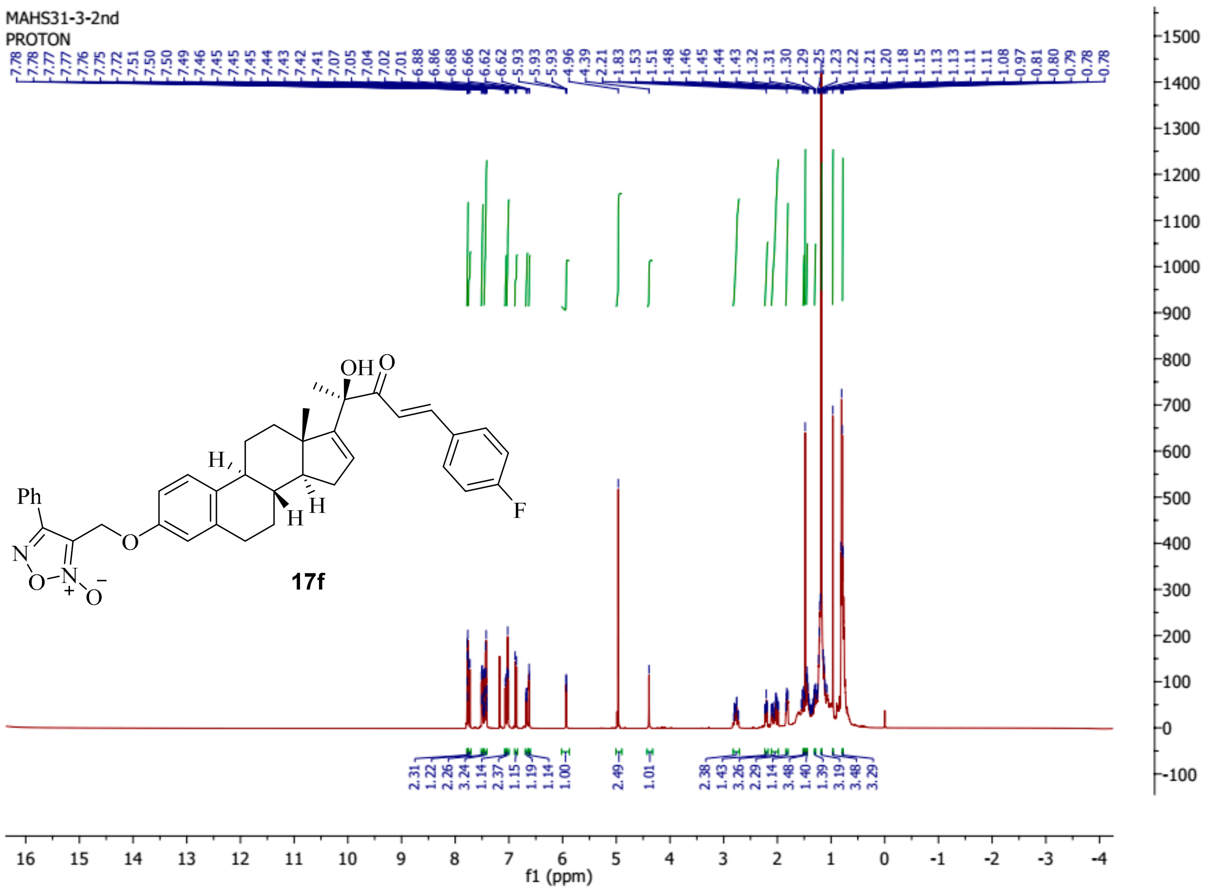

Figure S 78. ^{1}H NMR of compound **17f**.

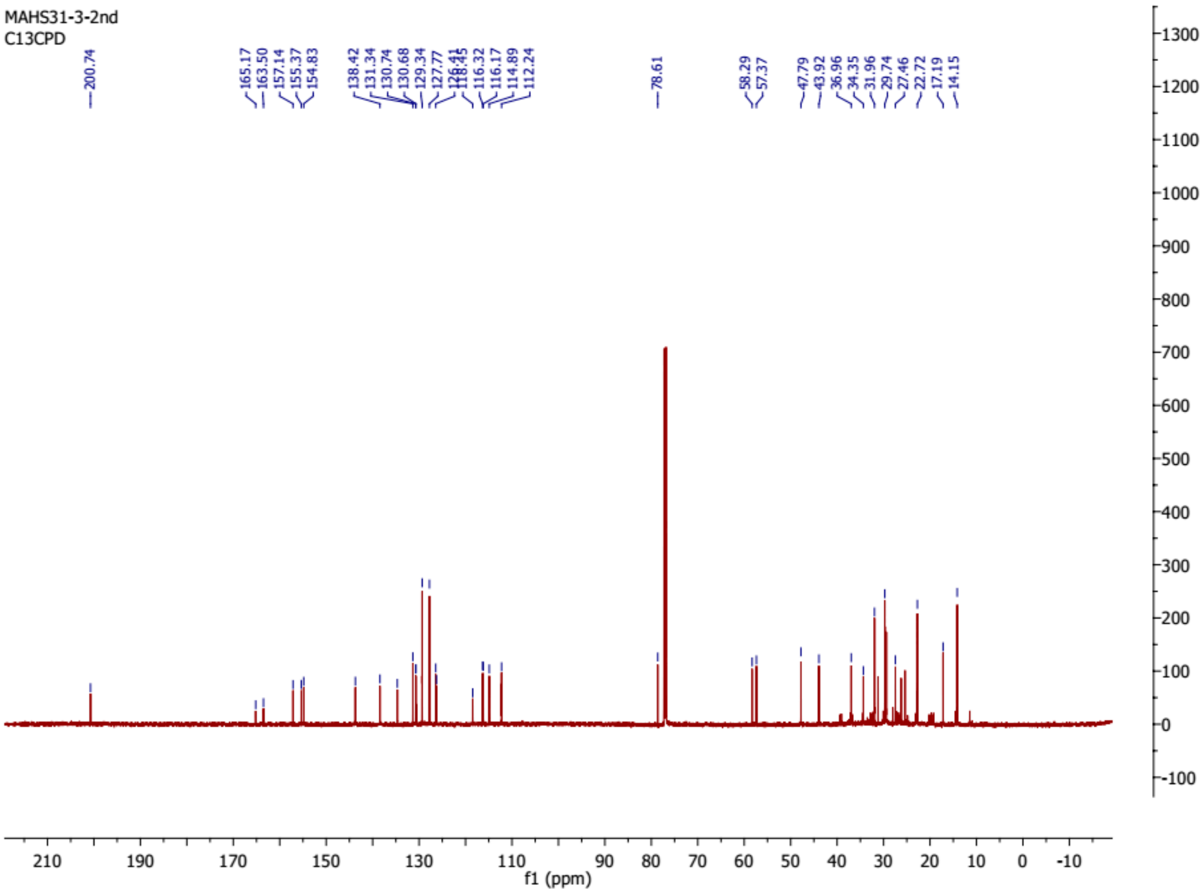

Figure S 79. ^{13}C NMR of compound **17f**.

1.39 Compound 17g

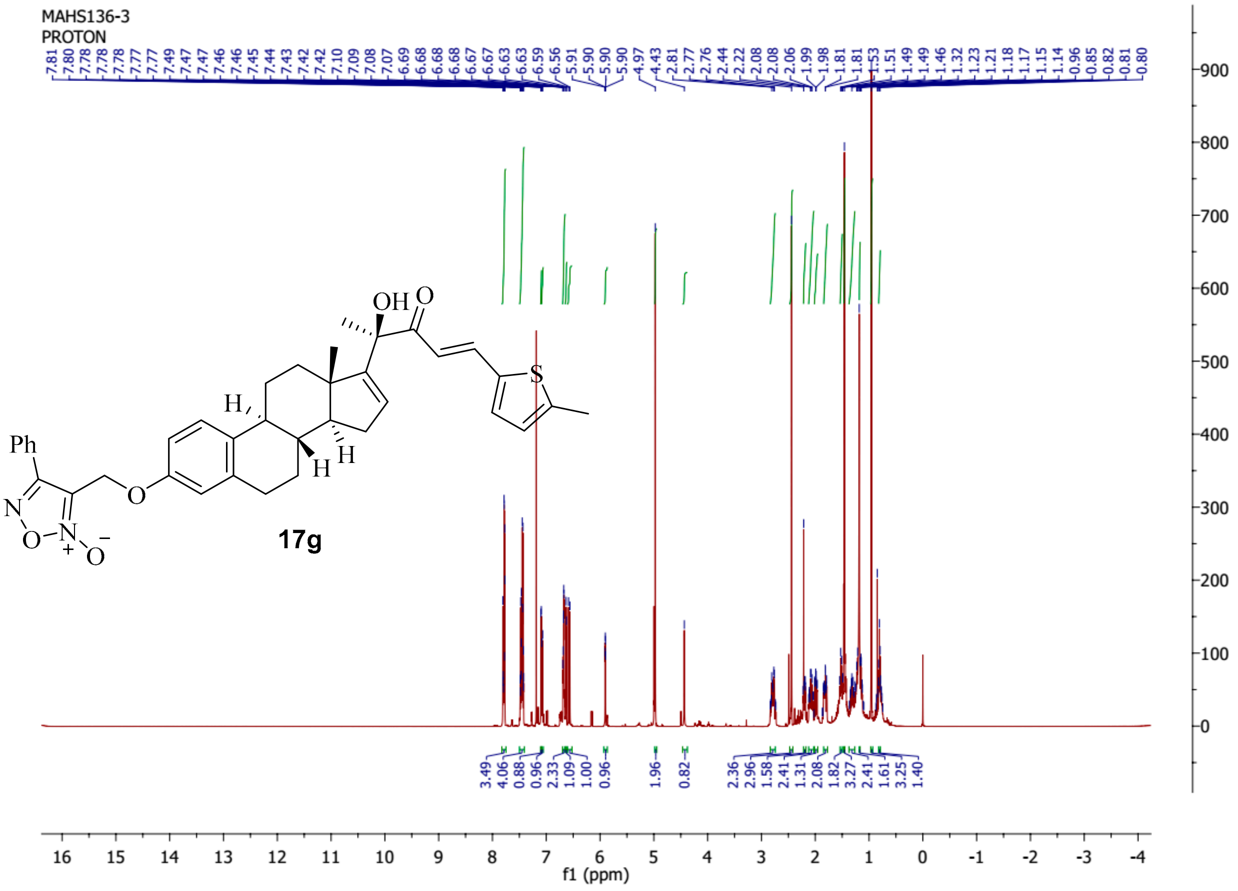

Figure S 80. ^{1}H NMR of compound **17g**.

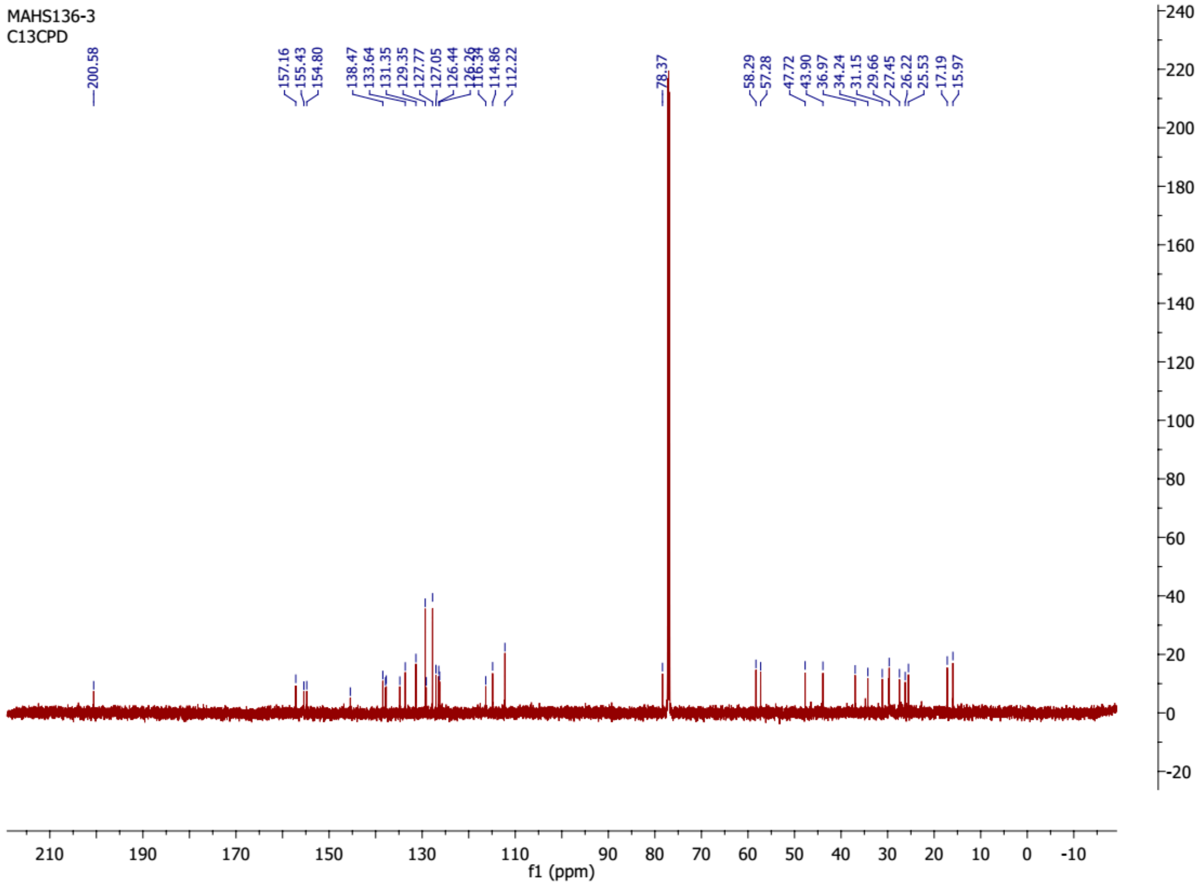

Figure S 81. ^{13}C NMR of compound **17g**.

1.40 Compound 17h

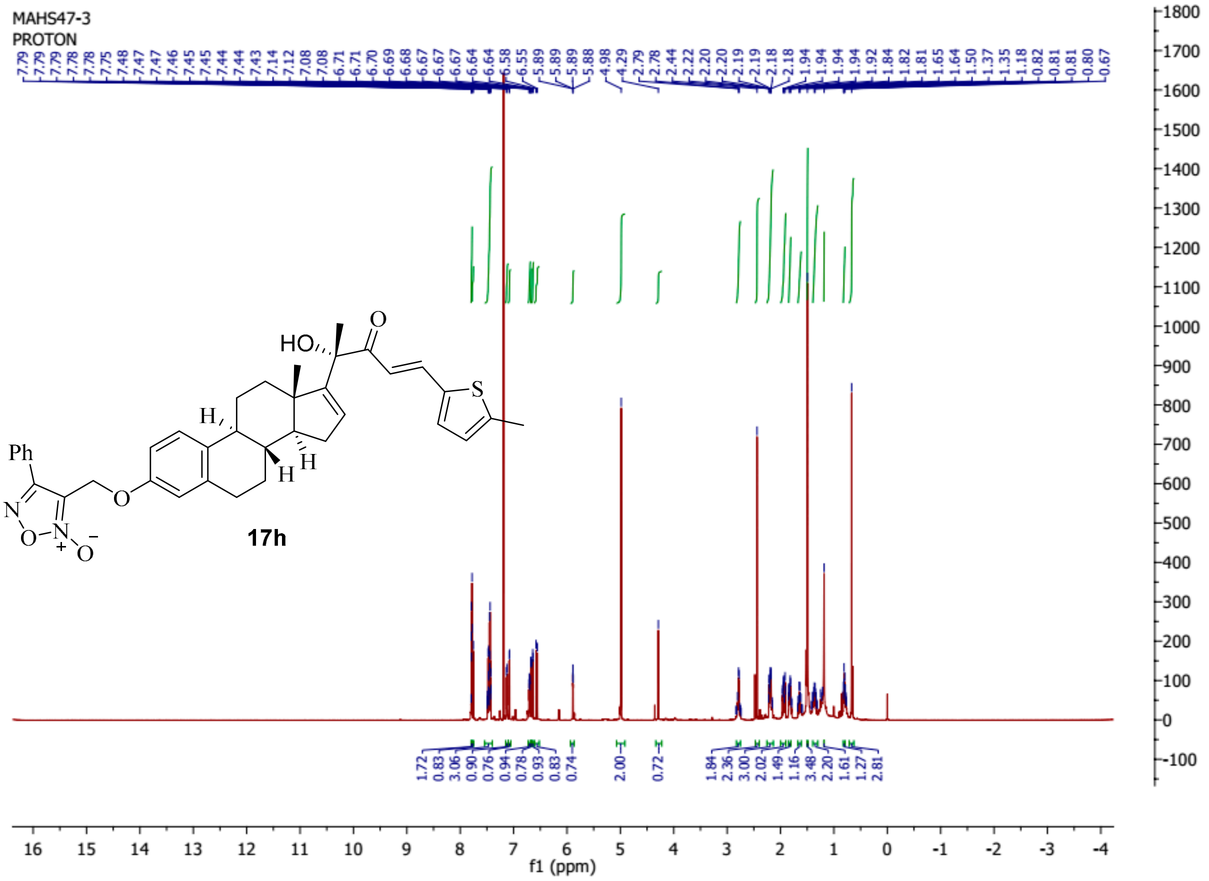

Figure S 82. ^{1}H NMR of compound **17h**.

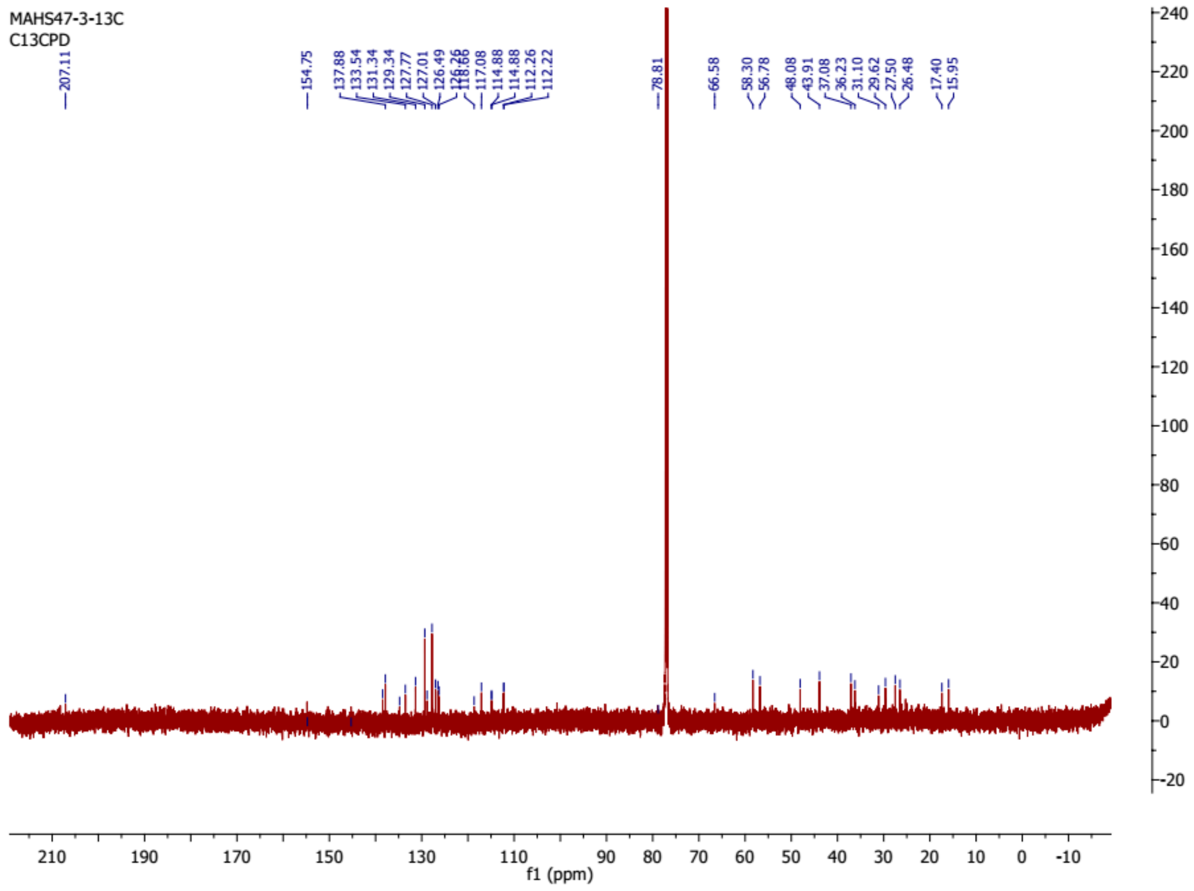

Figure S 83. ^{13}C NMR of compound **17h**.

1.41 Compound 17i

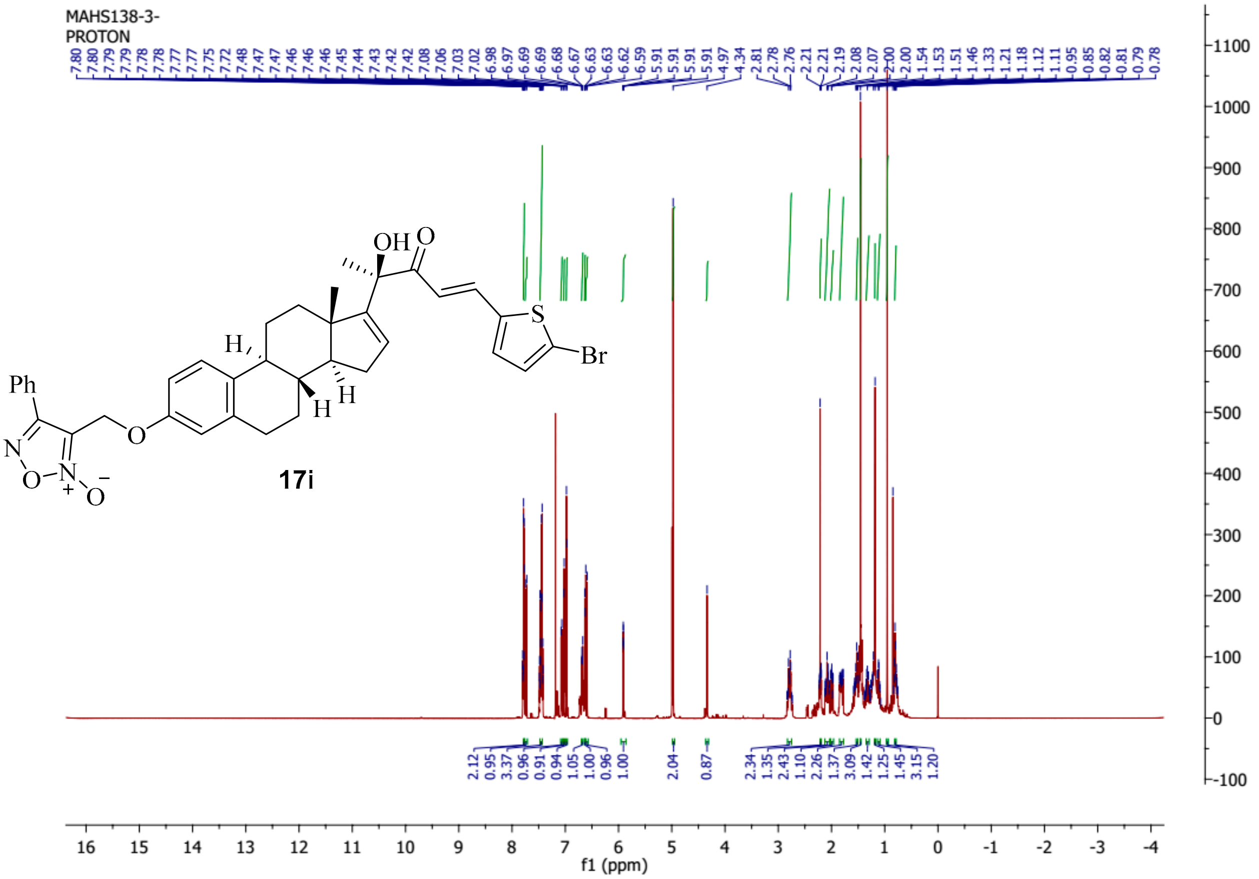

Figure S 84. ^{1}H NMR of compound **17i**.

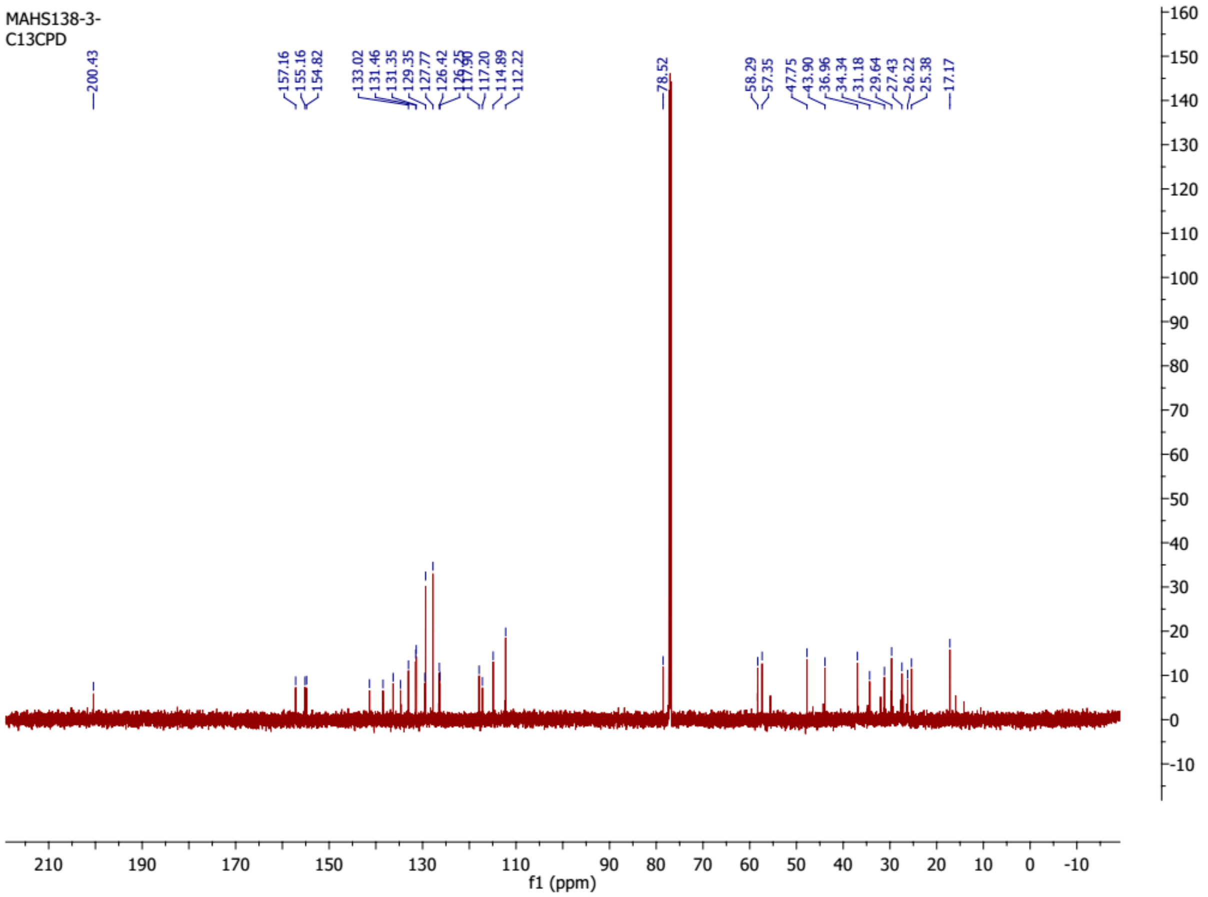

Figure S 85. ^{13}C NMR of compound **17i**.

1.42 Compound 17j

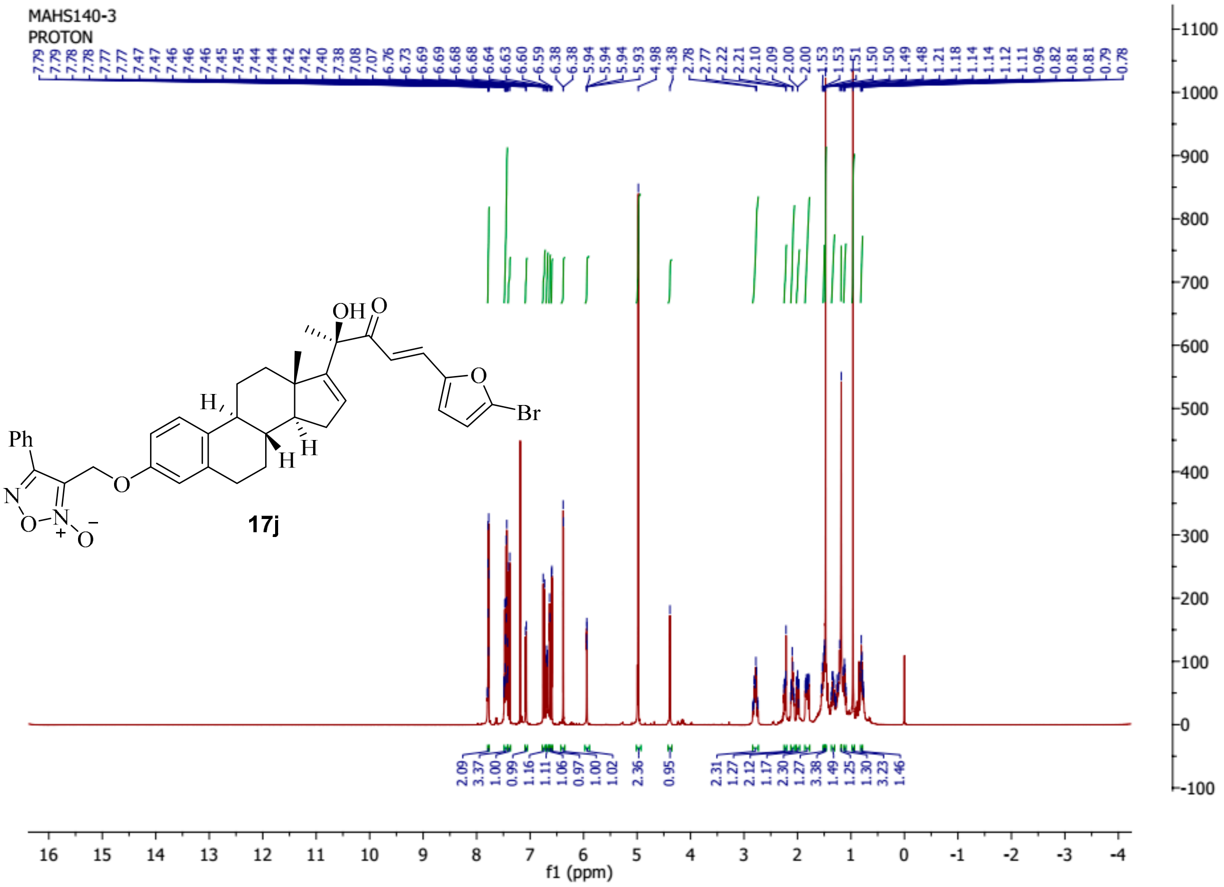

Figure S 86. ^{1}H NMR of compound **17j**.

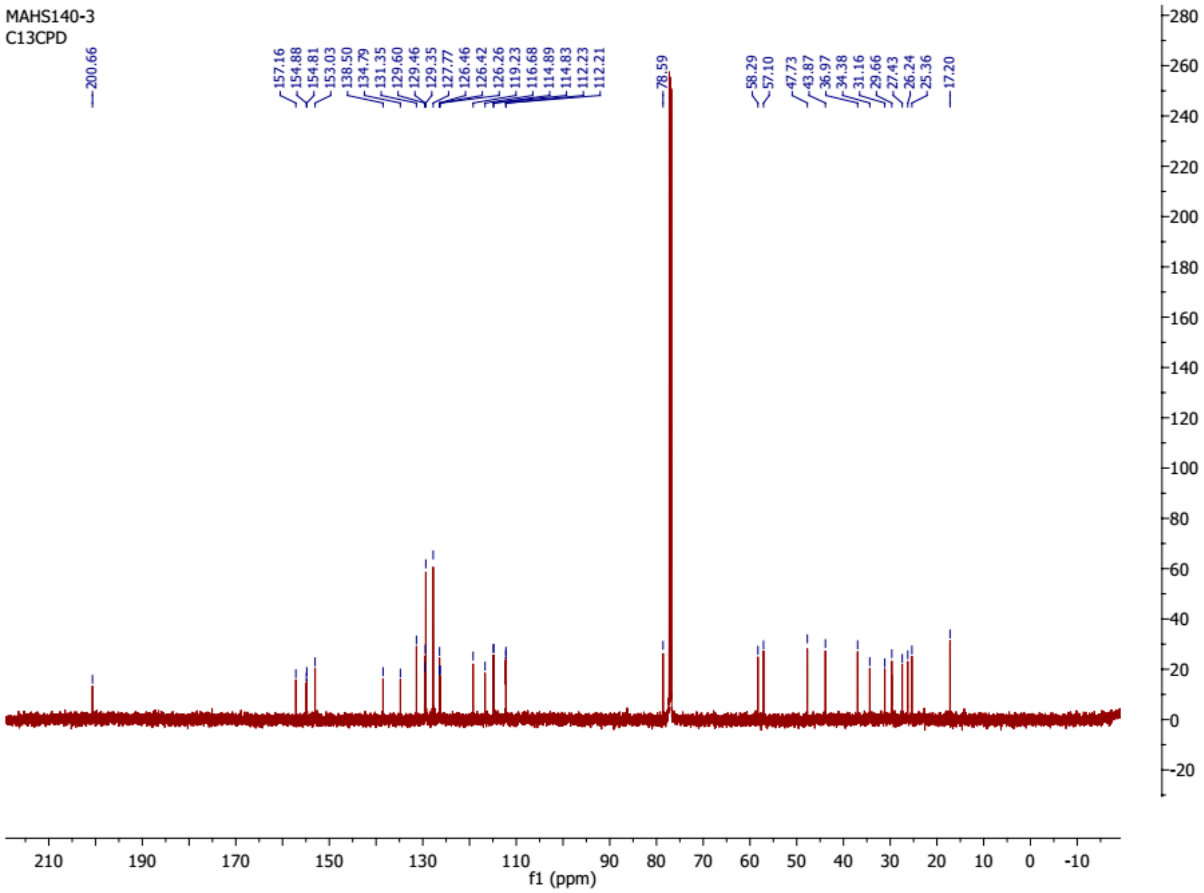

Figure S 87. ^{13}C NMR of compound **17j**.

1.43 Compound 18

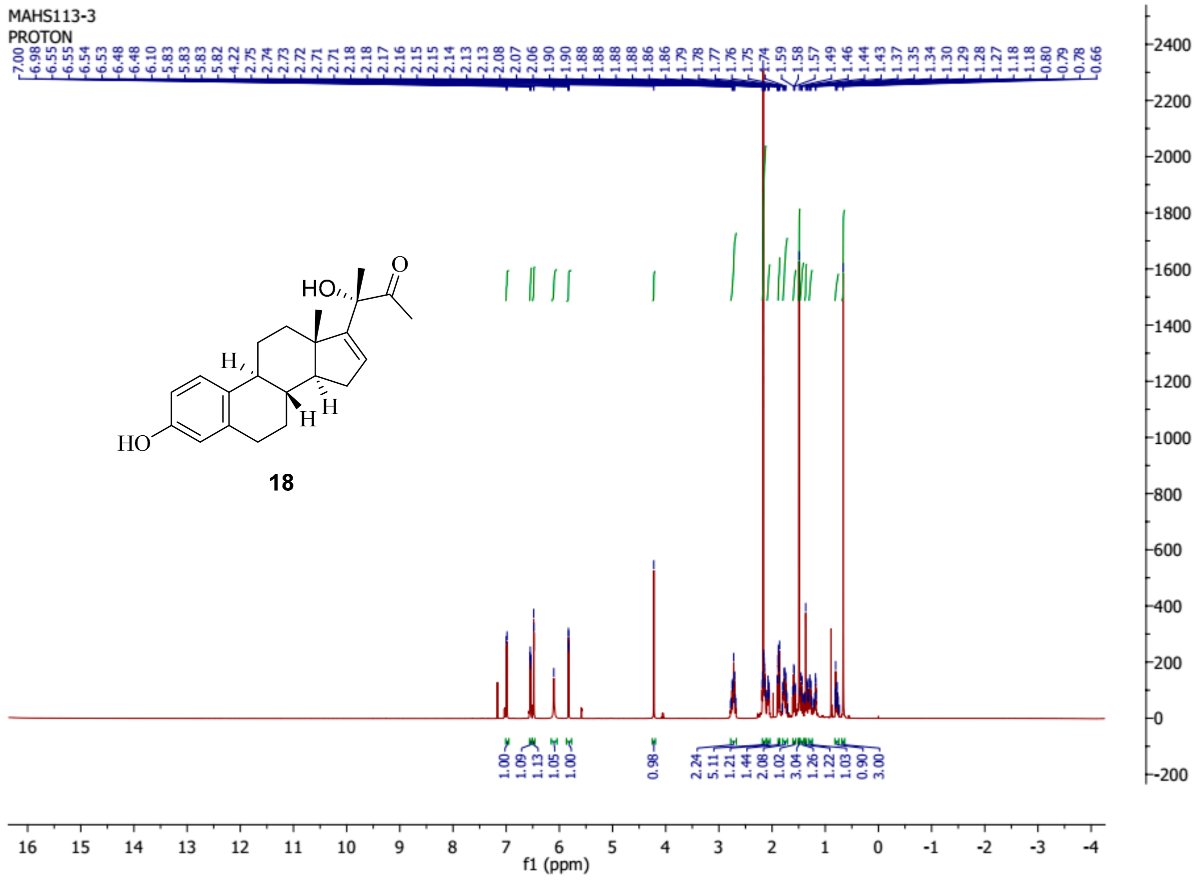

Figure S 88. ^{1}H NMR of compound **18**.

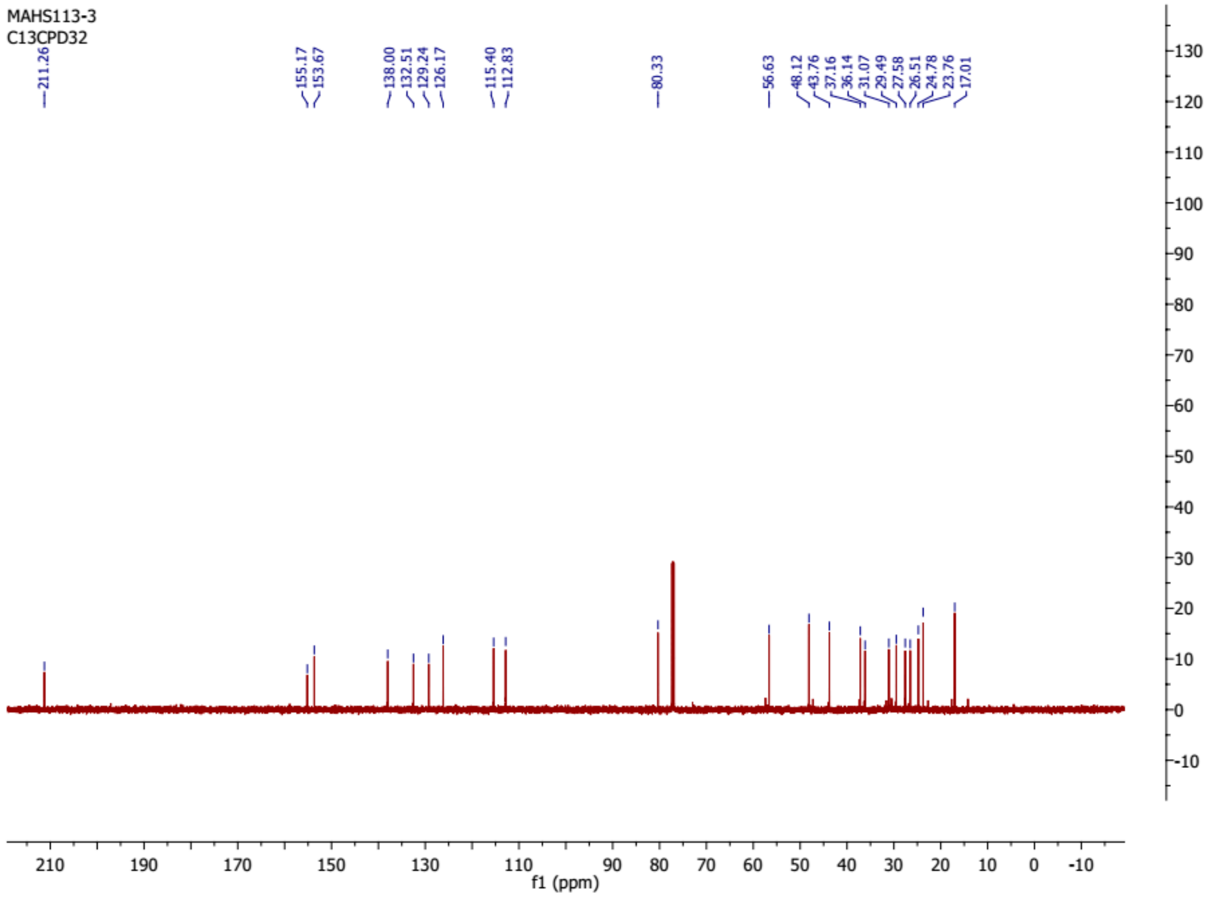

Figure S 89. ^{13}C NMR of compound **18**.

1.44 Compound 19

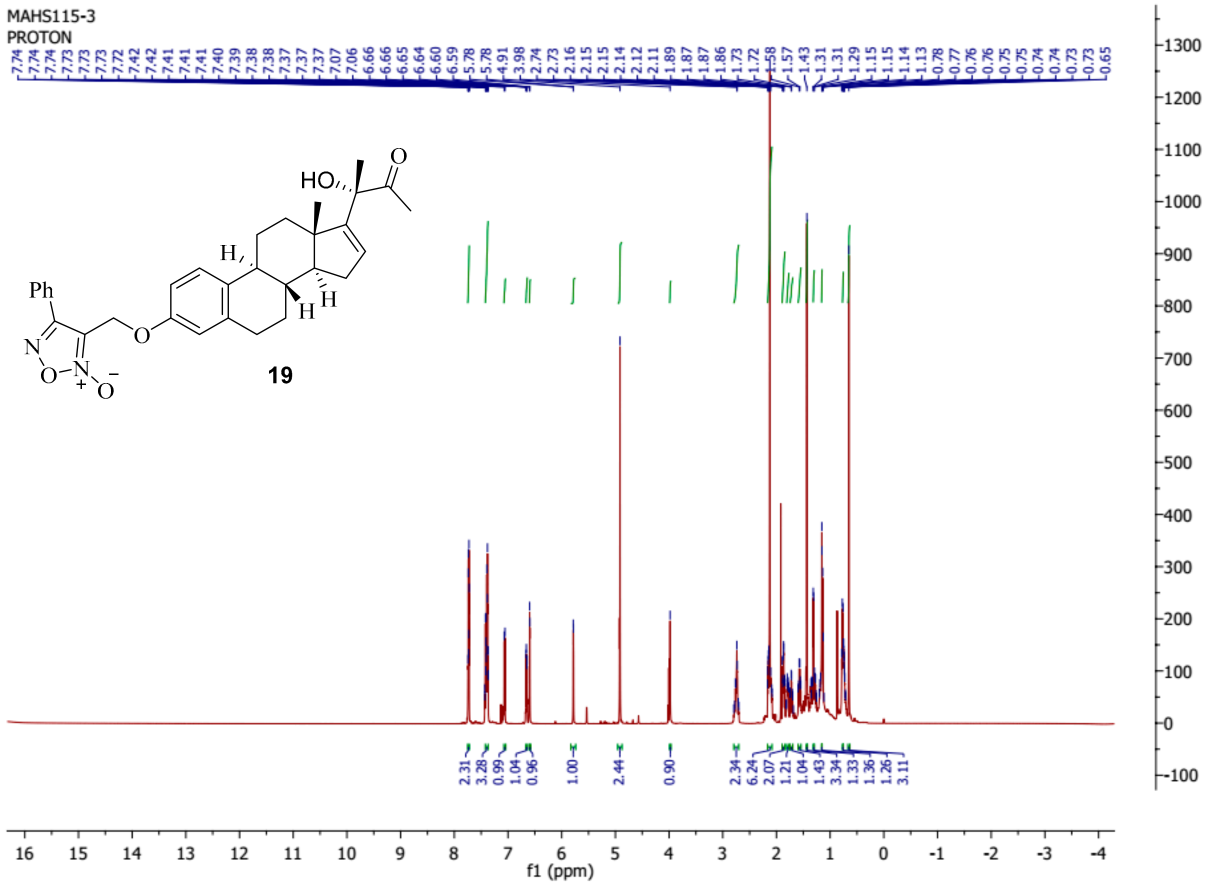

Figure S 90. ^{1}H NMR of compound **19**.

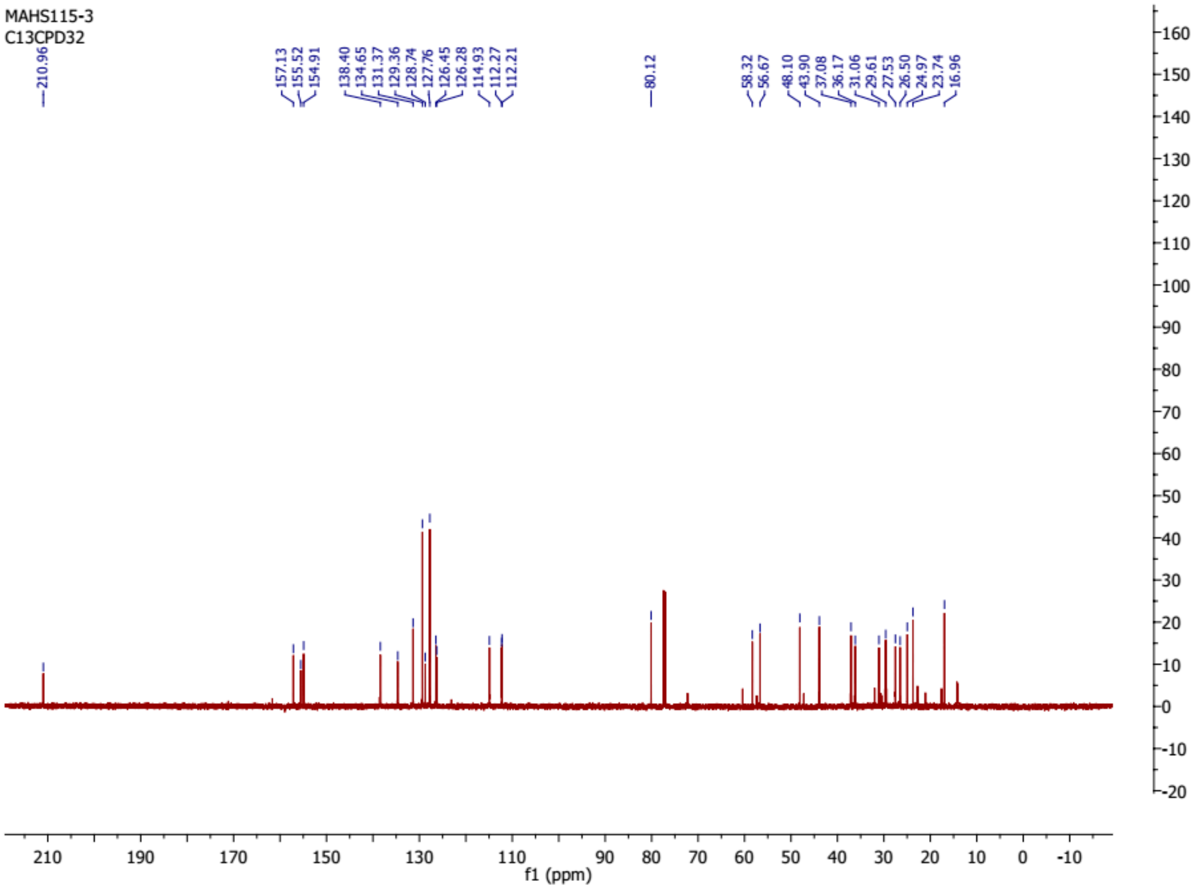

Figure S 91. ^{13}C NMR of compound **19**.

1.45 Compound 20a

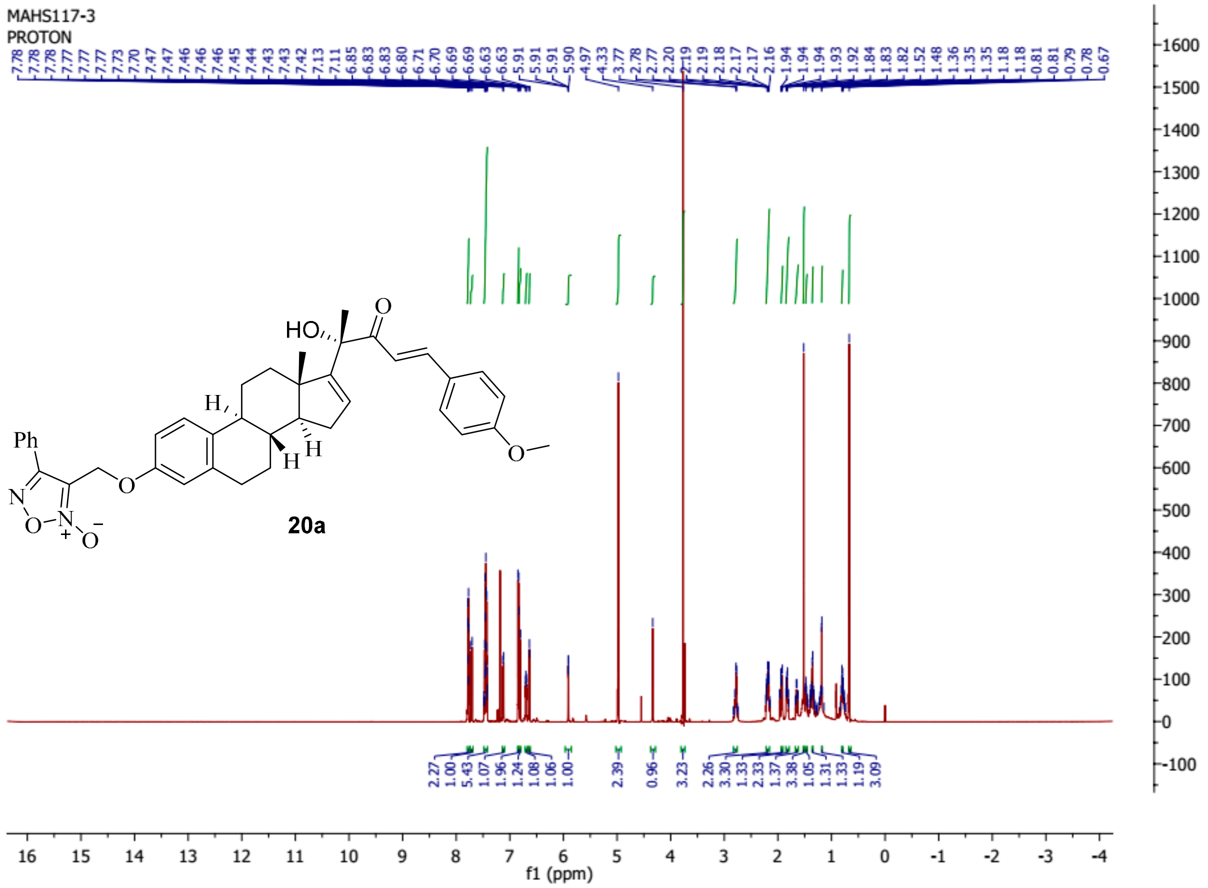

Figure S 92. ^{1}H NMR of compound **20a**.

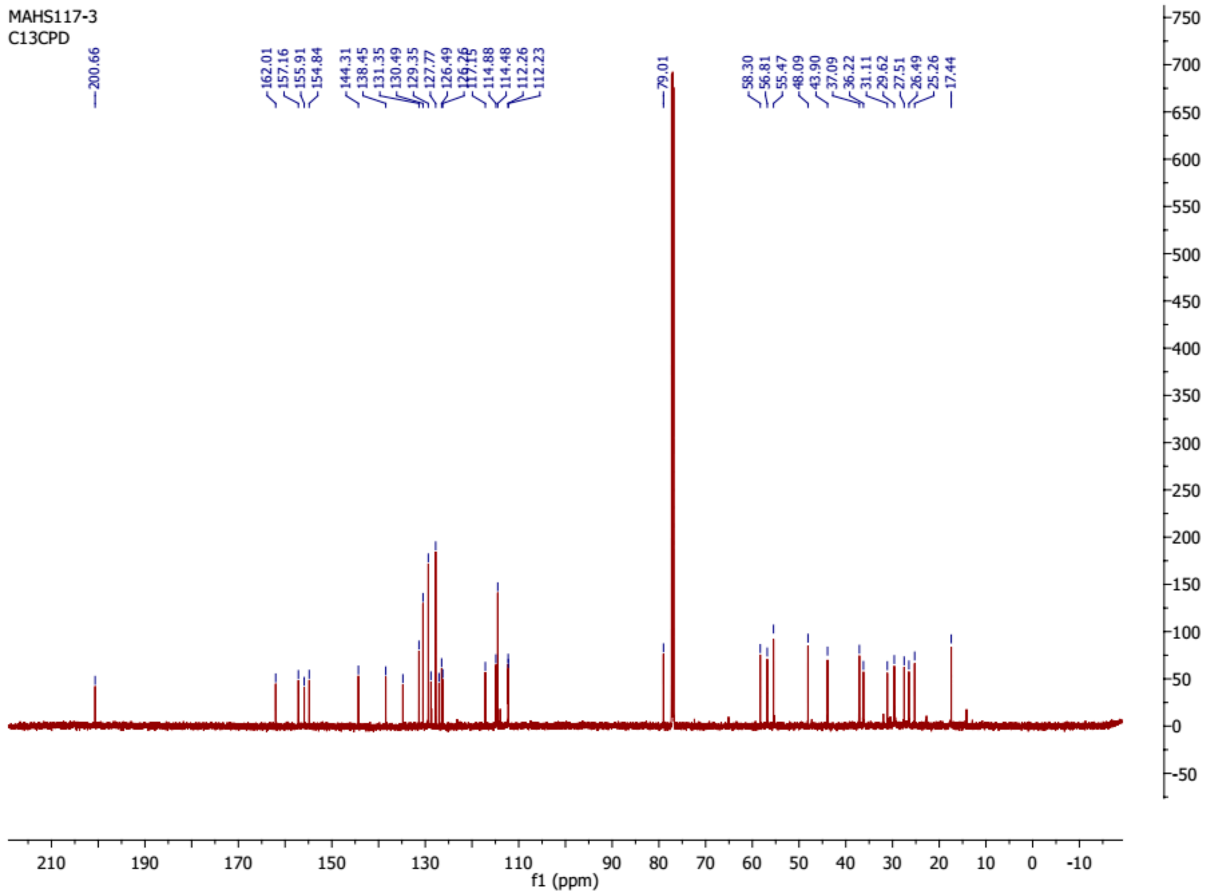

Figure S 93. ^{13}C NMR of compound **20a**.

1.46 Compound 20b

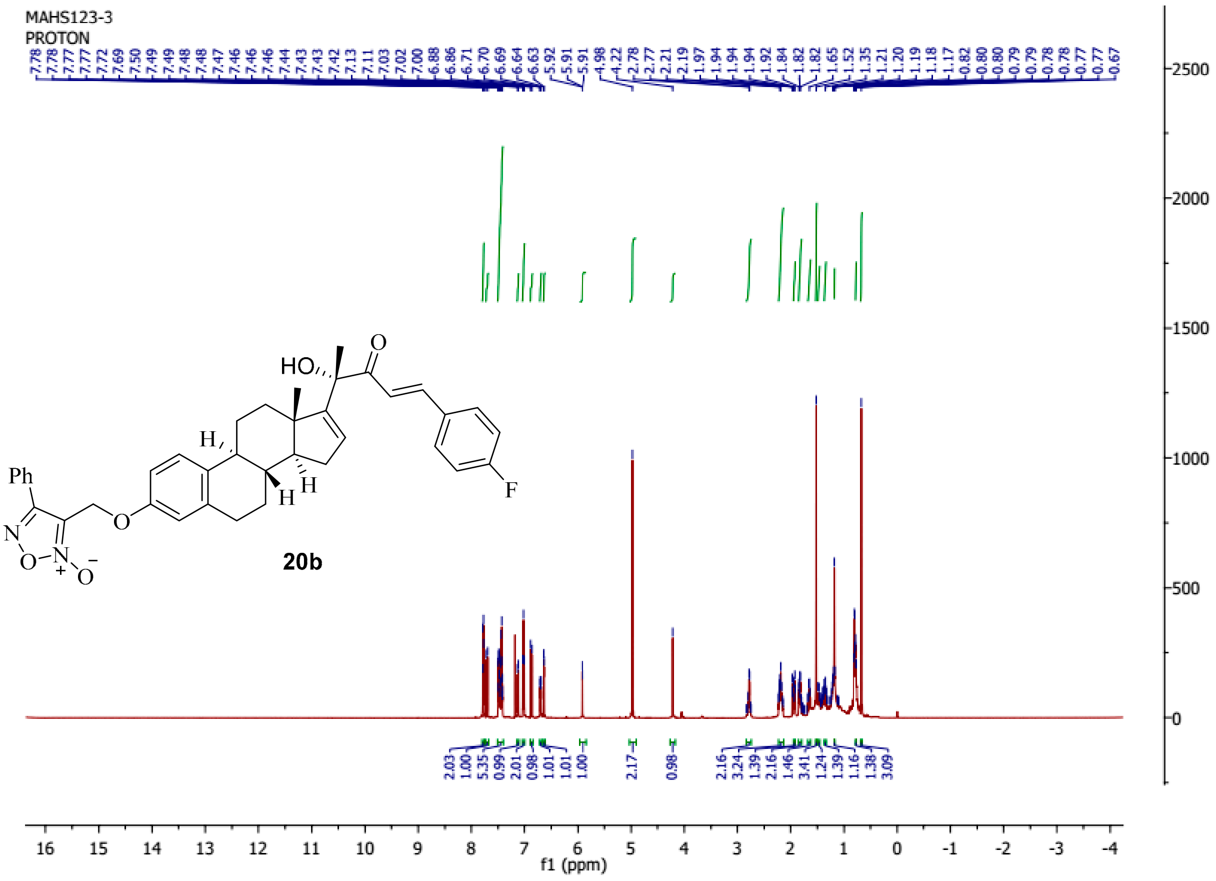

Figure S 94. ^{1}H NMR of compound **20b**.

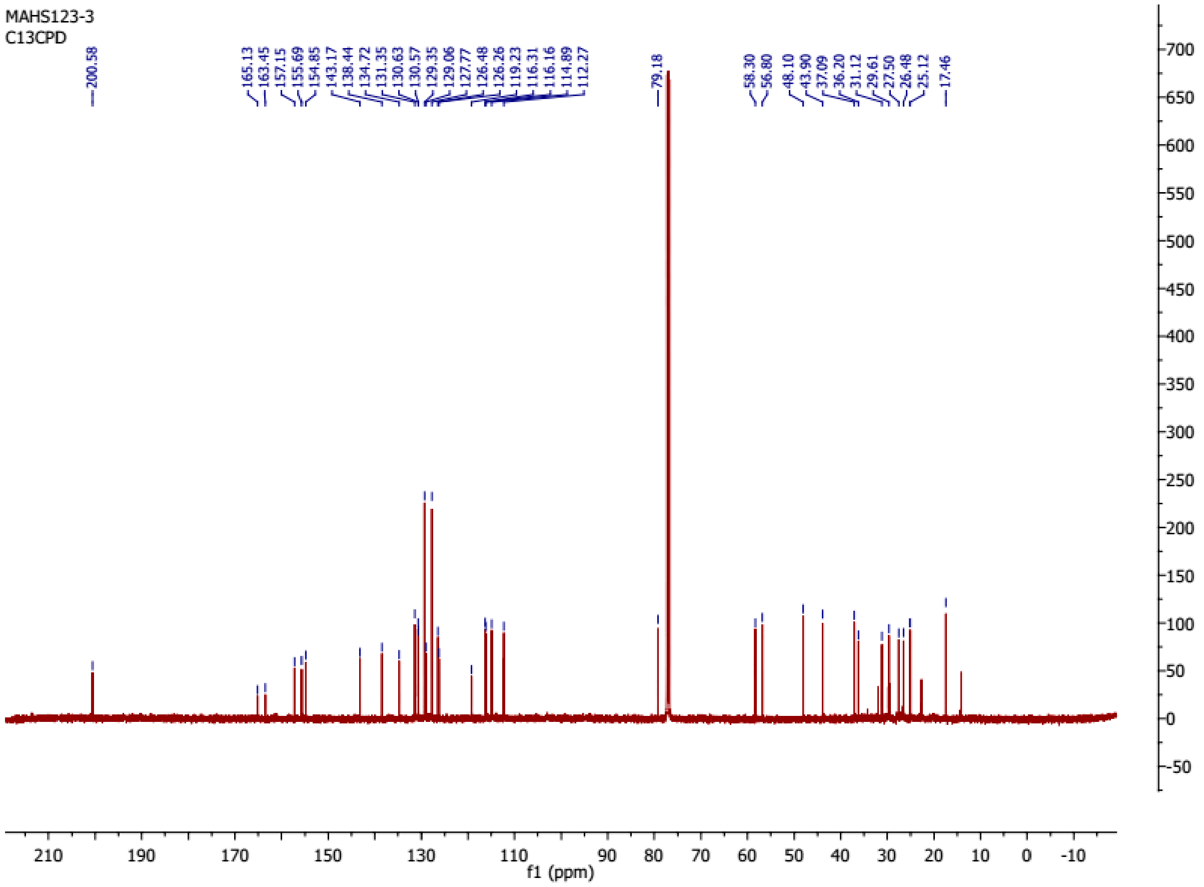

Figure S 95. ^{13}C NMR of compound **20b**.

1.47 Compound 20c

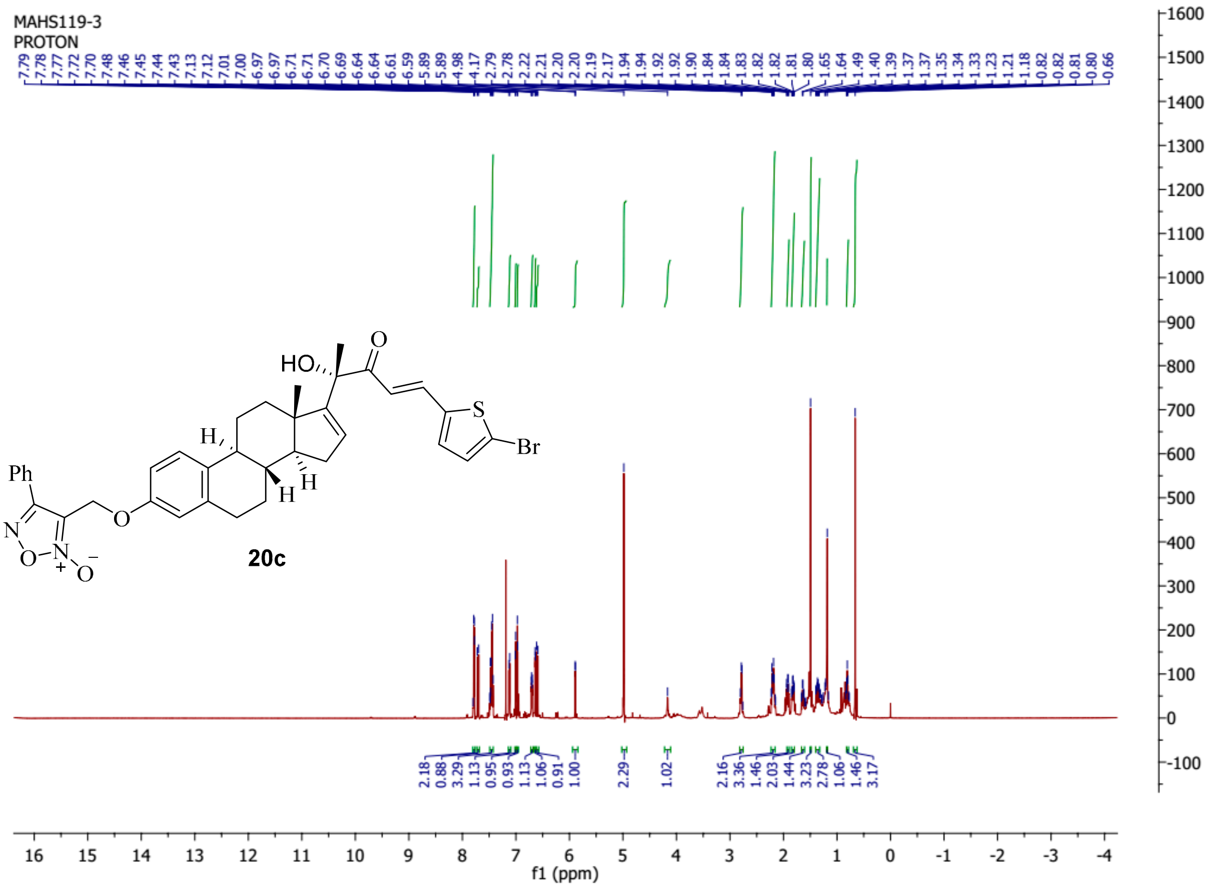

Figure S 96. ^{1}H NMR of compound **20c**.

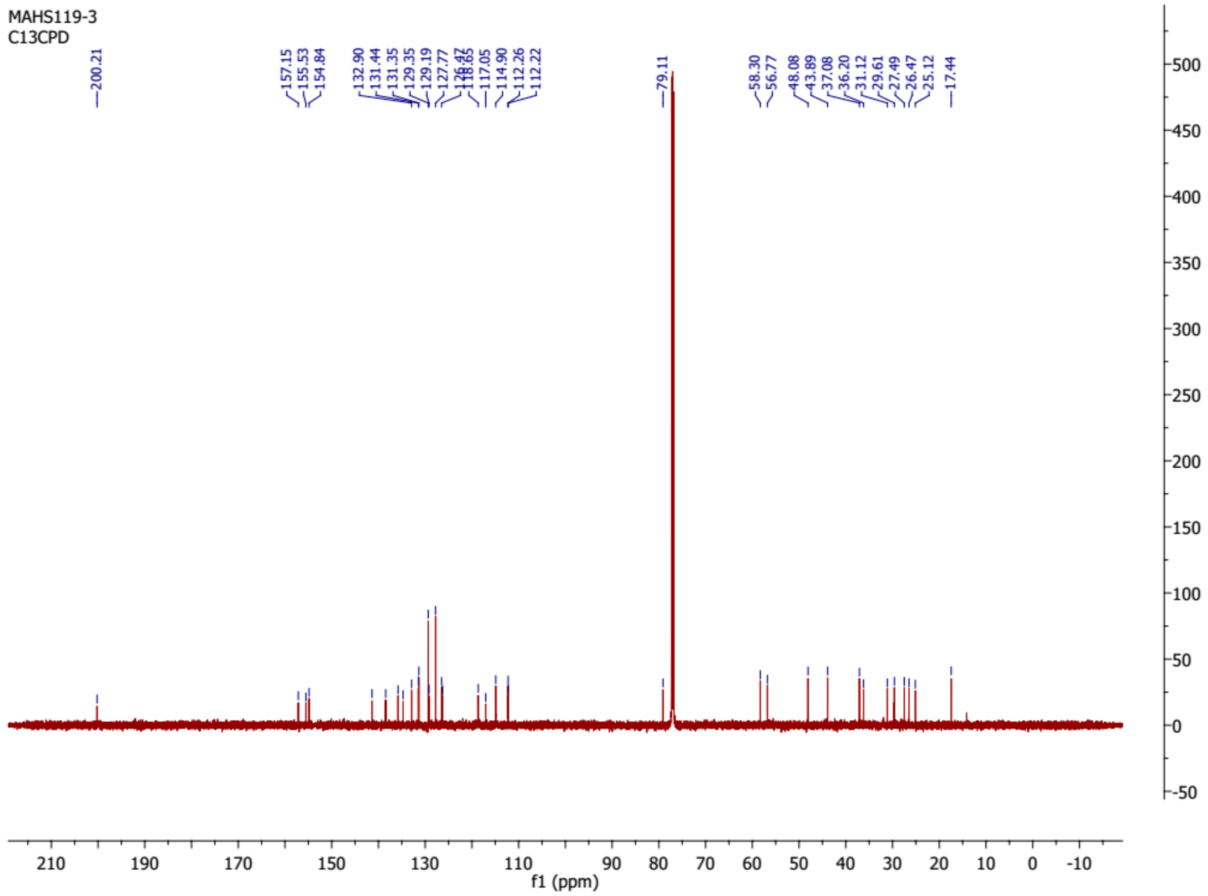

Figure S 97. ^{13}C NMR of compound **20c**.

1.48 Compound 20d

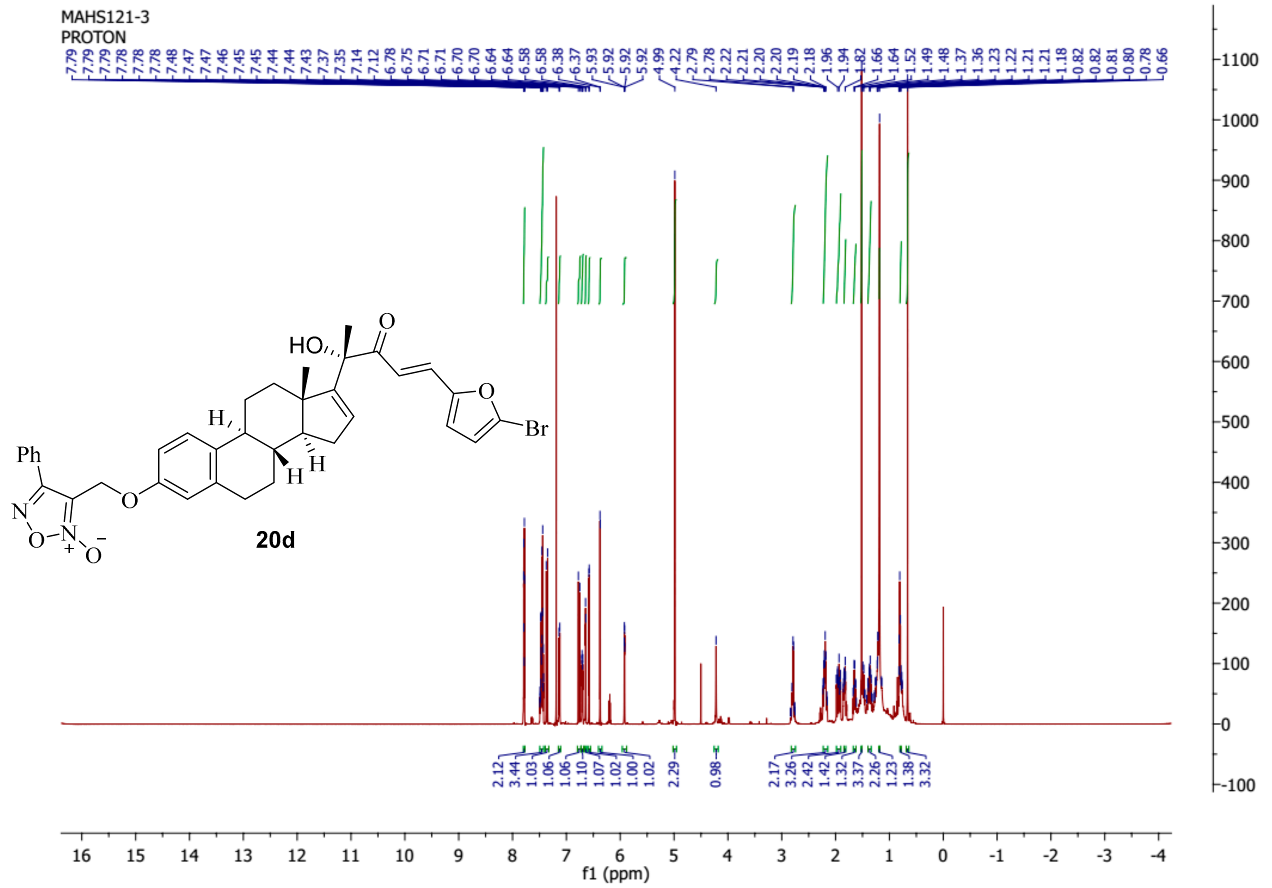

Figure S 98. ^{1}H NMR of compound **20d**.

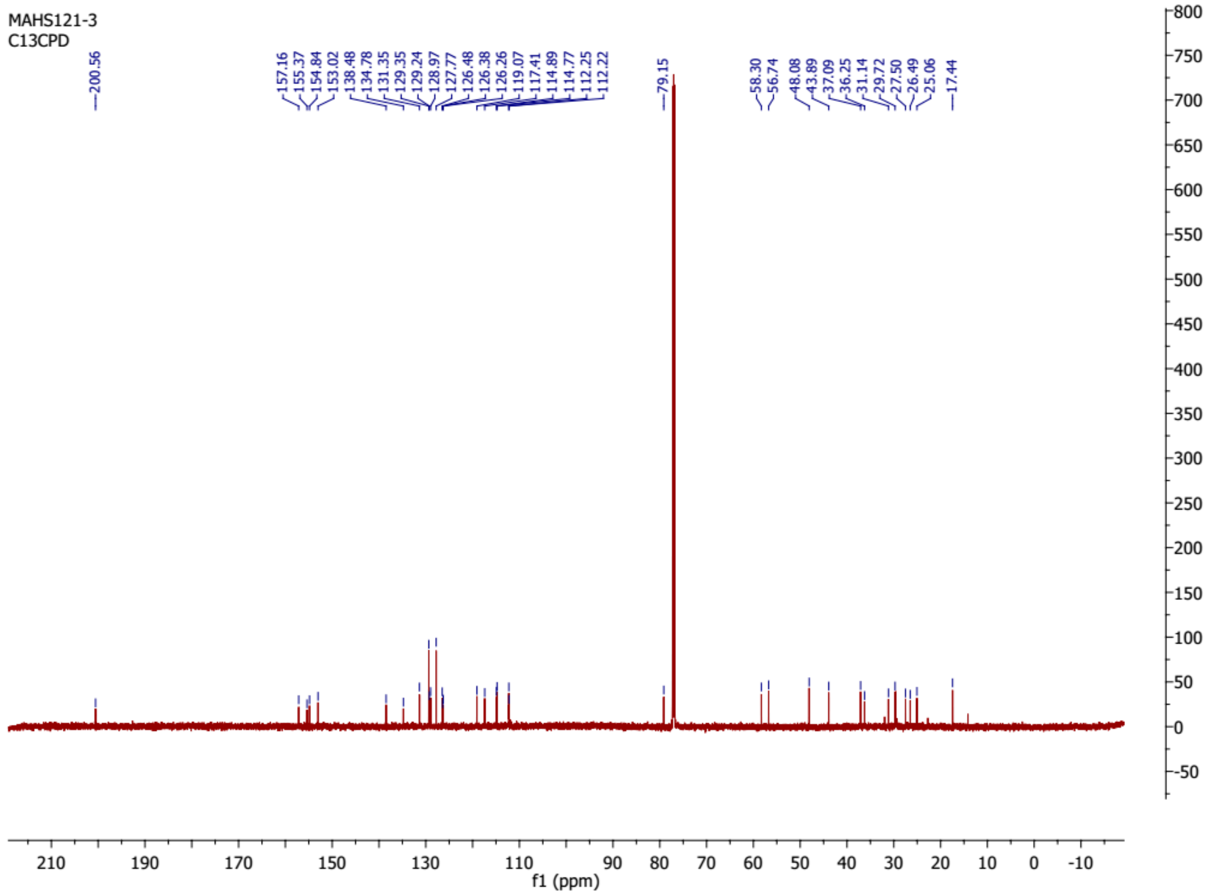

Figure S 99. ^{13}C NMR of compound **20d**.
